# Supplementary material for: FOXK2 in skeletal muscle development: a new pathogenic gene for congenital myopathy with ptosis
Source: EMBO Mol Med. 2025 May 23;17(7):1599–630. doi: 10.1038/s44321-025-00247-x (PMC12254393; doi:10.1038/s44321-025-00247-x)
Supplement: Supplementary file 1 — Appendix [file 44321_2025_247_MOESM1_ESM.pdf]

Appendix Figure

Appendix Figure S1. Generation of muscle stem cells-specific *Foxk2* conditional knockout mice (Page 2)

Appendix Figure S2. Generation of global *Foxk2* knockout mice and their gross phenotypes (Page 4)

Appendix Figure S3. Skeletal muscle dysplasia in global *Foxk2* knockout mice (Page 6)

Appendix Figure S4. The expression of FOXK1 in mouse hindlimb muscle and C2C12 cells (Page 8)

Appendix Table

Appendix Table S1. List of primer, single guide RNA and morpholino sequences (Page 9)

Appendix Table S2. List of antibodies (Page 10)

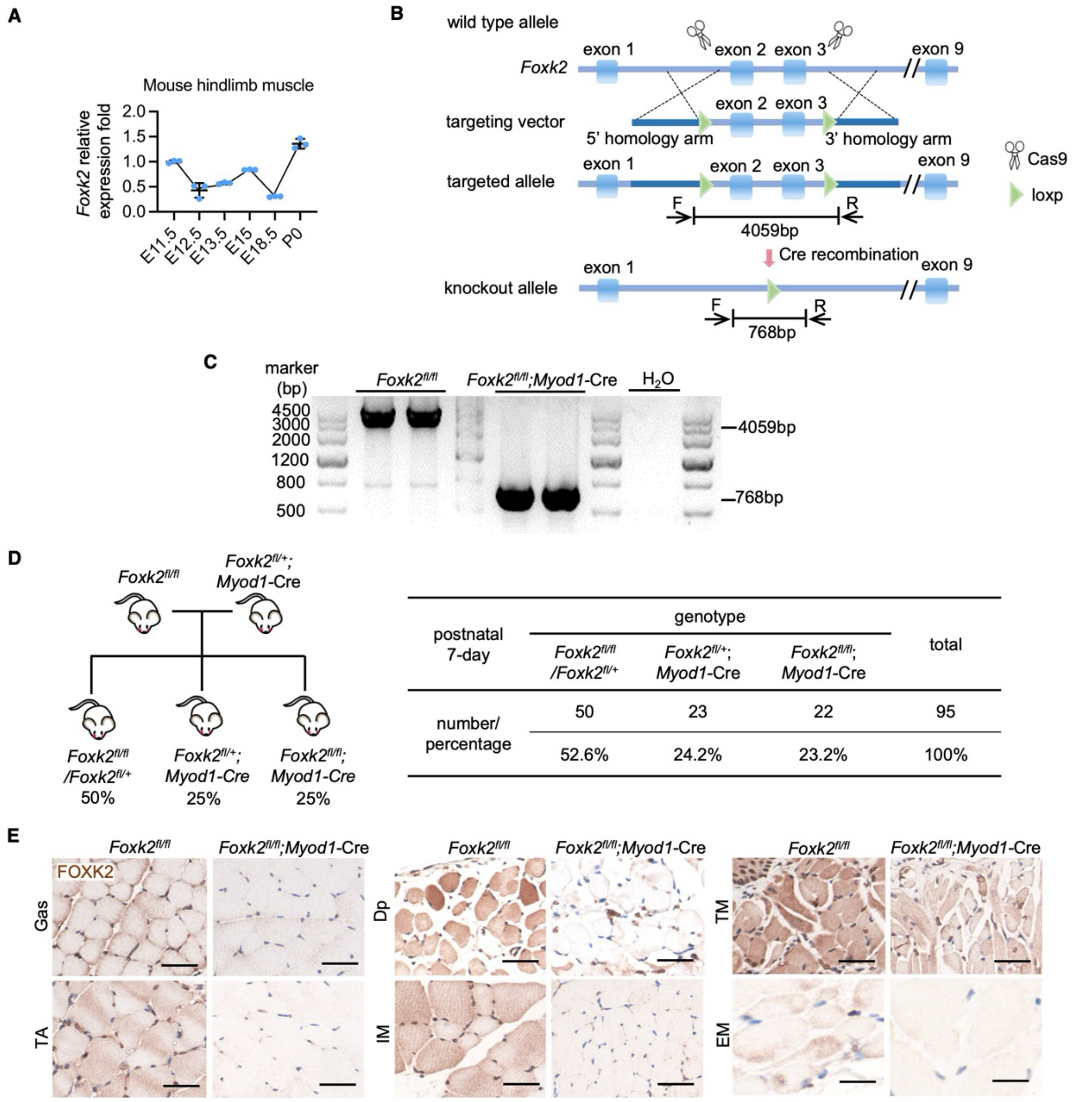

Appendix Figure S1. Generation of muscle stem cells-specific *Foxk2* conditional knockout mice

A. Quantitative of *Foxk2* expression level in hindlimb muscle of wild-type mouse by qPCR (n=3 repeats).

B. Generation of MuSCs-specific *Foxk2* conditional knockout mice using the Cre-loxP system, as described in the Methods.

C. Gel electrophoresis image showing the action of the Cre enzyme on the TA of *Foxk2*$^{fl/fl}$ and *Foxk2*$^{fl/fl}$; *Myod1*-Cre littermates. DEPC water served as the negative control.

D. Genotype distribution of individuals at postnatal day 7 resulting from the crossing of *Foxk2*$^{fl/fl}$ and *Foxk2*$^{fl/+}$; *Myod1*-Cre mice.

E. IHC staining of FOXK2 demonstrating the knockout efficiency in the skeletal muscles (Gas, TA, Dp, IM, TM, EM) of *Foxk2*$^{fl/fl}$; *Myod1*-Cre littermate at 8 weeks. Scale bars: 50 μm for Gas, TA, Dp, IM, and TM; 25 μm for EM.

All error bars indicate mean ± standard deviation.

Abbreviations: F, forward primer; R, reverse primer; Gas, gastrocnemius; TA, tibialis anterior; Dp, diaphragm; IM, intercostal muscle; TM, tongue muscle; EM, eyelid muscle; qPCR, quantitative real-time polymerase chain reaction; MuSCs, muscle stem cells; IHC, immunohistochemistry.

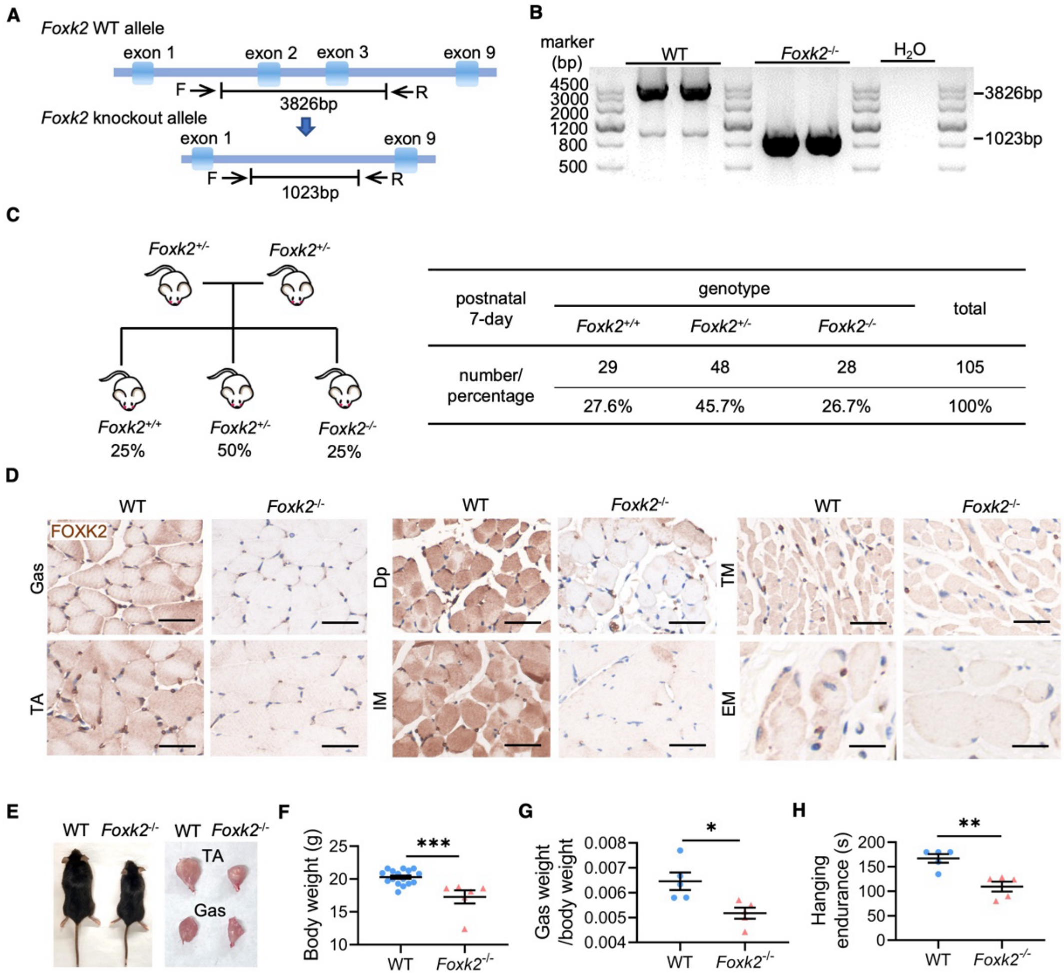

Appendix Figure S2. Generation of global *Foxk2* knockout mice and their gross phenotypes

A. Schematic diagram illustrating the generation of global *Foxk2*-knockout mice generation.

B. Gel electrophoresis image showing the knockout effect in mouse tail of WT and *Foxk2*$^{-/-}$ littermates. DEPC water served as the negative control.

C. Genotype distribution of individuals at postnatal day 7 resulting from *Foxk2*$^{+/-}$ and *Foxk2*$^{+/-}$ crosses.

D. IHC staining of FOXK2 demonstrating *Foxk2* knockout efficiency in skeletal muscles (Gas, TA, Dp, IM, TM, and EM) of WT and *Foxk2*$^{-/-}$ littermates at 8 weeks. Scale bars: 50 µm for Gas, TA, Dp, IM, and TM; 25 µm for EM.

E. Representative images of WT and *Foxk2*$^{-/-}$ littermates at 8 weeks, including their TA and Gas muscle.

F. Body weight of mice of WT and *Foxk2*$^{-/-}$ mice at 8 weeks (17 WT vs. 6 *Foxk2*$^{-/-}$ mice). *P* value: ***P=0.0004

G. Ratio of Gas muscle weight to body weight in WT and *Foxk2*$^{-/-}$ mice at 8 weeks (n=5 mice each group). *P* value: *P=0.0146.

H. Hanging endurance time measured by the hanging endurance test in WT and *Foxk2*$^{-/-}$ mice at 8 weeks (n=5 mice each group). *P* value: **P=0.0027.

Data were analyzed by Student's t test. All error bars indicate mean ± standard deviation.

Abbreviations: WT, wild type; F, forward primer; R, reverse primer; Gas, gastrocnemius; TA, tibialis anterior; Dp, diaphragm; IM, intercostal muscle; TM, tongue muscle; EM, eyelid muscle; IHC, immunohistochemistry.

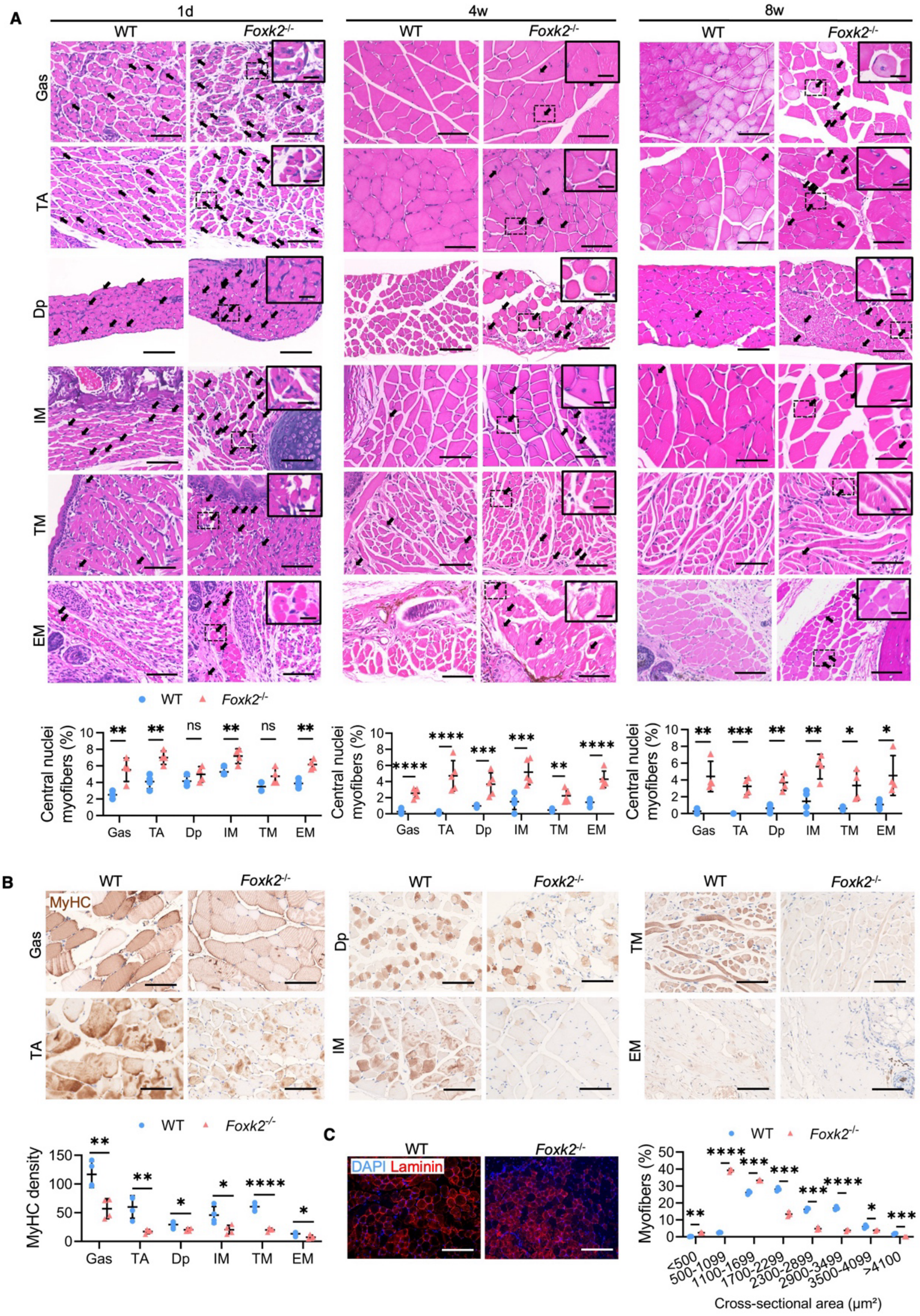

Appendix Figure S3. Skeletal muscle dysplasia in global *Foxk2* knockout mice

A. H&E staining illustrating histological aspects and the percentage of central nuclei myofibers in skeletal muscles (Gas, TA, Dp, IM, TM, EM) of WT and *Foxk2*$^{-/-}$ littermates at postnatal day 1, 4-weeks, and 8-weeks (n=4 areas). Black arrows

indicate central nuclei. The inset in the upper right corner (scale bars: 25 μm) is an enlarged view of the boxed area in the main image, highlighting central nuclei myofibers (scale bars: 100 μm). *P* value: 1d: Gas, $**P=0.0064$; TA, $**P=0.0026$; Dp, ns-$P=0.1867$; IM: $**P=0.0095$; TM, ns-$P=0.0667$; EM, $**P=0.0010$. 4w; $****P<0.0001$; $**P=0.0054$; Dp, $***P=0.0005$; IM, $***P=0.0009$. 8w: Gas, $**P=0.0040$; TA, $***P=0.0006$; Dp, $**P=0.0012$; IM, $**P=0.0054$; TM, $*P=0.0218$; EM, $*P=0.0297$.

B. IHC staining of MyHC in skeletal muscles (Gas, TA, Dp, IM, TM, EM) of WT and *Foxk2*$^{-/-}$ littermates at 8 weeks (n=4 areas). MyHC was quantified by InDen/Area using ImageJ software. Scale bars: 100 μm. *P* value: Gas, $**P=0.0059$; TA, $**P=0.0050$; Dp, $*P=0.0326$; IM: $*P=0.0238$; TM, $****P<0.0001$; EM, $*P=0.0435$.

C. IF staining of Laminin was performed on the TA of WT and *Foxk2*$^{-/-}$ littermates at 8 weeks. The distribution of cross-sectional area of myofibers was calculated (n=3 areas) (scale bars: 200 μm). *P* value: $****P<0.0001$; $**P=0.0022$; $*P=0.0219$; 1100-1699, $***P=0.0004$; 1700-2299, $***P=0.0002$; 2300-2899, $***P=0.0002$; >4100, $***P=0.0009$.

Data were analyzed by Student's t test. All error bars indicate mean ± standard deviation. Abbreviations: d, day; w, week; Gas, gastrocnemius; TA, tibialis anterior; Dp, diaphragm; IM, intercostal muscle; TM, tongue muscle; EM, eyelid muscle; H&E, hematoxylin-eosin staining; IHC, immunohistochemistry; IF, immunofluorescence.

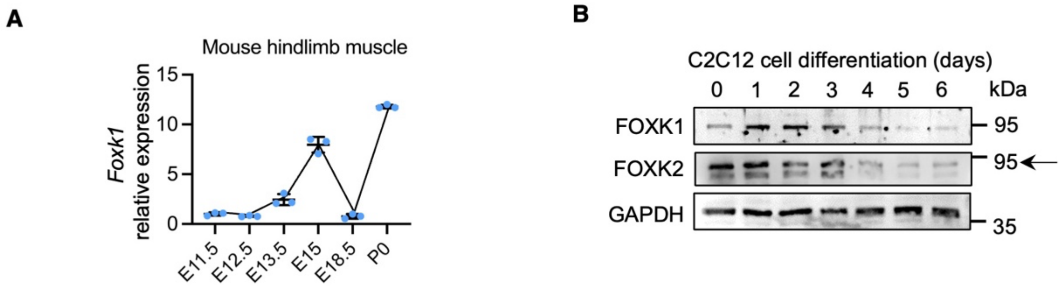

Appendix Figure S4. The expression of FOXK1 in mouse hindlimb muscle and C2C12 cells

A. Quantitative analysis of *Foxk1* expression levels in the hindlimb muscle of wild-type mice using qPCR (n=3 repeats).

B. Western blot analysis of FOXK1 and FOXK2 expression levels in C2C12 cells. The cells were induced to undergo myogenic differentiation, and whole-cell lysates were isolated at the indicated times for analysis. The black arrows point to specific protein bands.

All error bars indicate mean ± standard deviation.

Abbreviations: qPCR, quantitative real-time polymerase chain reaction.

Appendix Table Legends

Appendix Table S1. List of primer, single guide RNA and morpholino sequences

**primers for quantitative real-time polymerase chain reaction**		
gene name	Forward (5’-3’)	Reverse (5’-3’)
zebrafish *foxk2*	GGTTCCCCAGTACGAACATC	GGACTGGATGGACACGAGTT
zebrafish *ef1a*	GGAAATTCGAGACCAGCAAATAC	GATACCAGCCTCAAACTCACC
Mouse *Foxk2*	TCTCACCCCTGACCATCAAC	CCACCIGACGCTCCTTTTC
Mouse *Tfb2m*	CCAAAACCCATCCCGTCAAAT	AAGGGCTCCAAATGTGGAATAAA
Mouse *Cycs*	GCAAGCATAAGACTGGACCAAA	TTGTTGGCATCTGTGTAAGAGAATC
Mouse *Cox4l*	ACCAAGCGAATGCTGGACAT	GGCGGAGAAGCCCTGAA
Mouse *Myog*	CGCCATCCAGTACATTGAGC	GACCGAACTCCAGTGCATTG
Mouse *Myod1*	CACCGCCTACTACAGTGAGG	GTCTGGGTTCCCTGTTCTGT
Mouse *Myf5*	GCTTTCGAGACGCTCAAGAG	AAAGCTGCTGTTCTTTCGGG
Mouse *Pax7*	CATGAACCCTGTCAGCAATG	CACTGTAGCCAGTGGTGCTG
Mouse *Myh4*	AGCTTGAAAACGAGGTGGAA	CCTCCTCAGCCTGTCTCTTG
Mouse *Pgc1a*	AAGTGTGGAACTCTCTGGAACTG	GGGTTATCTTGGTTGGCTTTATG
Mouse *Tfb1m*	AAGATGGCCCTTTCGTTTATGG	GACTGTGCTGTTTGCTTCCTG
Mouse *Foxk1*	ACCCACGAATAGCTTGACTGG	GCATTAGCGGCTACTGAGACG
Mouse *Gapdh*	GGGTCCCAGCTTAGGTTCAT	CATTCTCGGCCTTGACTGTG
Mouse *Mt-Co2*	GCCGACTAAATCAAGCAACA	CAATGGGCATAAAGCTATGG
Mouse *Cry1*	CACTGGTTCCGAAAGGGACTC	CTGAAGCAAAAATCGCCACCT
Mouse *Pdk4*	AGGGAGGTCGAGCTGTTCTC	GGAGTGTTCACTAAGCGGTCA
Human *GAPDH*	GGAGCGAGATCCCTCCAAAAT	GGCTGTTGTCATACTTCTCATGG
Human *CRY1*	CTCCTCCAATGTGGGCATCAA	CCACGAATCACAAACAGACGG

Human *PDK4*	GGAGCATTTCTCGCGCTACA	ACAGGCAATTCTTGTCGCAAA
**primers for mouse genotyping identification**		
Myod1-Cre	GCCGATCCGAATTCGAAGTTCC	TGGGTCTCCAAAGCGACTCC
Foxk2-loxP	TCAGAGGCAGCACAGGAGTA	AGCAAGTTCAAGGGCAACCT
Foxk2-loxp for verifying	ATTGATTTCCATGGGGCGGT	GTTCTGCTCACTCCATGGCT
Foxk2 knockout-1	GTAGGGTAGAGGCCAGTGGA	CCACAGCAACTCCCTTCTGT
Foxk2 knockout-2	ATTGATTTCCATGGGGCGGT	CTTCTCGCTGGATAGGGCTG
Foxk2 knockout for verifying	GTAGGGTAGAGGCCAGTGGA	CCACAGCAACTCCCTTCTGT
**morpholinos (5'-3')**		
ATG-MO	ATCGCTGCCATCTTTATCTTTTCGG	
I2E3-MO	GCGCTGAACACAGAGAACACAGAGA	
control-MO	CCTCTTACCTCAGTTACAATTTATA	
**single guide RNA (5'-3')**		
oligo 1	CACCGGGGGCGTGAAGATCTCCAGG	
oligo 2	AAACCCTGGAGATCTTCACGCCCCC	

The primer sequences for quantitative real-time polymerase chain reaction and mouse genotyping identification were listed here. The morpholinos for knockdown *foxk2* in zebrafish and single guide RNA for knockout FOXK2 in C2C12 cells were also listed below.

Appendix Table S2. List of antibodies

**antibody**	**company**	**reference**	**dilution**
Myosin heavy chain (MyHC)	Abmart	T56635	1:1000 for western blot<br>1:50 for immunohistochemistry
Myosin heavy chain (MyHC)	R&D systems	MAB4470	1:100 for immunofluorescence
FOXK2	Abmart	PU248547	1:500 for immunohistochemistry

FOXK2	Novas Biologicals	NBP1-87700	1:1000 for western blot
FOXK2	CST	28712T	1: 50 for chromatin immunoprecipitation sequencing
Laminin beta 1	proteintech	23498-1-AP	1:100 for immunofluorescence
DPR1	proteintech	12957-1-AP	1:200 for immunofluorescence<br>1:1000 for western blot
OPA1	proteintech	27733-1-AP	1:200 for immunofluorescence<br>1:1000 for western blot
MYOG	Abcam	ab77232	1:500 for western blot
MYOD1	proteintech	18943-1-AP	1:1000 for western blot
MYF5	Abmart	T58504	1:2000 for western blot
PAX7	DSHB	AB_528428	1:500 for western blot
GAPDH	proteintech	60004-1-Ig	1:50000 for western blot
FOXK1	Abcam	ab309510	1:2000 for western blot
PDK4	Abmart	TD7169	1:2000 for western blot<br>1:100 for immunohistochemistry
CRY1	Abmart	PC11441	1:2000 for western blot<br>1:100 for immunohistochemistry
Anti-rabbit secondary antibody	Jackson ImmunoResearch	111-035-003	1:5000 for western blot
YSFluor™ 488 Goat anti-rabbit secondary antibody	Yeasen	33106ES	1:200 for immunofluorescence

The company, reference information and the concentration of dilutions in the experiment of antibodies.
