# Supplementary figures and images for: FOXK2 in skeletal muscle development: a new pathogenic gene for congenital myopathy with ptosis (part 1 of 2)
Source: EMBO Mol Med. 2025 May 23;17(7):1599–630. doi: 10.1038/s44321-025-00247-x (PMC12254393; doi:10.1038/s44321-025-00247-x)

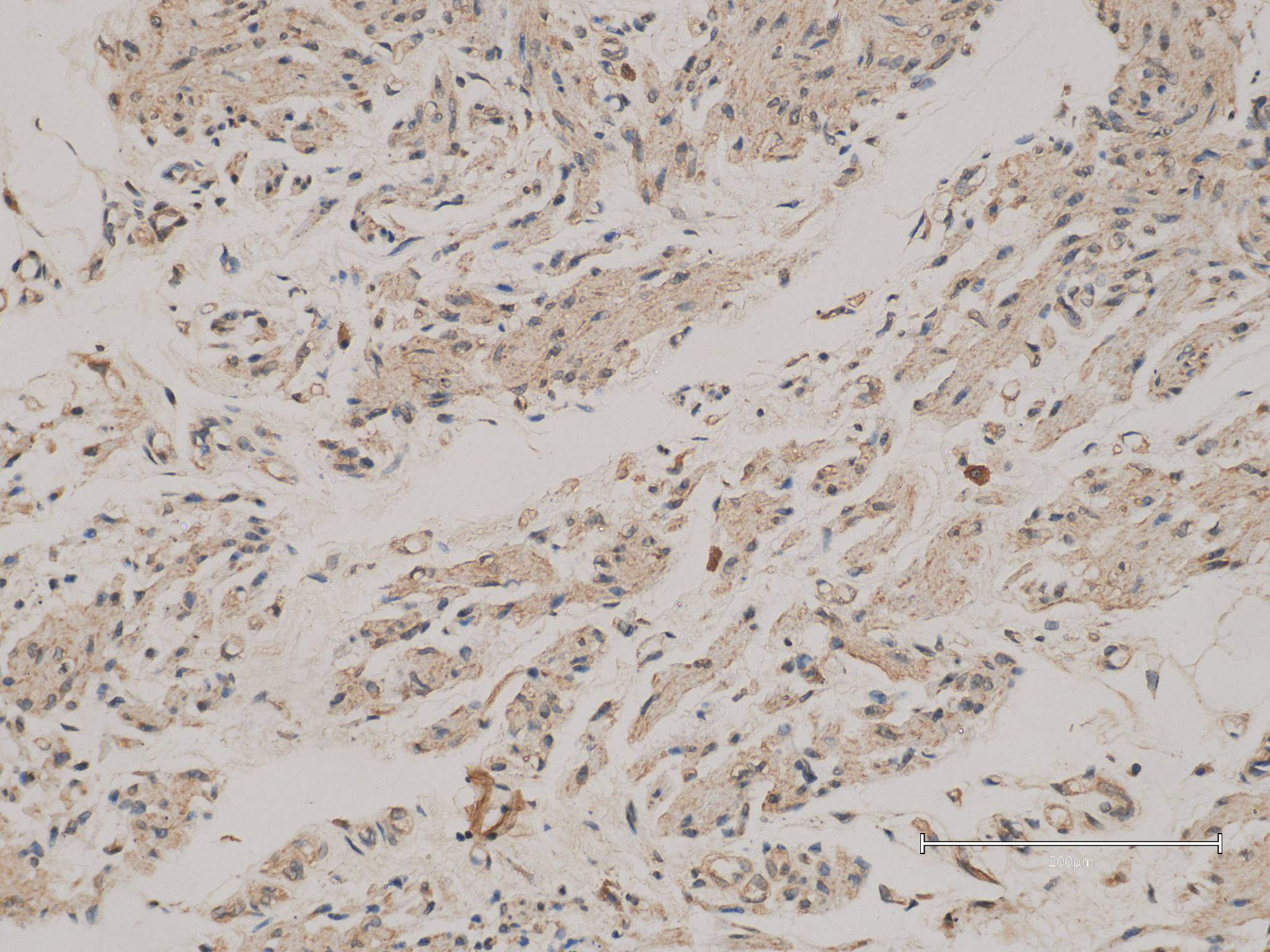

Supplement: Supplementary file 8 — Source data Fig. 1 [file 44321_2025_247_MOESM8_ESM.zip › Figure 1/Figure 1_Panel C/Figure 1_Panel C_IHC_MyHC_II-5.tif]

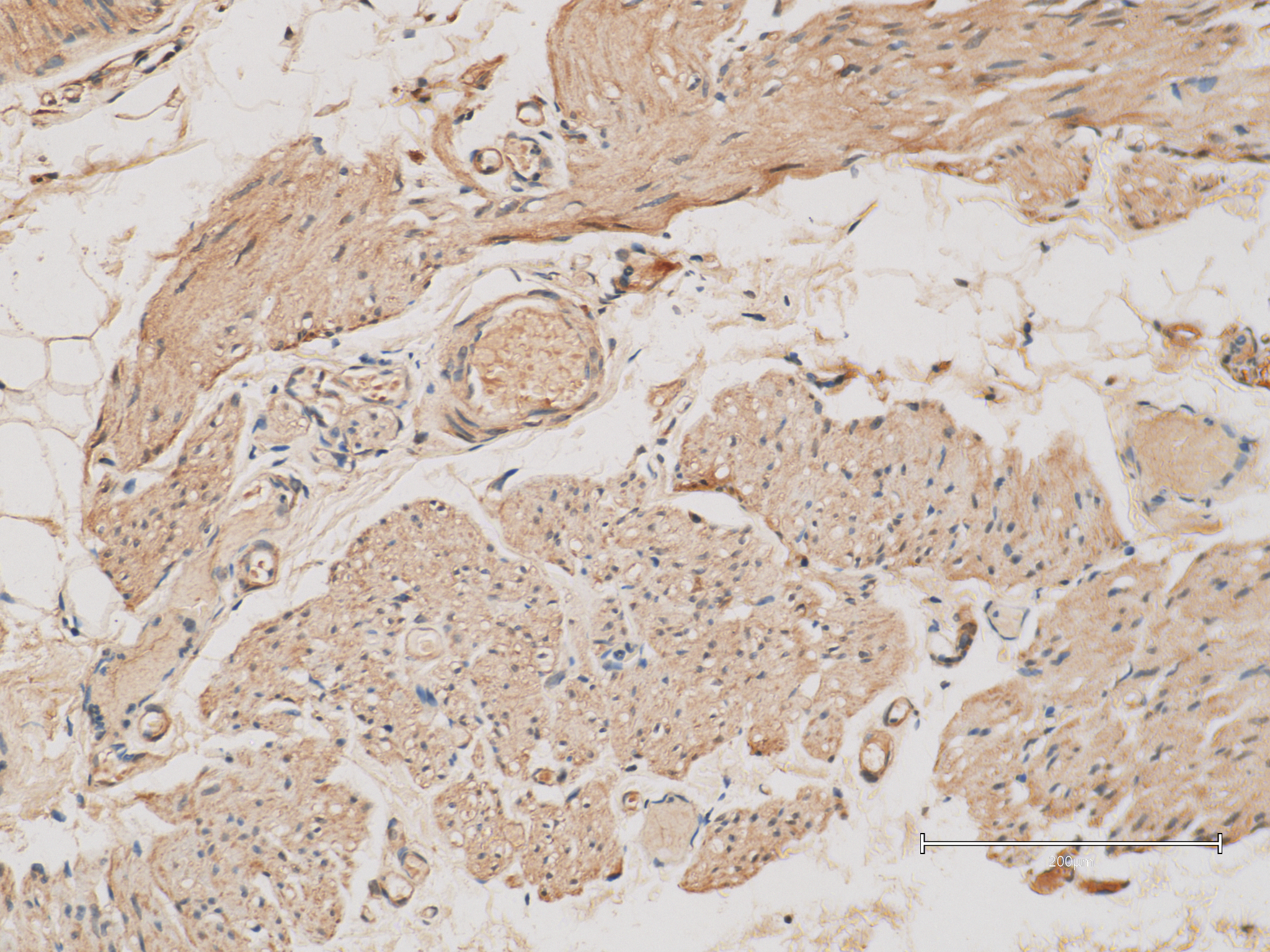

Supplement: Supplementary file 8 — Source data Fig. 1 [file 44321_2025_247_MOESM8_ESM.zip › Figure 1/Figure 1_Panel C/Figure 1_Panel C_IHC_MyHC_control.tif]

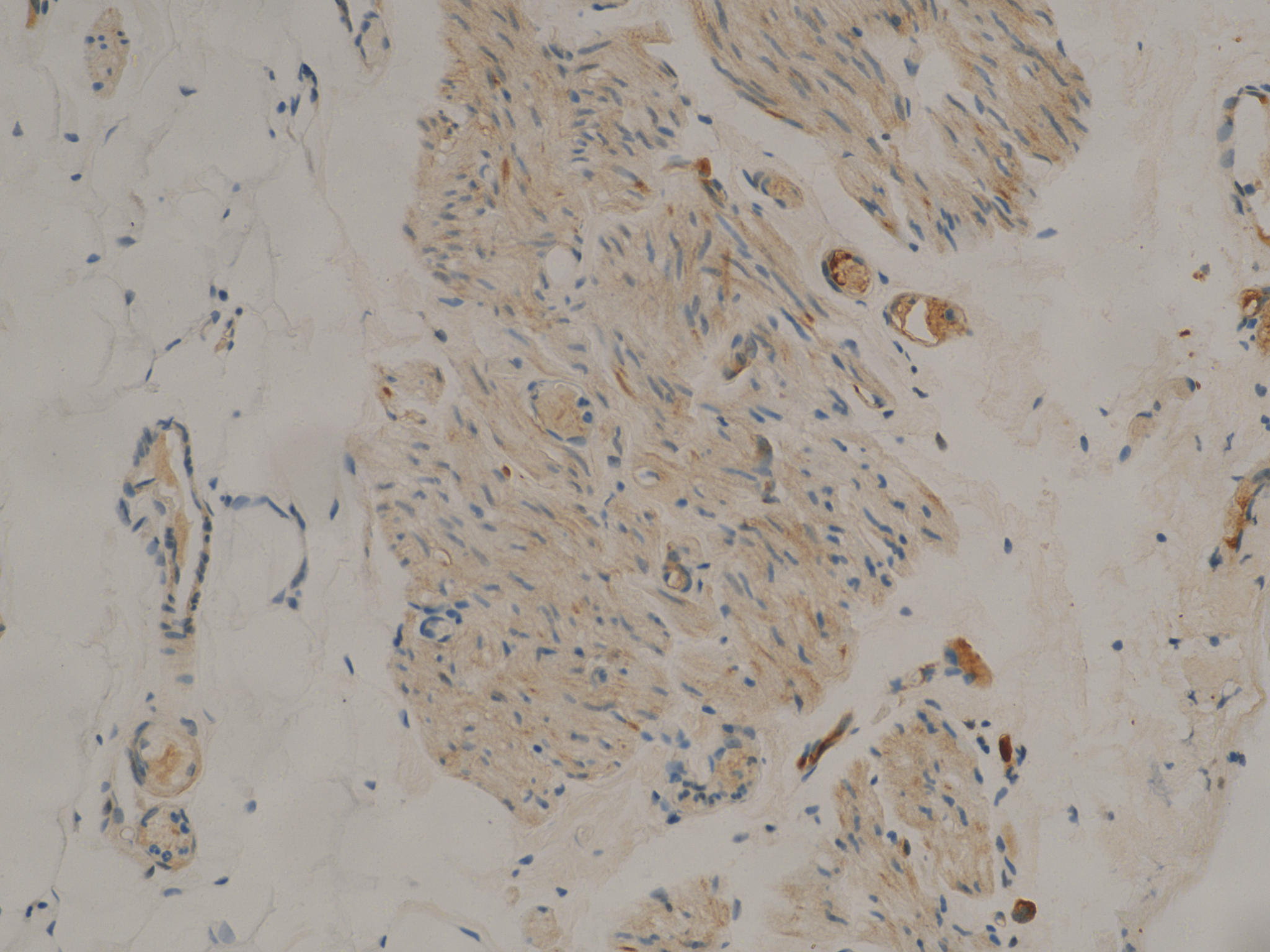

Supplement: Supplementary file 8 — Source data Fig. 1 [file 44321_2025_247_MOESM8_ESM.zip › Figure 1/Figure 1_Panel D/Figure 1_Panel D_IHC_FOXK2_control.tif]

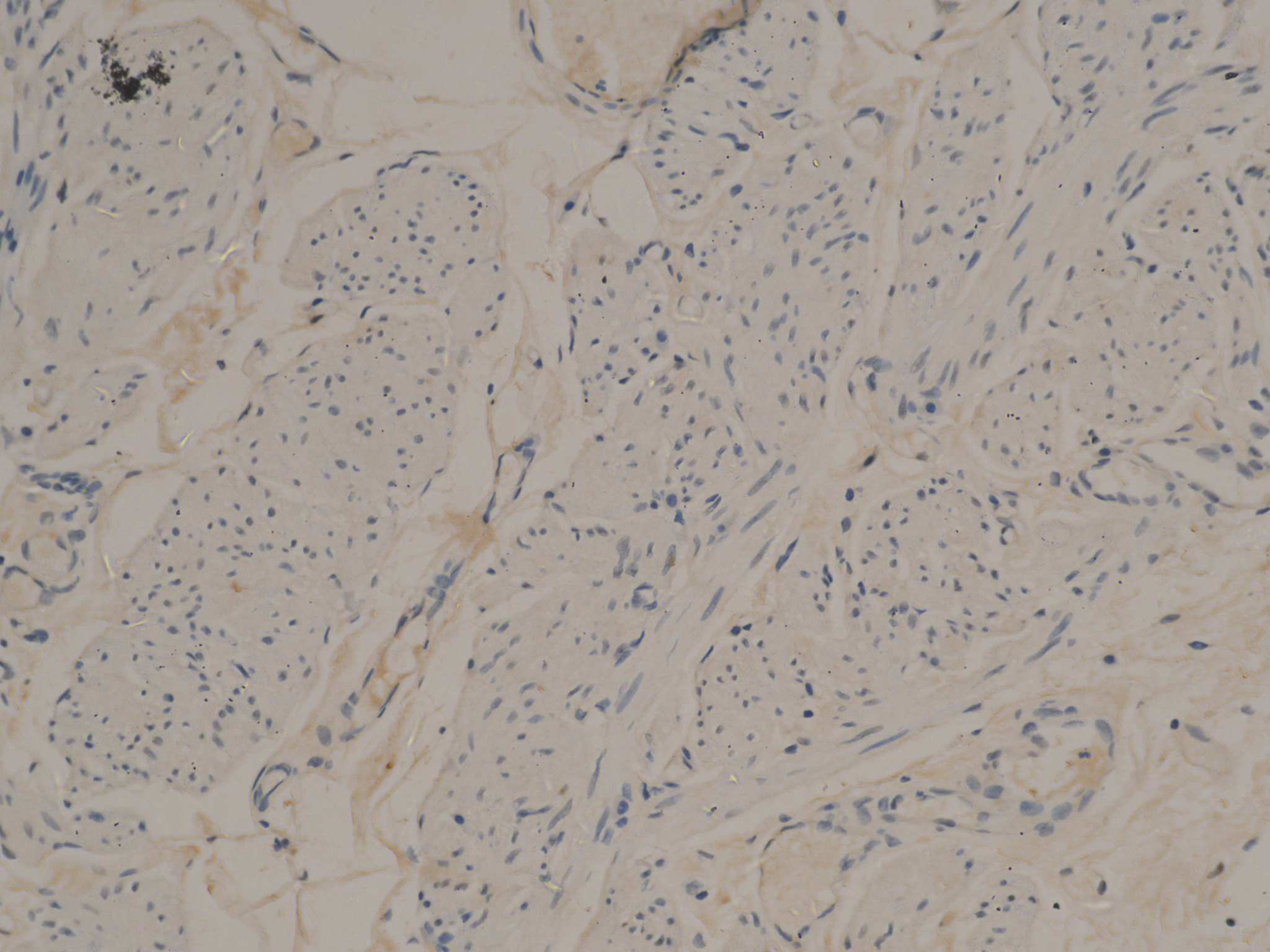

Supplement: Supplementary file 8 — Source data Fig. 1 [file 44321_2025_247_MOESM8_ESM.zip › Figure 1/Figure 1_Panel D/Figure 1_Panel D_IHC_FOXK2_II-5.tif]

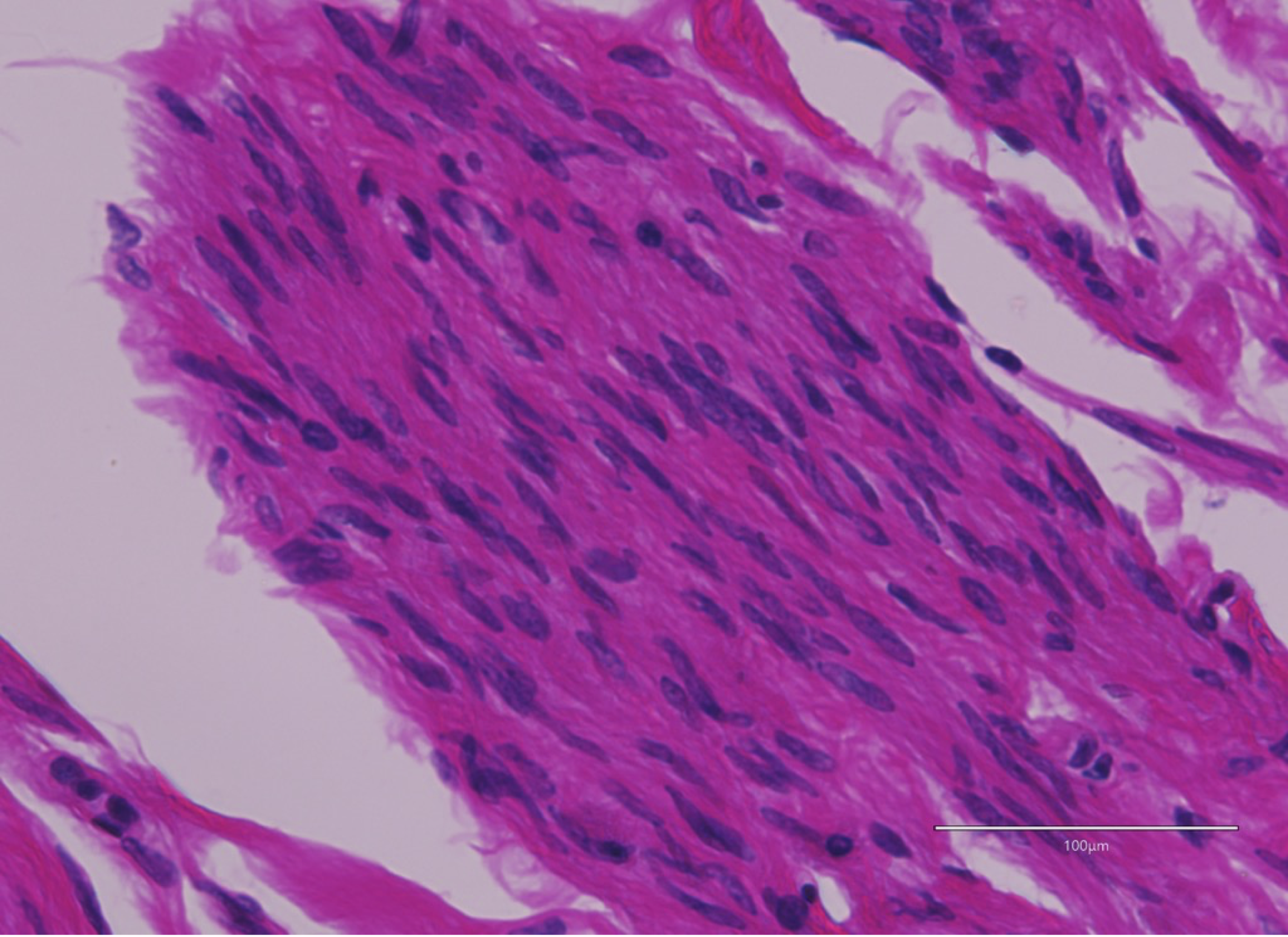

Supplement: Supplementary file 8 — Source data Fig. 1 [file 44321_2025_247_MOESM8_ESM.zip › Figure 1/Figure 1_Panel B/Figure 1_Panel B_HE_control.tif]

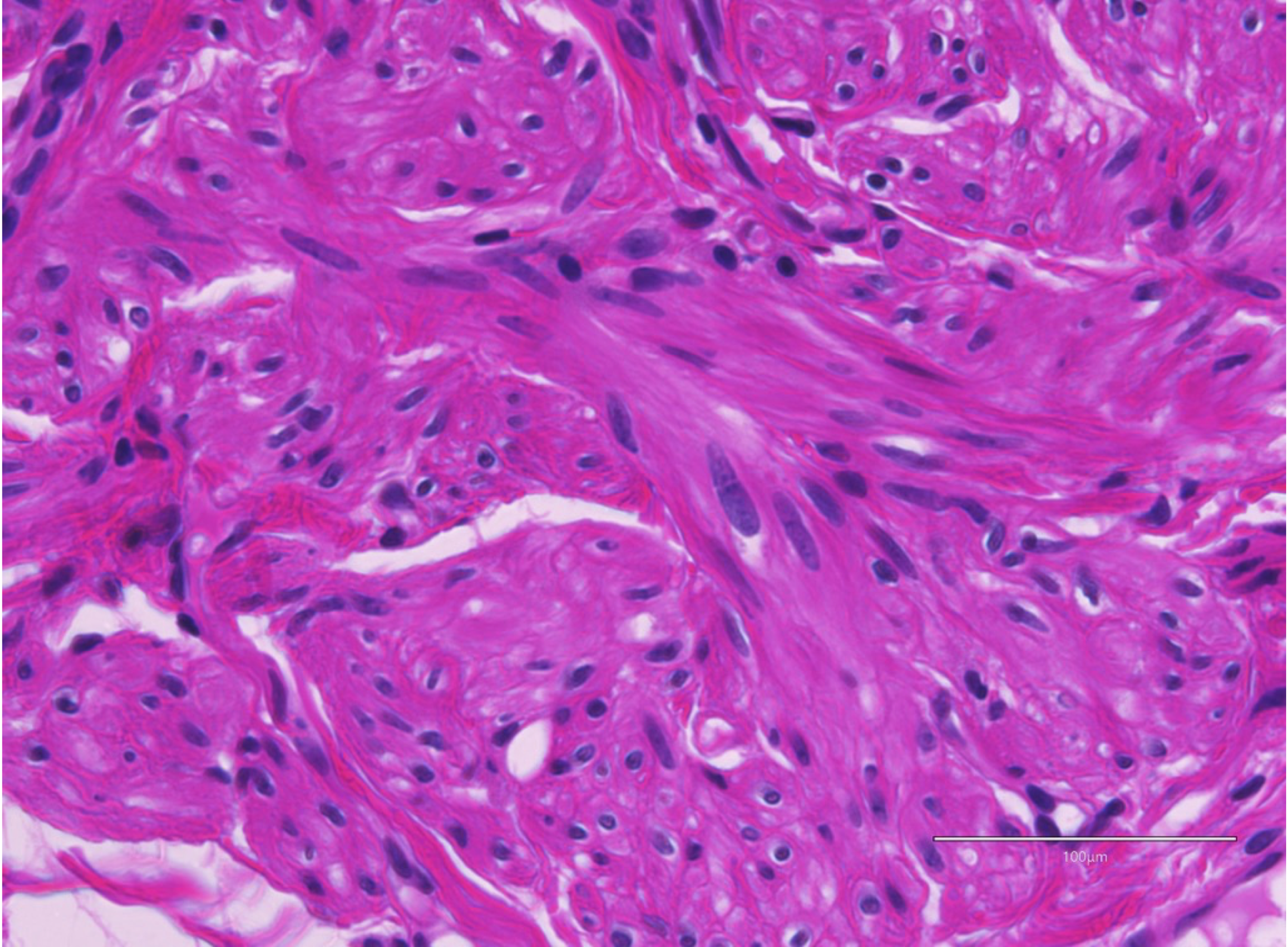

Supplement: Supplementary file 8 — Source data Fig. 1 [file 44321_2025_247_MOESM8_ESM.zip › Figure 1/Figure 1_Panel B/Figure 1_Panel B_HE_II-5.tif]

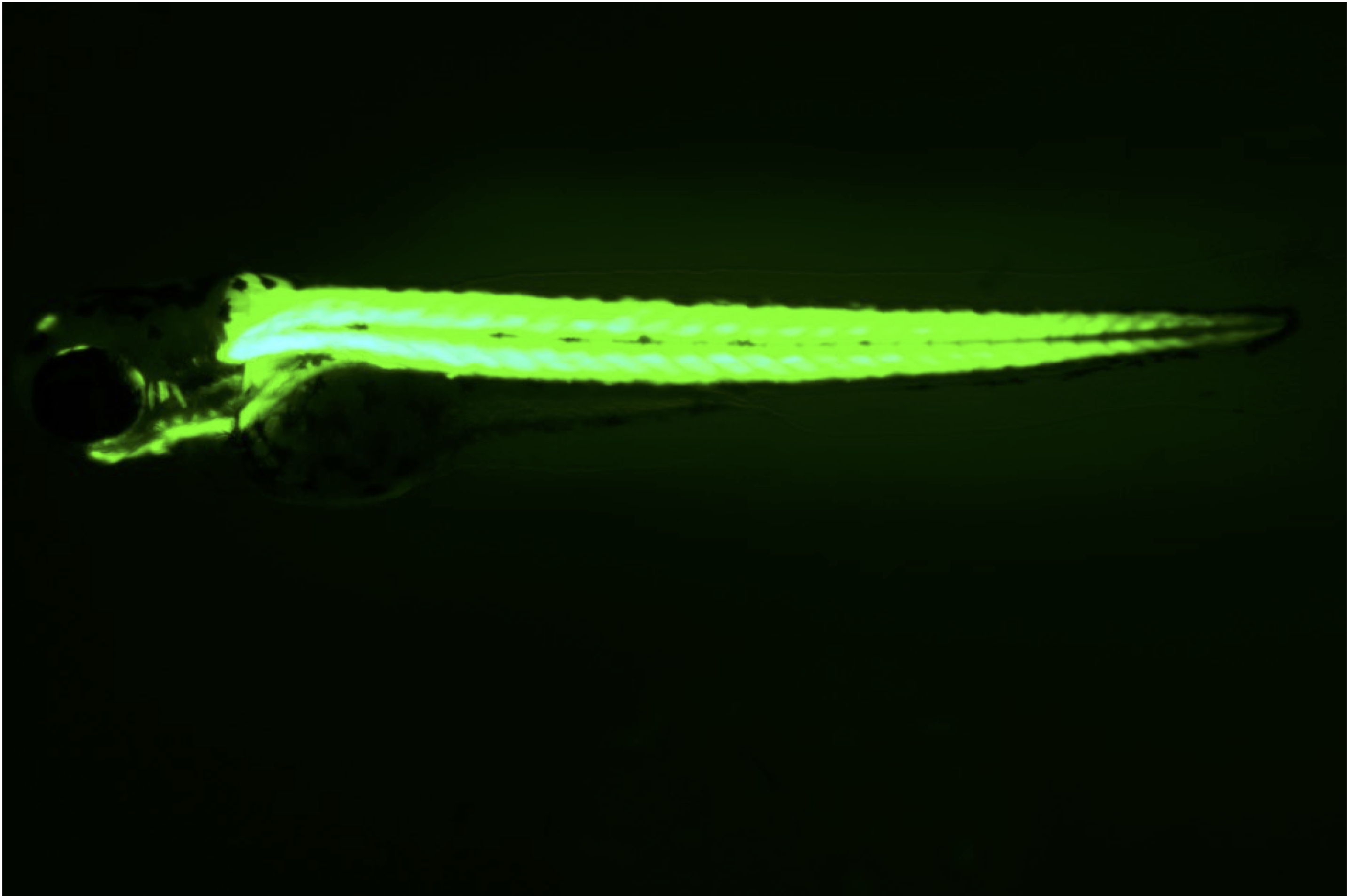

Supplement: Supplementary file 9 — Source data Fig. 2 [file 44321_2025_247_MOESM9_ESM.zip › Figure 2/Figure 2_Panel E/Figure 2_Panel E_control-MO_Tg(-1.9mylpfa-EGFP).tif]

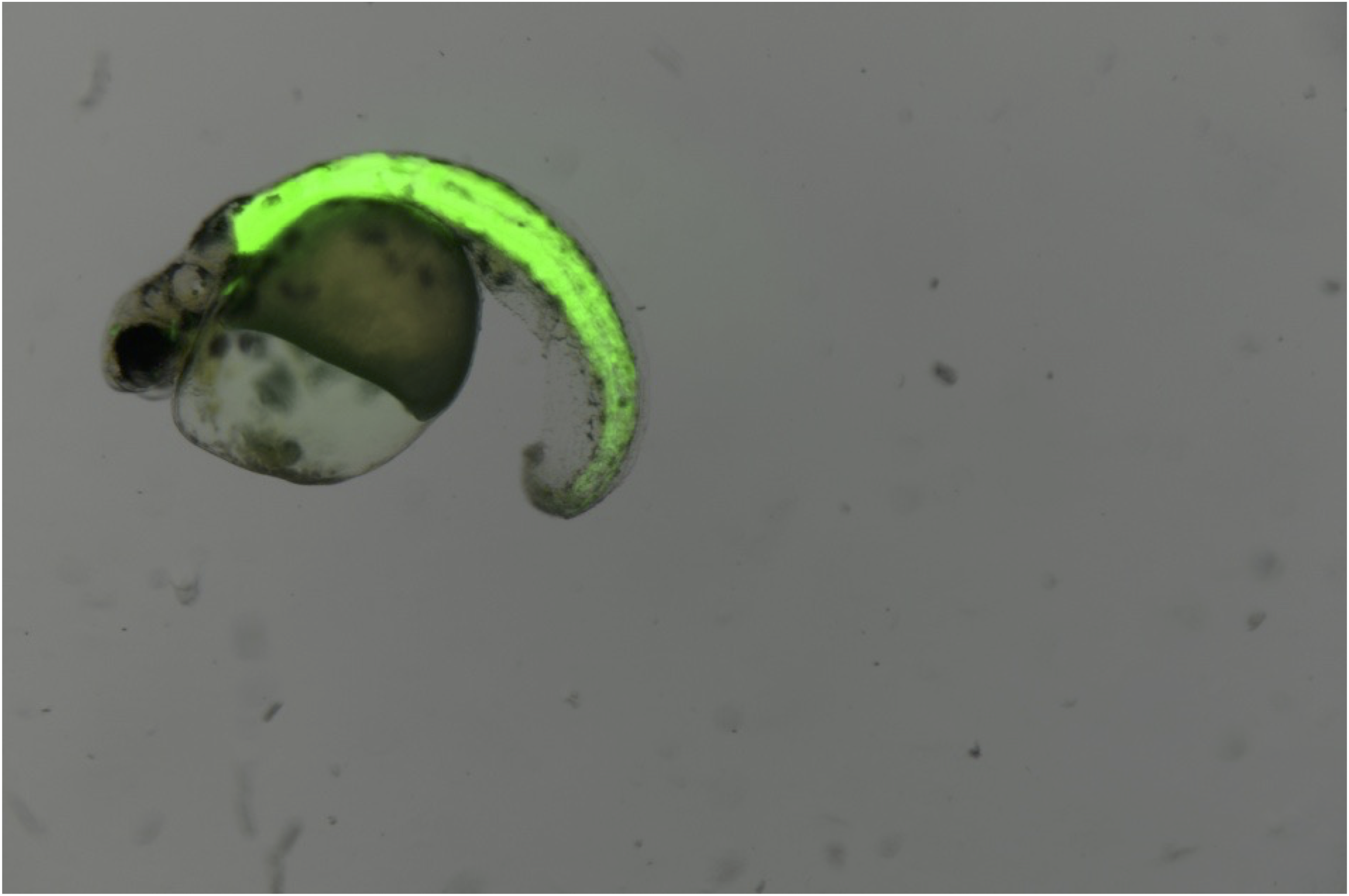

Supplement: Supplementary file 9 — Source data Fig. 2 [file 44321_2025_247_MOESM9_ESM.zip › Figure 2/Figure 2_Panel E/Figure 2_Panel E_ATG-MO_merge.tif]

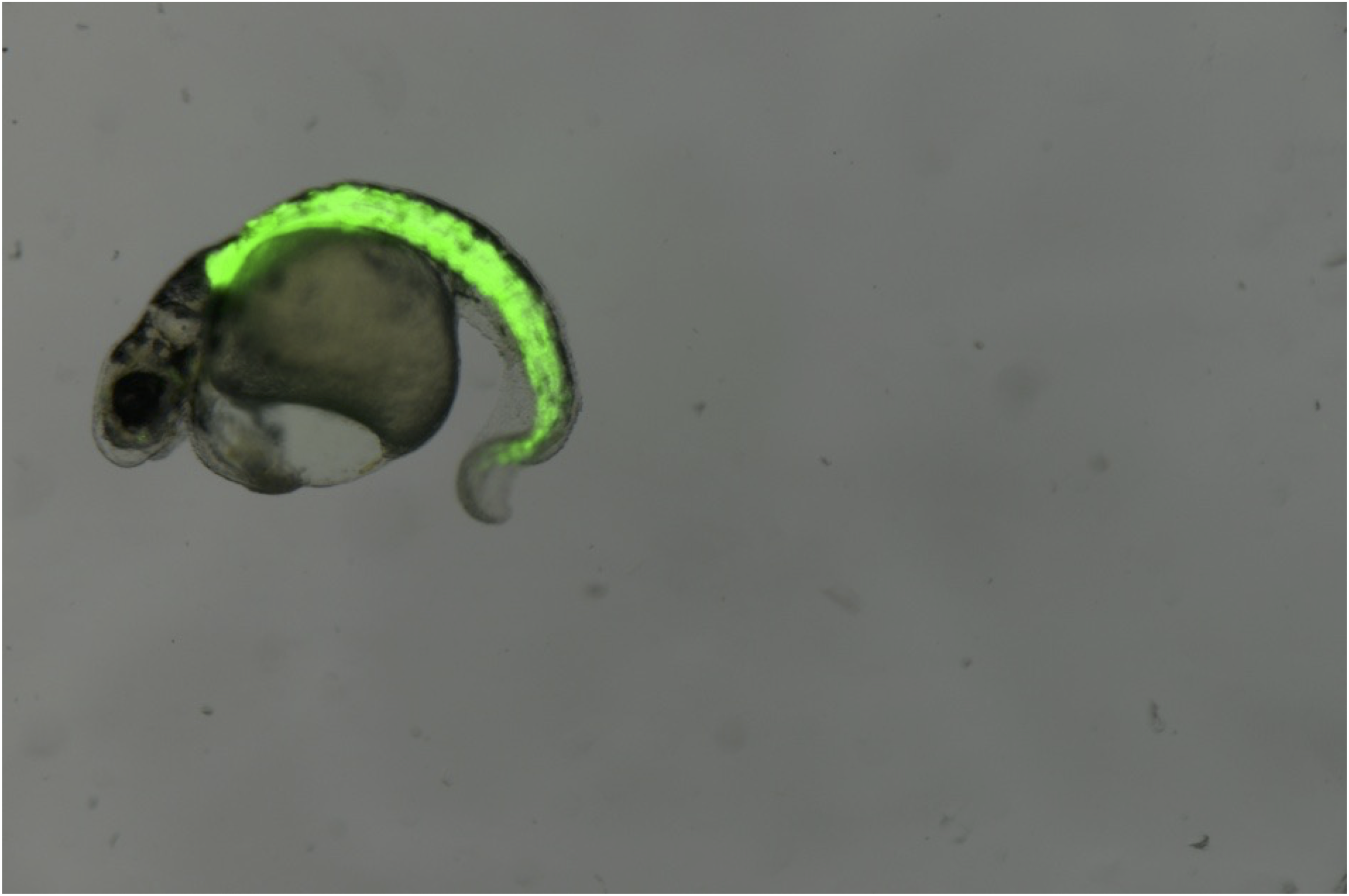

Supplement: Supplementary file 9 — Source data Fig. 2 [file 44321_2025_247_MOESM9_ESM.zip › Figure 2/Figure 2_Panel E/Figure 2_Panel E_I2E3-MO_merge.tif]

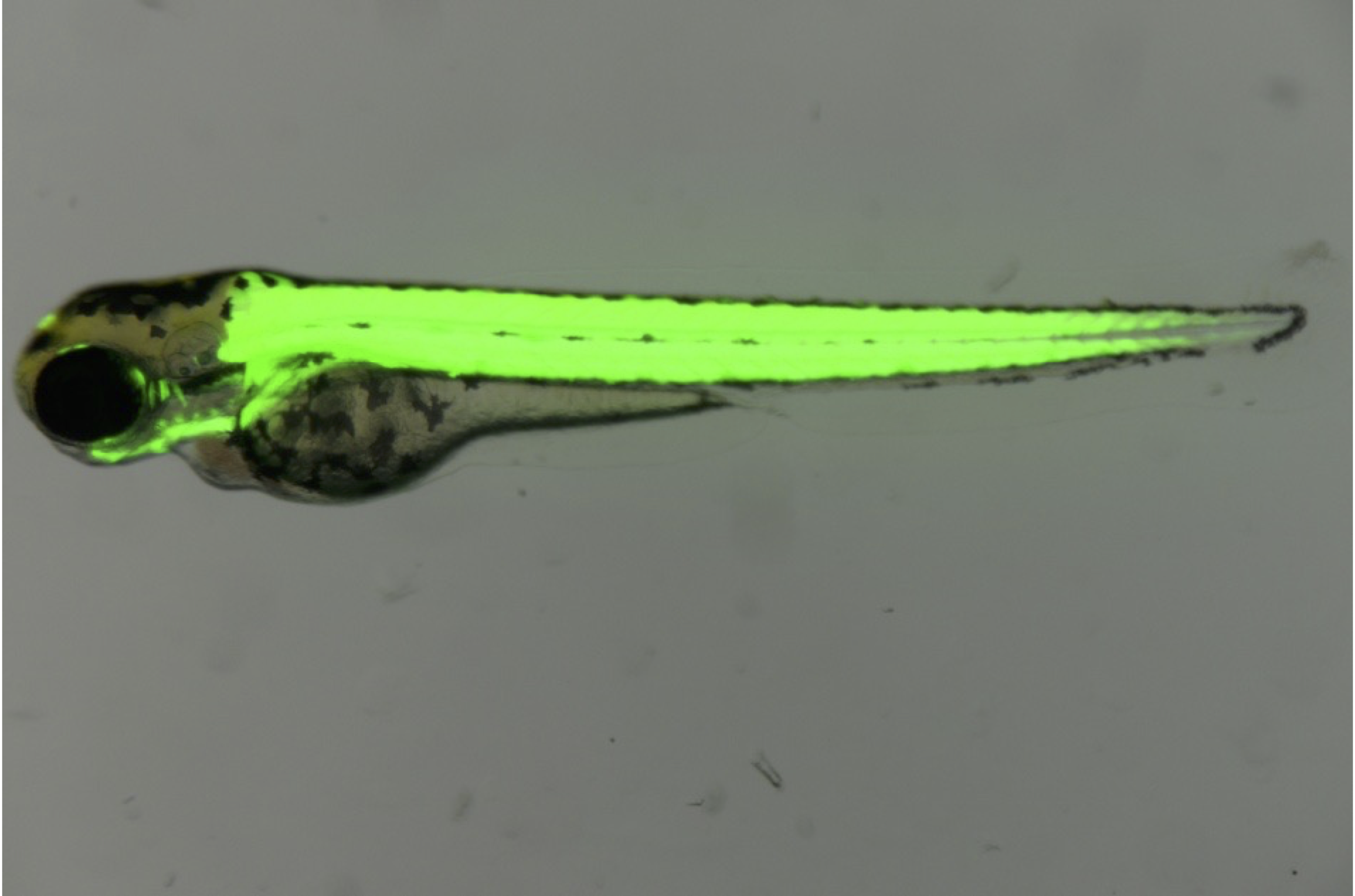

Supplement: Supplementary file 9 — Source data Fig. 2 [file 44321_2025_247_MOESM9_ESM.zip › Figure 2/Figure 2_Panel E/Figure 2_Panel E_control-MO_merge.tif]

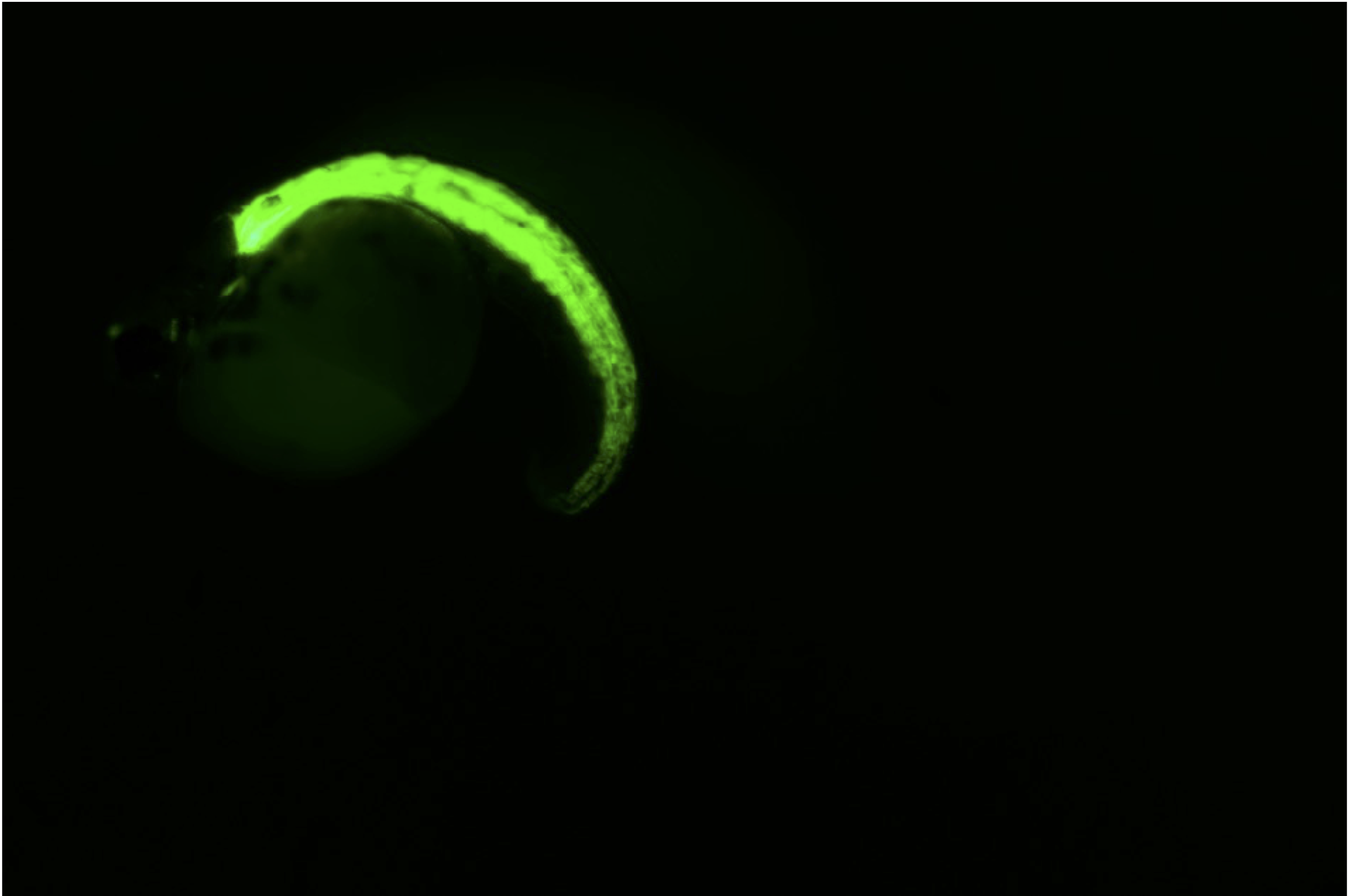

Supplement: Supplementary file 9 — Source data Fig. 2 [file 44321_2025_247_MOESM9_ESM.zip › Figure 2/Figure 2_Panel E/Figure 2_Panel E_ATG-MO_Tg(-1.9mylpfa-EGFP).tif]

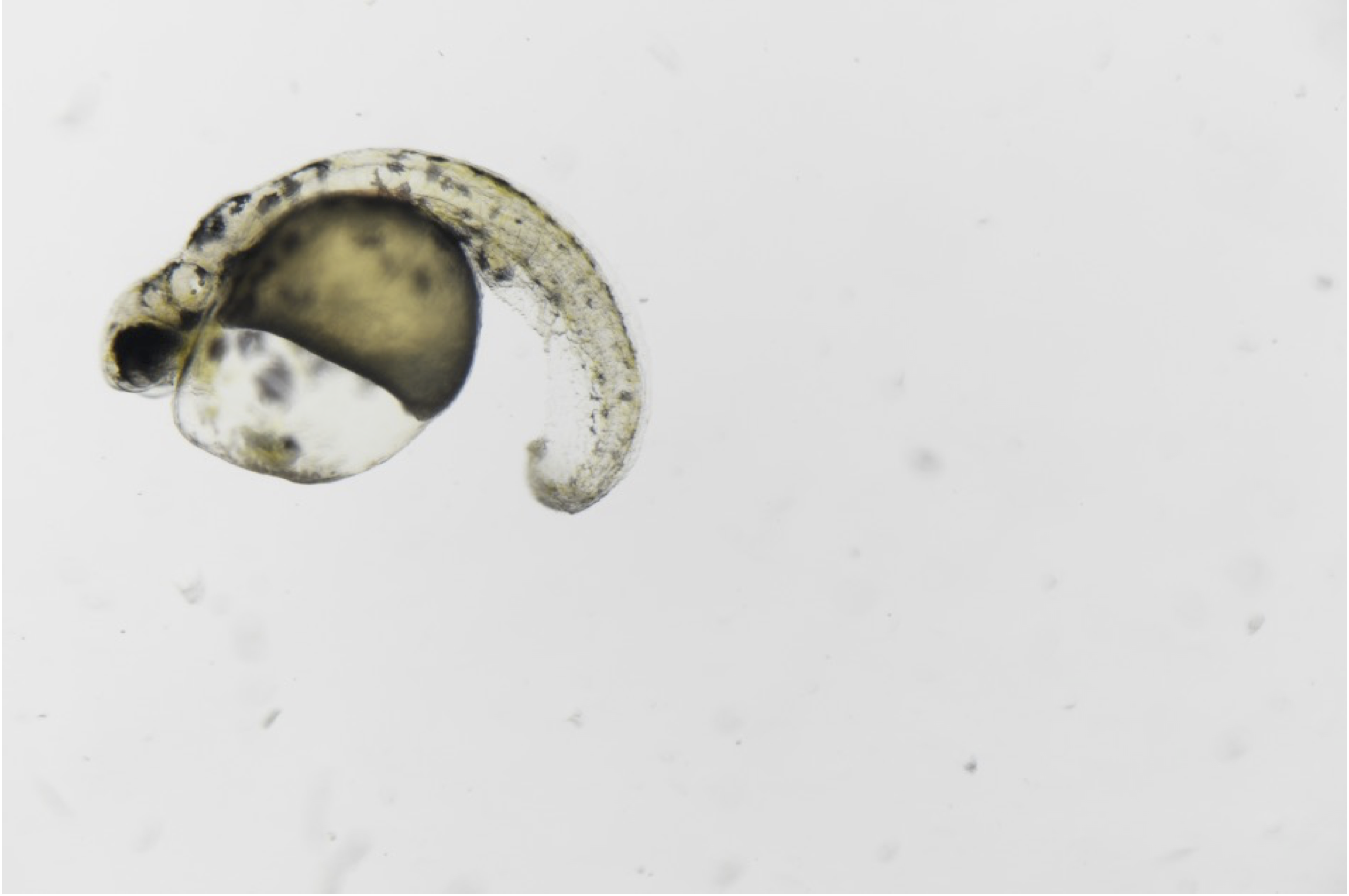

Supplement: Supplementary file 9 — Source data Fig. 2 [file 44321_2025_247_MOESM9_ESM.zip › Figure 2/Figure 2_Panel E/Figure 2_Panel E_ATG-MO_bright field.tif]

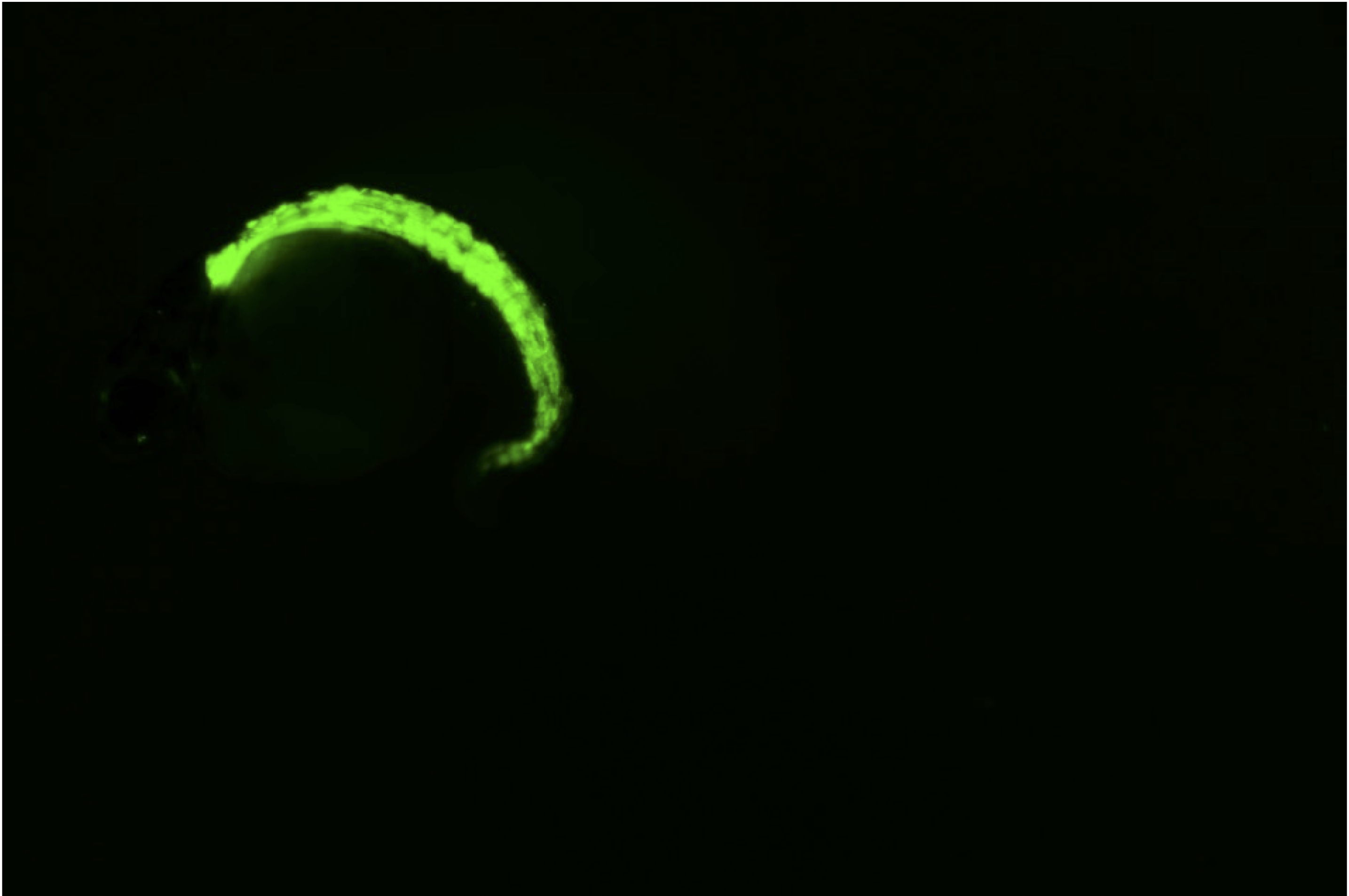

Supplement: Supplementary file 9 — Source data Fig. 2 [file 44321_2025_247_MOESM9_ESM.zip › Figure 2/Figure 2_Panel E/Figure 2_Panel E_I2E3-MO_Tg(-1.9mylpfa-EGFP).tif]

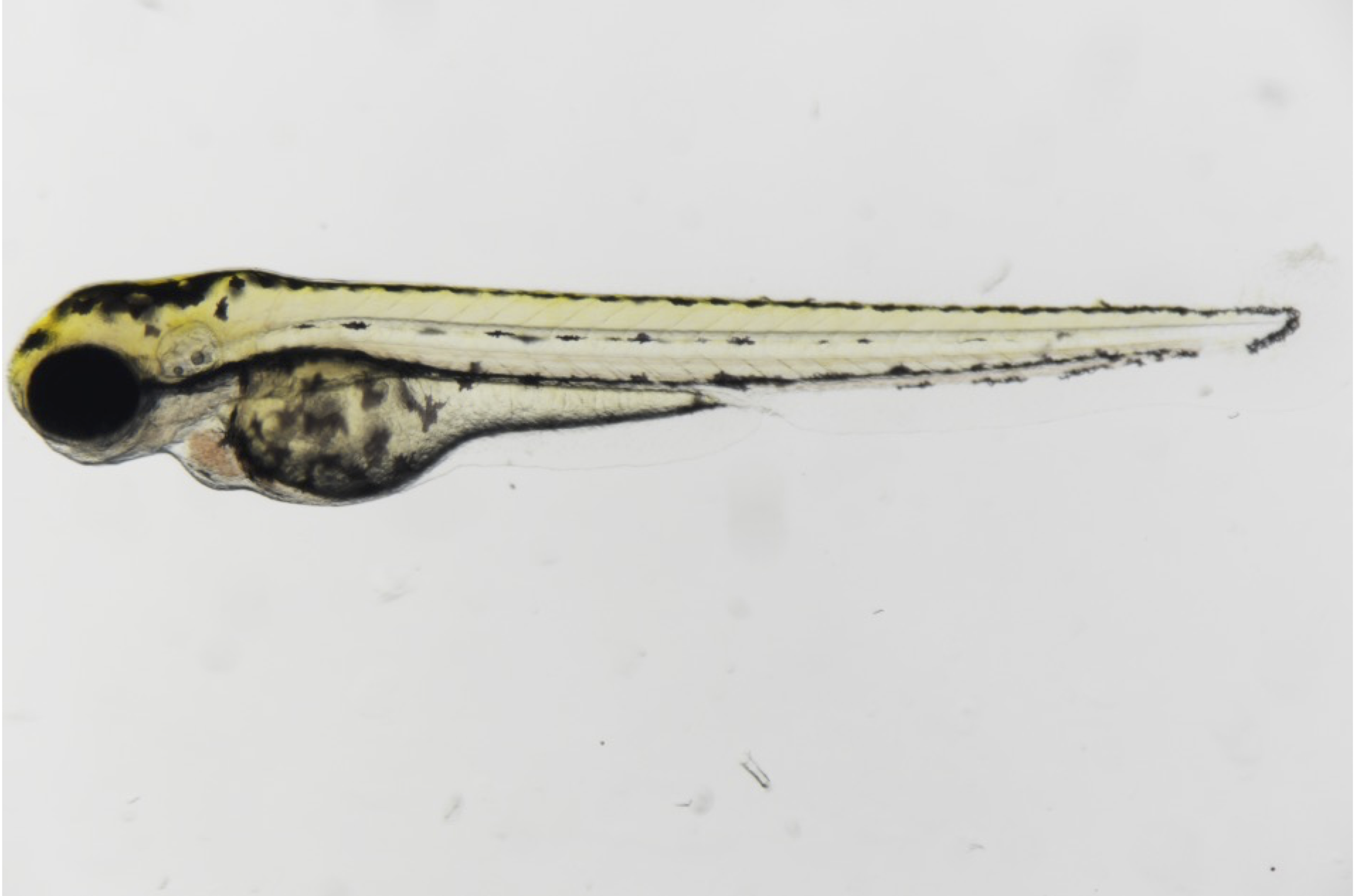

Supplement: Supplementary file 9 — Source data Fig. 2 [file 44321_2025_247_MOESM9_ESM.zip › Figure 2/Figure 2_Panel E/Figure 2_Panel E_control-MO_bright field.tif]

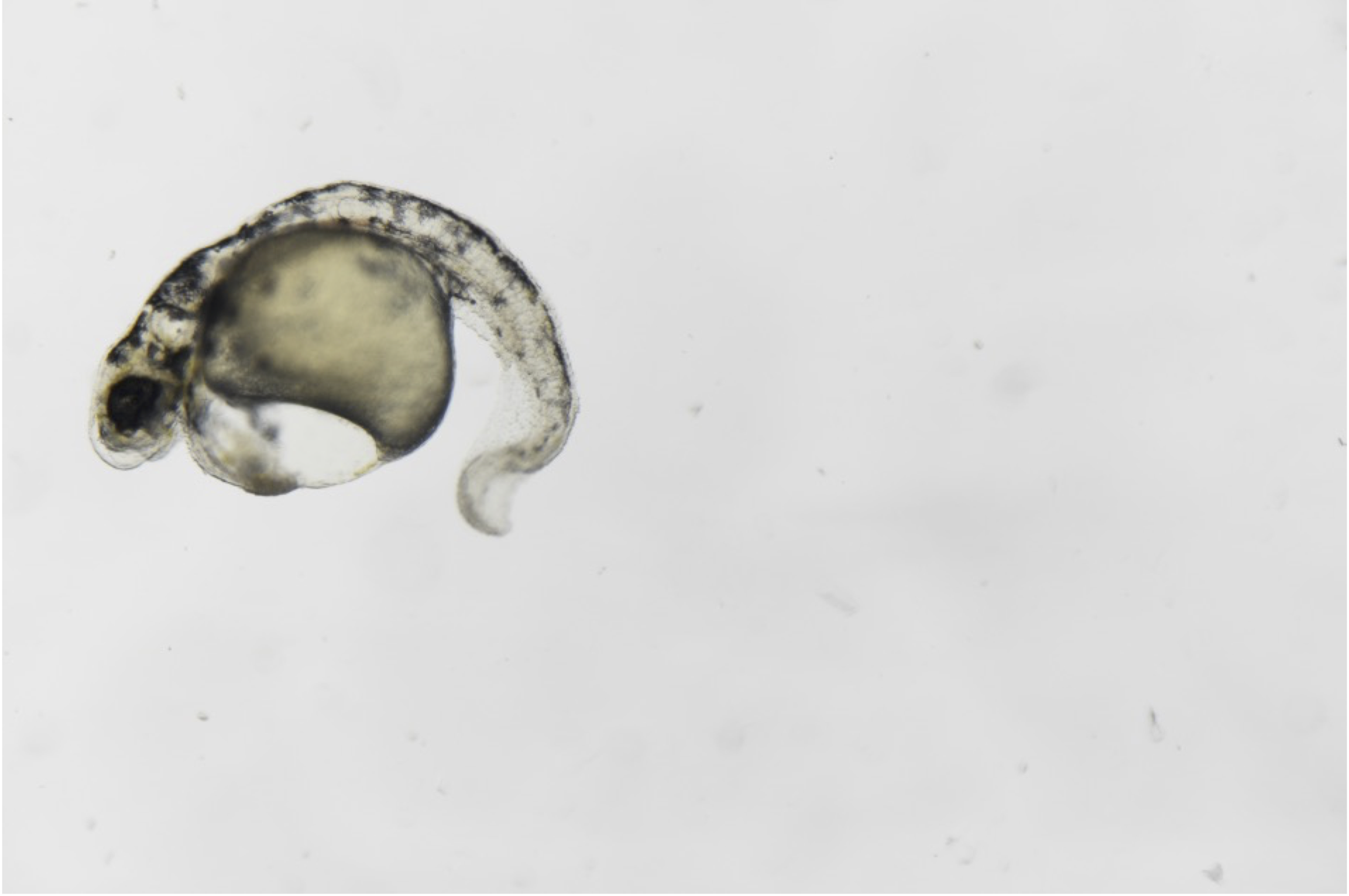

Supplement: Supplementary file 9 — Source data Fig. 2 [file 44321_2025_247_MOESM9_ESM.zip › Figure 2/Figure 2_Panel E/Figure 2_Panel E_I2E3-MO_bright field.tif]

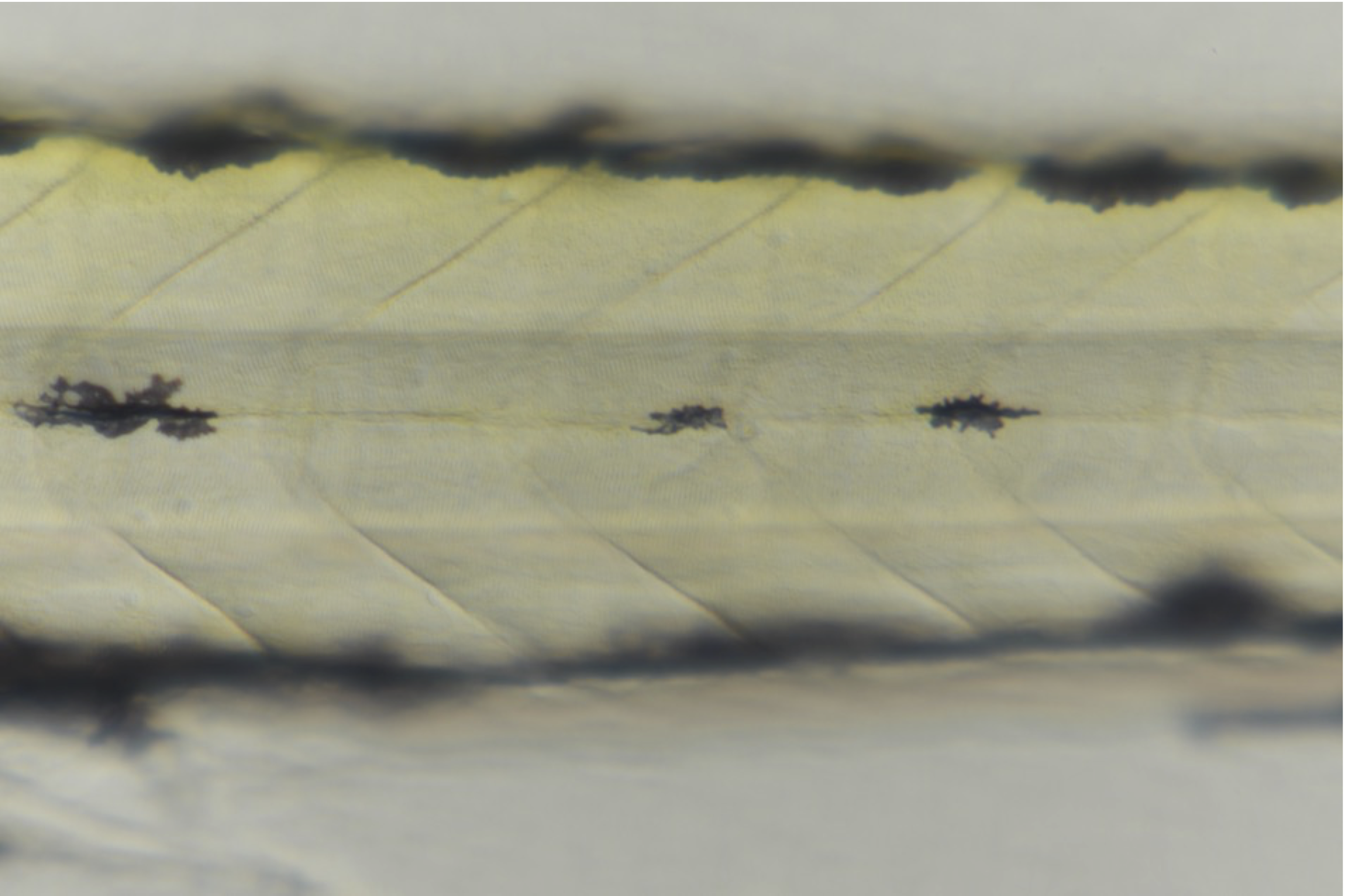

Supplement: Supplementary file 9 — Source data Fig. 2 [file 44321_2025_247_MOESM9_ESM.zip › Figure 2/Figure 2_Panel F/Figure 2_Panel F_control-MO_bright field.tif]

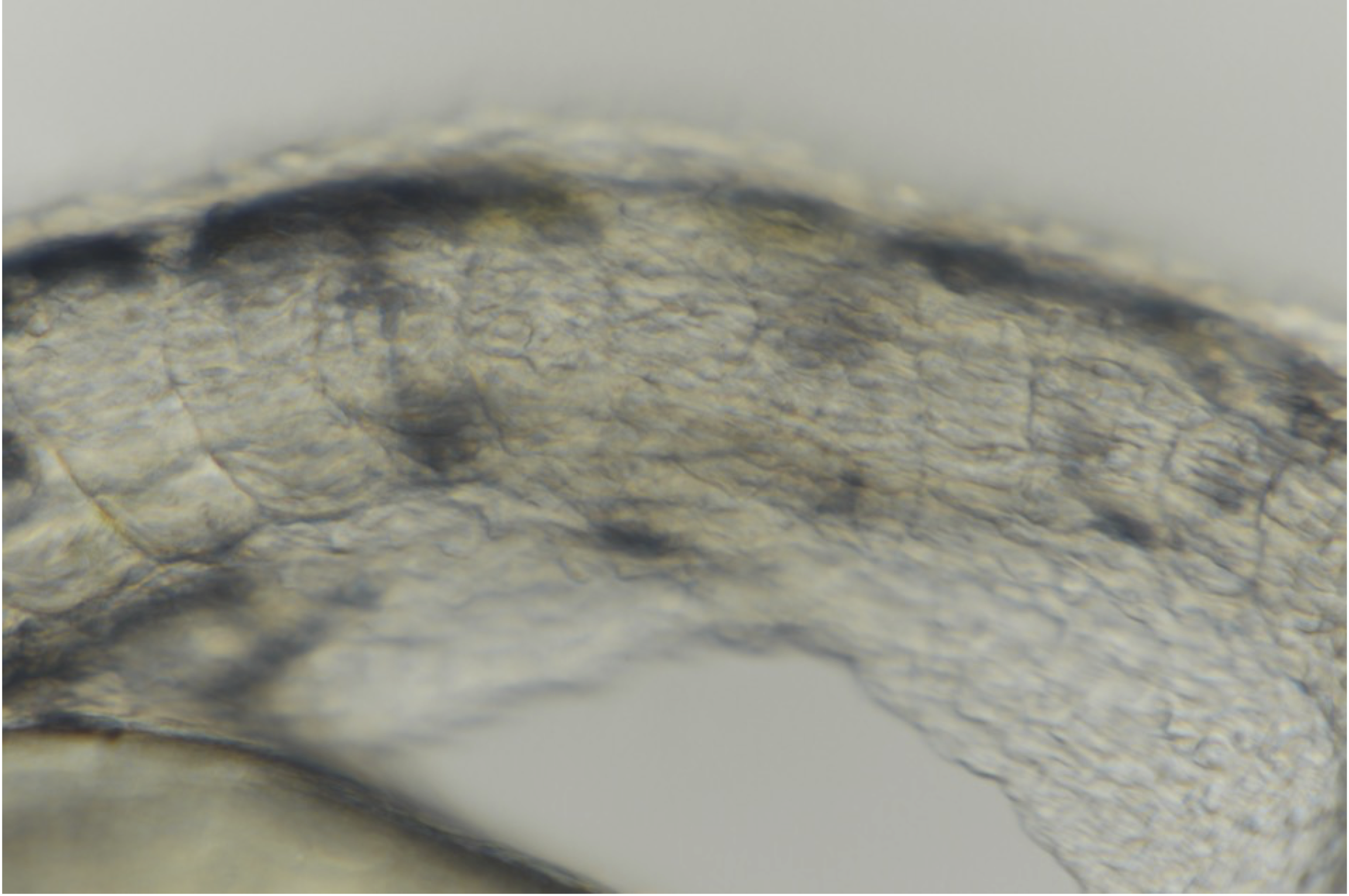

Supplement: Supplementary file 9 — Source data Fig. 2 [file 44321_2025_247_MOESM9_ESM.zip › Figure 2/Figure 2_Panel F/Figure 2_Panel F_I2E3-MO_bright field.tif]

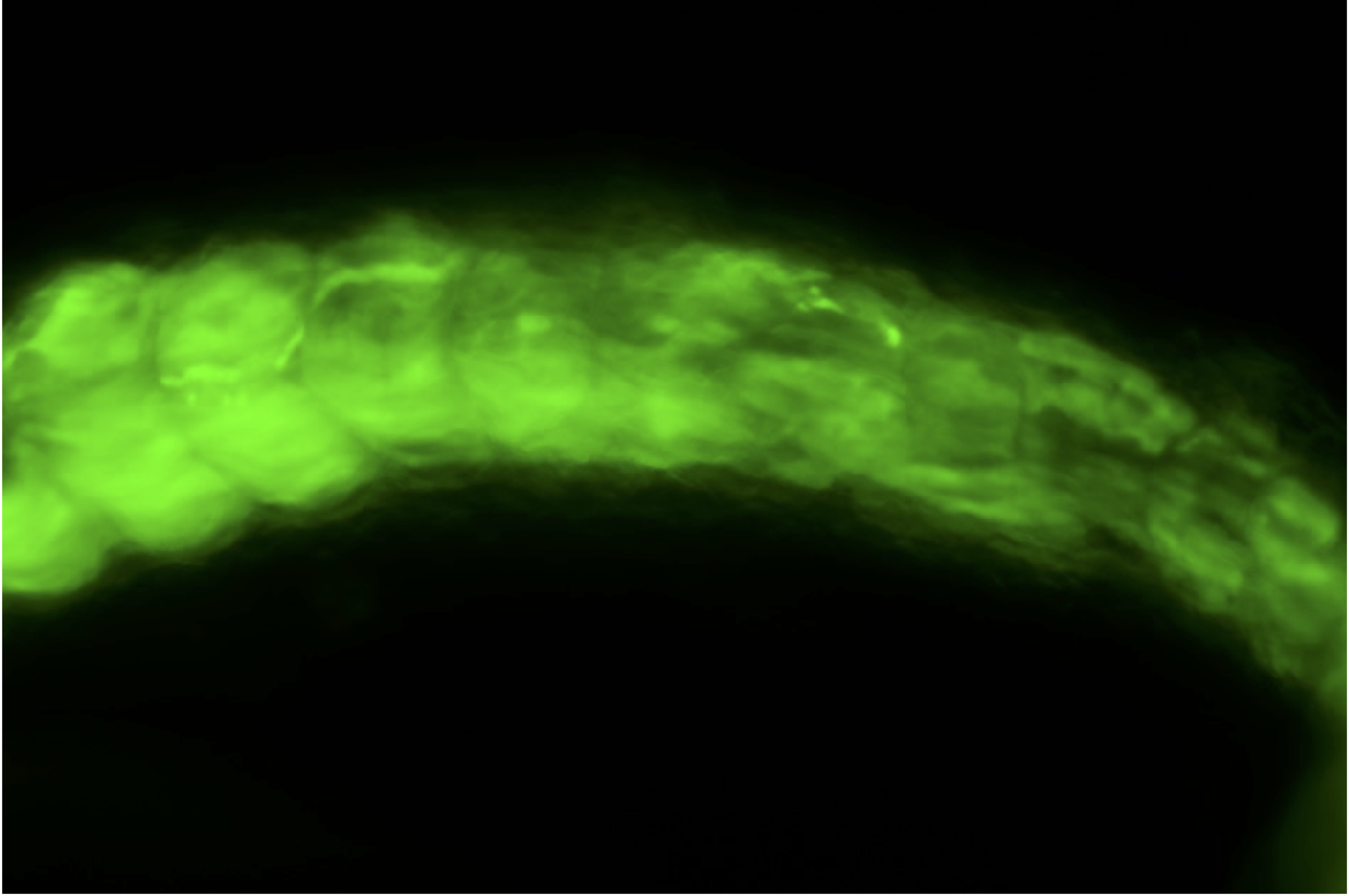

Supplement: Supplementary file 9 — Source data Fig. 2 [file 44321_2025_247_MOESM9_ESM.zip › Figure 2/Figure 2_Panel F/Figure 2_Panel F_I2E3-MO_Tg(-1.9mylpfa-EGFP).tif]

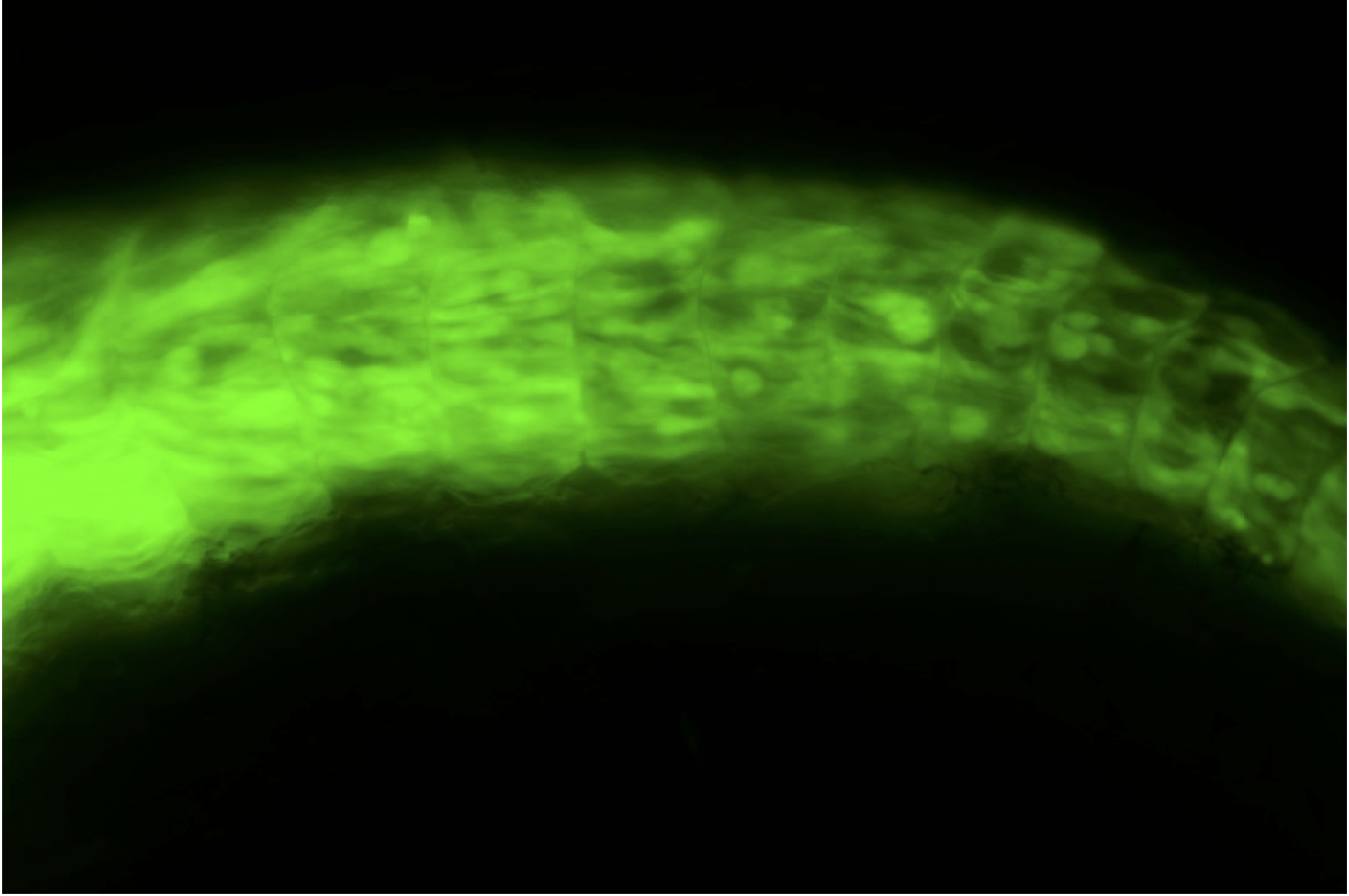

Supplement: Supplementary file 9 — Source data Fig. 2 [file 44321_2025_247_MOESM9_ESM.zip › Figure 2/Figure 2_Panel F/Figure 2_Panel F_ATG-MO_Tg(-1.9mylpfa-EGFP).tif]

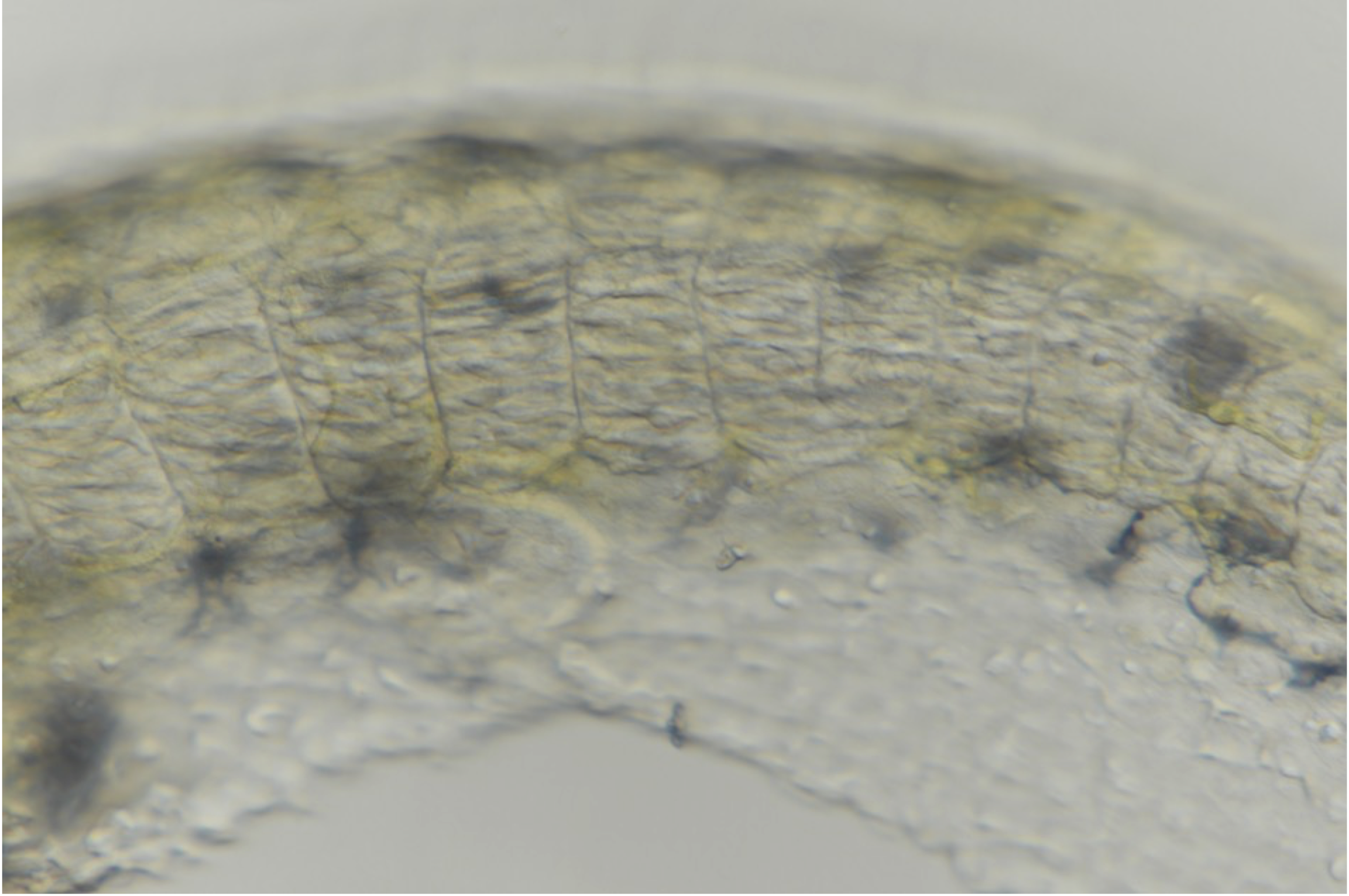

Supplement: Supplementary file 9 — Source data Fig. 2 [file 44321_2025_247_MOESM9_ESM.zip › Figure 2/Figure 2_Panel F/Figure 2_Panel F_ATG-MO_bright field.tif]

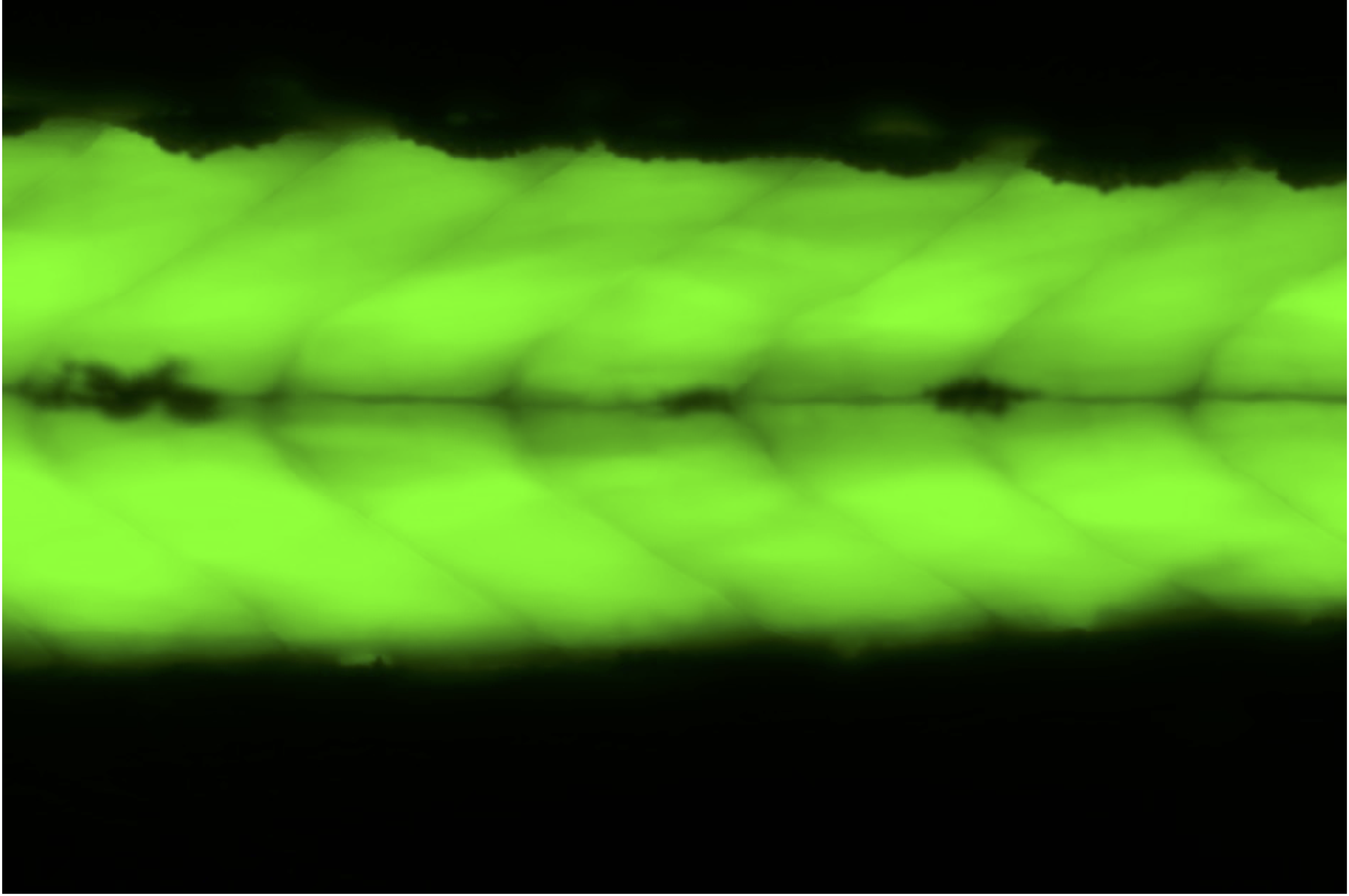

Supplement: Supplementary file 9 — Source data Fig. 2 [file 44321_2025_247_MOESM9_ESM.zip › Figure 2/Figure 2_Panel F/Figure 2_Panel F_control-MO_Tg(-1.9mylpfa-EGFP).tif]

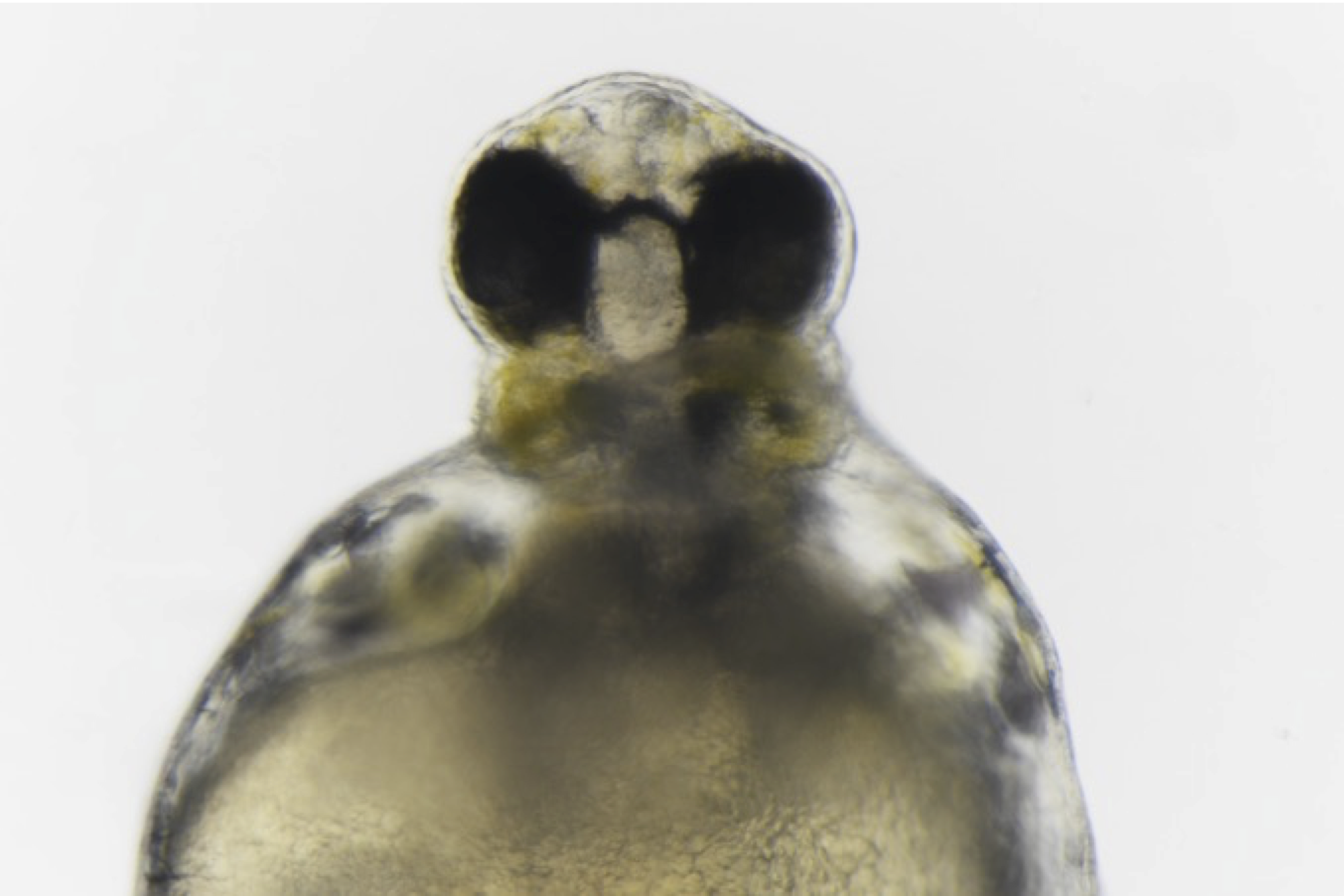

Supplement: Supplementary file 9 — Source data Fig. 2 [file 44321_2025_247_MOESM9_ESM.zip › Figure 2/Figure 2_Panel G/Figure 2_Panel G_ATG-MO_bright field.tif]

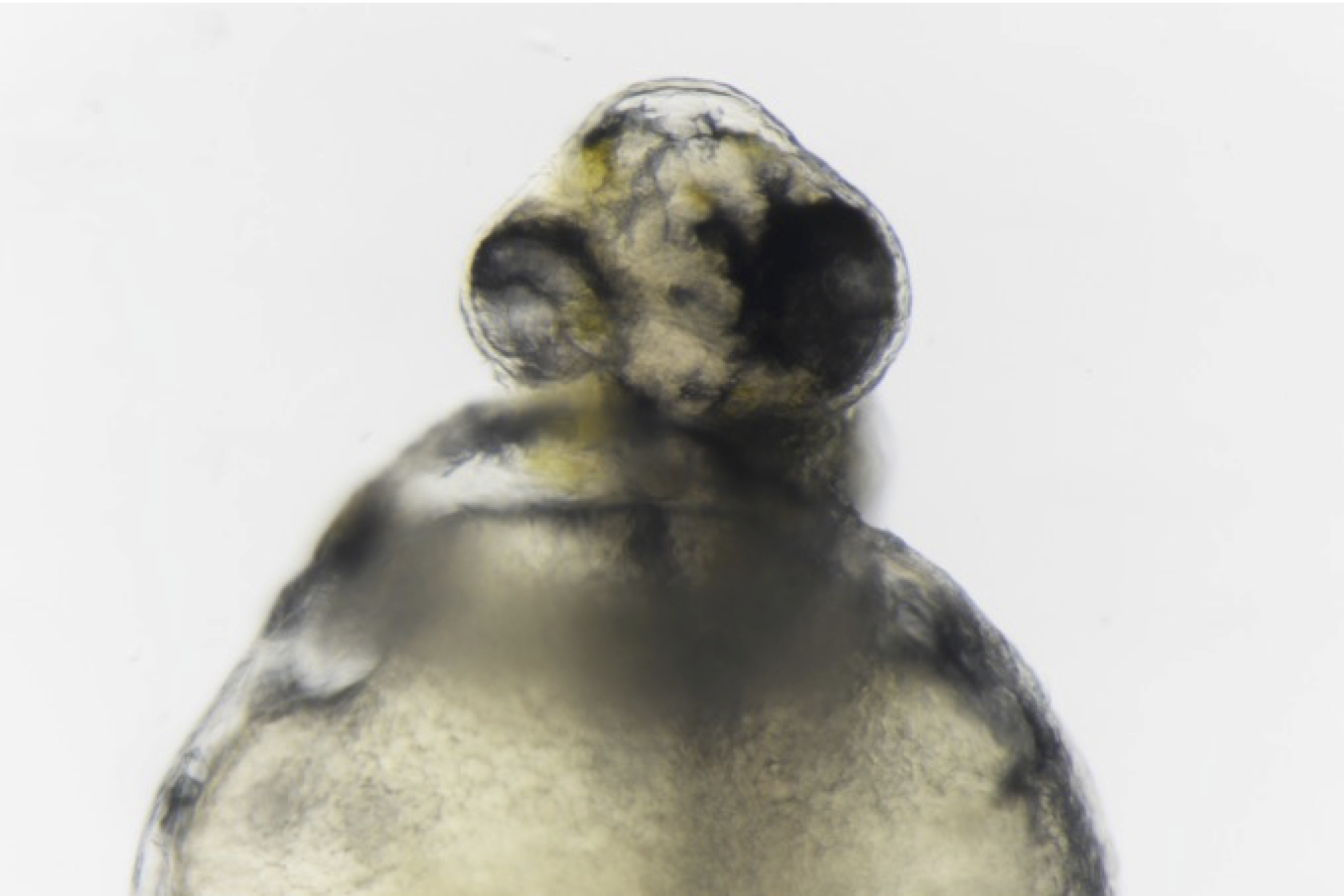

Supplement: Supplementary file 9 — Source data Fig. 2 [file 44321_2025_247_MOESM9_ESM.zip › Figure 2/Figure 2_Panel G/Figure 2_Panel G_I2E3-MO_bright field.tif]

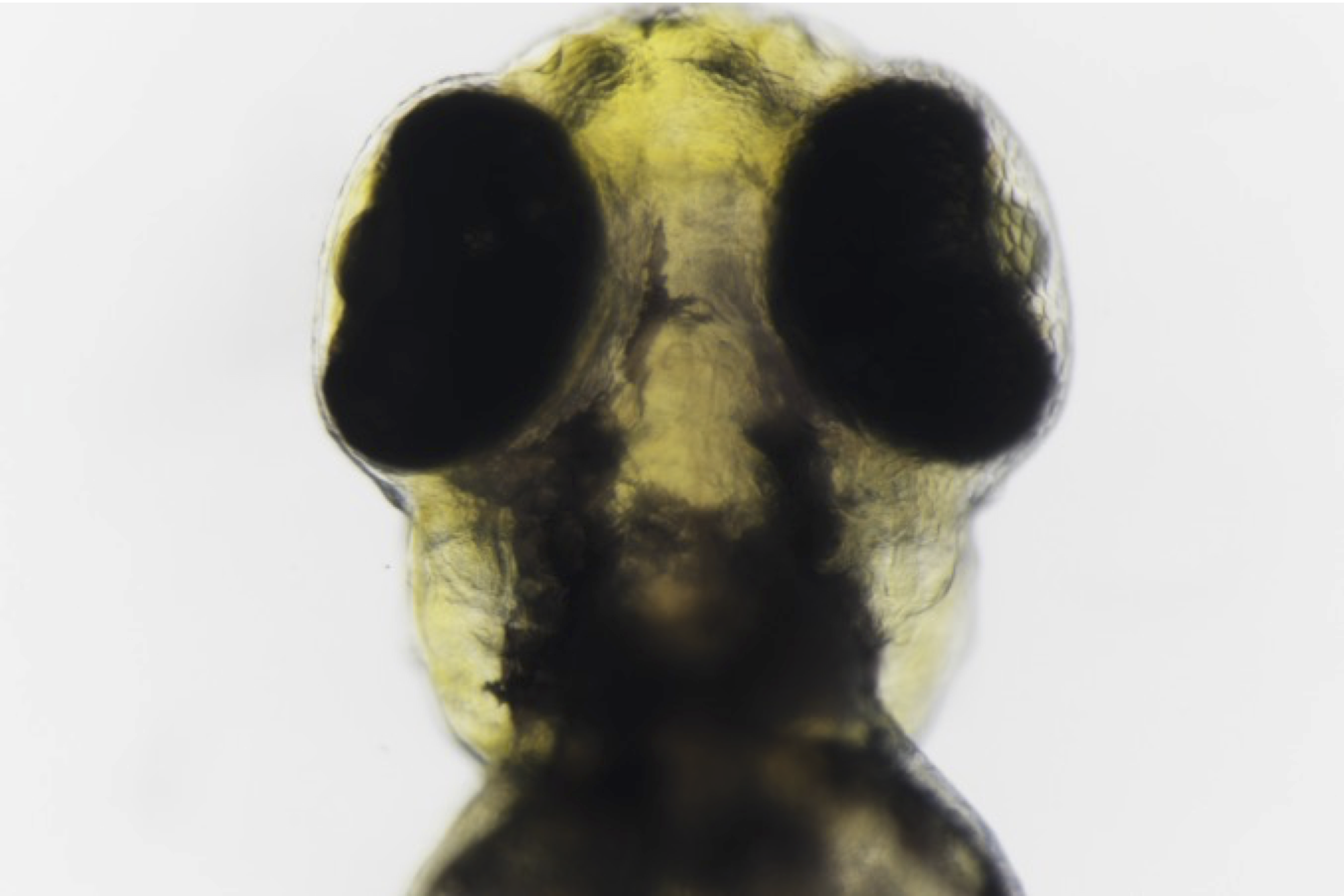

Supplement: Supplementary file 9 — Source data Fig. 2 [file 44321_2025_247_MOESM9_ESM.zip › Figure 2/Figure 2_Panel G/Figure 2_Panel G_control-MO_bright field.tif]

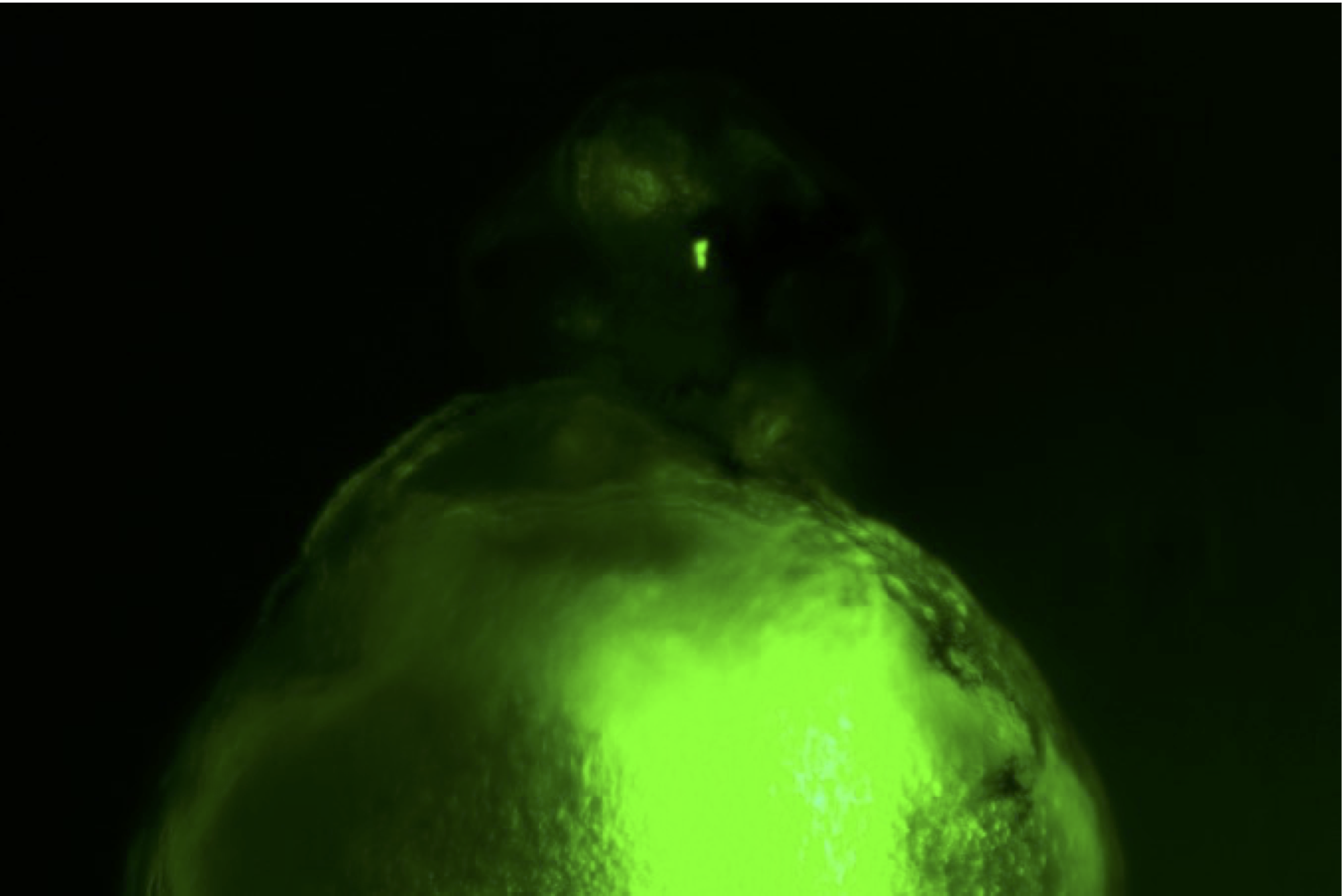

Supplement: Supplementary file 9 — Source data Fig. 2 [file 44321_2025_247_MOESM9_ESM.zip › Figure 2/Figure 2_Panel G/Figure 2_Panel G_I2E3-MO_Tg(-1.9mylpfa-EGFP).tif]

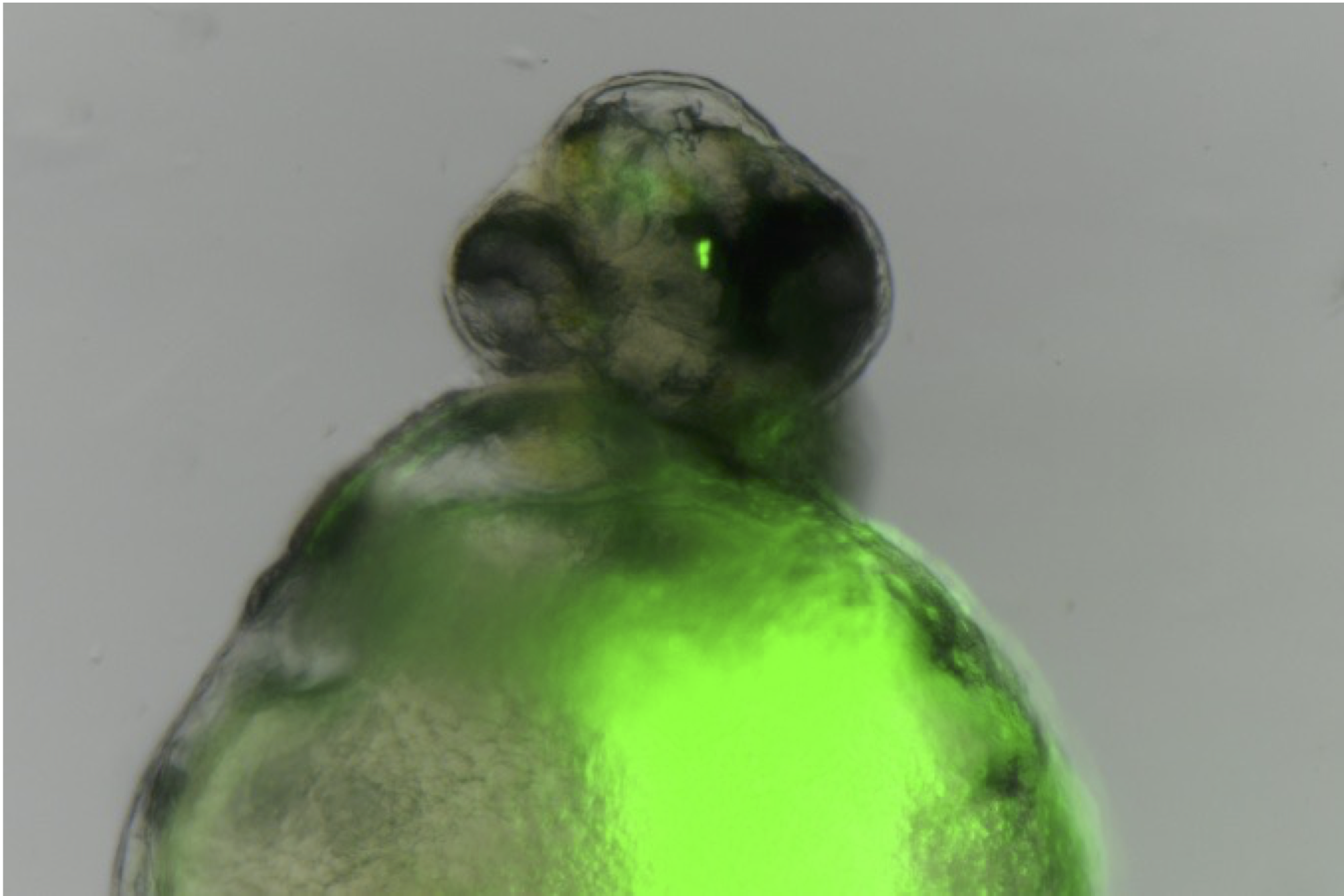

Supplement: Supplementary file 9 — Source data Fig. 2 [file 44321_2025_247_MOESM9_ESM.zip › Figure 2/Figure 2_Panel G/Figure 2_Panel G_I2E3-MO_merge.tif]

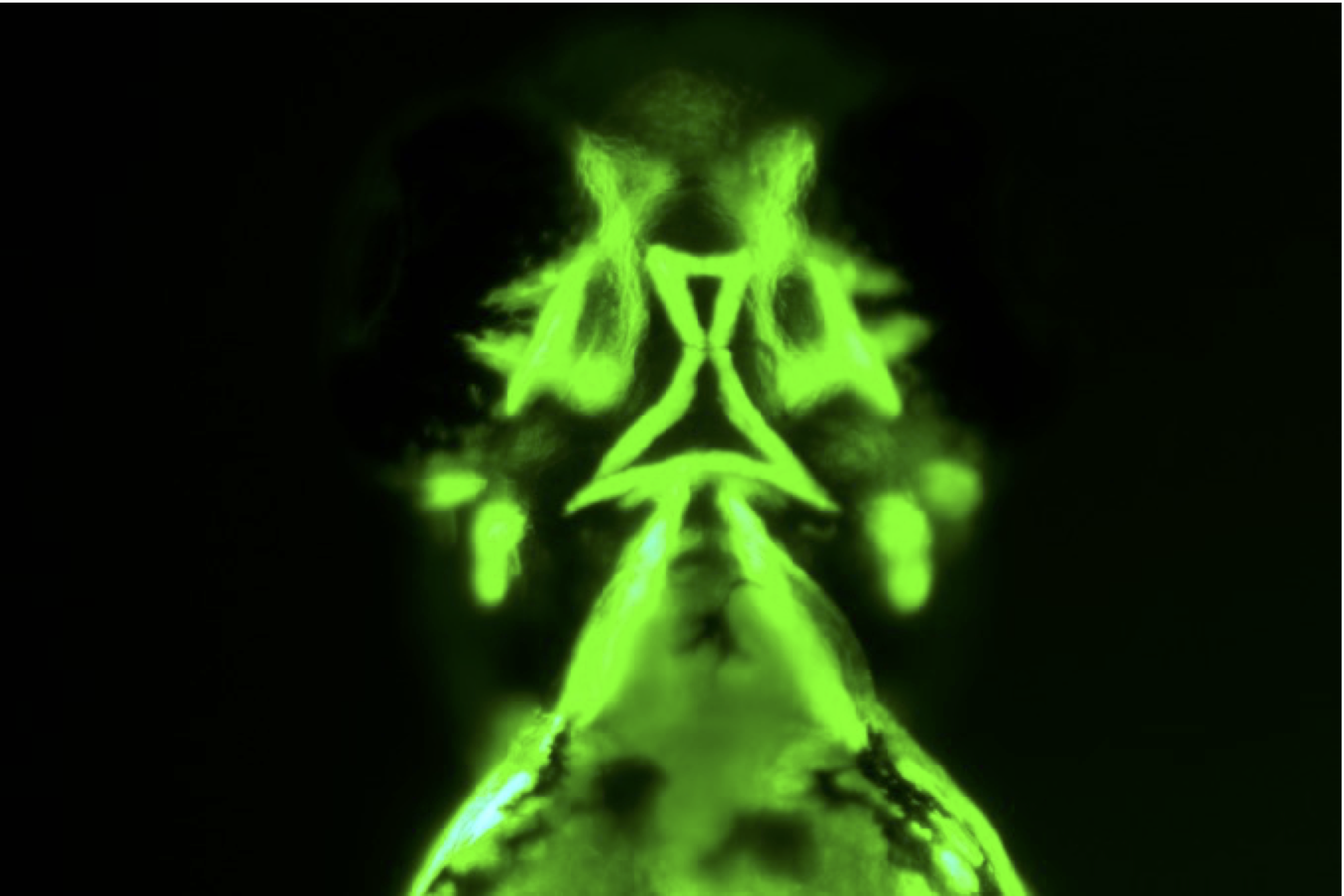

Supplement: Supplementary file 9 — Source data Fig. 2 [file 44321_2025_247_MOESM9_ESM.zip › Figure 2/Figure 2_Panel G/Figure 2_Panel G_control-MO_Tg(-1.9mylpfa-EGFP).tif]

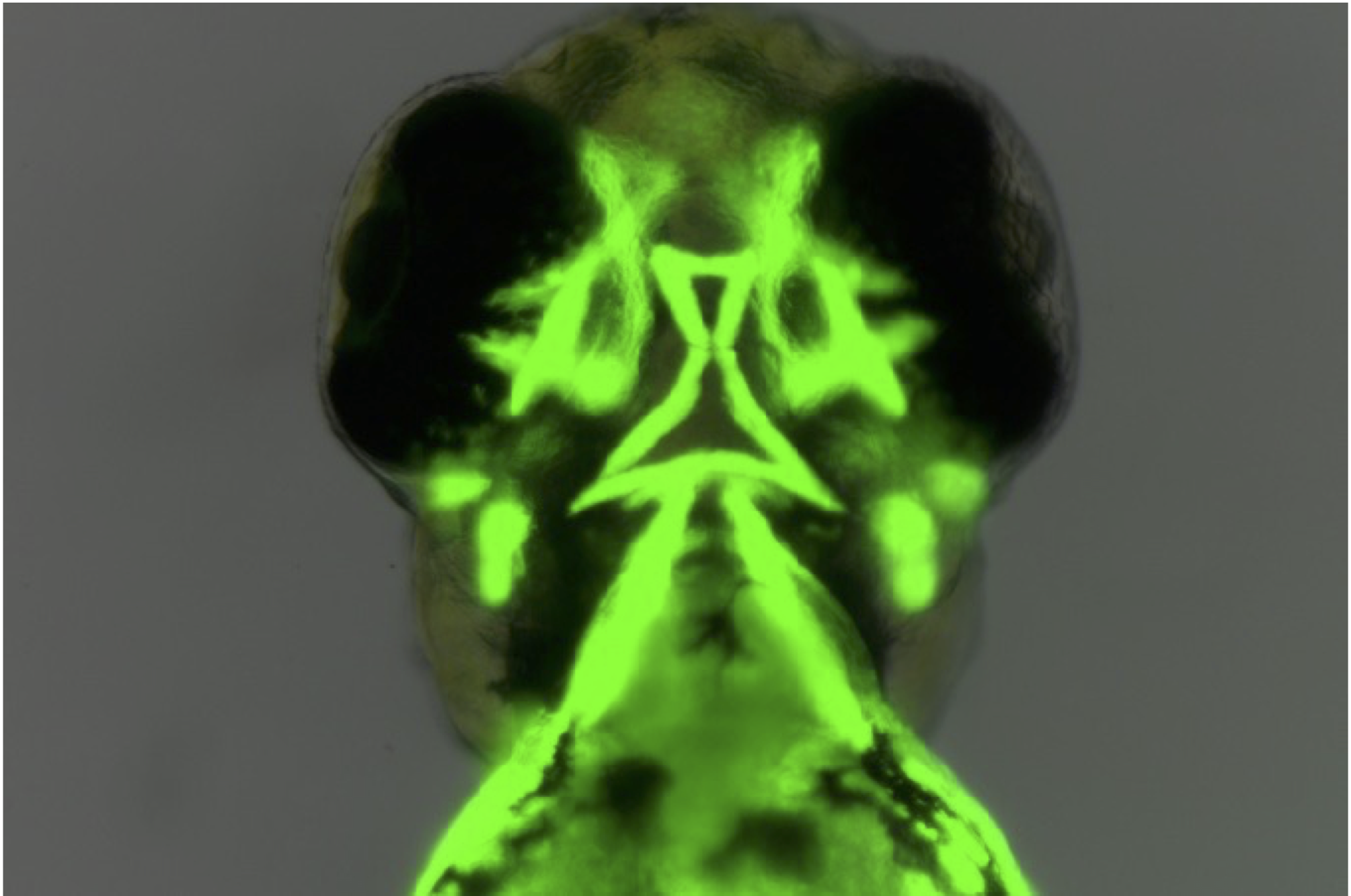

Supplement: Supplementary file 9 — Source data Fig. 2 [file 44321_2025_247_MOESM9_ESM.zip › Figure 2/Figure 2_Panel G/Figure 2_Panel G_control-MO_merge.tif]

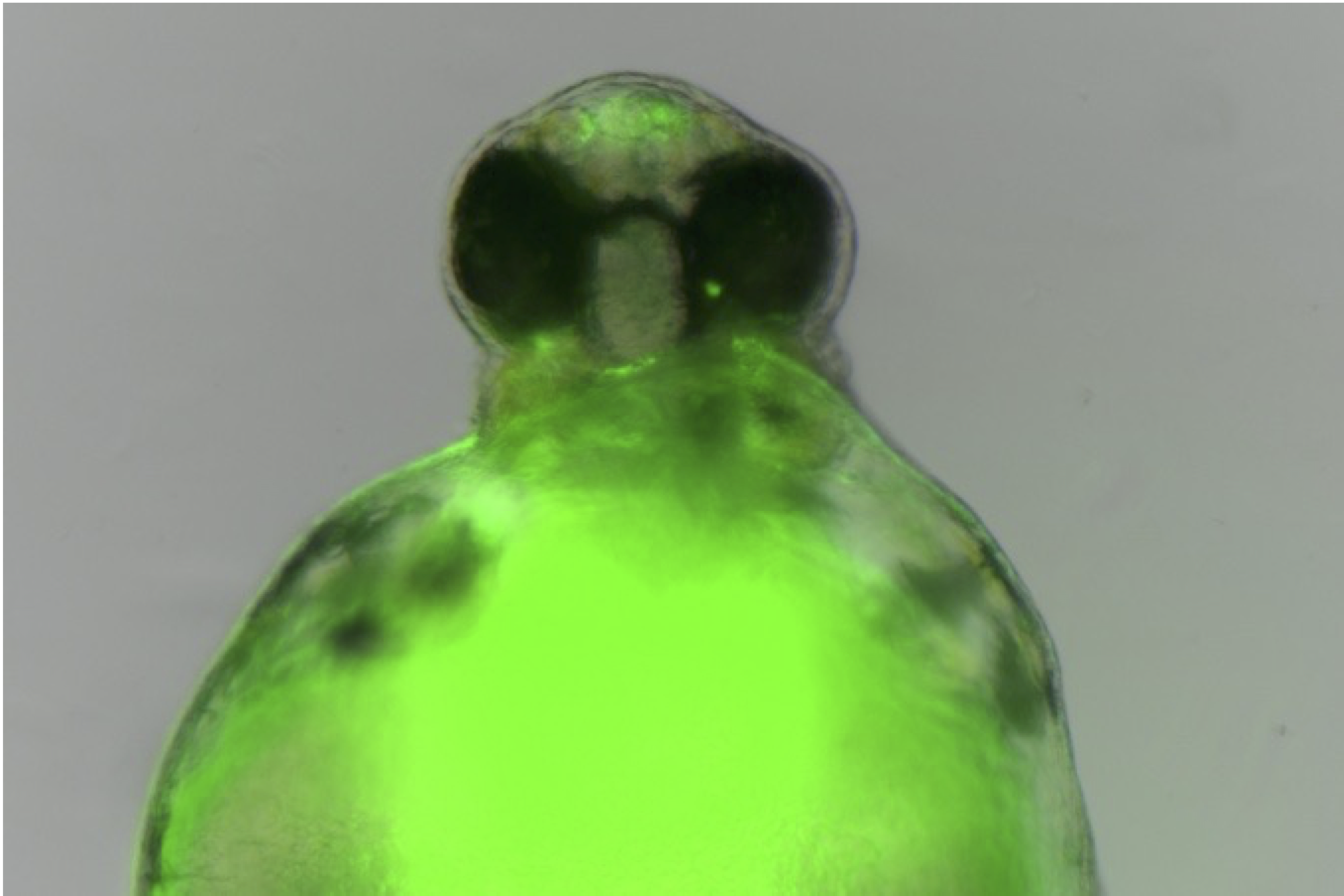

Supplement: Supplementary file 9 — Source data Fig. 2 [file 44321_2025_247_MOESM9_ESM.zip › Figure 2/Figure 2_Panel G/Figure 2_Panel G_ATG-MO_merge.tif]

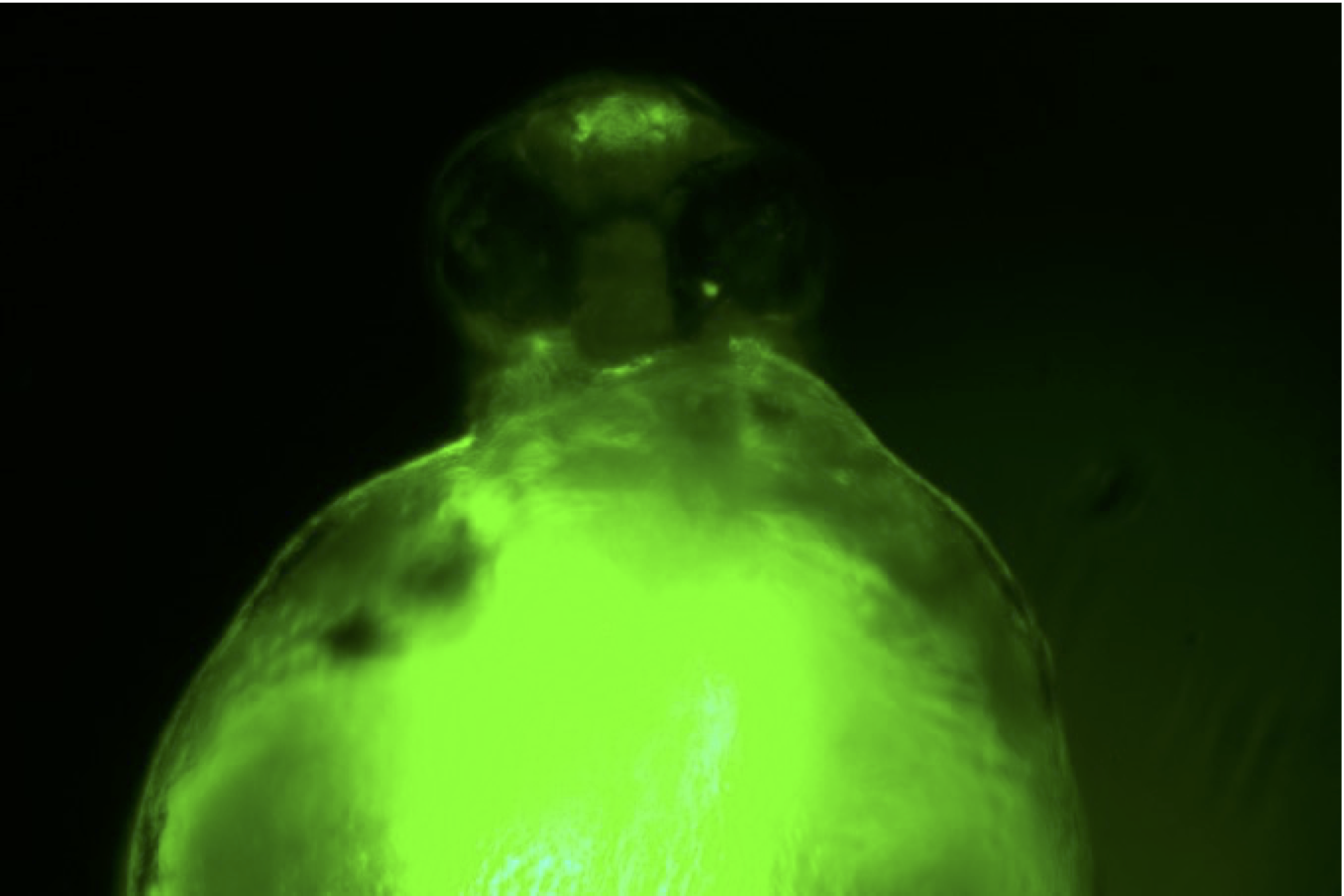

Supplement: Supplementary file 9 — Source data Fig. 2 [file 44321_2025_247_MOESM9_ESM.zip › Figure 2/Figure 2_Panel G/Figure 2_Panel G_ATG-MO_Tg(-1.9mylpfa-EGFP).tif]

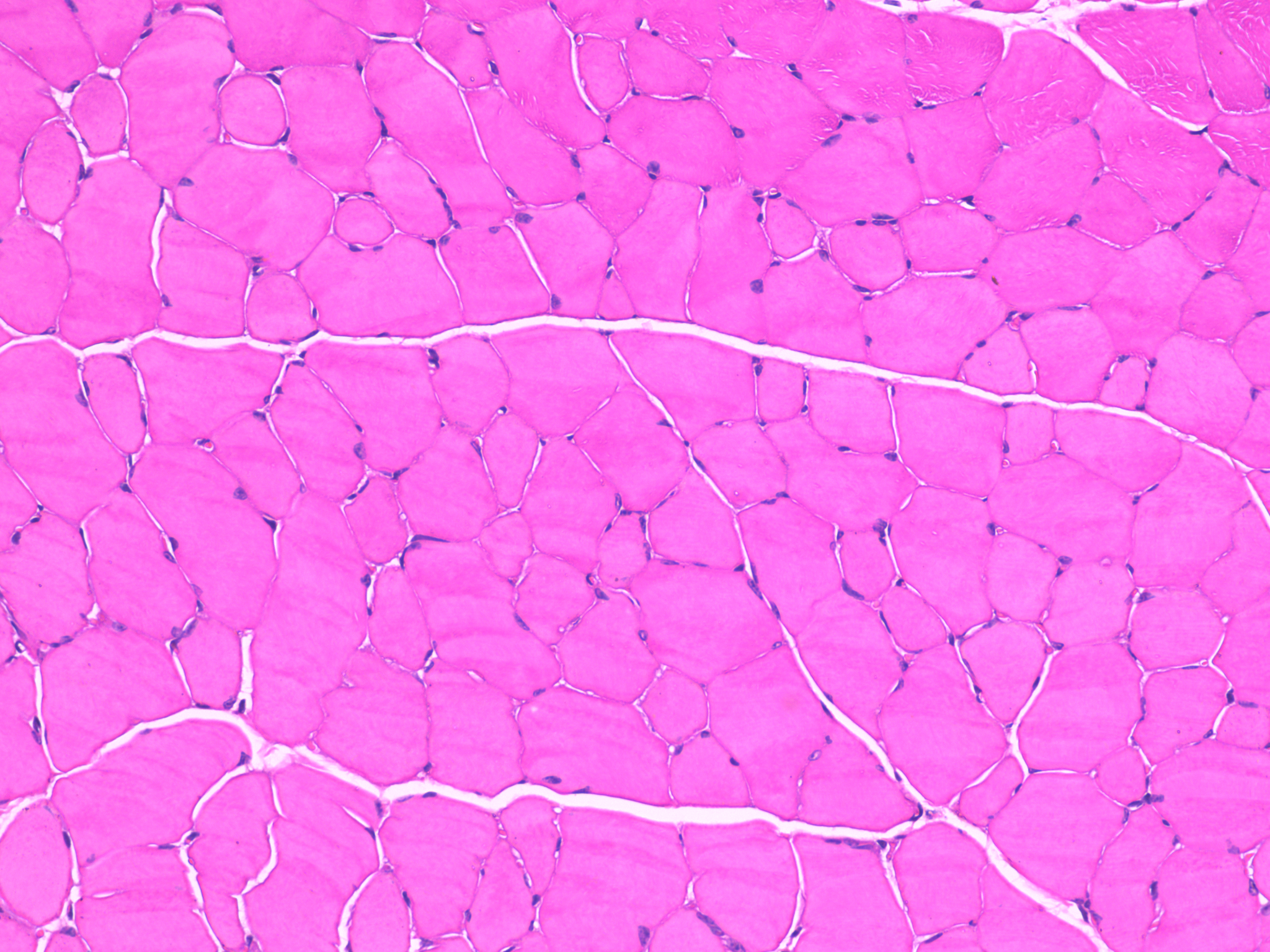

Supplement: Supplementary file 10 — Source data Fig. 3 [file 44321_2025_247_MOESM10_ESM.zip › Figure 3/Figure 3_Panel E/Figure 3_Panel E_HE_8w-Foxk2fl:fl-TA.tif]

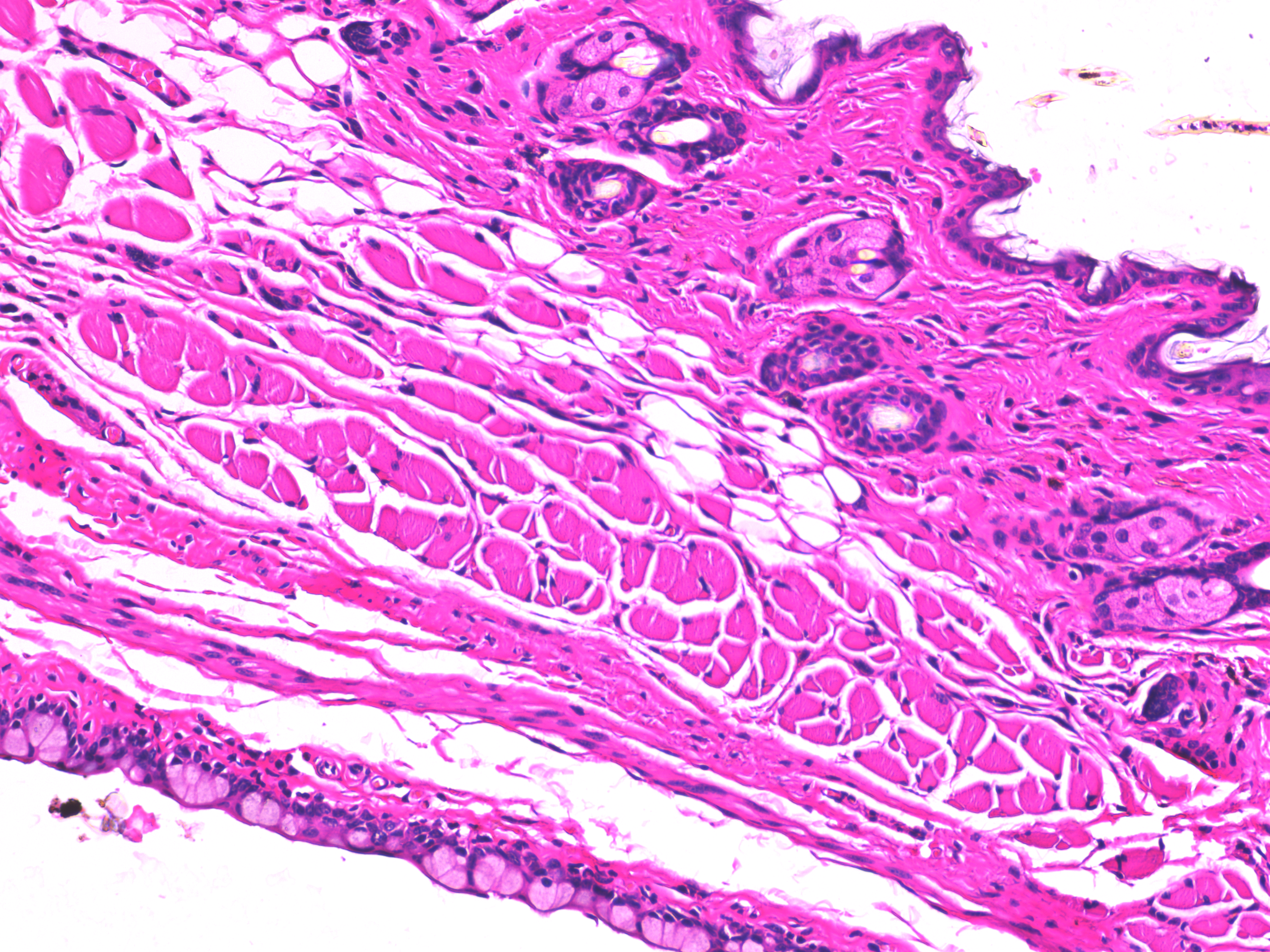

Supplement: Supplementary file 10 — Source data Fig. 3 [file 44321_2025_247_MOESM10_ESM.zip › Figure 3/Figure 3_Panel E/Figure 3_Panel E_HE_8w-Foxk2fl:fl-Myod1-Cre-EM.tif]

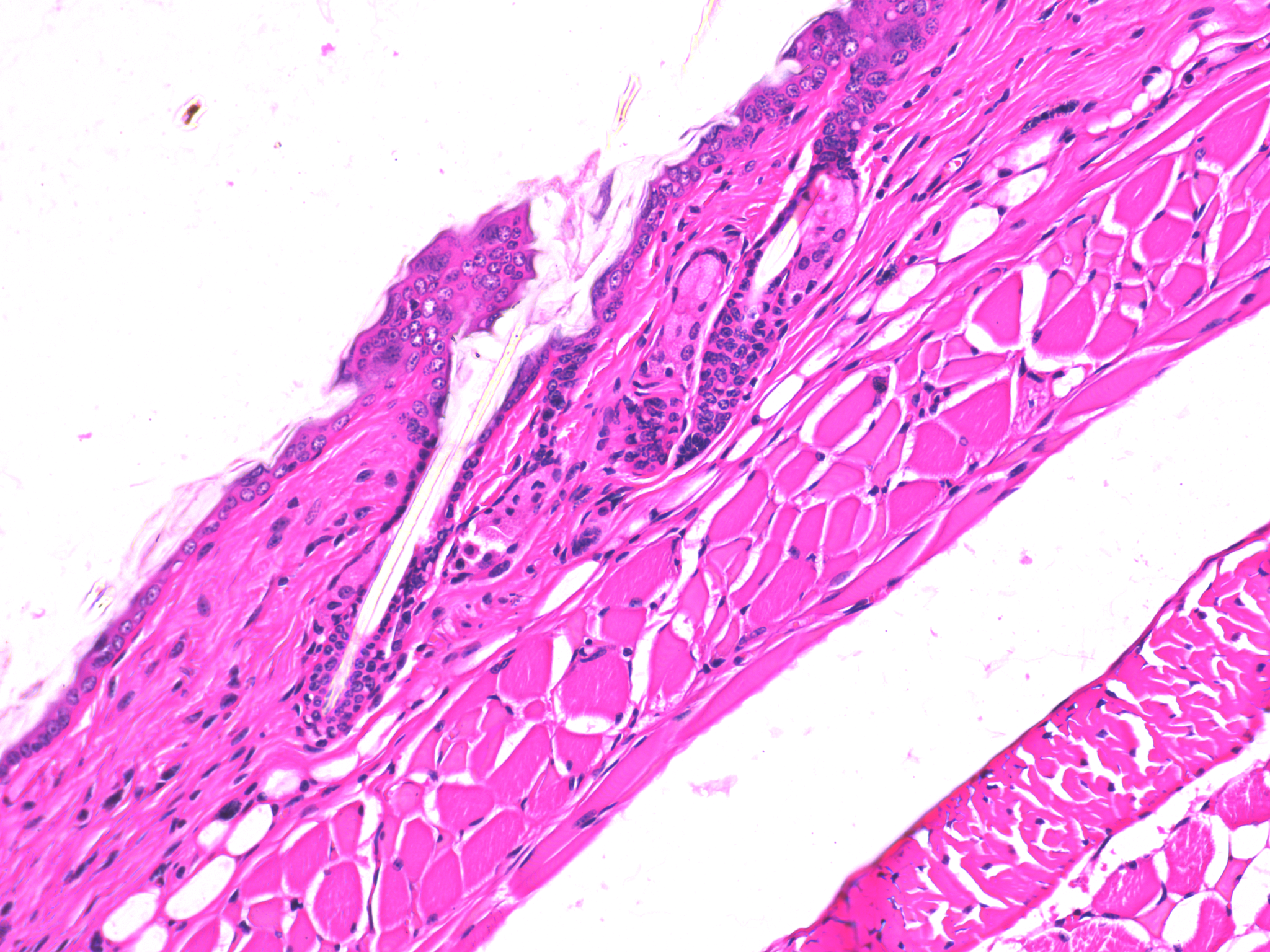

Supplement: Supplementary file 10 — Source data Fig. 3 [file 44321_2025_247_MOESM10_ESM.zip › Figure 3/Figure 3_Panel E/Figure 3_Panel E_HE_8w-Foxk2fl:fl-EM.tif]

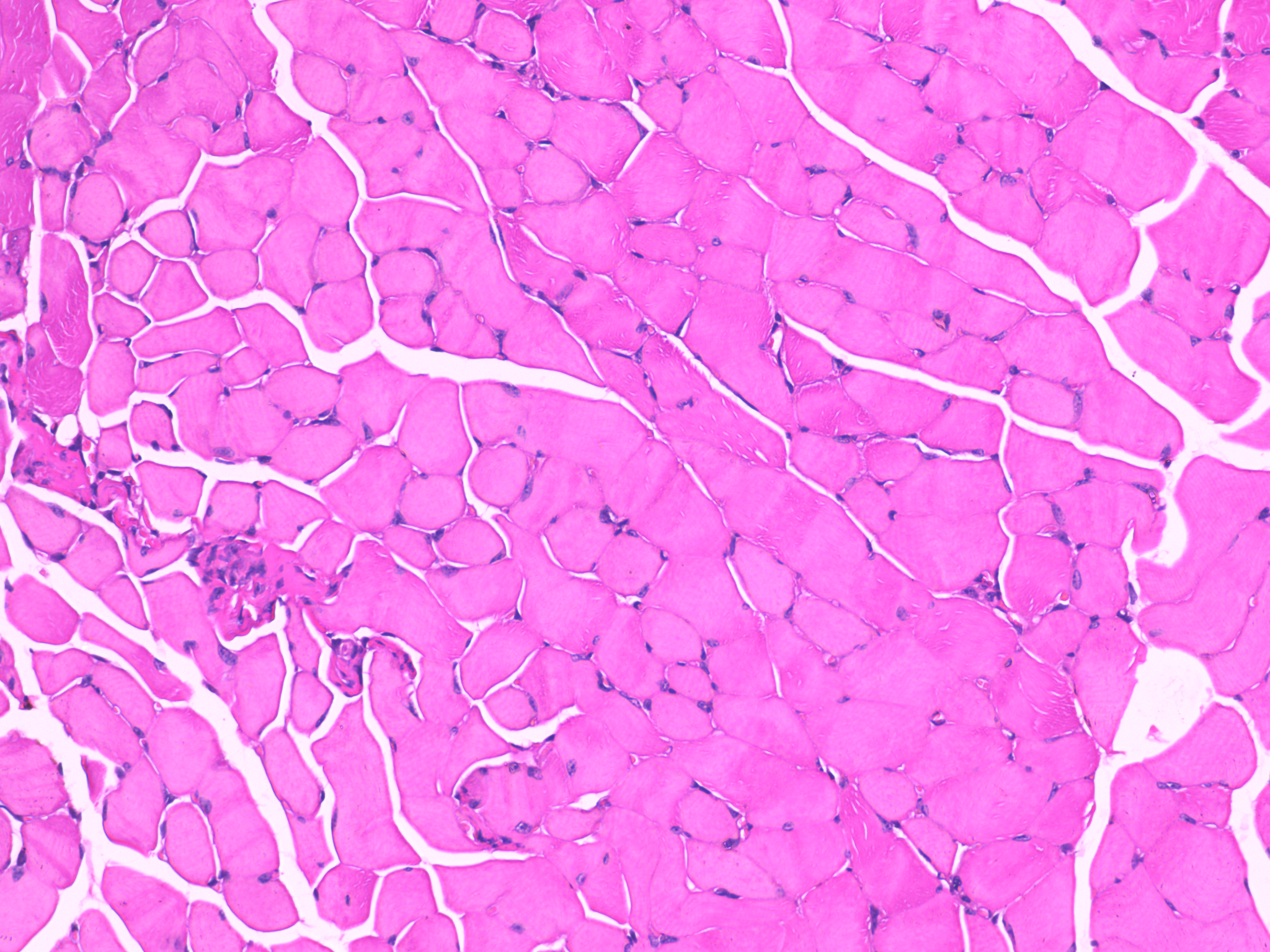

Supplement: Supplementary file 10 — Source data Fig. 3 [file 44321_2025_247_MOESM10_ESM.zip › Figure 3/Figure 3_Panel E/Figure 3_Panel E_HE_8w-Foxk2fl:fl-Myod1-Cre-TA.tif]

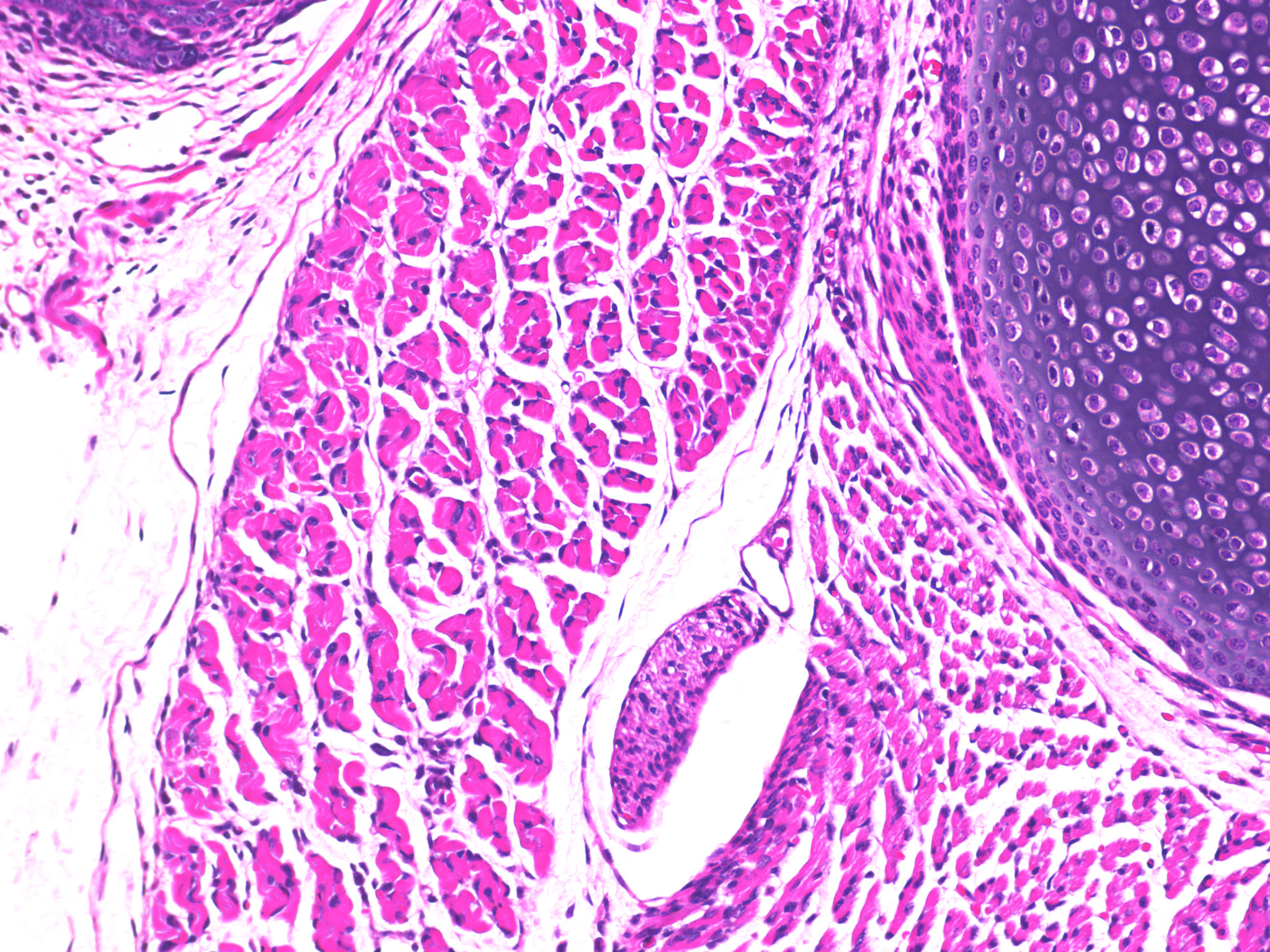

Supplement: Supplementary file 10 — Source data Fig. 3 [file 44321_2025_247_MOESM10_ESM.zip › Figure 3/Figure 3_Panel E/Figure 3_Panel E_HE_1d-Foxk2fl:fl-TA.tif]

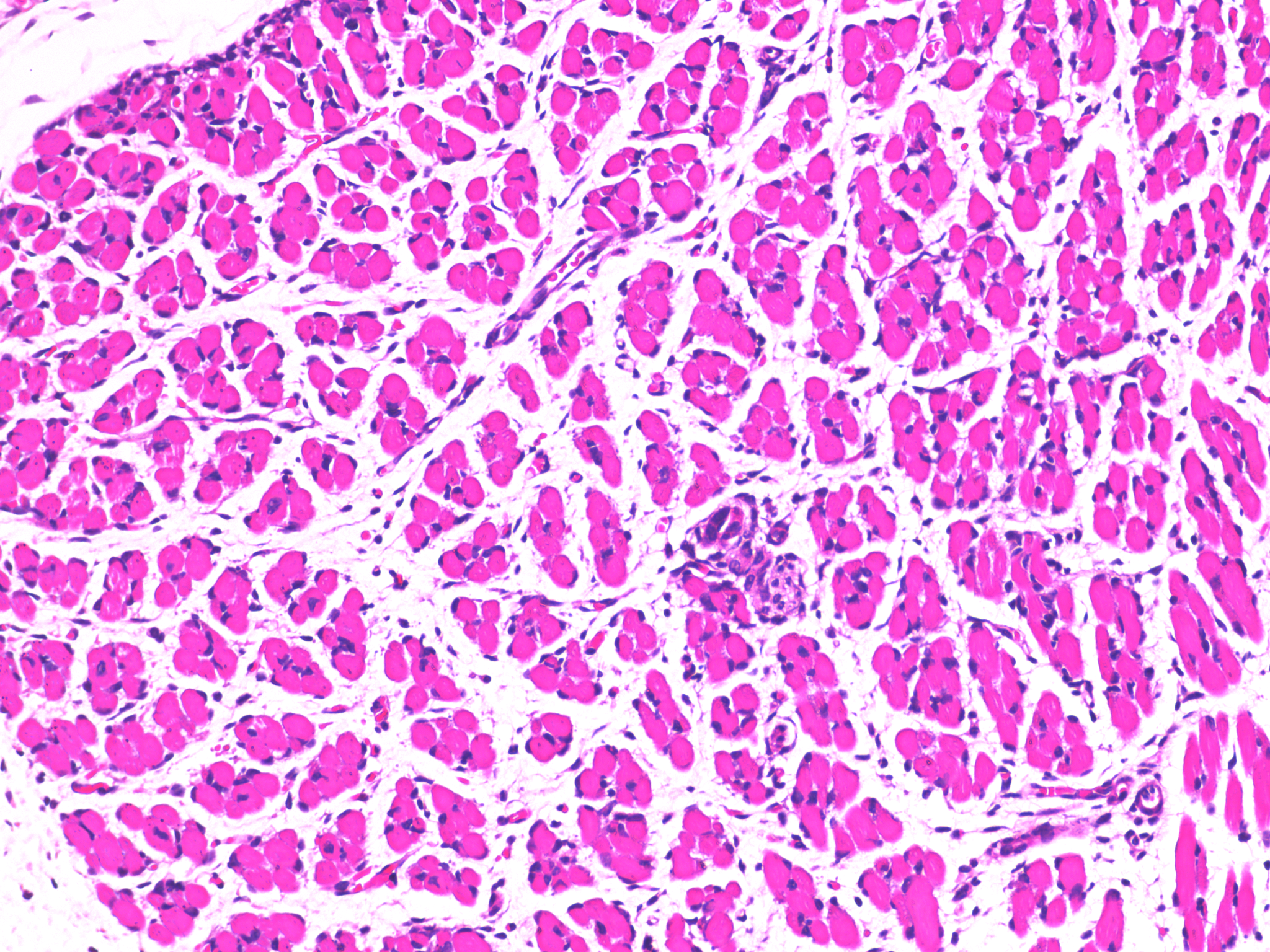

Supplement: Supplementary file 10 — Source data Fig. 3 [file 44321_2025_247_MOESM10_ESM.zip › Figure 3/Figure 3_Panel E/Figure 3_Panel E_HE_1d-Foxk2fl:fl-Gas.tif]

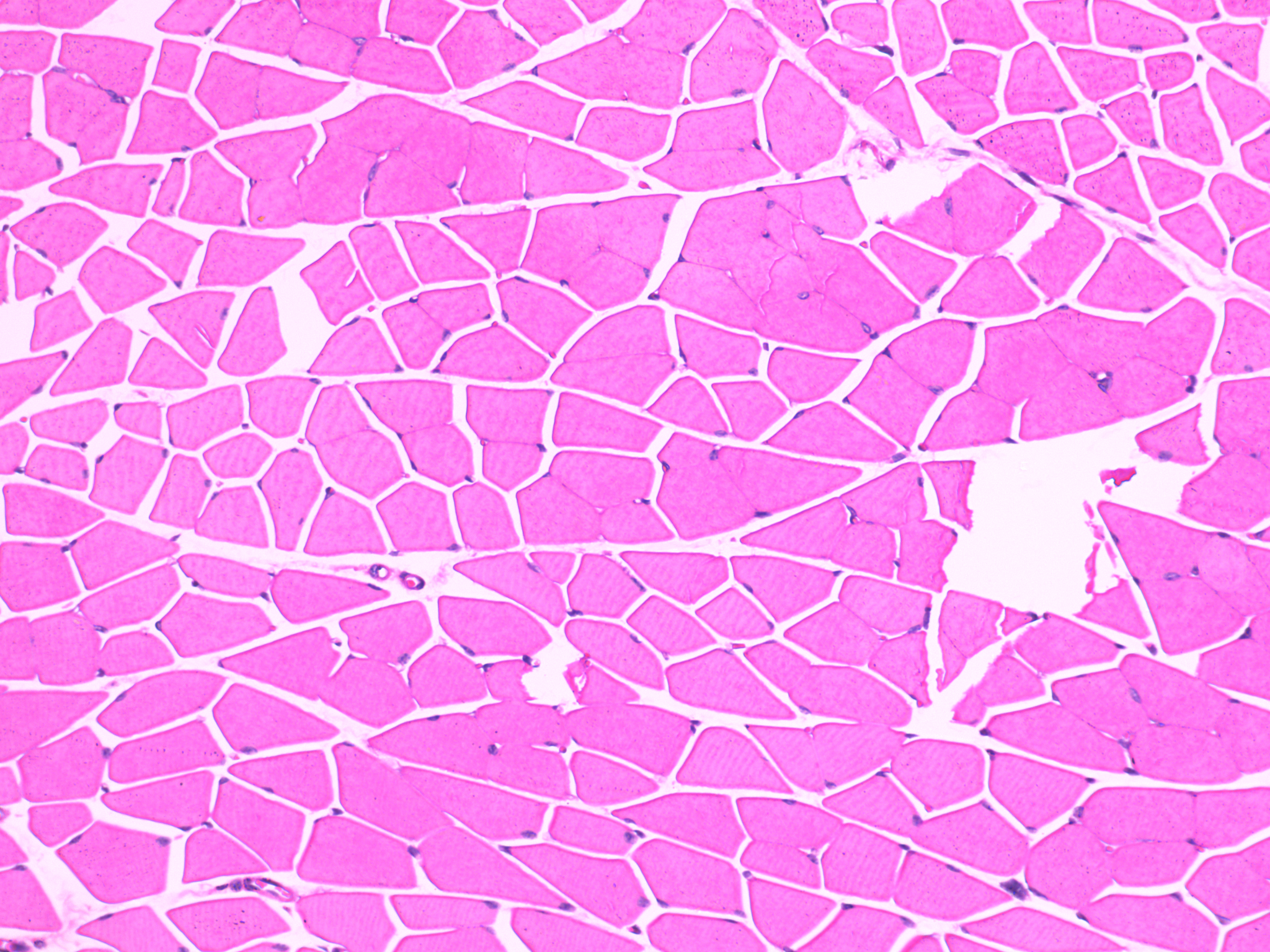

Supplement: Supplementary file 10 — Source data Fig. 3 [file 44321_2025_247_MOESM10_ESM.zip › Figure 3/Figure 3_Panel E/Figure 3_Panel E_HE_8w-Foxk2fl:fl-Myod1-Cre-Gas.tif]

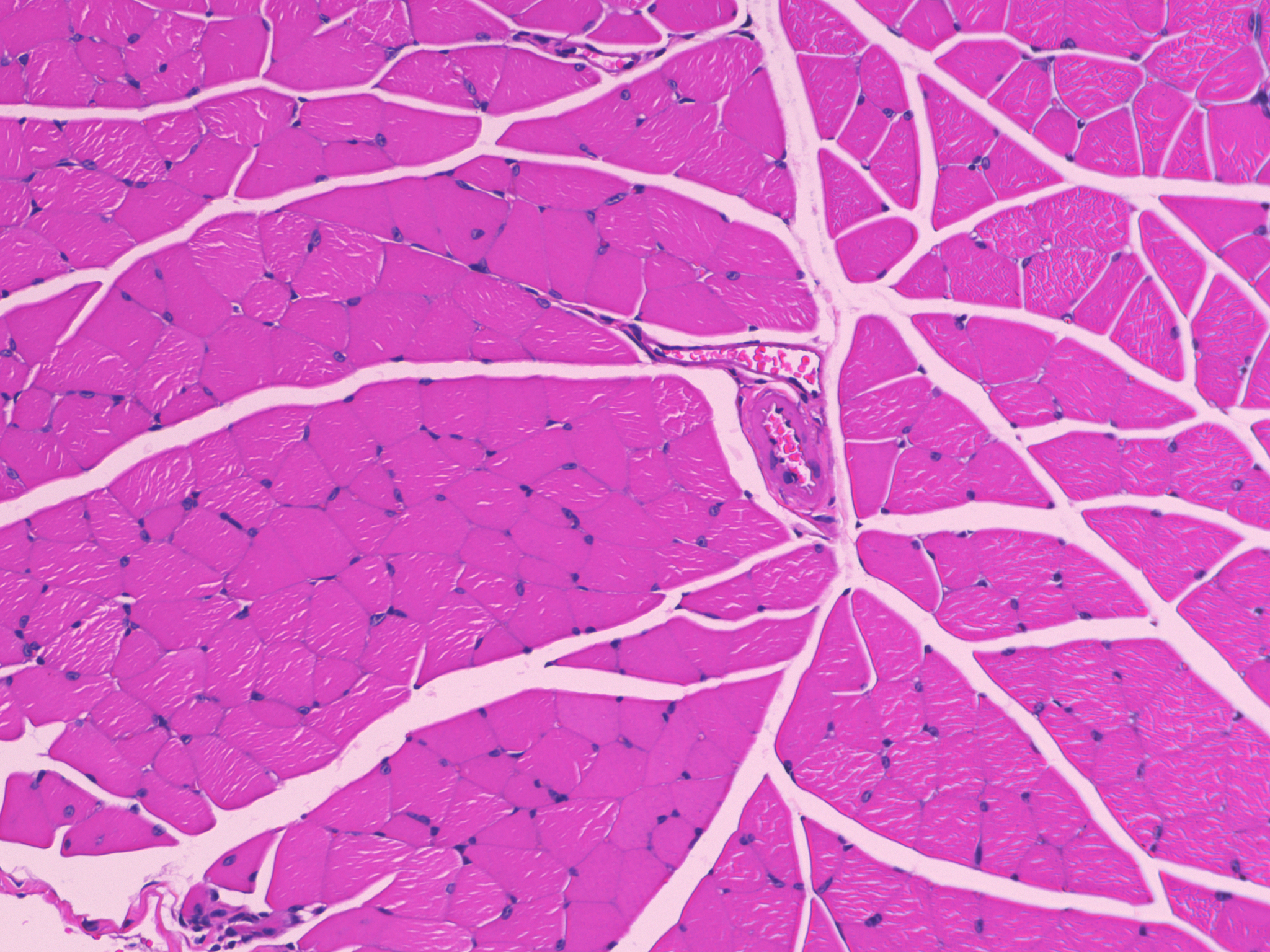

Supplement: Supplementary file 10 — Source data Fig. 3 [file 44321_2025_247_MOESM10_ESM.zip › Figure 3/Figure 3_Panel E/Figure 3_Panel E_HE_4w-Foxk2fl:fl-Myod1-Cre-Gas.tif]

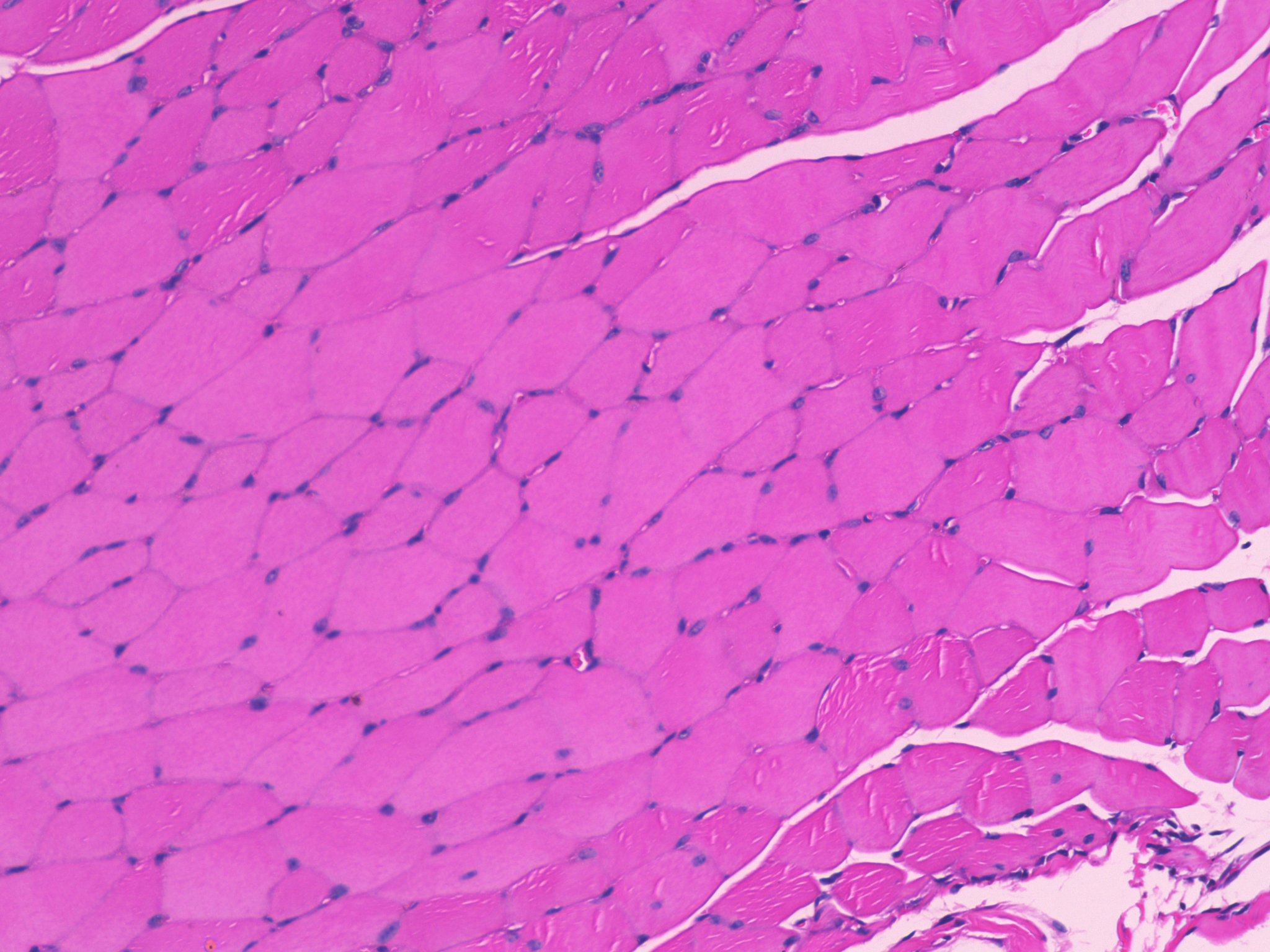

Supplement: Supplementary file 10 — Source data Fig. 3 [file 44321_2025_247_MOESM10_ESM.zip › Figure 3/Figure 3_Panel E/Figure 3_Panel E_HE_4w-Foxk2fl:fl-Myod1-Cre-TA.tif]

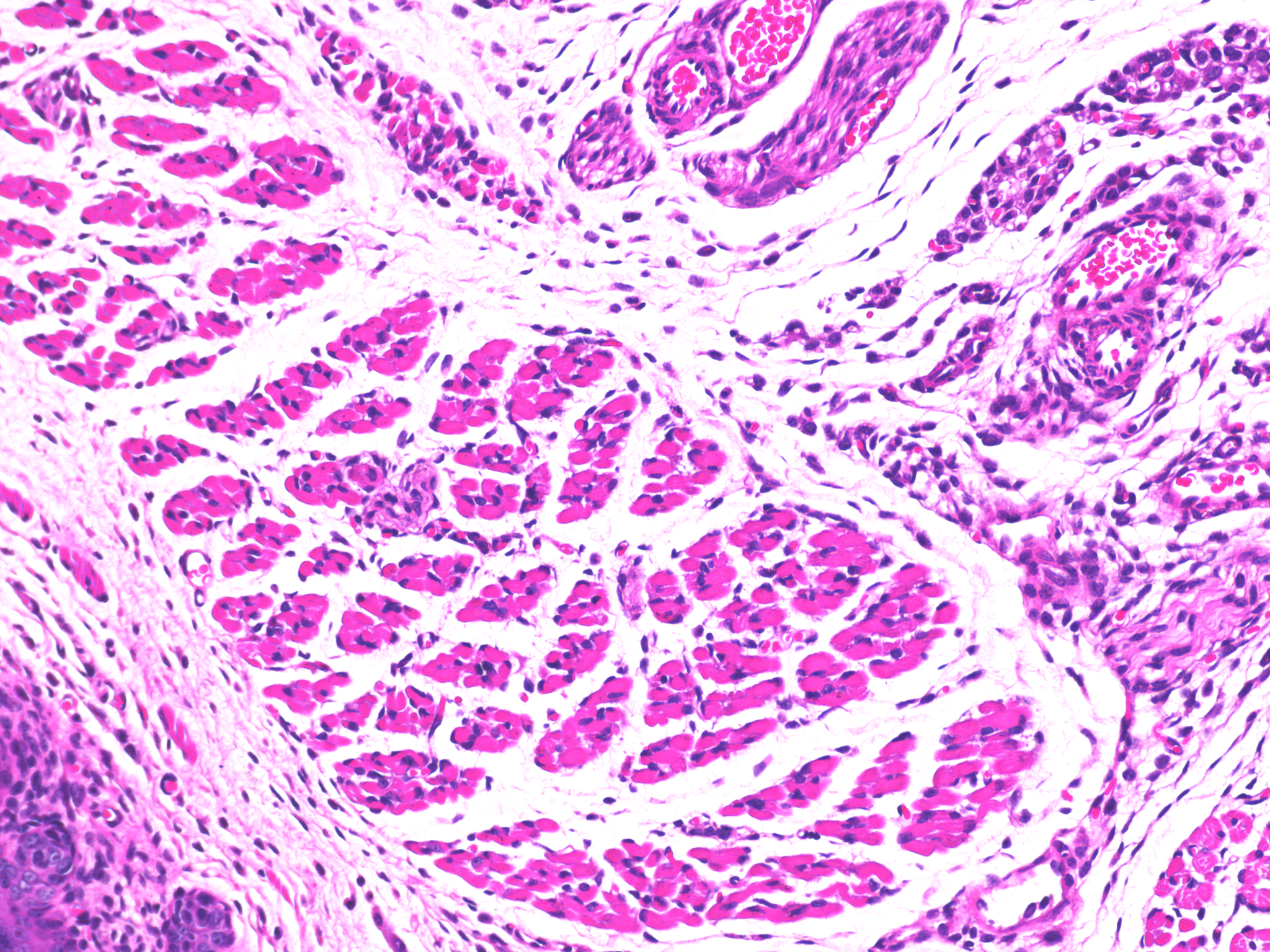

Supplement: Supplementary file 10 — Source data Fig. 3 [file 44321_2025_247_MOESM10_ESM.zip › Figure 3/Figure 3_Panel E/Figure 3_Panel E_HE_1d-Foxk2fl:fl-Myod1-Cre-TA.tif]

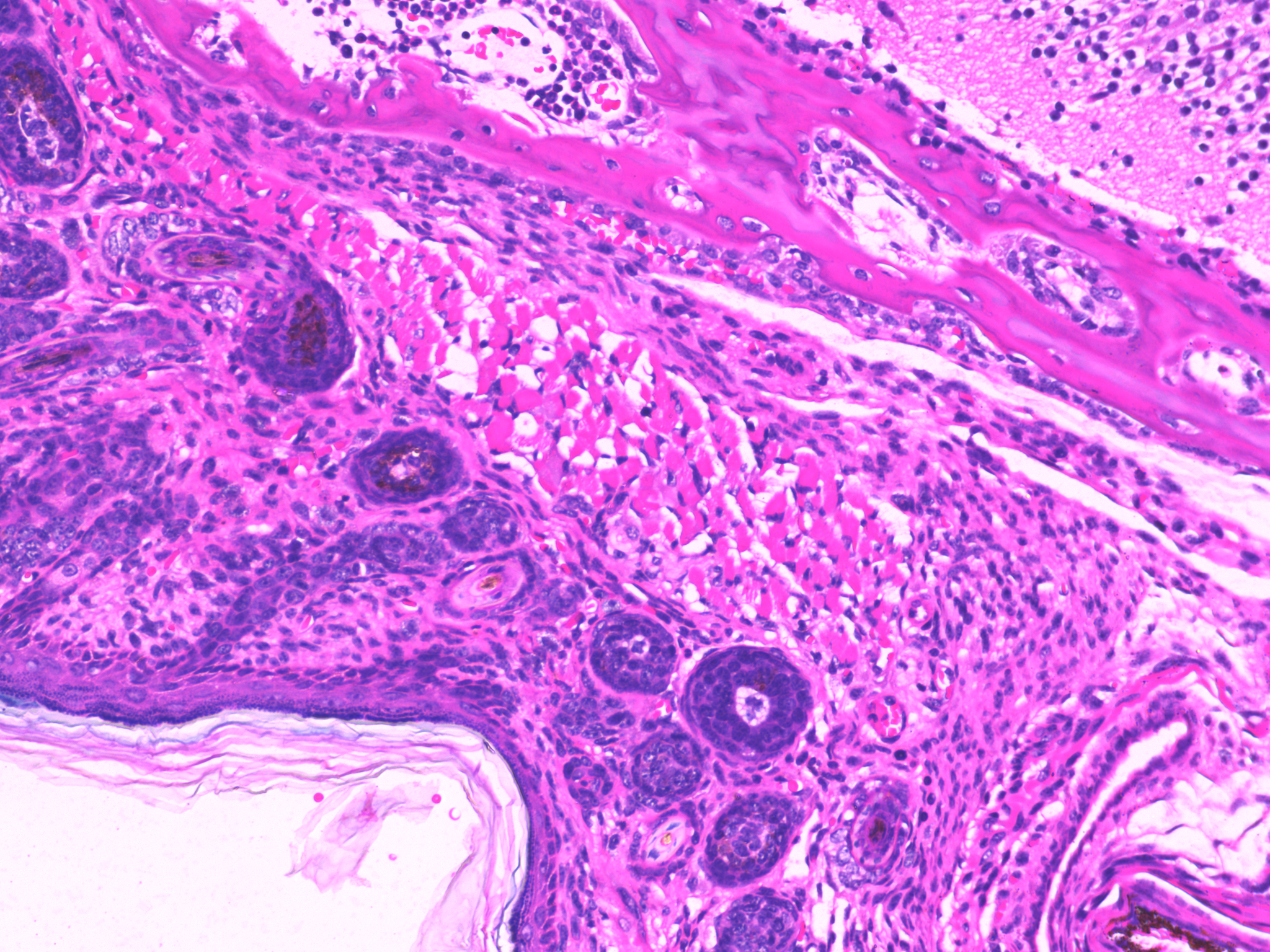

Supplement: Supplementary file 10 — Source data Fig. 3 [file 44321_2025_247_MOESM10_ESM.zip › Figure 3/Figure 3_Panel E/Figure 3_Panel E_HE_1d-Foxk2fl:fl_EM.tif]

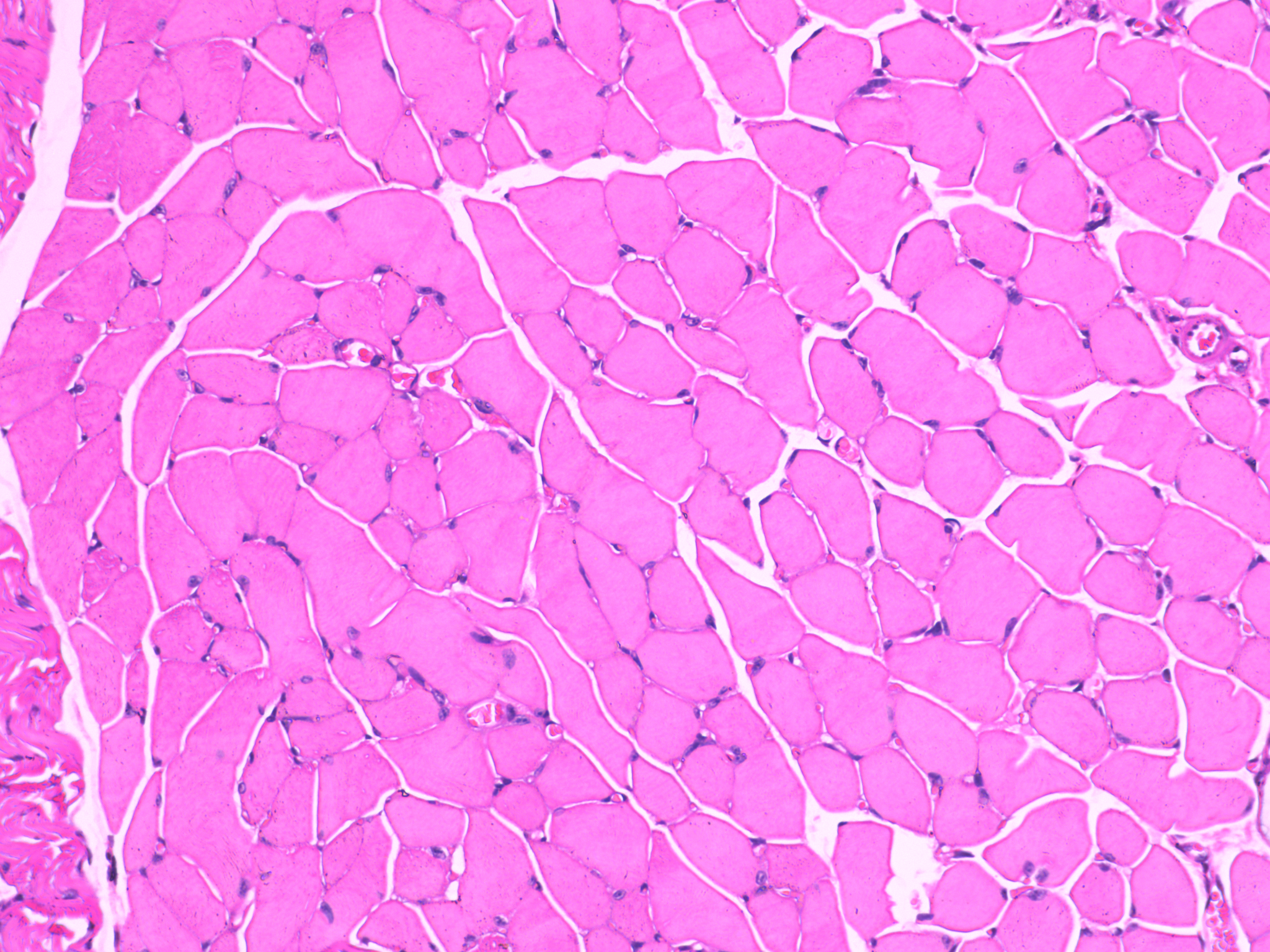

Supplement: Supplementary file 10 — Source data Fig. 3 [file 44321_2025_247_MOESM10_ESM.zip › Figure 3/Figure 3_Panel E/Figure 3_Panel E_HE_8w-Foxk2fl:fl-Gas.tif]

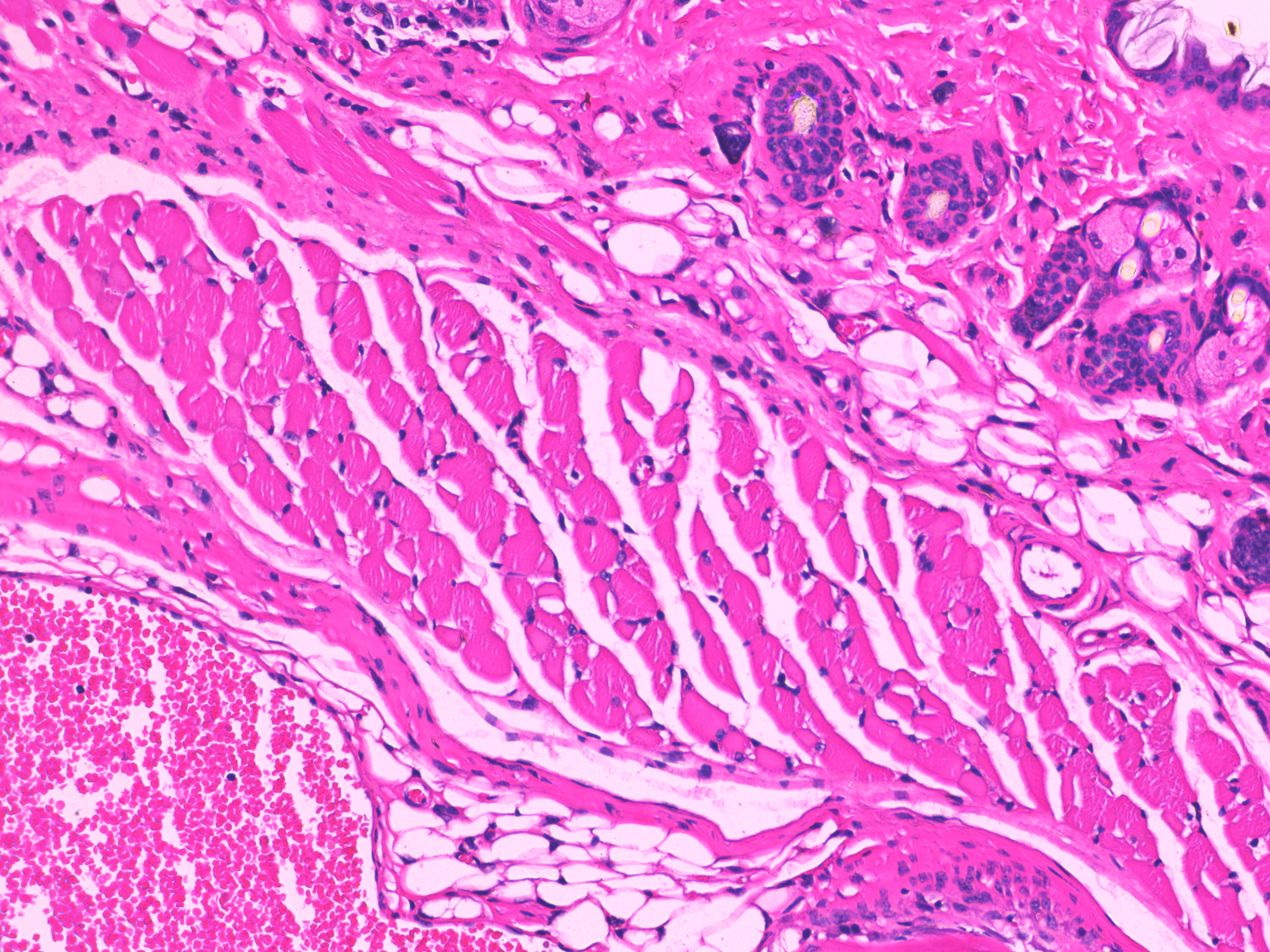

Supplement: Supplementary file 10 — Source data Fig. 3 [file 44321_2025_247_MOESM10_ESM.zip › Figure 3/Figure 3_Panel E/Figure 3_Panel E_HE_4w-Foxk2fl:fl-EM.tif]

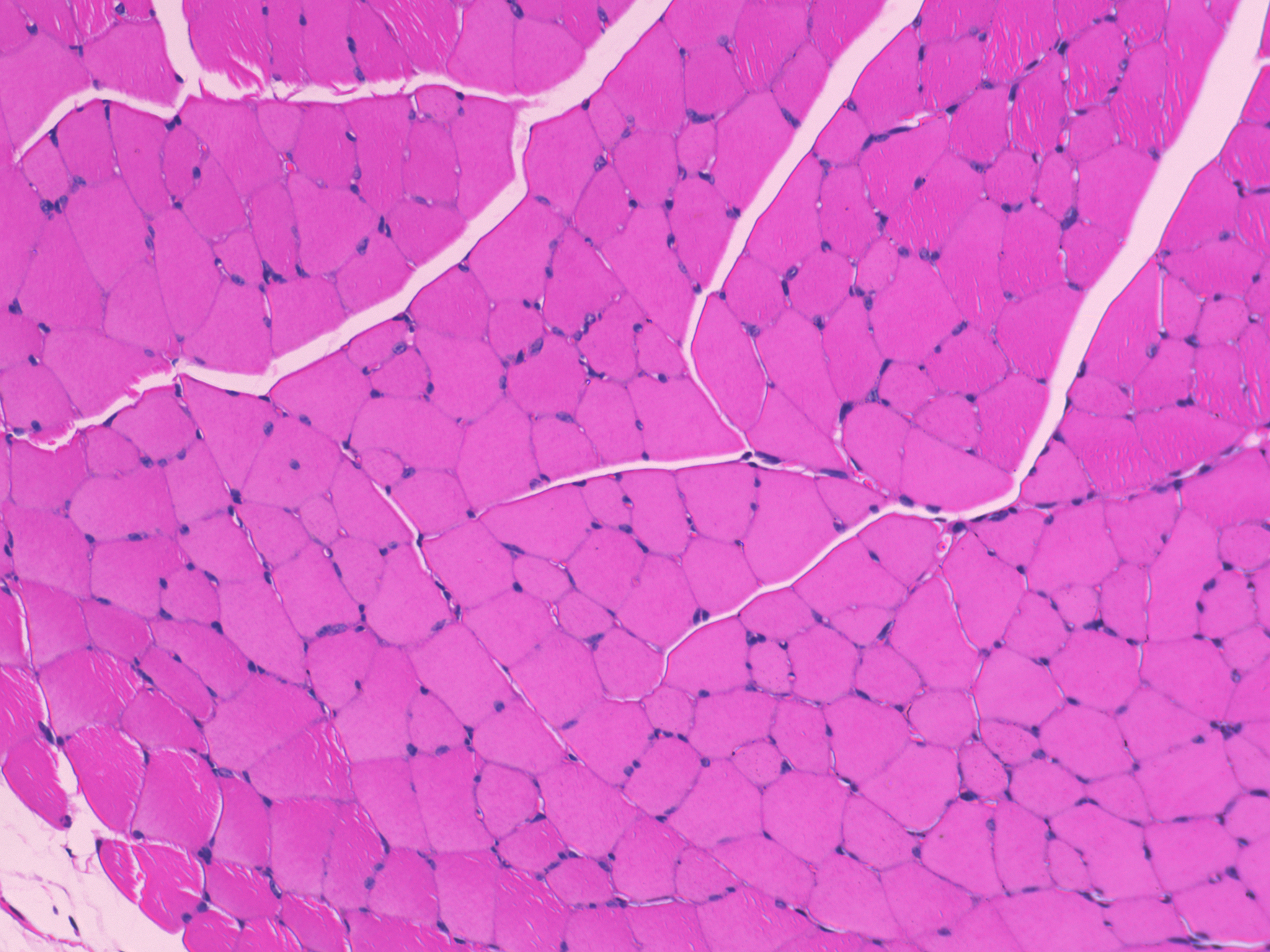

Supplement: Supplementary file 10 — Source data Fig. 3 [file 44321_2025_247_MOESM10_ESM.zip › Figure 3/Figure 3_Panel E/Figure 3_Panel E_HE_4w-Foxk2fl:fl-TA.tif]

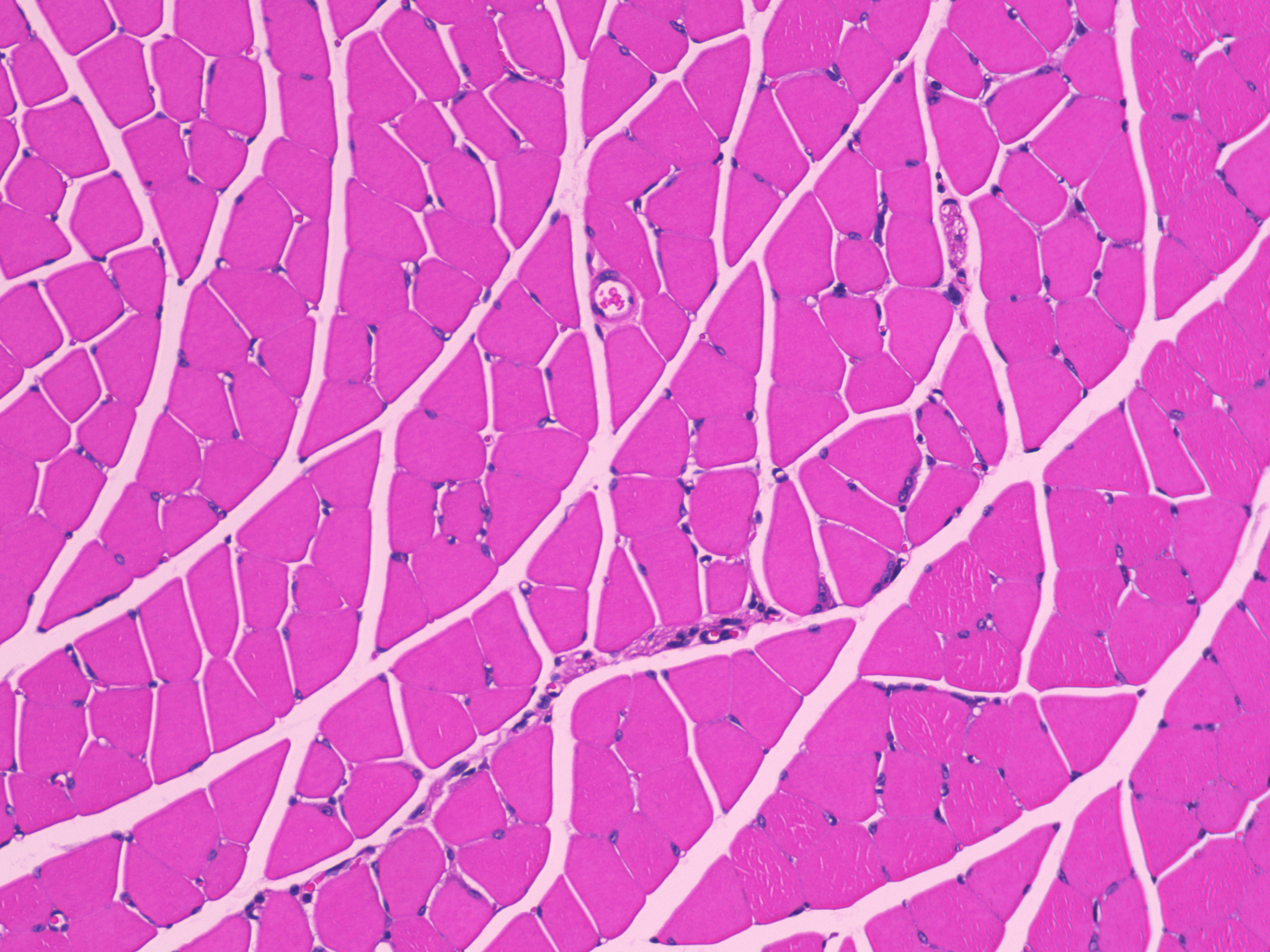

Supplement: Supplementary file 10 — Source data Fig. 3 [file 44321_2025_247_MOESM10_ESM.zip › Figure 3/Figure 3_Panel E/Figure 3_Panel E_HE_4w-Foxk2fl:fl-Gas.tif]

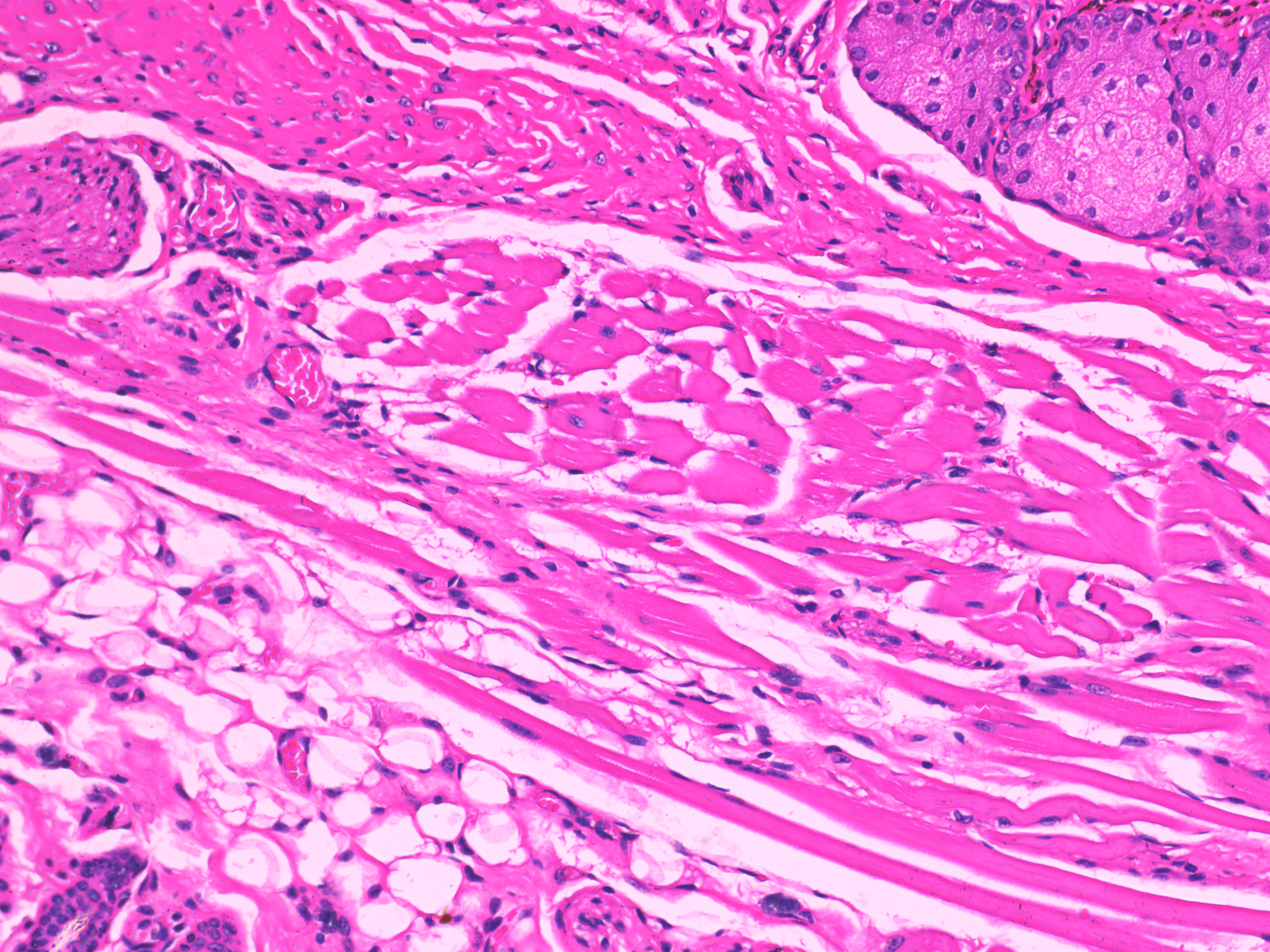

Supplement: Supplementary file 10 — Source data Fig. 3 [file 44321_2025_247_MOESM10_ESM.zip › Figure 3/Figure 3_Panel E/Figure 3_Panel E_HE_4w-Foxk2fl:fl-Myod1-Cre-EM.tif]

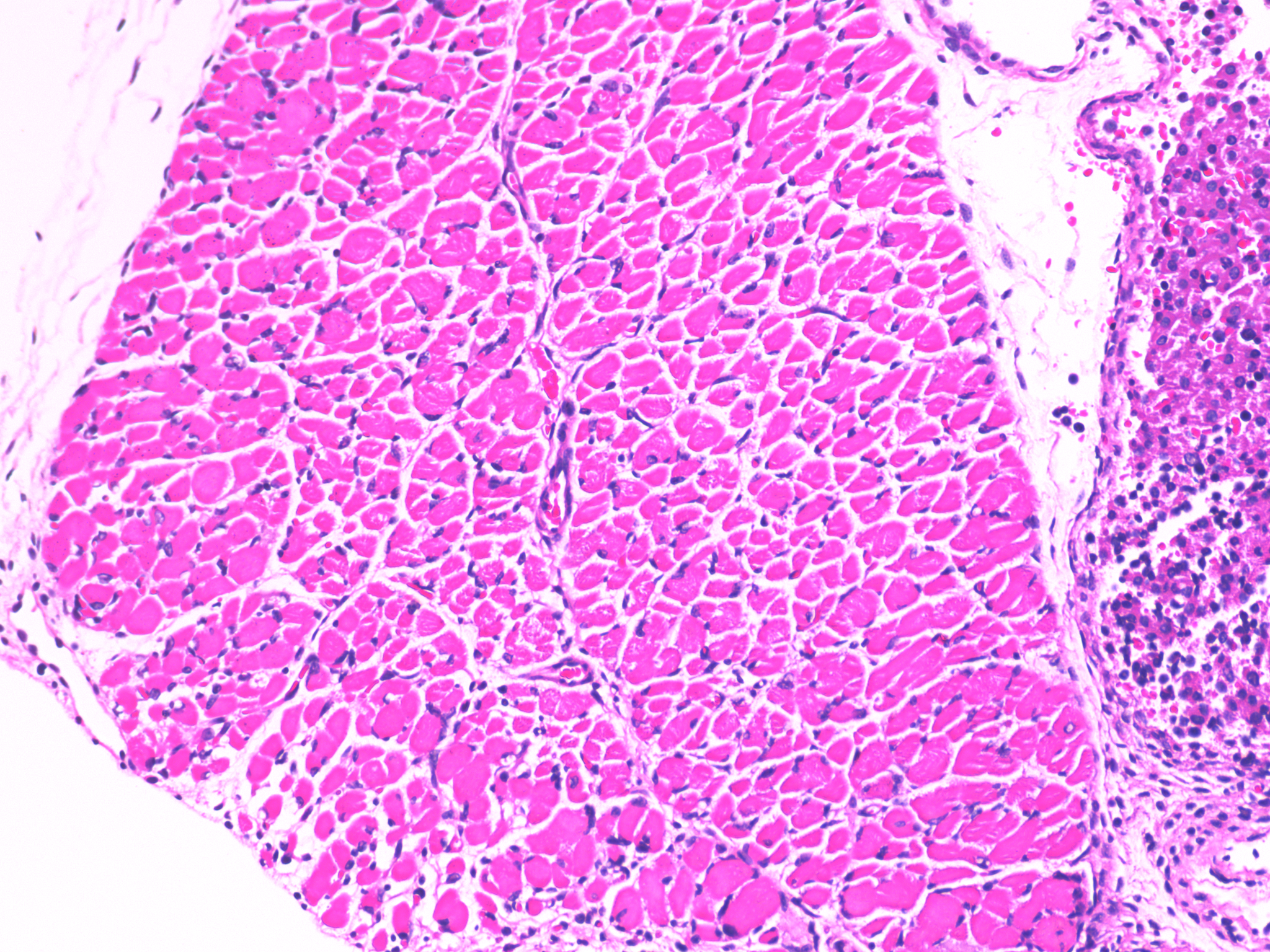

Supplement: Supplementary file 10 — Source data Fig. 3 [file 44321_2025_247_MOESM10_ESM.zip › Figure 3/Figure 3_Panel E/Figure 3_Panel E_HE_1d-Foxk2fl:fl-Myod1-Cre-Gas.tif]

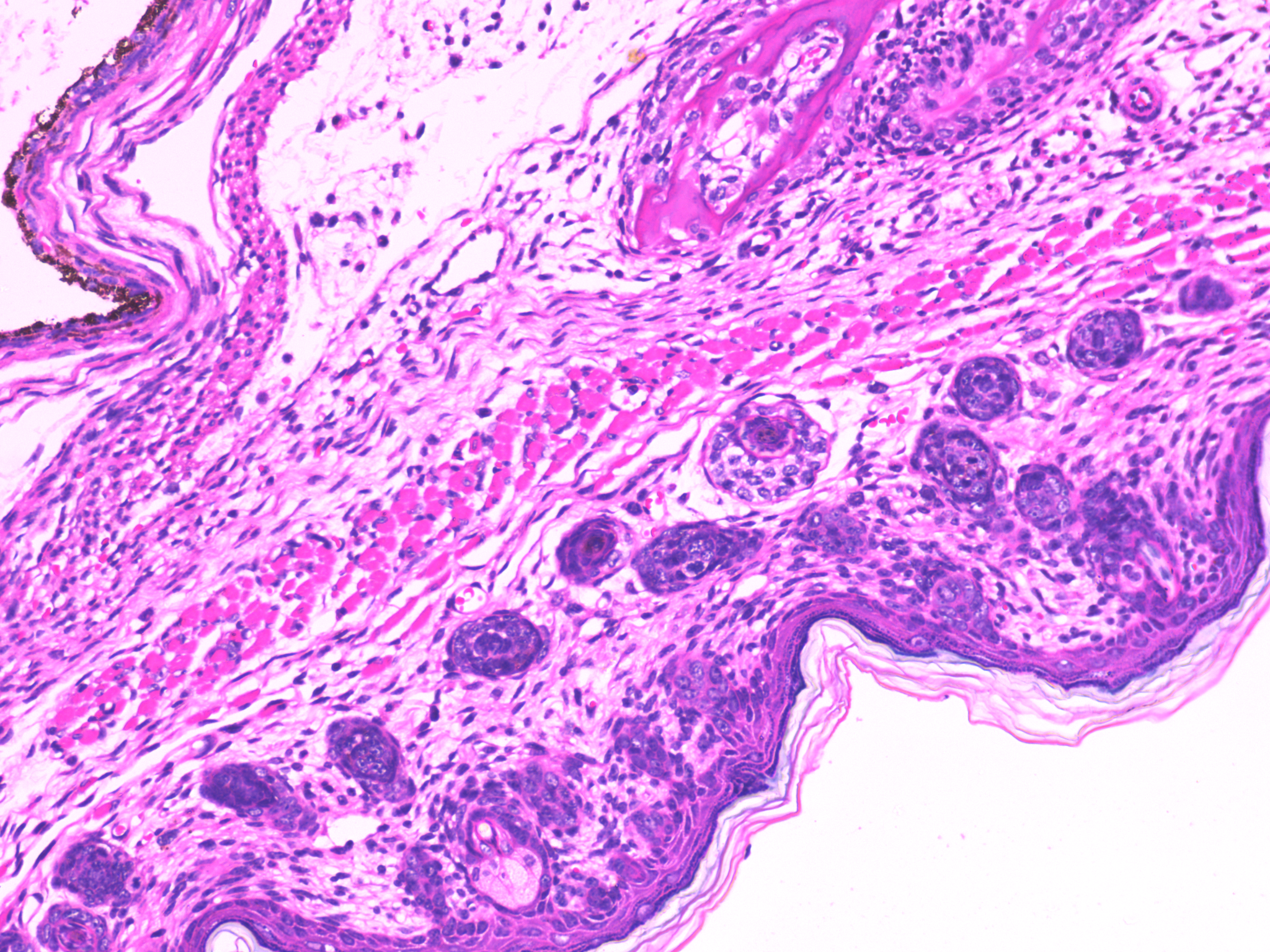

Supplement: Supplementary file 10 — Source data Fig. 3 [file 44321_2025_247_MOESM10_ESM.zip › Figure 3/Figure 3_Panel E/Figure 3_Panel E_HE_1d-Foxk2fl:fl-Myod1-Cre-EM.tif]

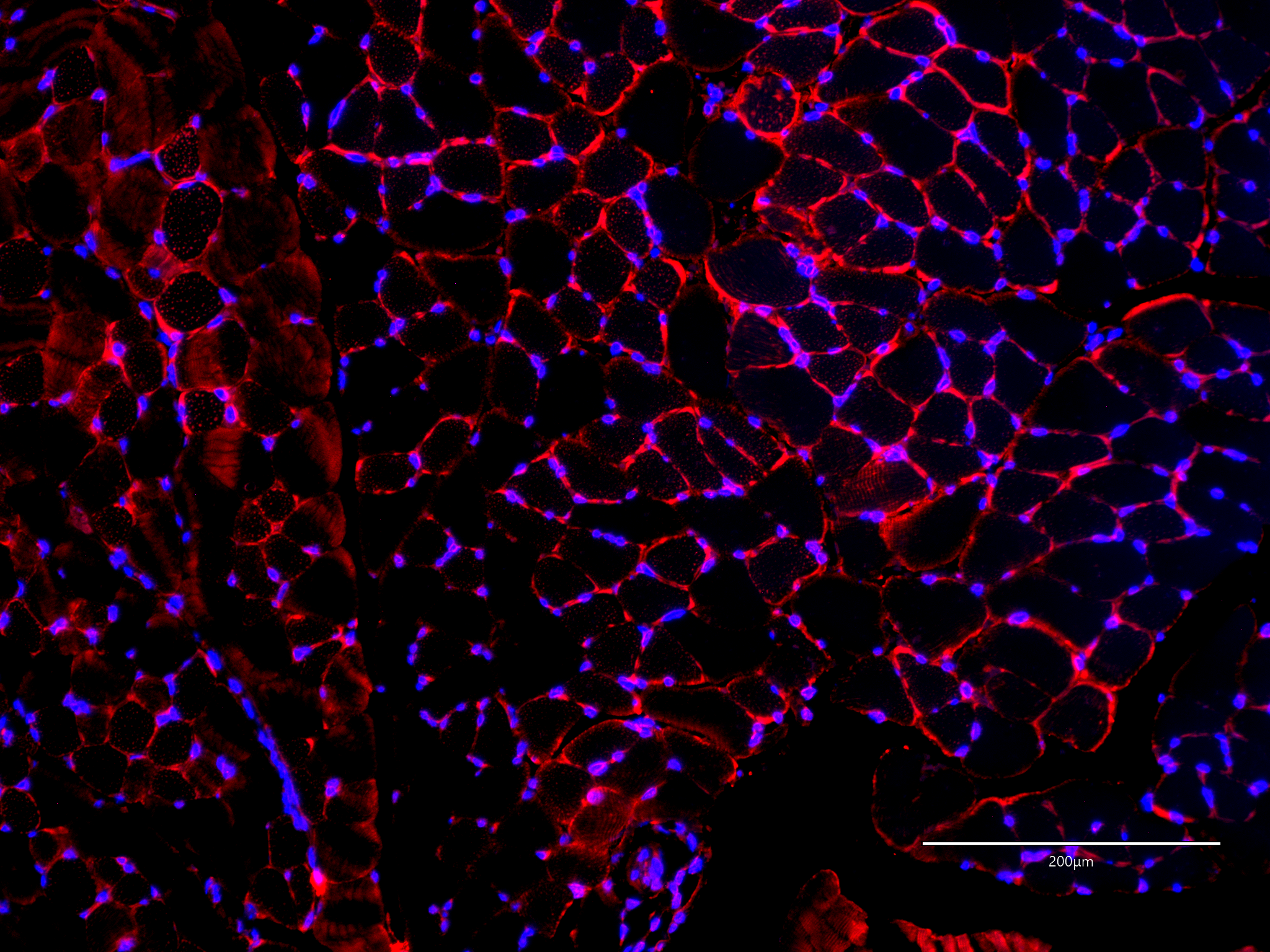

Supplement: Supplementary file 10 — Source data Fig. 3 [file 44321_2025_247_MOESM10_ESM.zip › Figure 3/Figure 3_Panel G/Figure 3_Panel G_IF-LAMININ_Foxk2fl:fl-Myod1-Cre.tif]

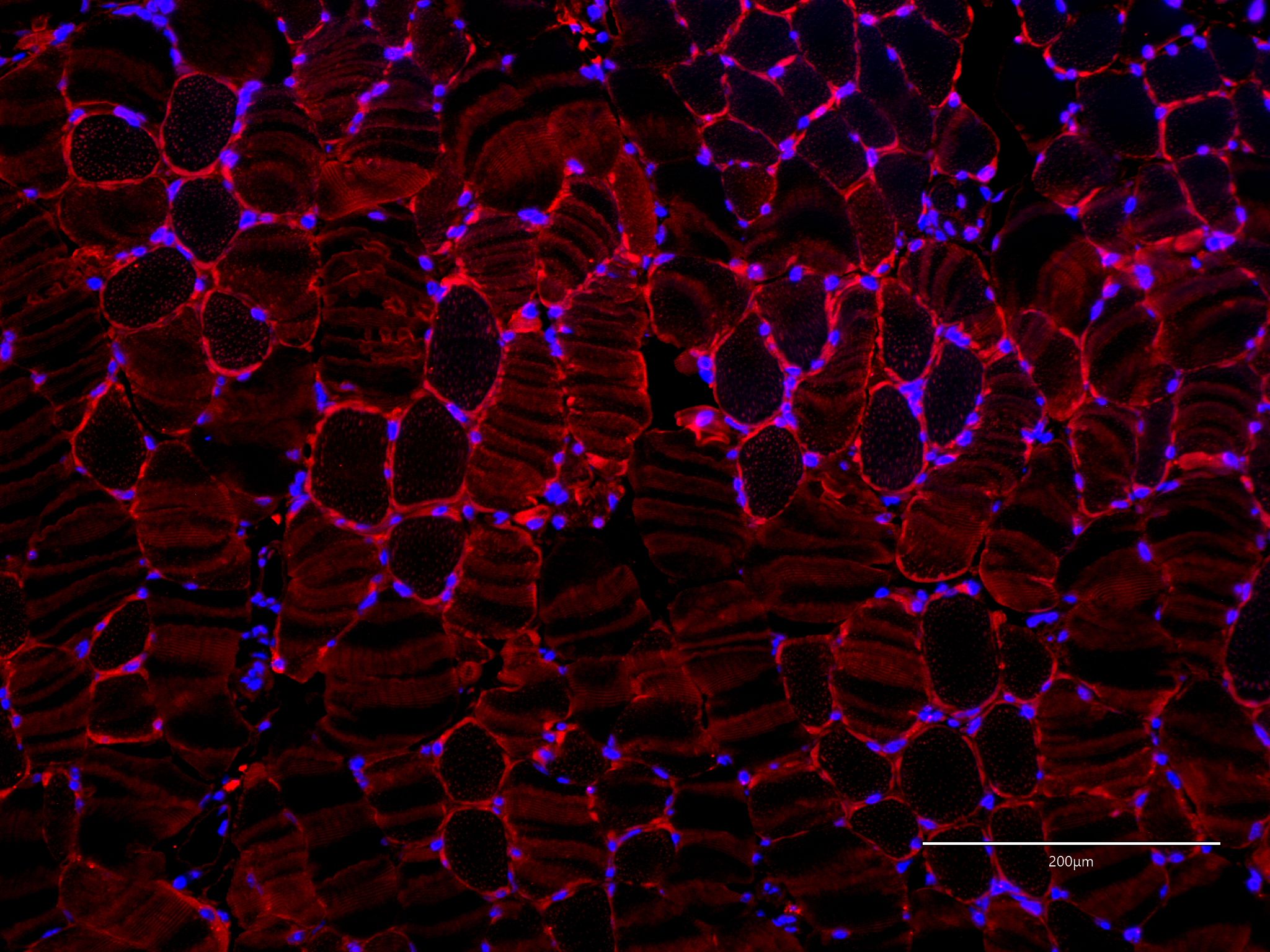

Supplement: Supplementary file 10 — Source data Fig. 3 [file 44321_2025_247_MOESM10_ESM.zip › Figure 3/Figure 3_Panel G/Figure 3_Panel G_IF-LAMININ_Foxk2fl:fl.tif]

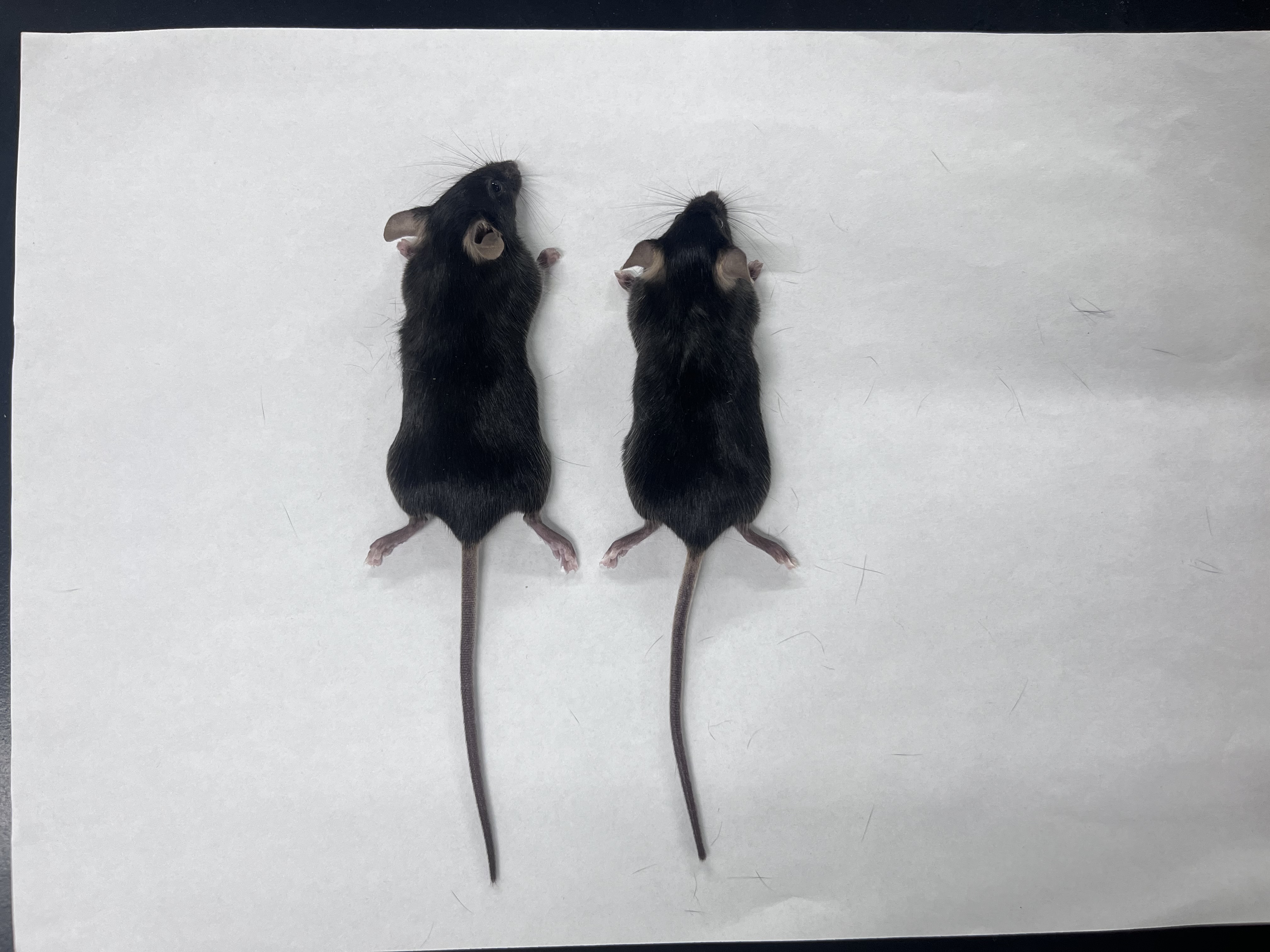

Supplement: Supplementary file 10 — Source data Fig. 3 [file 44321_2025_247_MOESM10_ESM.zip › Figure 3/Figure 3_Panel A/Figure 3_Panel A_mouse.jpg]

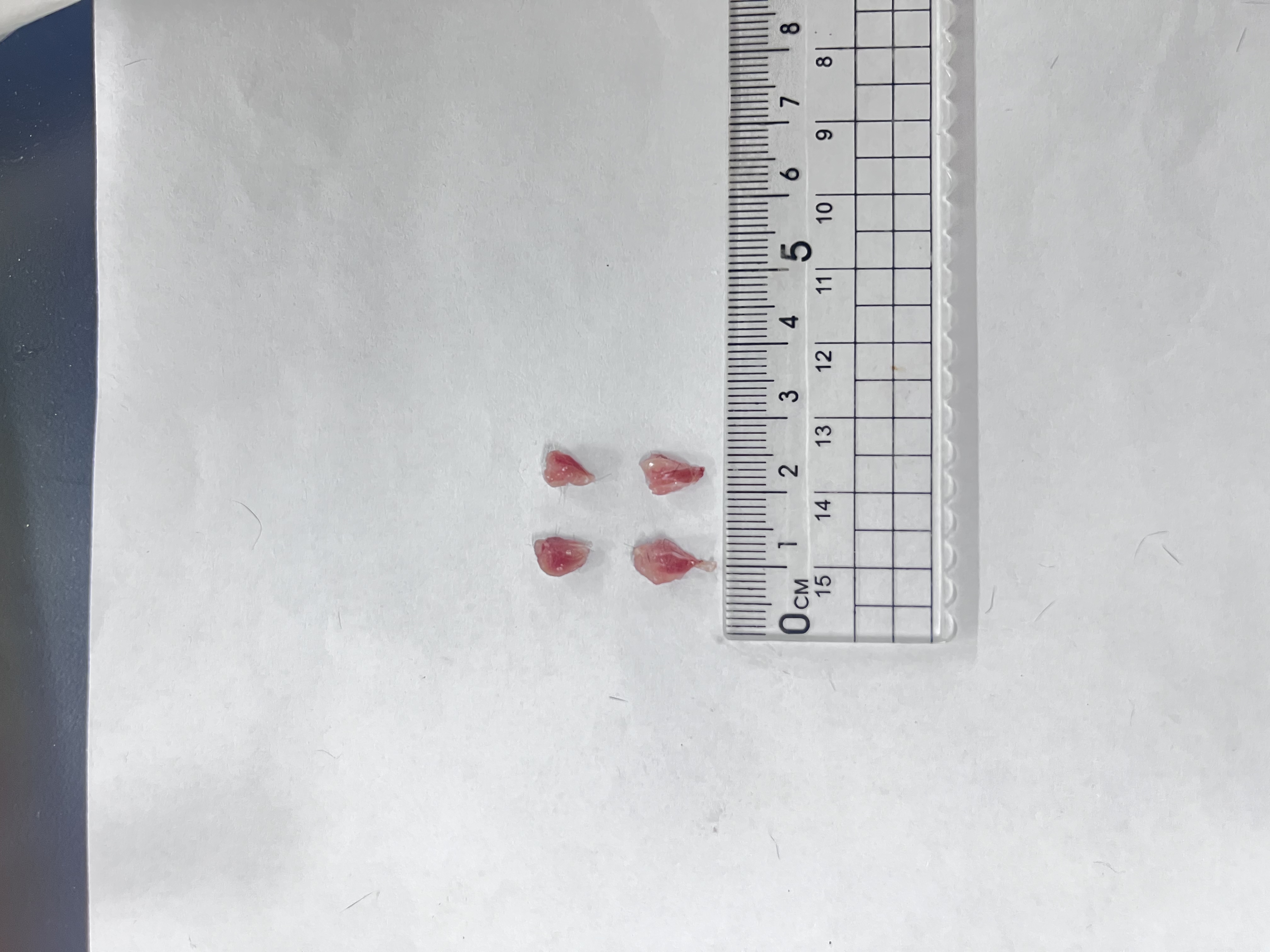

Supplement: Supplementary file 10 — Source data Fig. 3 [file 44321_2025_247_MOESM10_ESM.zip › Figure 3/Figure 3_Panel A/Figure 3_Panel A_tissues.jpg]

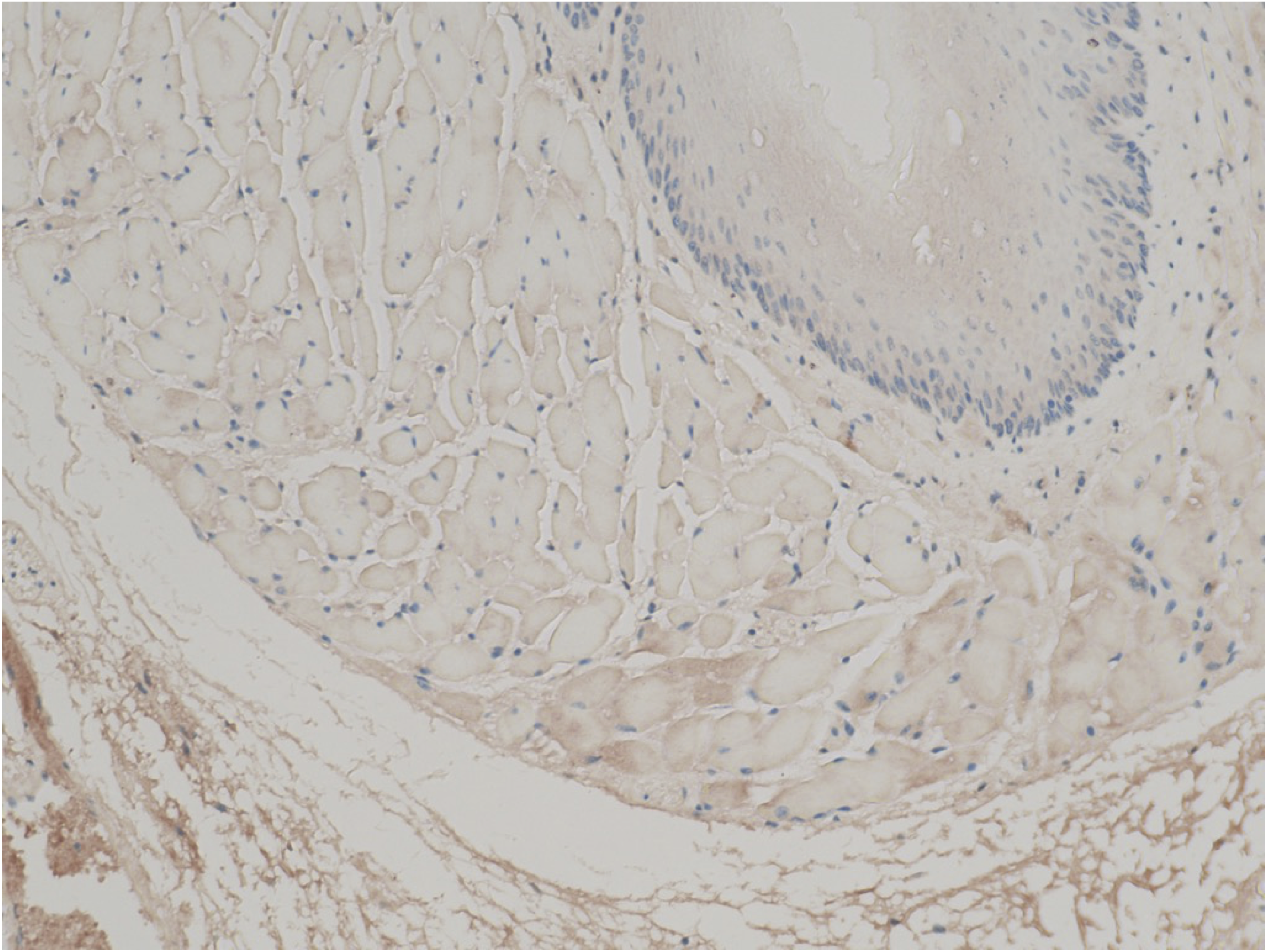

Supplement: Supplementary file 10 — Source data Fig. 3 [file 44321_2025_247_MOESM10_ESM.zip › Figure 3/Figure 3_Panel F/Figure 3_Panel F_IHC_Foxk2fl:fl-EM.tif]

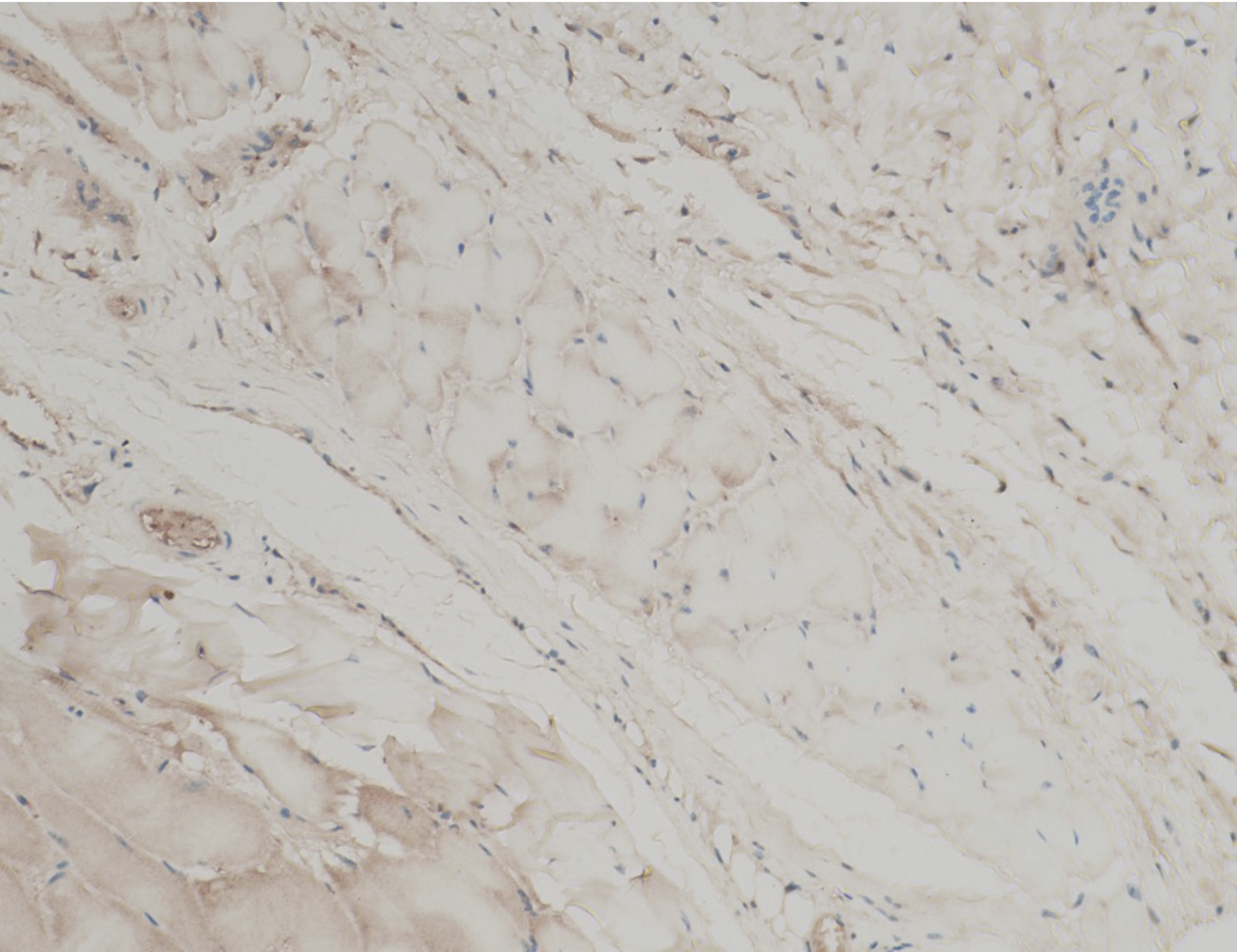

Supplement: Supplementary file 10 — Source data Fig. 3 [file 44321_2025_247_MOESM10_ESM.zip › Figure 3/Figure 3_Panel F/Figure 3_Panel F_IHC_Foxk2fl:fl-Myod1-Cre-EM.tif]

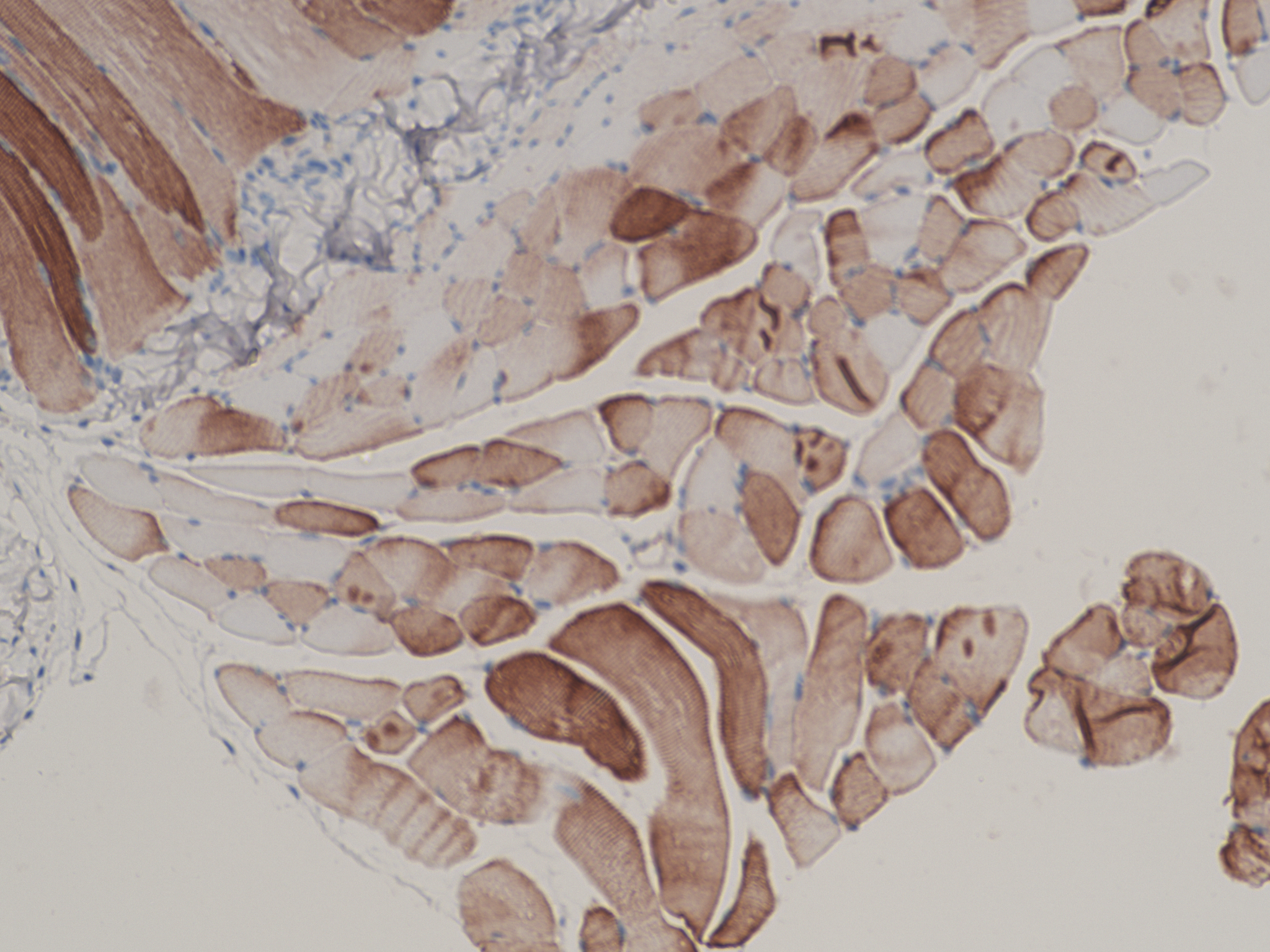

Supplement: Supplementary file 10 — Source data Fig. 3 [file 44321_2025_247_MOESM10_ESM.zip › Figure 3/Figure 3_Panel F/Figure 3_Panel F_IHC_Foxk2fl:fl-Gas.tif]

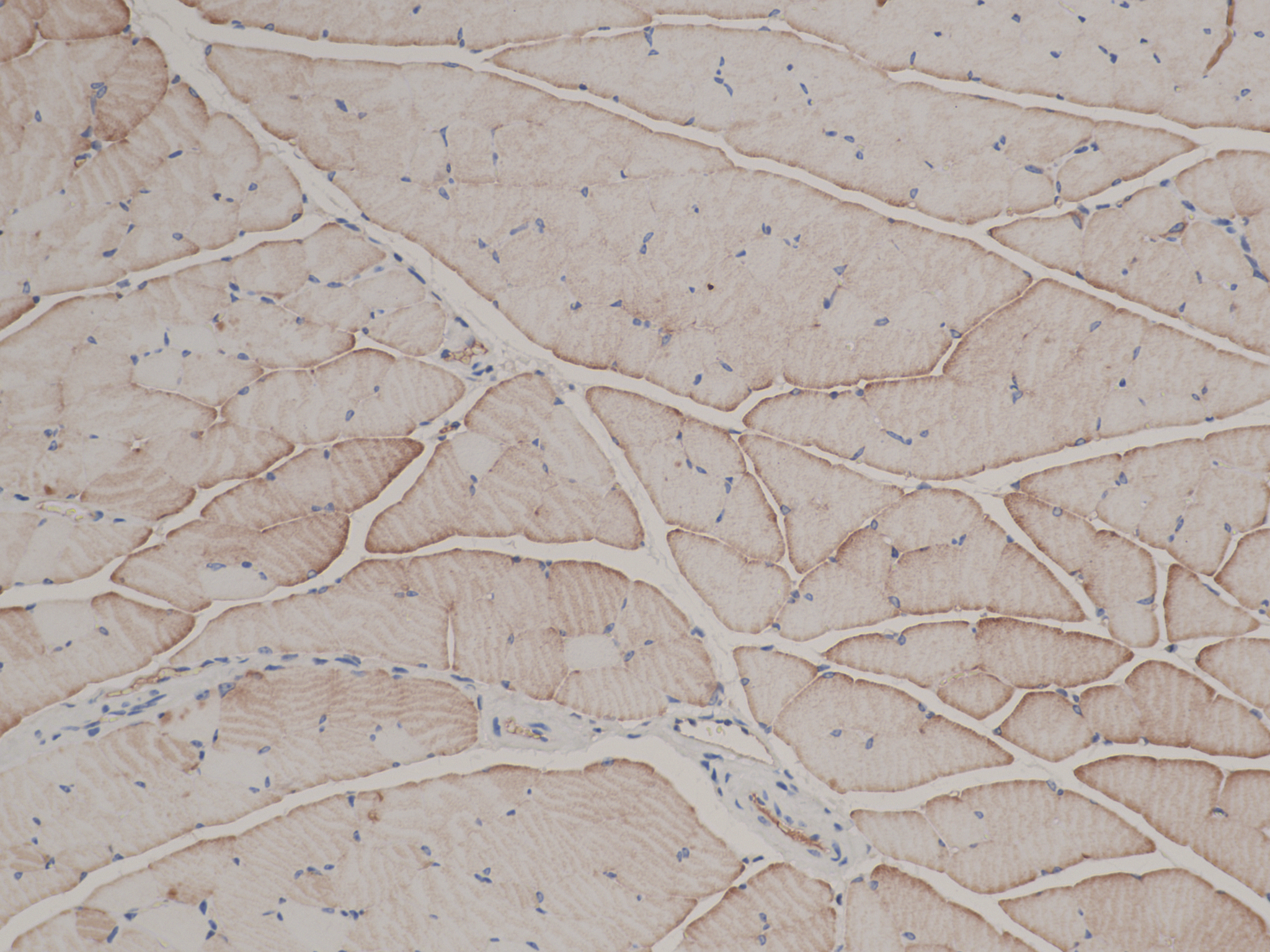

Supplement: Supplementary file 10 — Source data Fig. 3 [file 44321_2025_247_MOESM10_ESM.zip › Figure 3/Figure 3_Panel F/Figure 3_Panel F_IHC_Foxk2fl:fl-Myod1-Cre-Gas.tif]

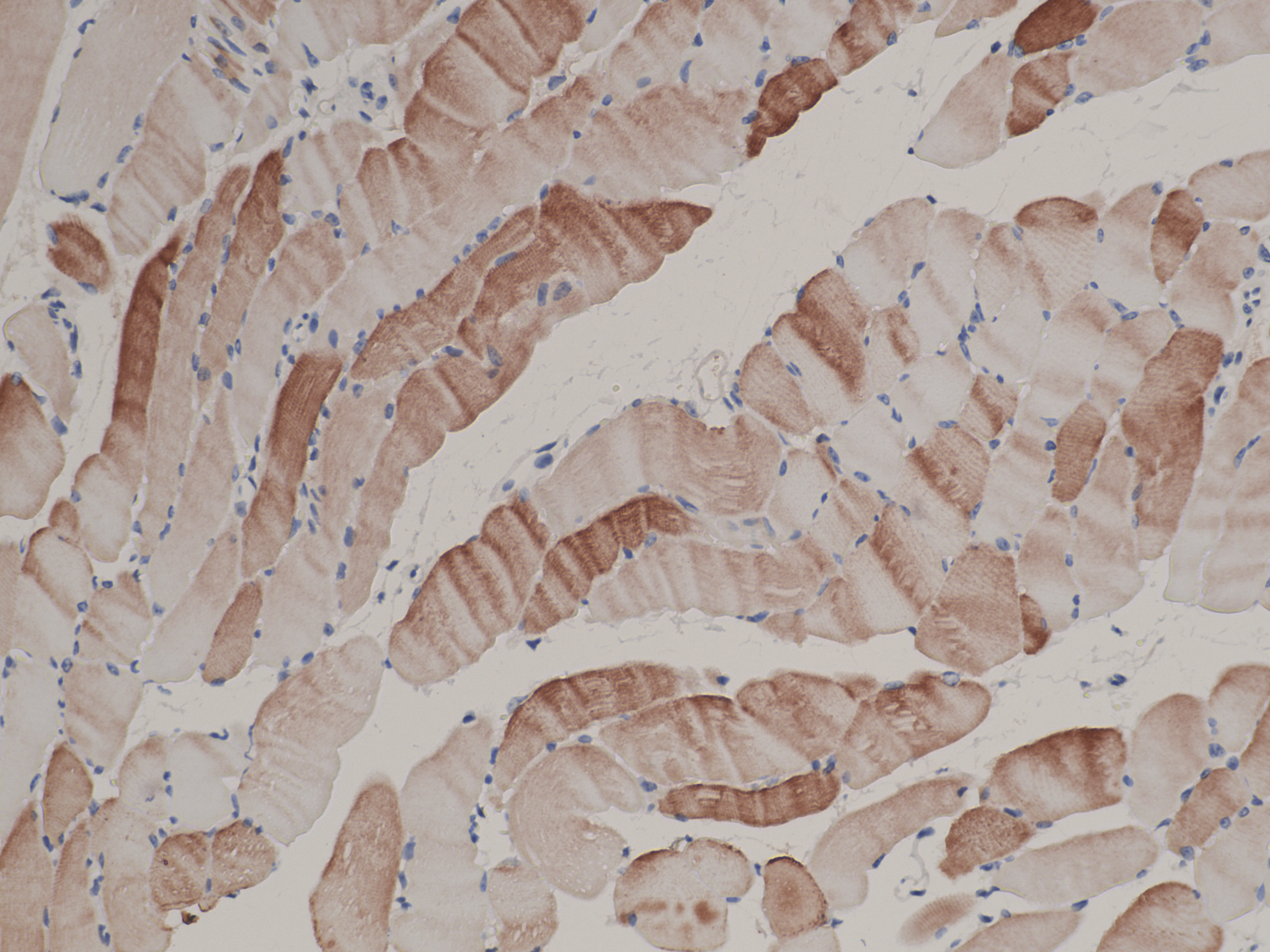

Supplement: Supplementary file 10 — Source data Fig. 3 [file 44321_2025_247_MOESM10_ESM.zip › Figure 3/Figure 3_Panel F/Figure 3_Panel F_IHC_Foxk2fl:fl-TA.tif]

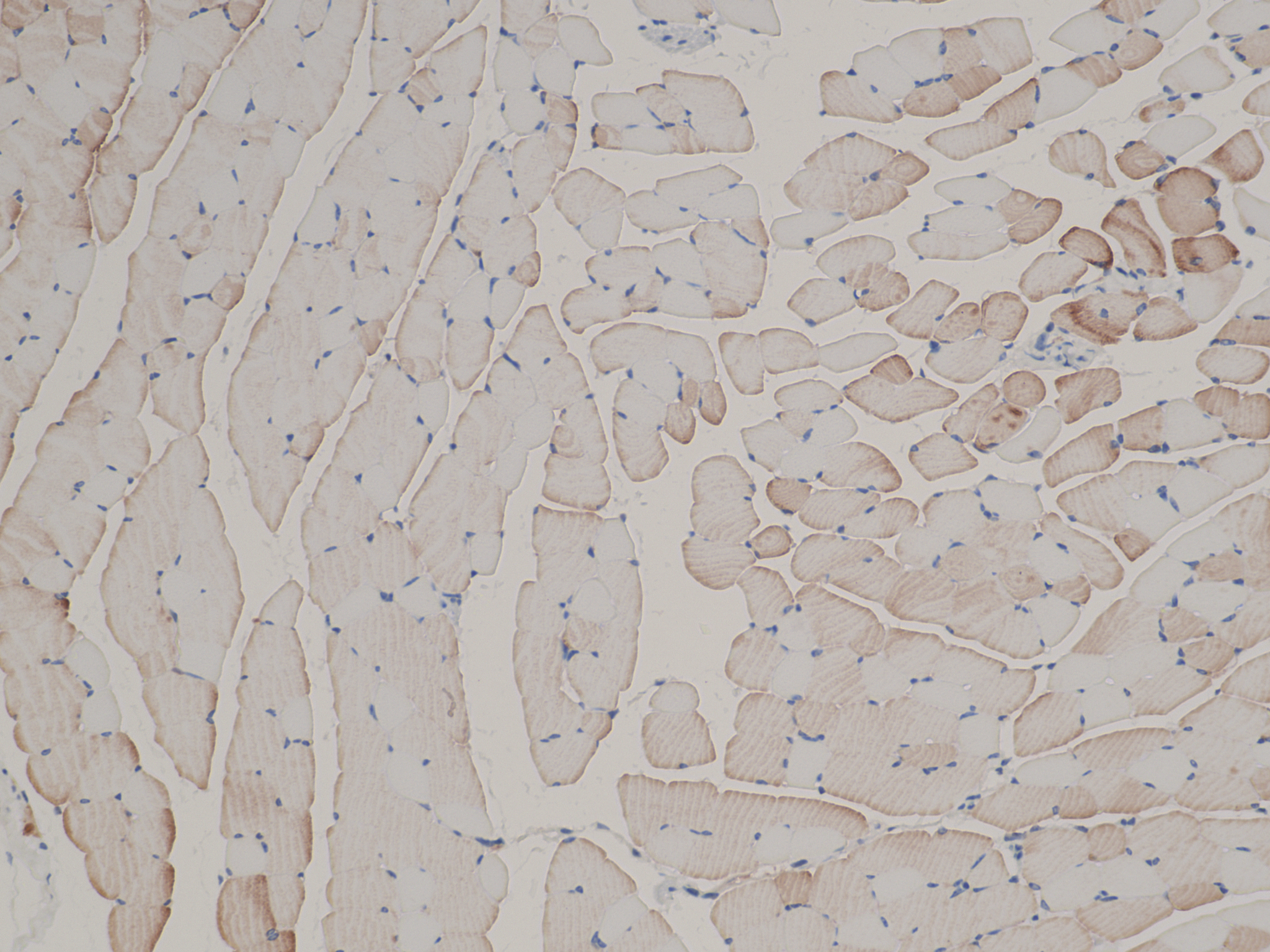

Supplement: Supplementary file 10 — Source data Fig. 3 [file 44321_2025_247_MOESM10_ESM.zip › Figure 3/Figure 3_Panel F/Figure 3_Panel F_IHC_Foxk2fl:fl-Myod1-Cre-TA.tif]

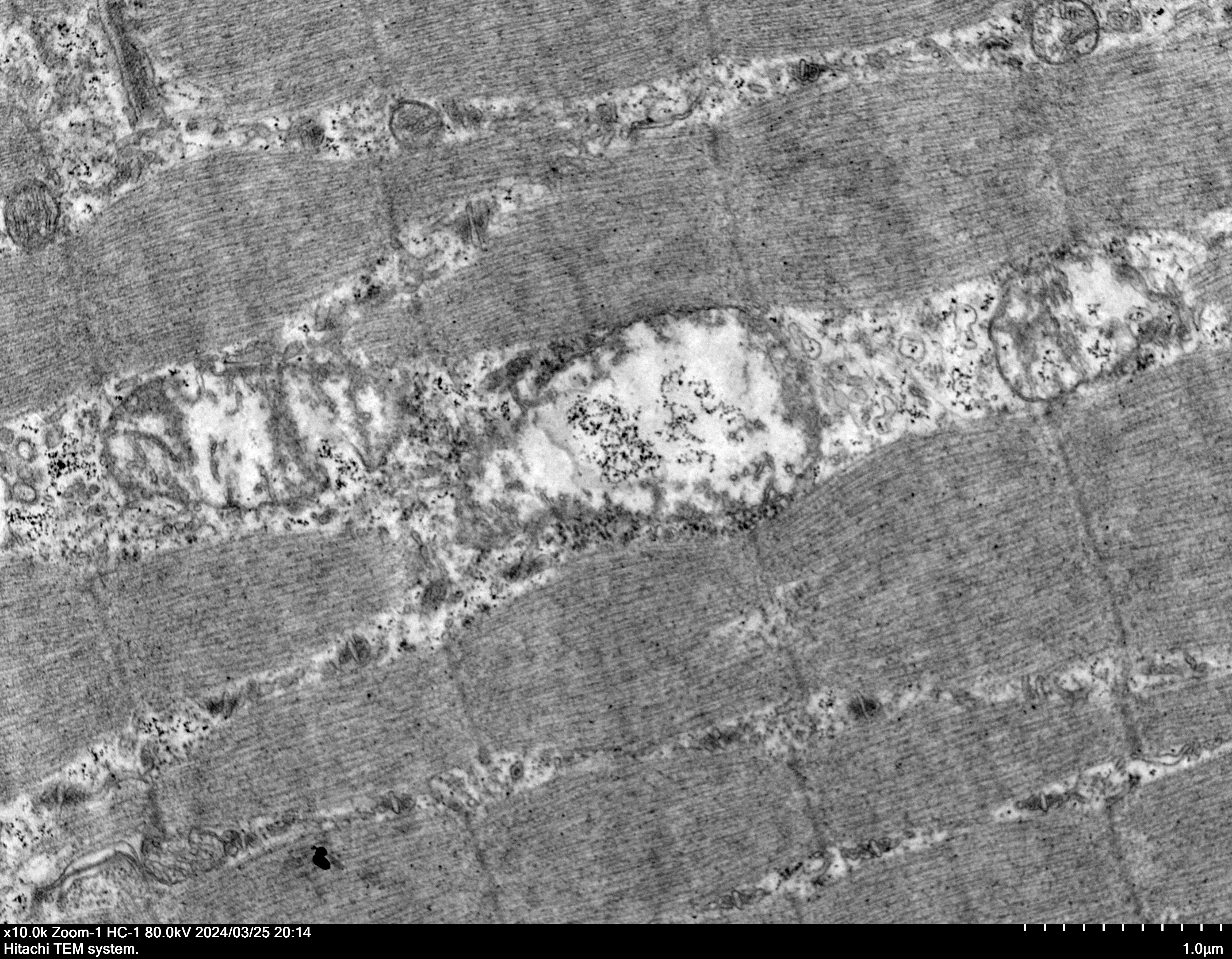

Supplement: Supplementary file 11 — Source data Fig. 4 [file 44321_2025_247_MOESM11_ESM.zip › Figure 4/Figure 4_Panel A/Figure 4_Panel A_TEM_Foxk2fl:fl-Myod1-Cre.tif]

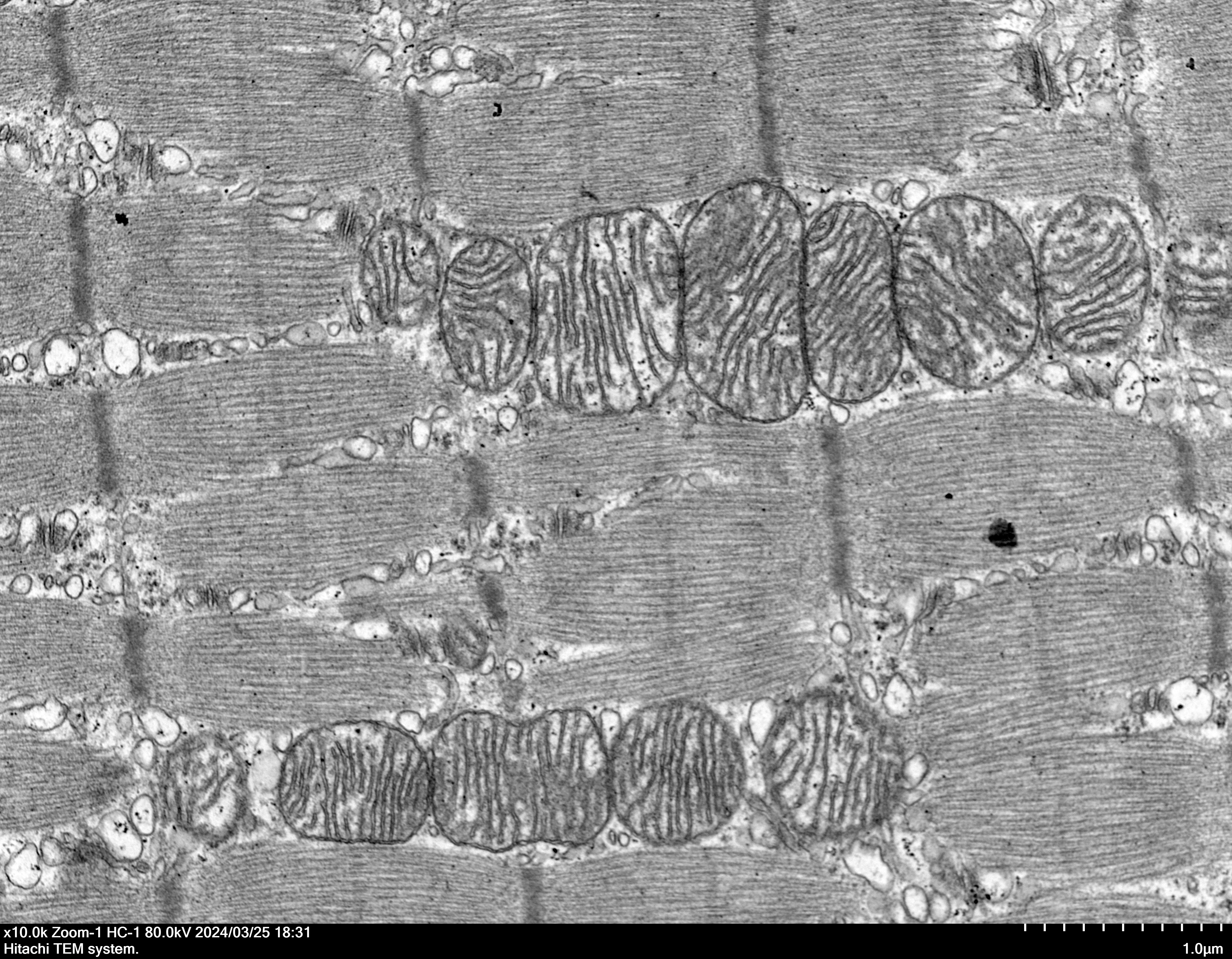

Supplement: Supplementary file 11 — Source data Fig. 4 [file 44321_2025_247_MOESM11_ESM.zip › Figure 4/Figure 4_Panel A/Figure 4_Panel A_TEM_Foxk2fl:fl.tif]

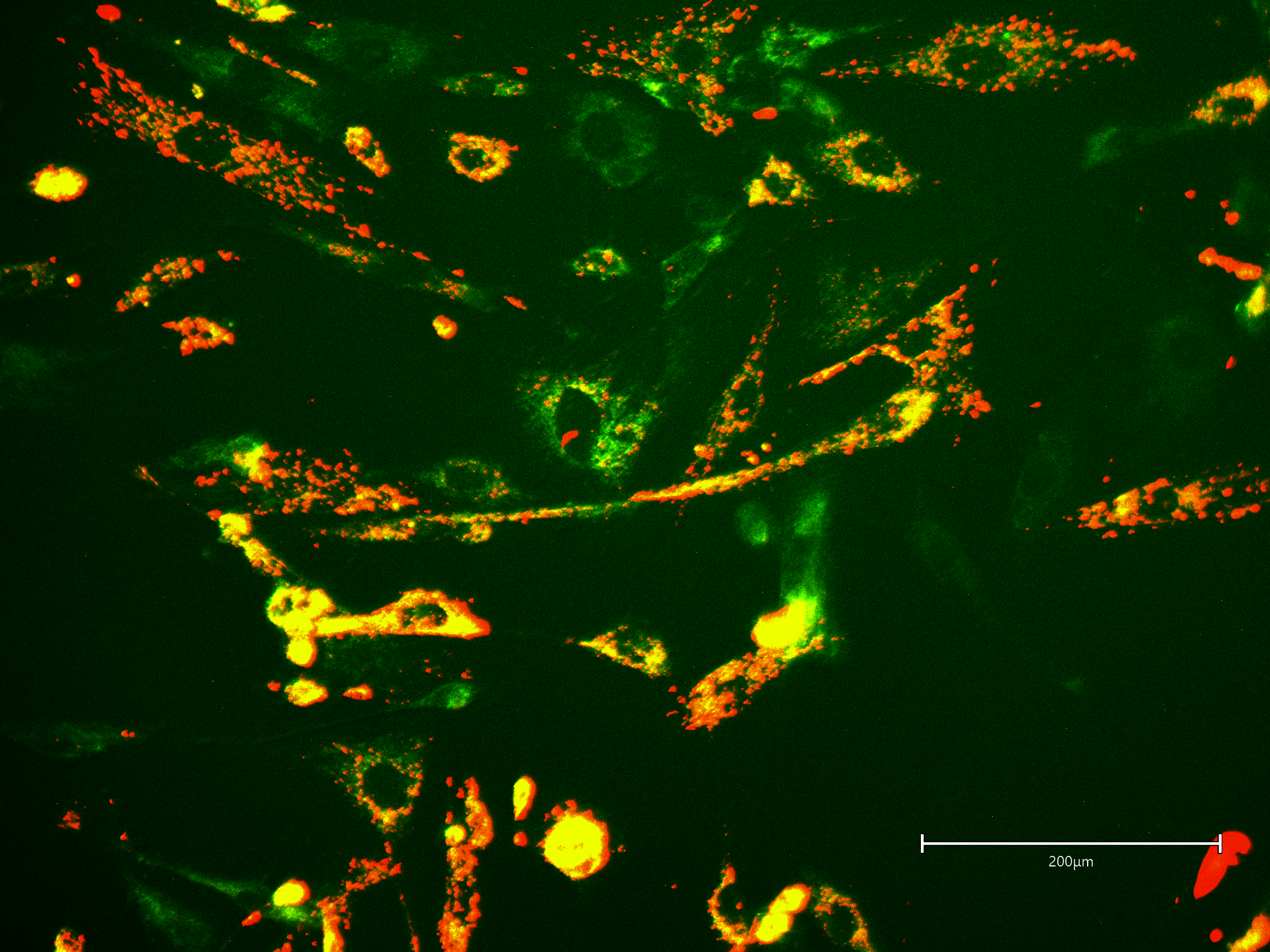

Supplement: Supplementary file 11 — Source data Fig. 4 [file 44321_2025_247_MOESM11_ESM.zip › Figure 4/Figure 4_Panel E/Figure 4_Panel E_Foxk2fl:fl-Myod1-Cre_JC-1.tif]

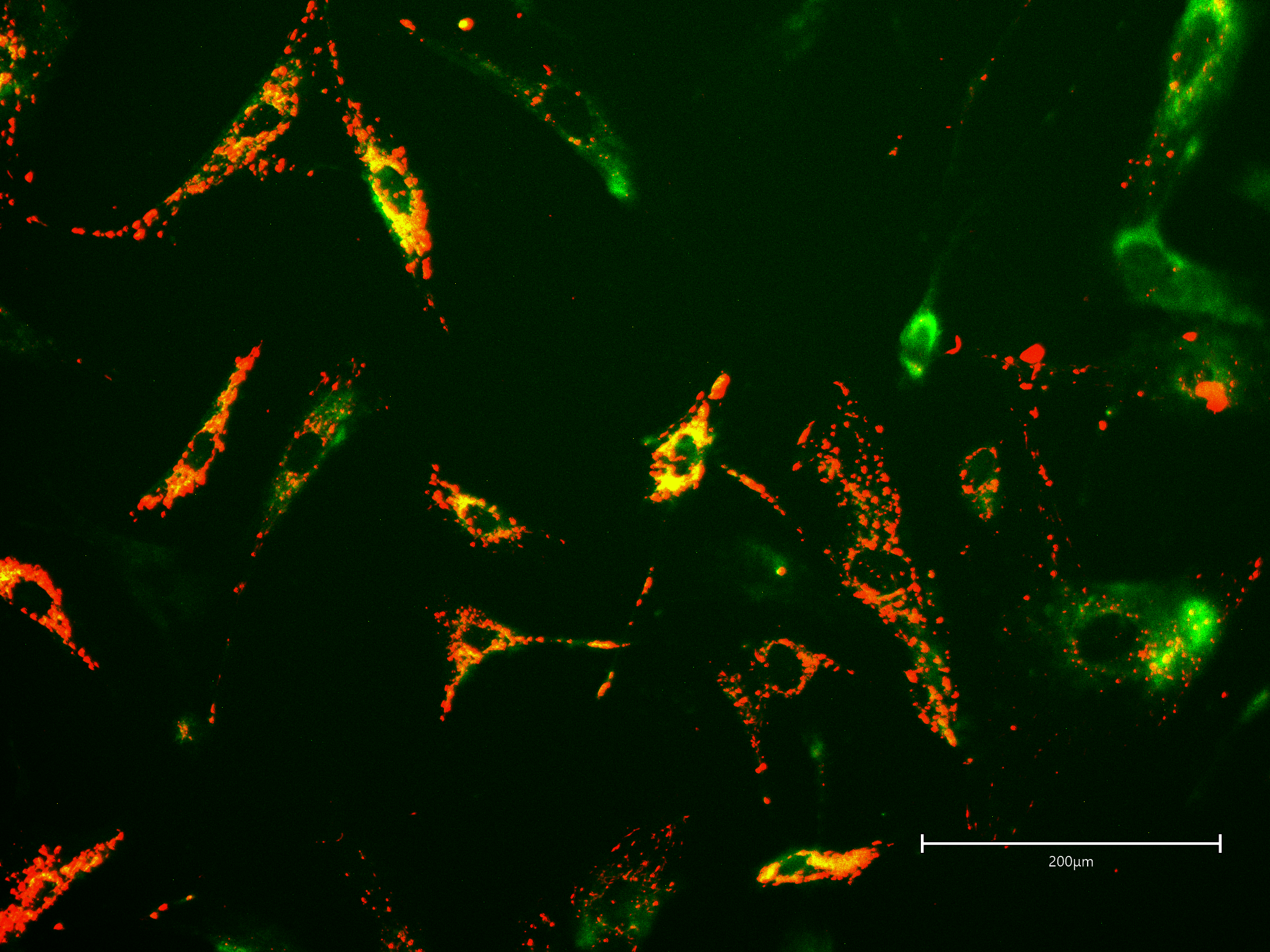

Supplement: Supplementary file 11 — Source data Fig. 4 [file 44321_2025_247_MOESM11_ESM.zip › Figure 4/Figure 4_Panel E/Figure 4_Panel E_Foxk2fl:fl_JC-1.tif]

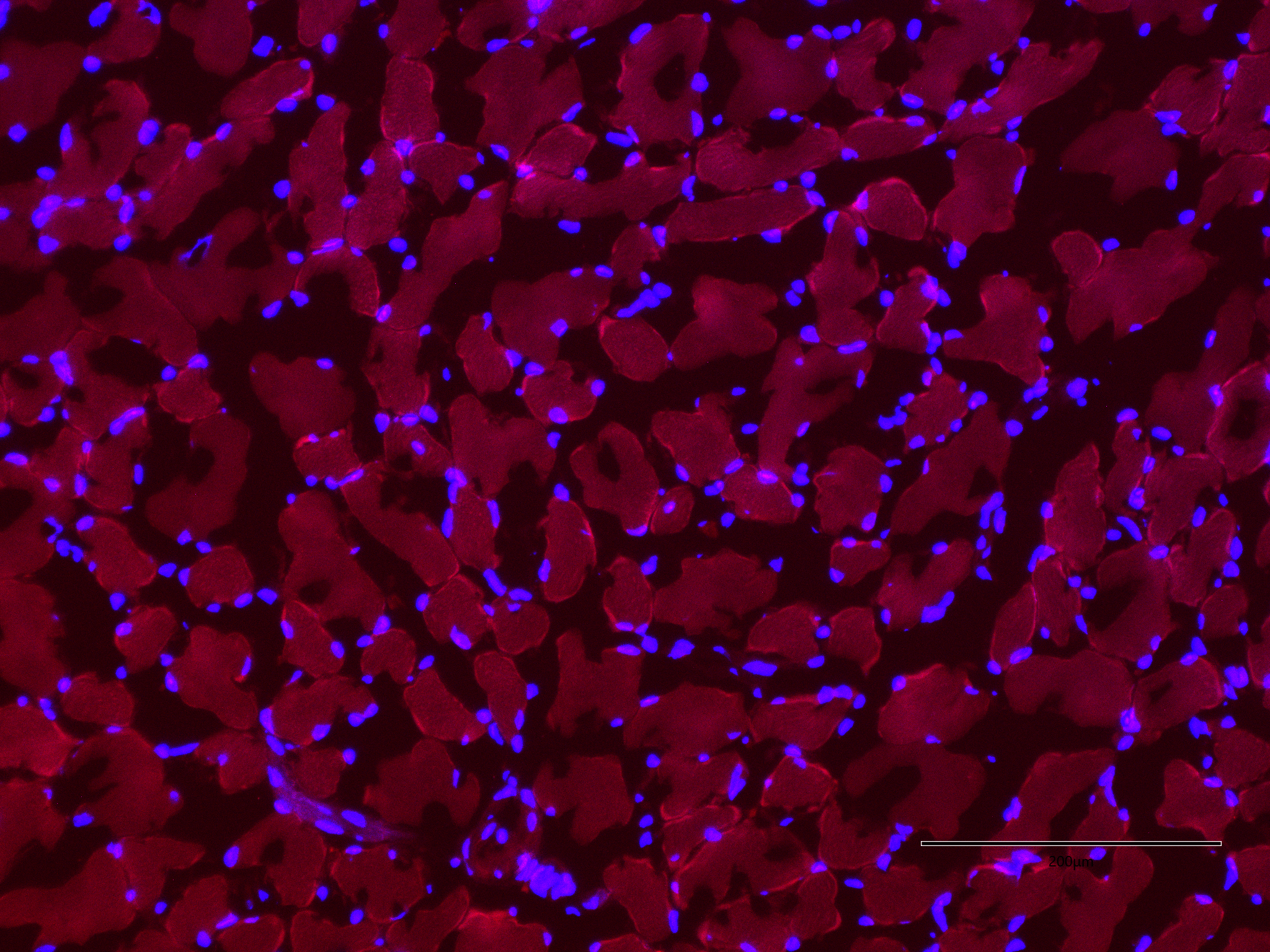

Supplement: Supplementary file 11 — Source data Fig. 4 [file 44321_2025_247_MOESM11_ESM.zip › Figure 4/Figure 4_Panel B/Figure 4_Panel B_ROS_Foxk2fl:fl.tif]

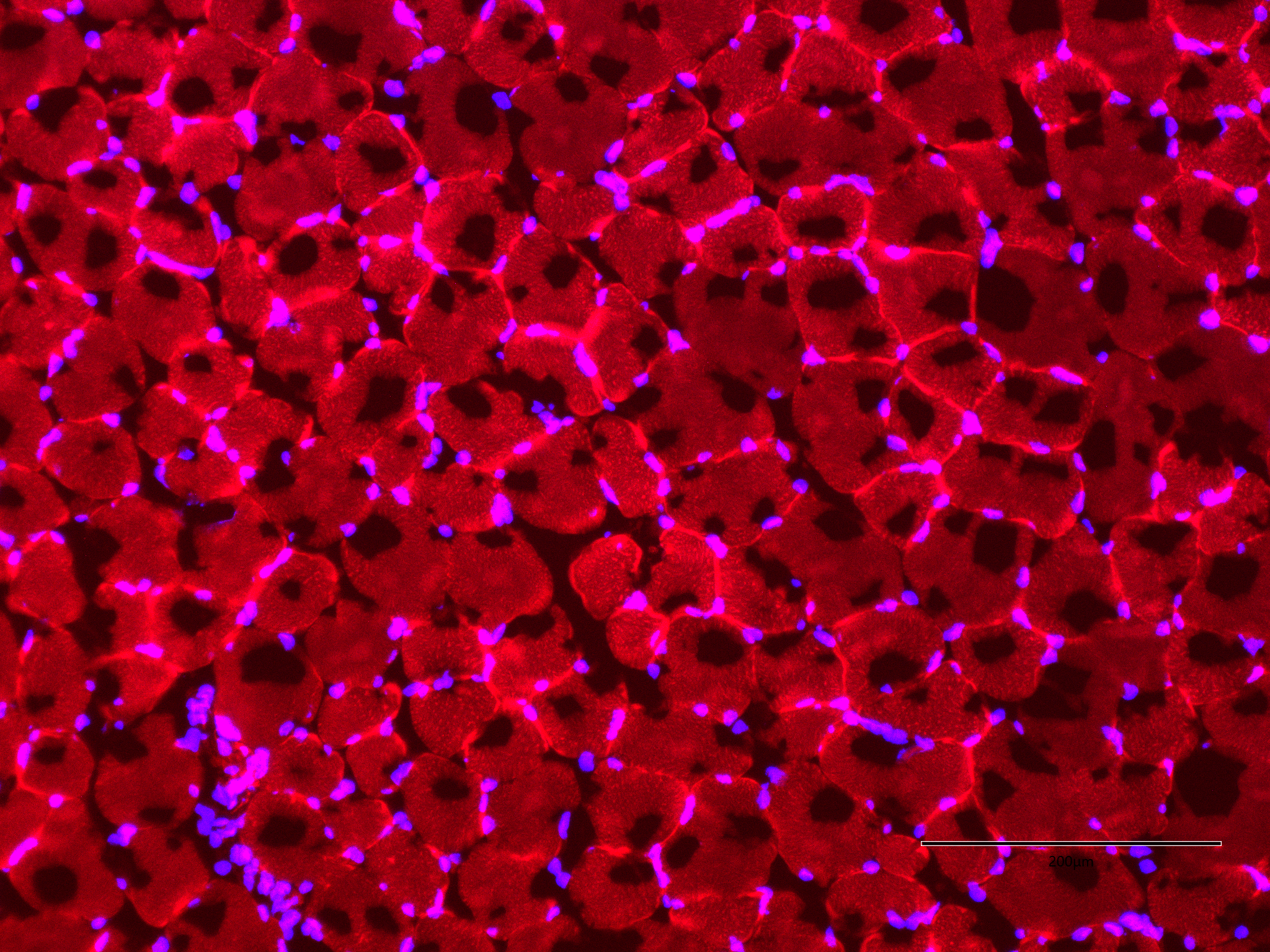

Supplement: Supplementary file 11 — Source data Fig. 4 [file 44321_2025_247_MOESM11_ESM.zip › Figure 4/Figure 4_Panel B/Figure 4_Panel B_ROS_Foxk2fl:fl-Myod1-Cre.tif]

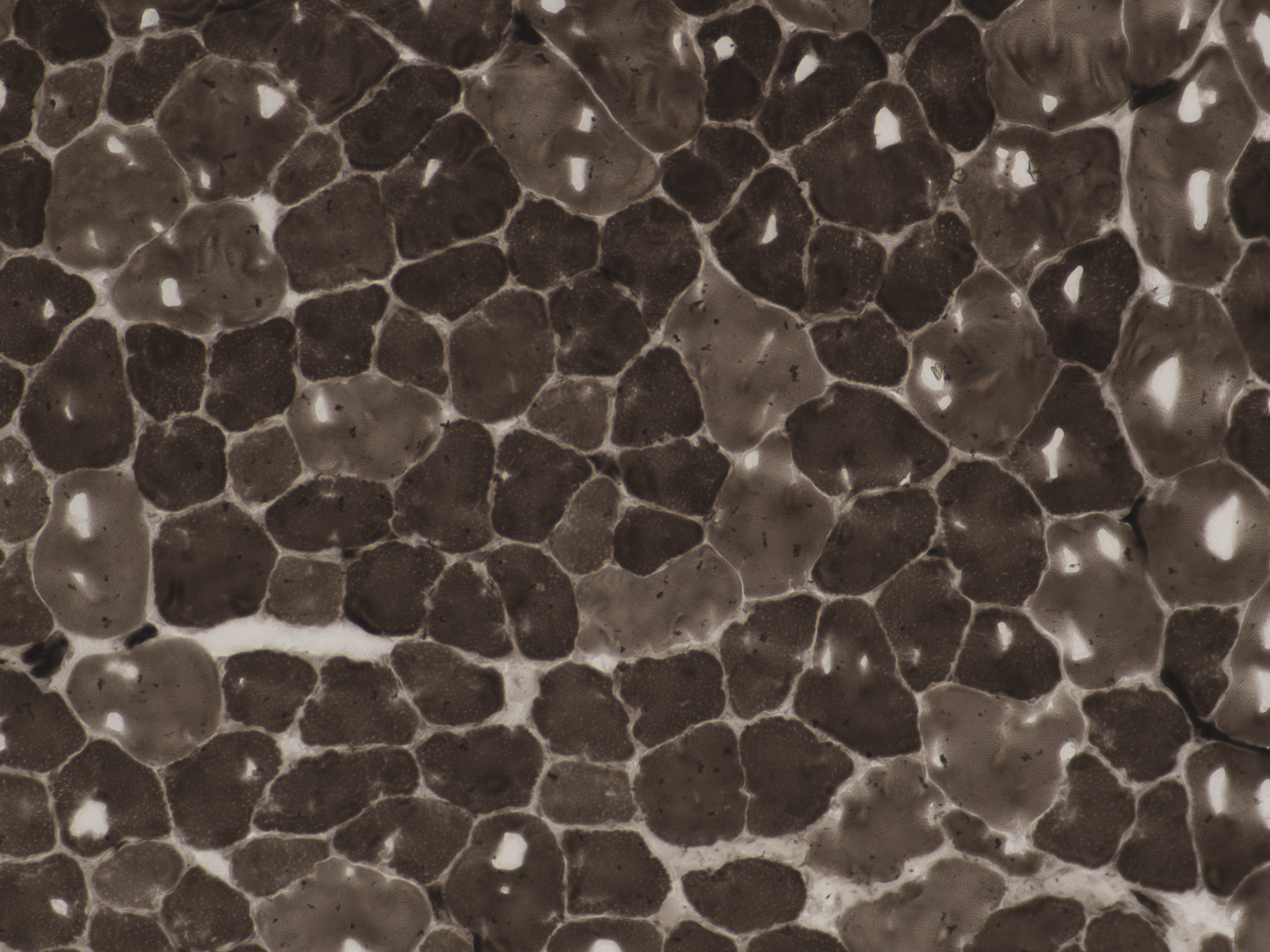

Supplement: Supplementary file 11 — Source data Fig. 4 [file 44321_2025_247_MOESM11_ESM.zip › Figure 4/Figure 4_Panel C/Figure 4_Panel C_ATPase_Foxk2fl:fl.tif]

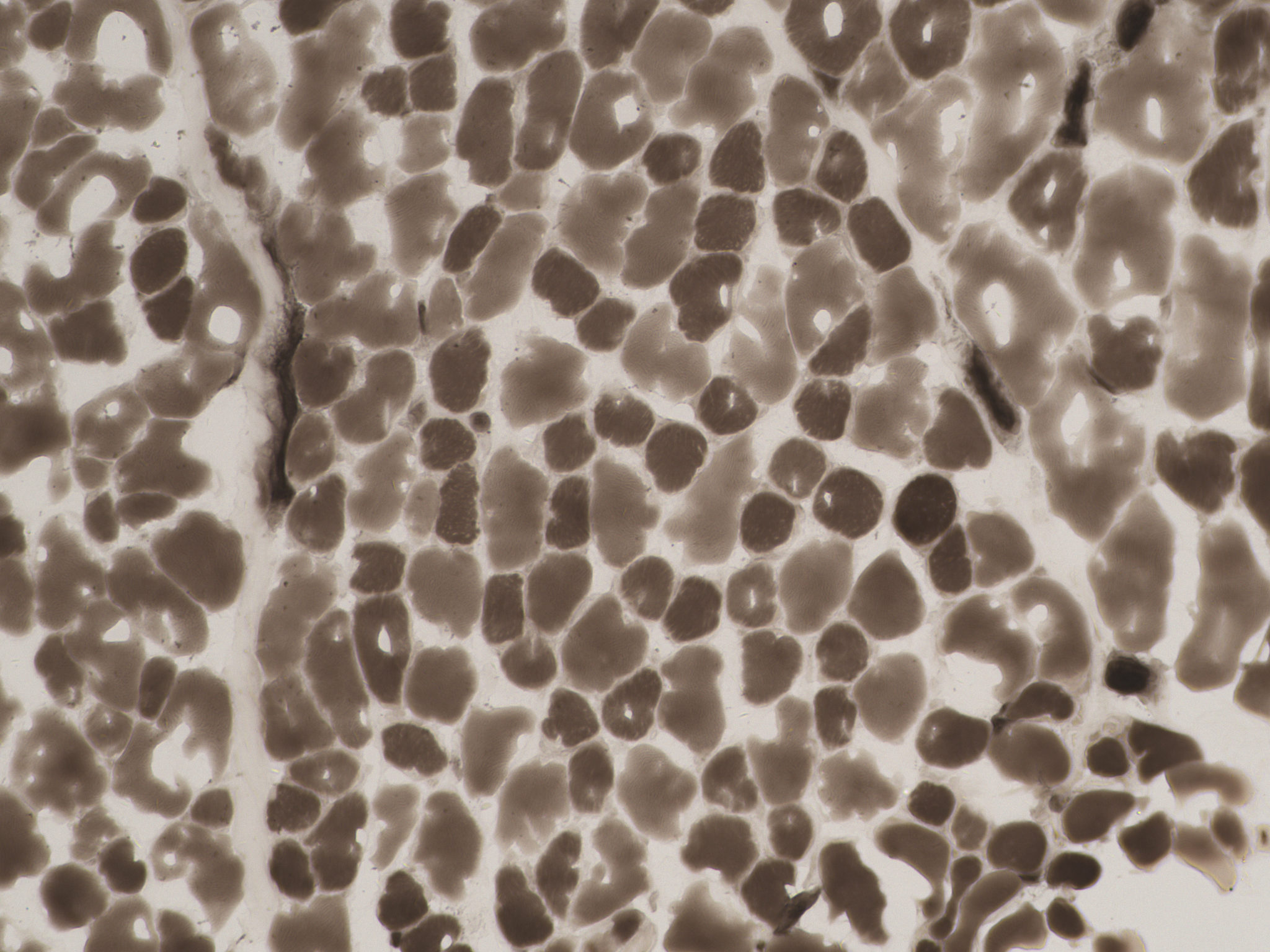

Supplement: Supplementary file 11 — Source data Fig. 4 [file 44321_2025_247_MOESM11_ESM.zip › Figure 4/Figure 4_Panel C/Figure 4_Panel C_ATPase_Foxk2fl:fl-Myod1-Cre.tif]

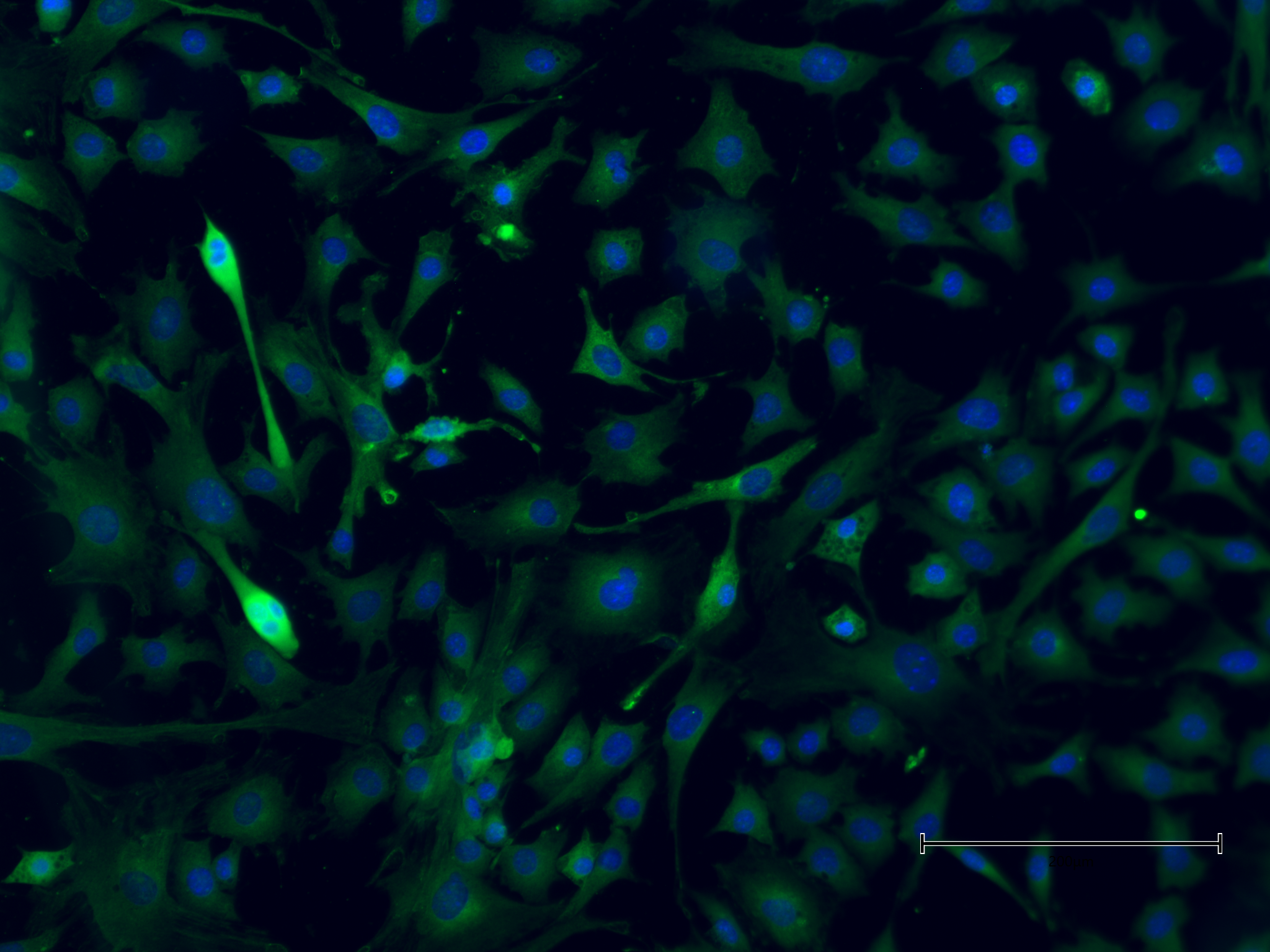

Supplement: Supplementary file 11 — Source data Fig. 4 [file 44321_2025_247_MOESM11_ESM.zip › Figure 4/Figure 4_Panel D/Figure 4_Panel D_Foxk2fl:fl_IF-OPA1.tif]

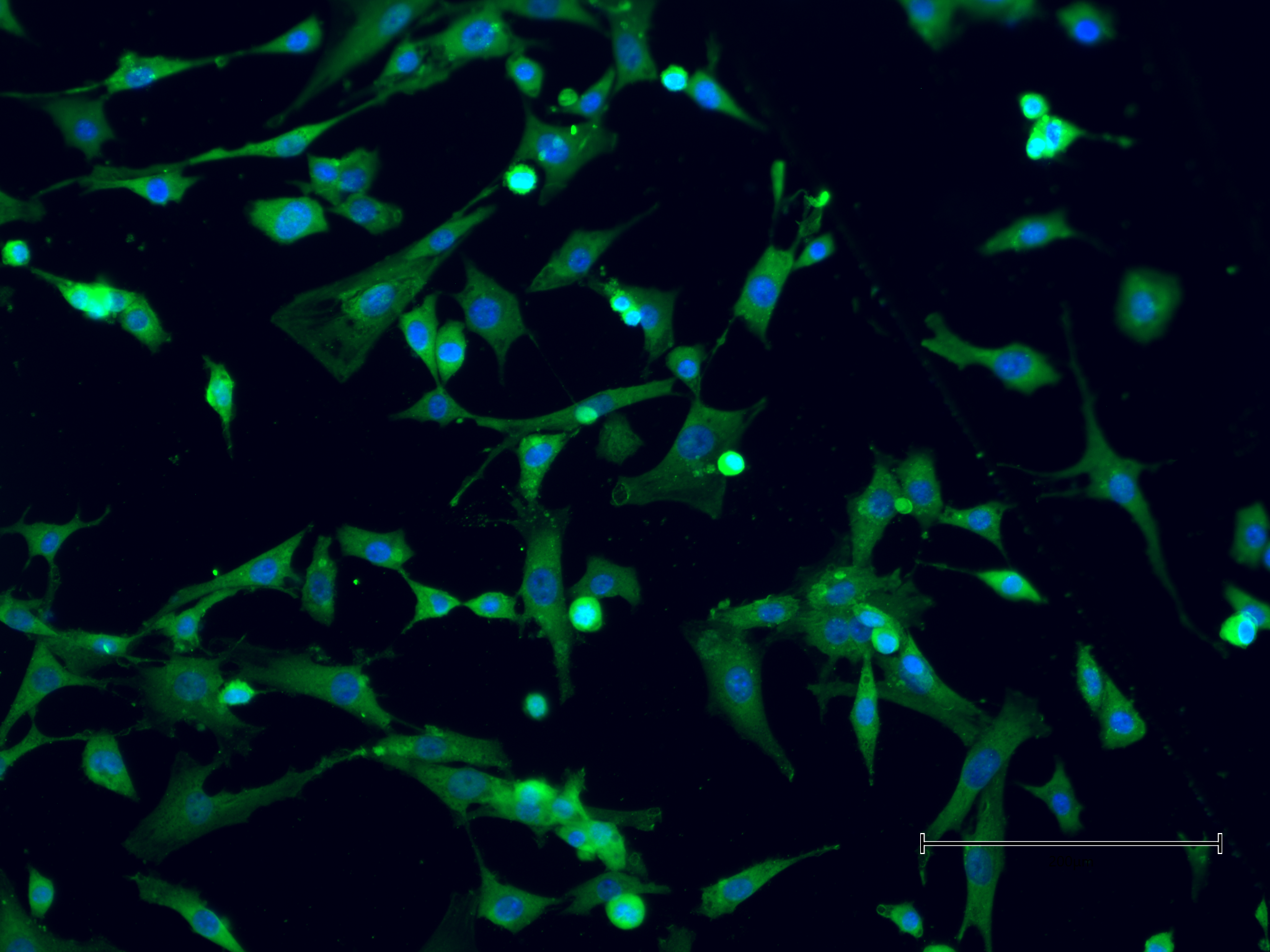

Supplement: Supplementary file 11 — Source data Fig. 4 [file 44321_2025_247_MOESM11_ESM.zip › Figure 4/Figure 4_Panel D/Figure 4_Panel D_Foxk2fl:fl-Myod1-Cre_IF-DRP1.tif]

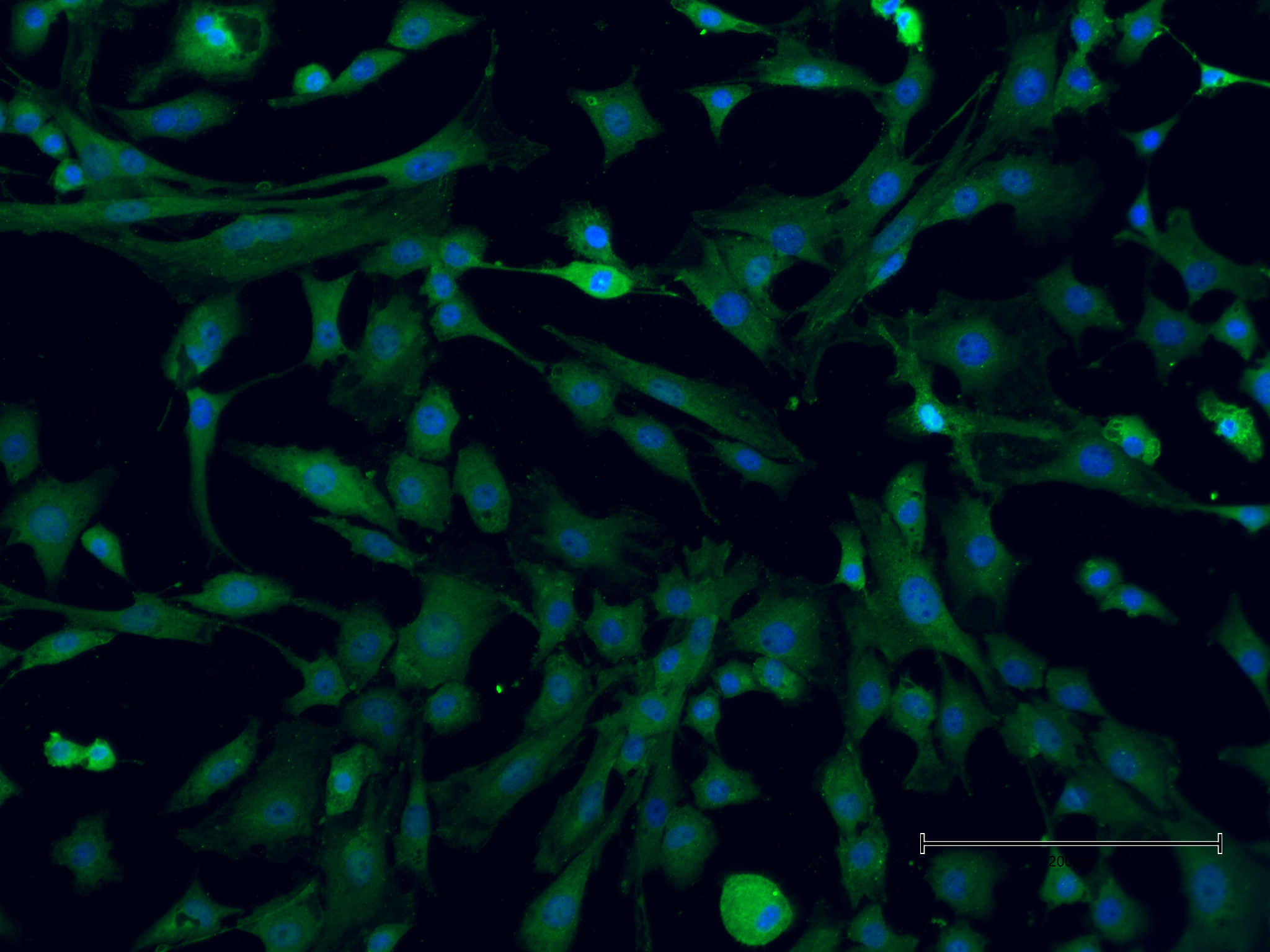

Supplement: Supplementary file 11 — Source data Fig. 4 [file 44321_2025_247_MOESM11_ESM.zip › Figure 4/Figure 4_Panel D/Figure 4_Panel D_Foxk2fl:fl_IF-DRP1.tif]

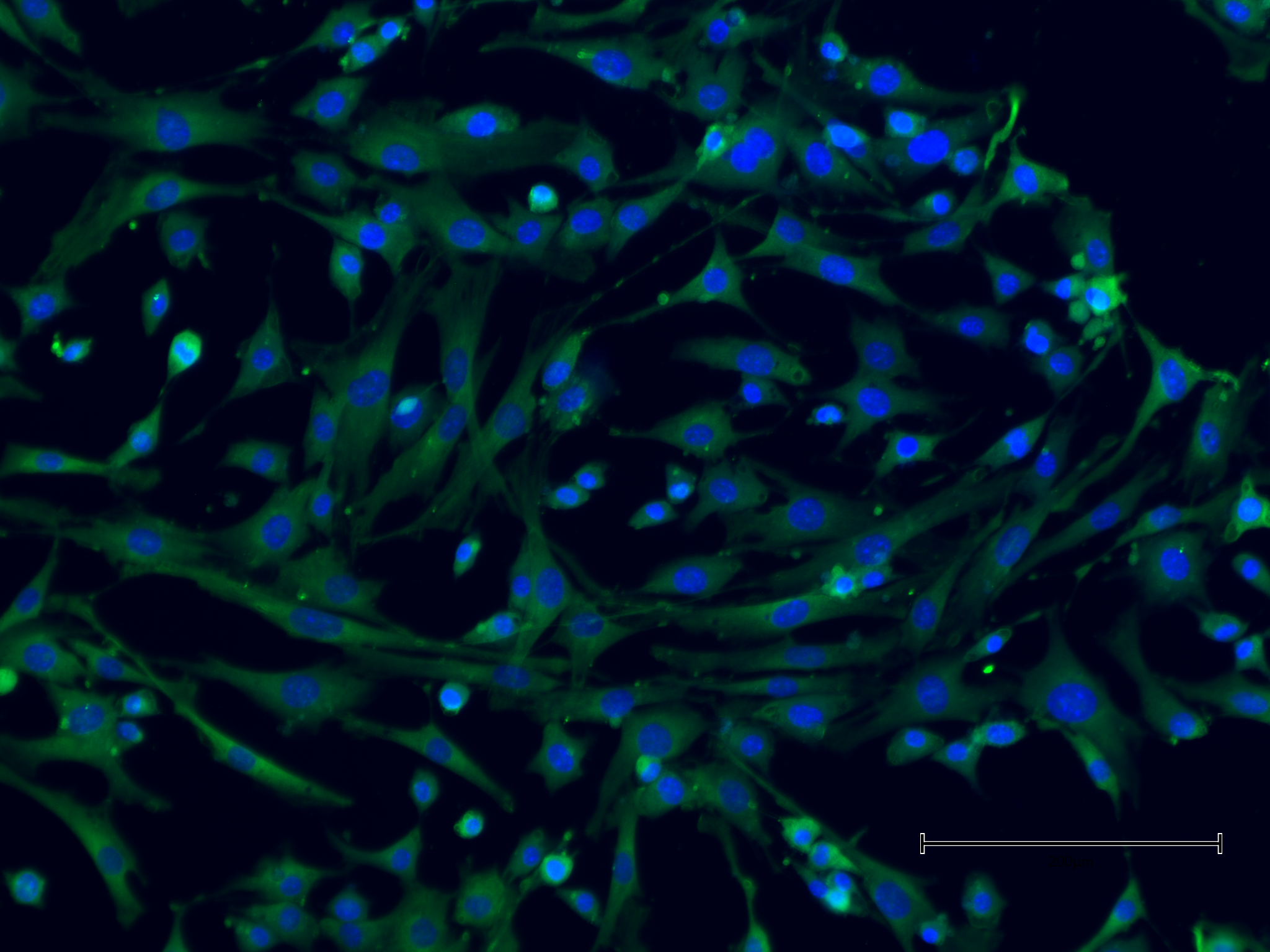

Supplement: Supplementary file 11 — Source data Fig. 4 [file 44321_2025_247_MOESM11_ESM.zip › Figure 4/Figure 4_Panel D/Figure 4_Panel D_Foxk2fl:fl-Myod1-Cre_IF-OPA1.tif]

Western blot image in Figure 5A:


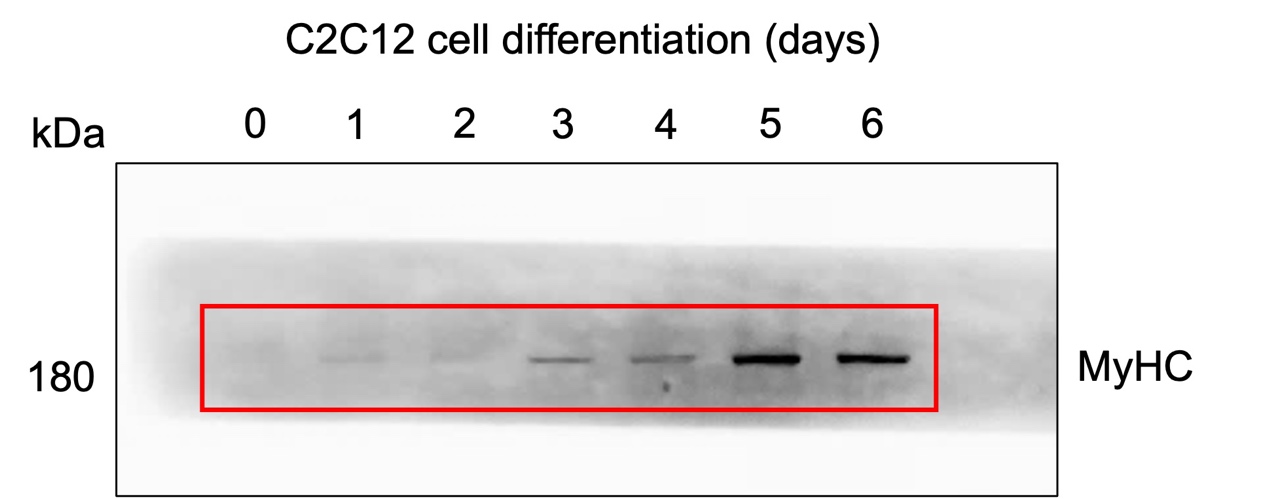


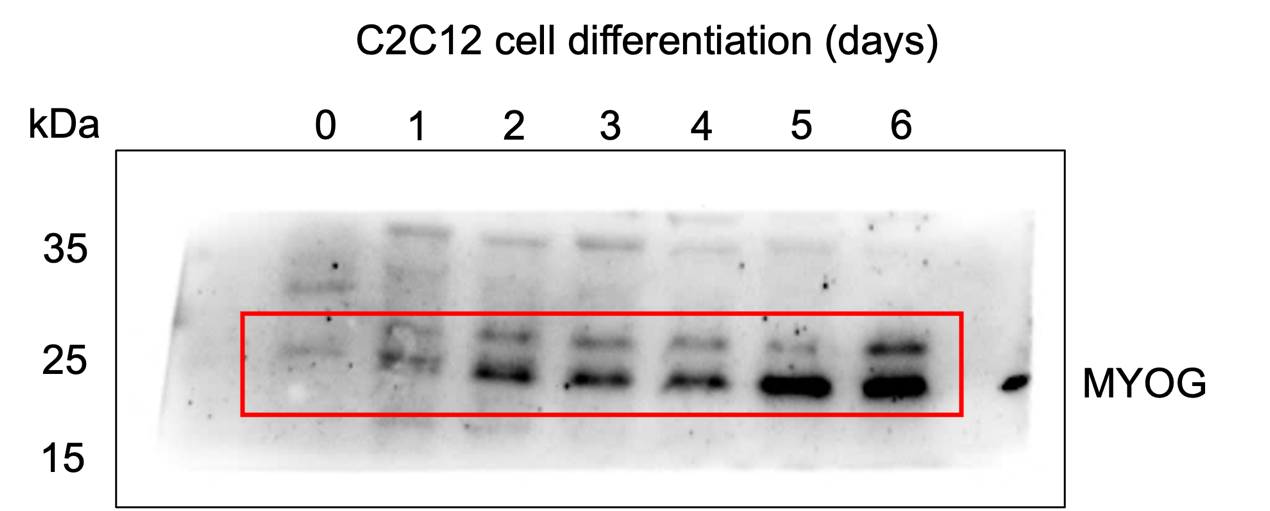


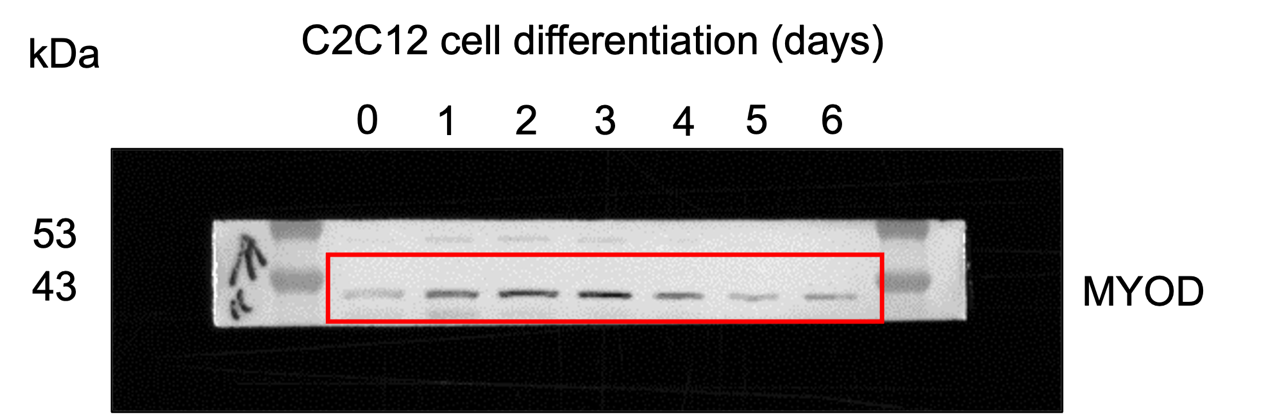


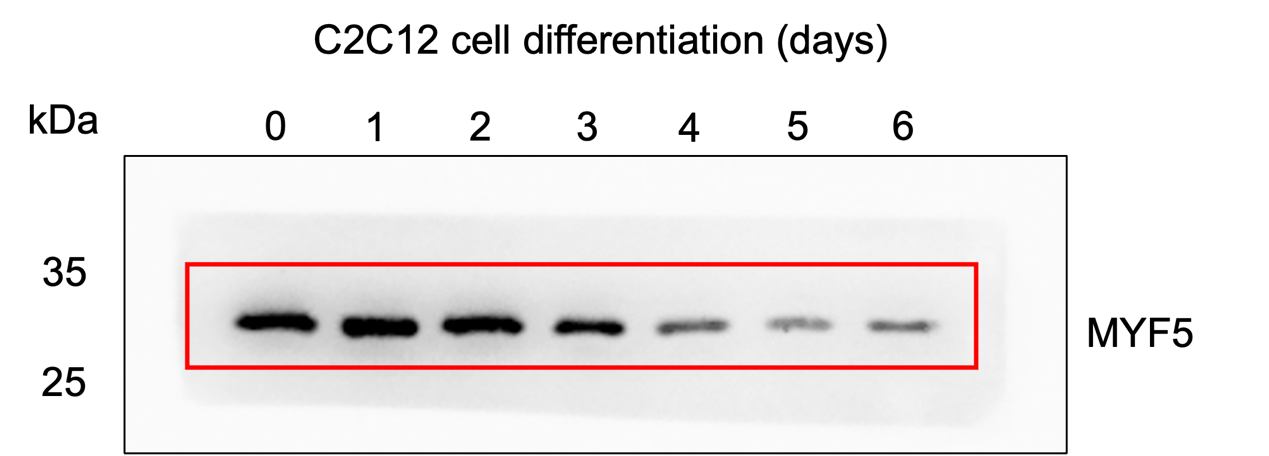


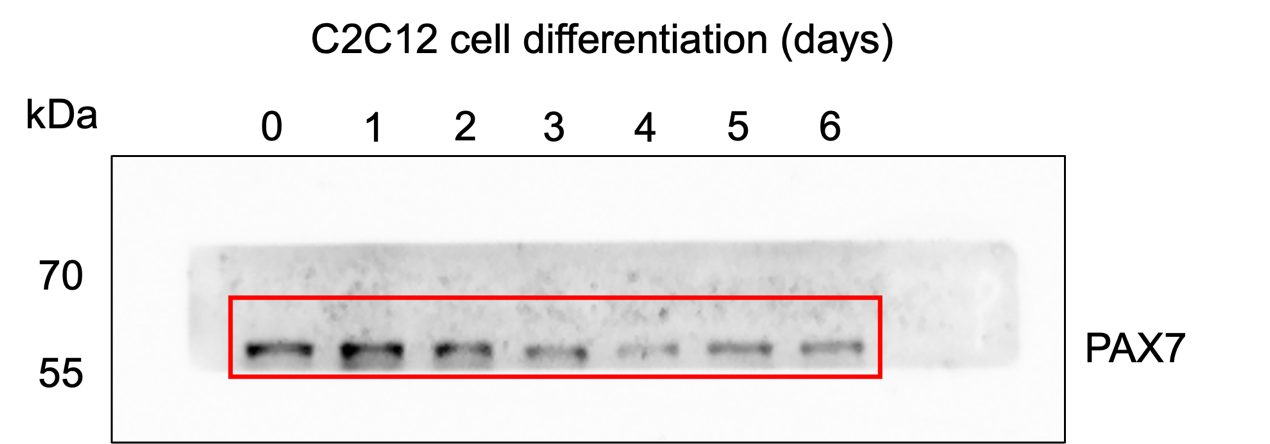


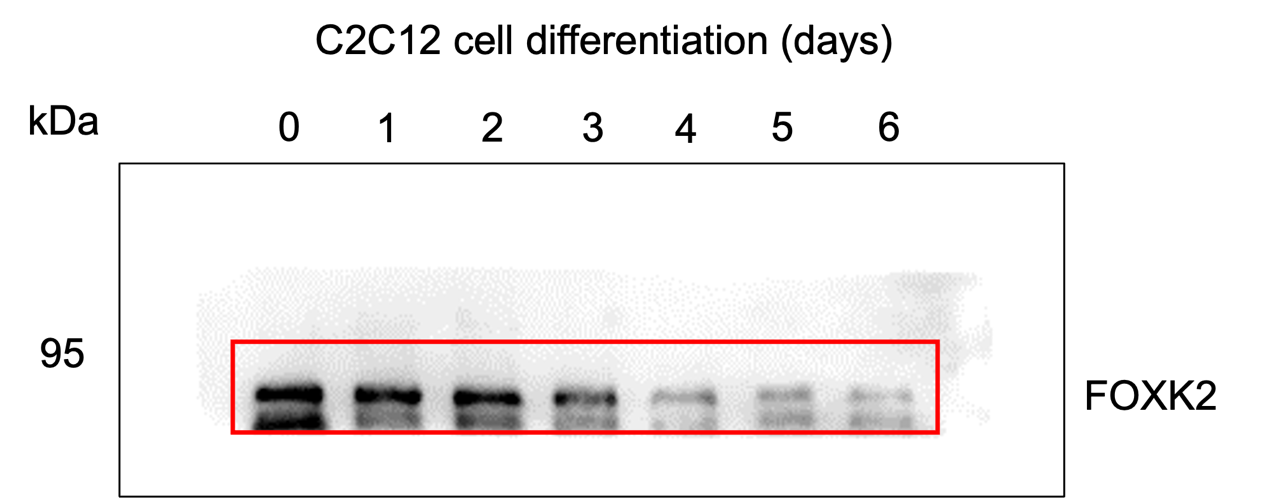


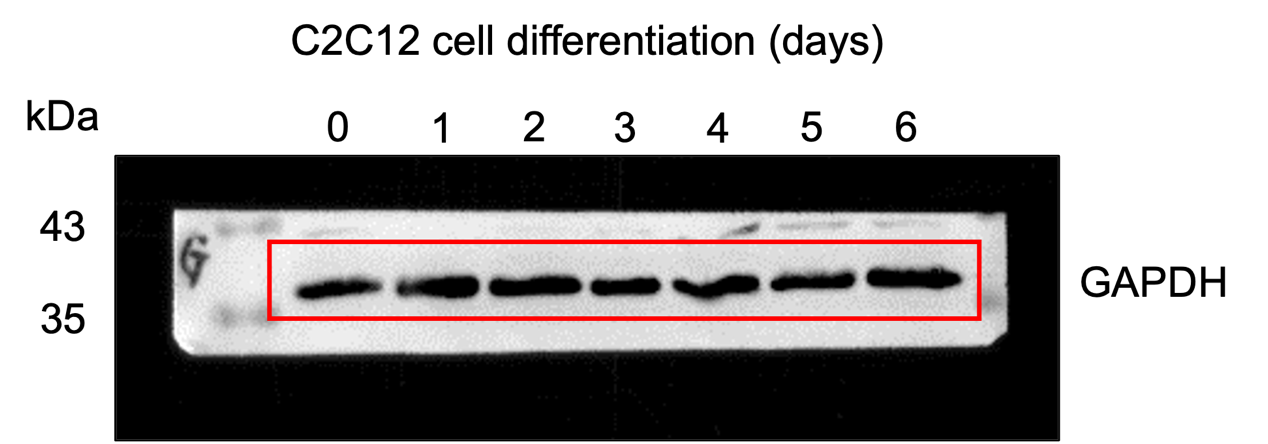

Supplement: Supplementary file 12 — Source data Fig. 5 [file 44321_2025_247_MOESM12_ESM.zip › Figure 5/Figure 5_Panel A/Read me.docx]

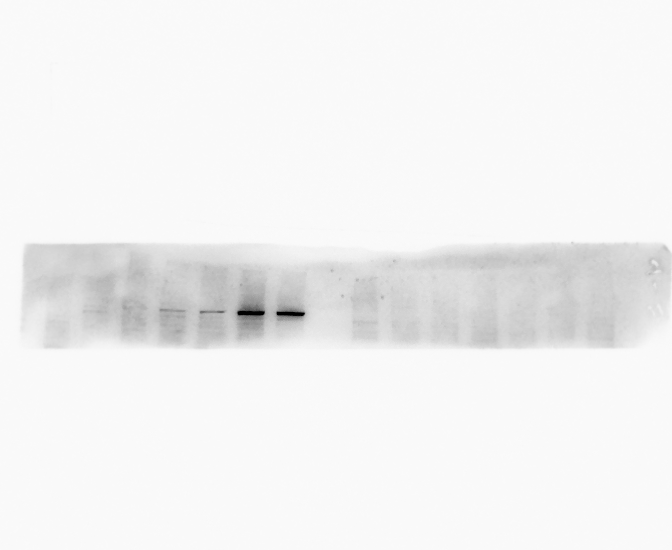

Supplement: Supplementary file 12 — Source data Fig. 5 [file 44321_2025_247_MOESM12_ESM.zip › Figure 5/Figure 5_Panel A/Figure 5_Panel A_WB-MyHC.tif]

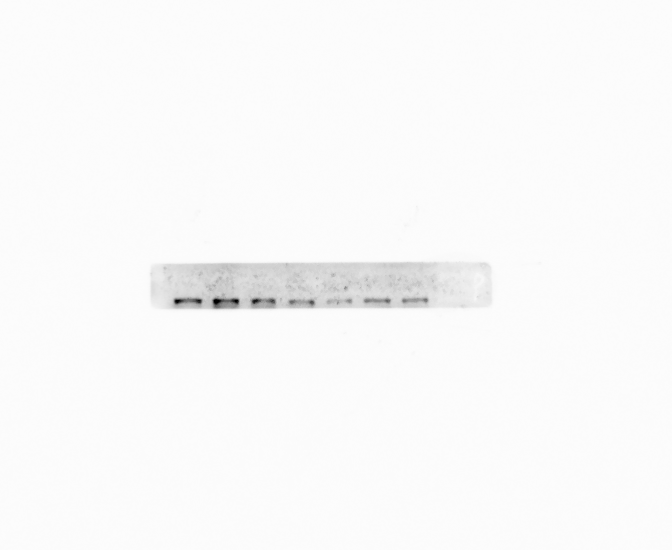

Supplement: Supplementary file 12 — Source data Fig. 5 [file 44321_2025_247_MOESM12_ESM.zip › Figure 5/Figure 5_Panel A/Figure 5_Panel A_WB-PAX7.tif]

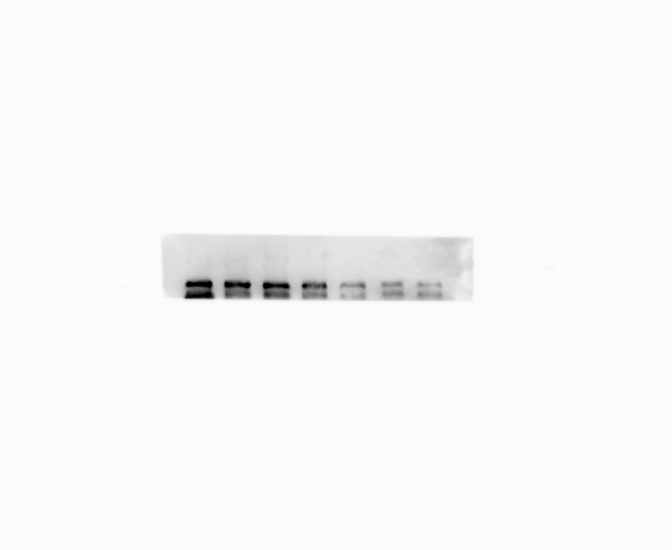

Supplement: Supplementary file 12 — Source data Fig. 5 [file 44321_2025_247_MOESM12_ESM.zip › Figure 5/Figure 5_Panel A/Figure 5_Panel A_WB-FOXK2.tif]

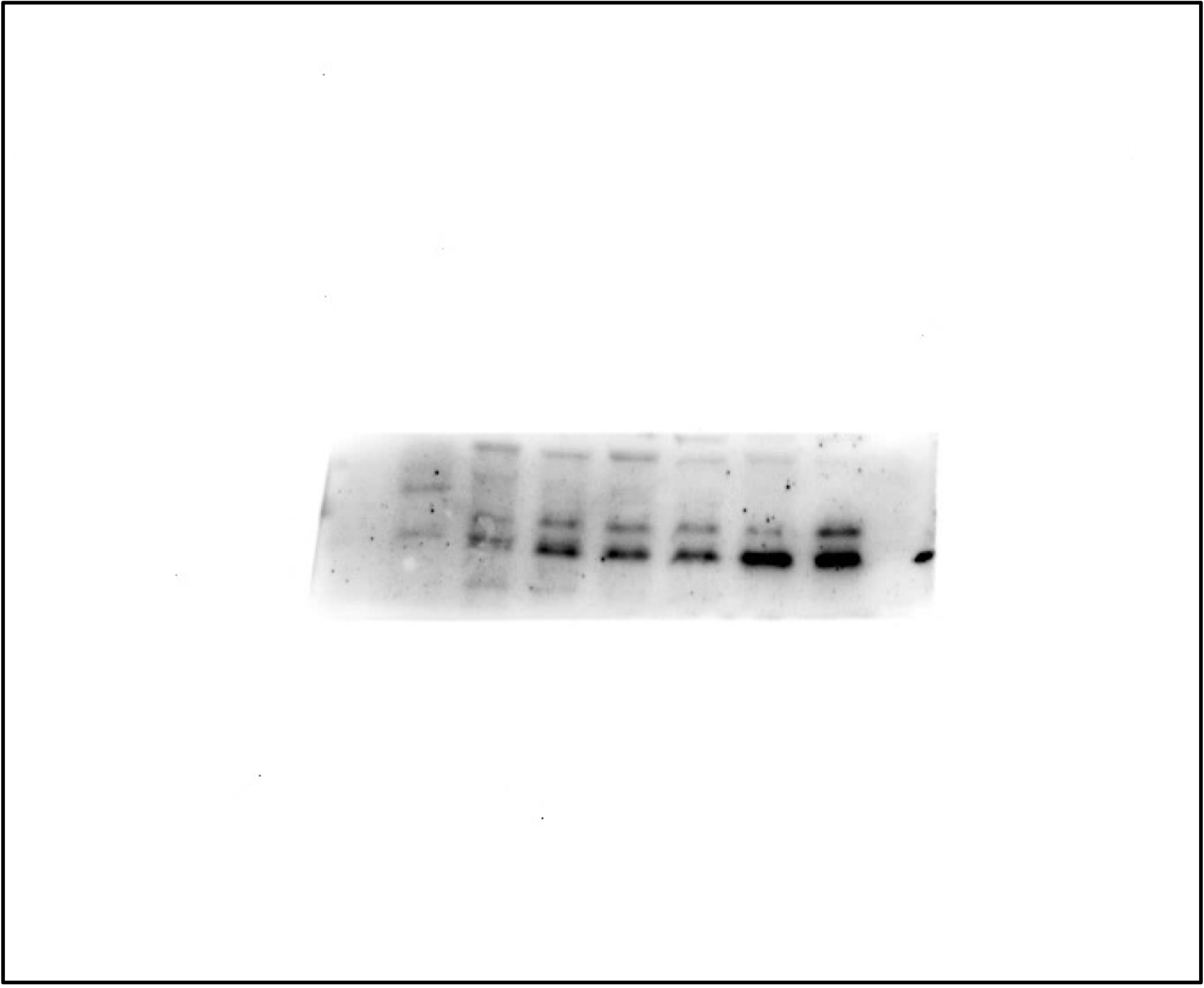

Supplement: Supplementary file 12 — Source data Fig. 5 [file 44321_2025_247_MOESM12_ESM.zip › Figure 5/Figure 5_Panel A/Figure 5_Panel A_WB-MYOG.tif]

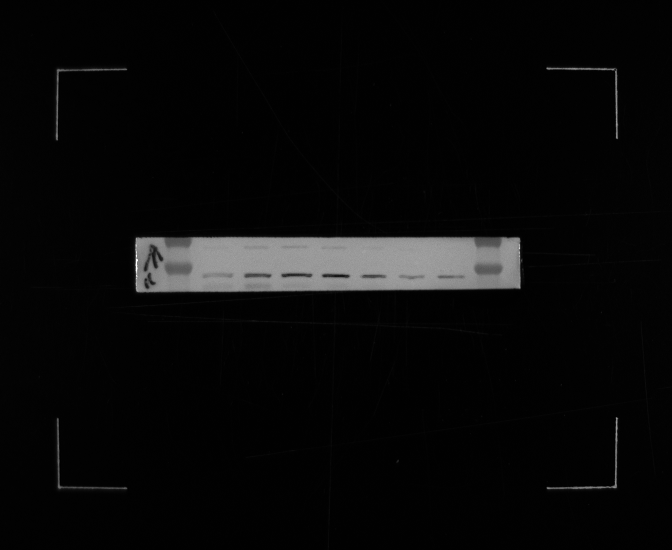

Supplement: Supplementary file 12 — Source data Fig. 5 [file 44321_2025_247_MOESM12_ESM.zip › Figure 5/Figure 5_Panel A/Figure 5_Panel A_WB-MYOD.tif]

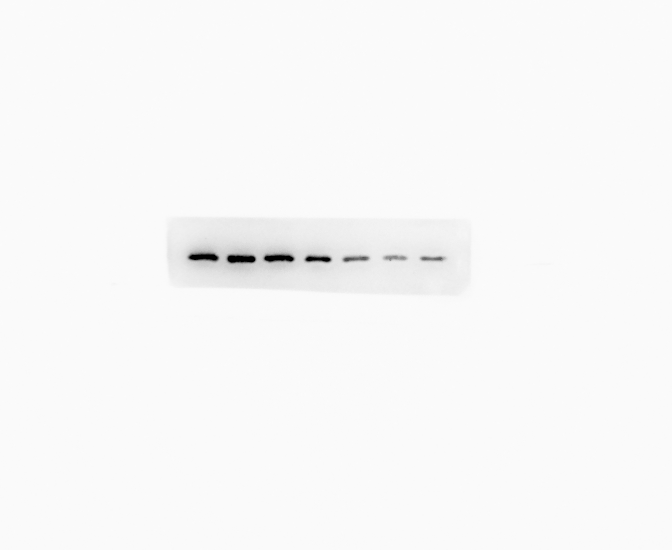

Supplement: Supplementary file 12 — Source data Fig. 5 [file 44321_2025_247_MOESM12_ESM.zip › Figure 5/Figure 5_Panel A/Figure 5_Panel A_WB-MYF5.tif]

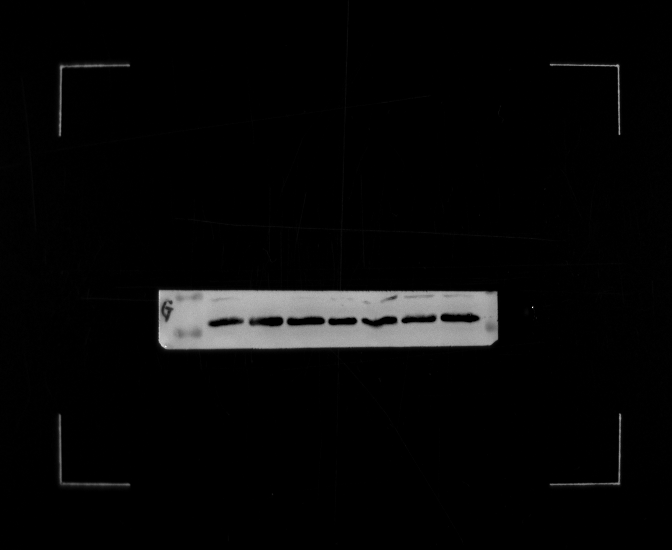

Supplement: Supplementary file 12 — Source data Fig. 5 [file 44321_2025_247_MOESM12_ESM.zip › Figure 5/Figure 5_Panel A/Figure 5_Panel A_WB-GAPDH.tif]

Western blot image in Figure 5C:


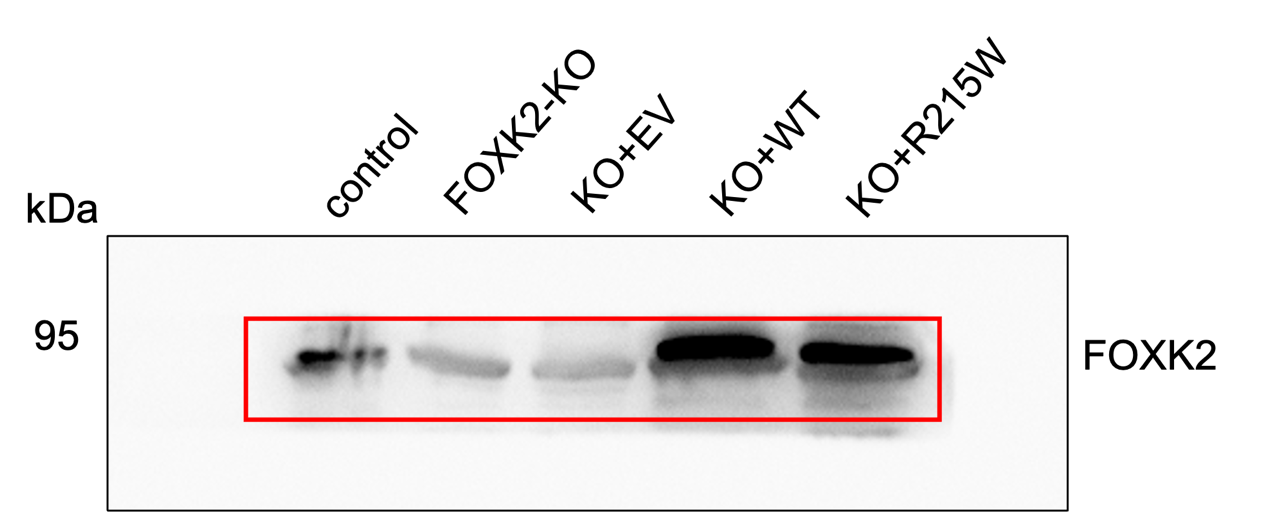


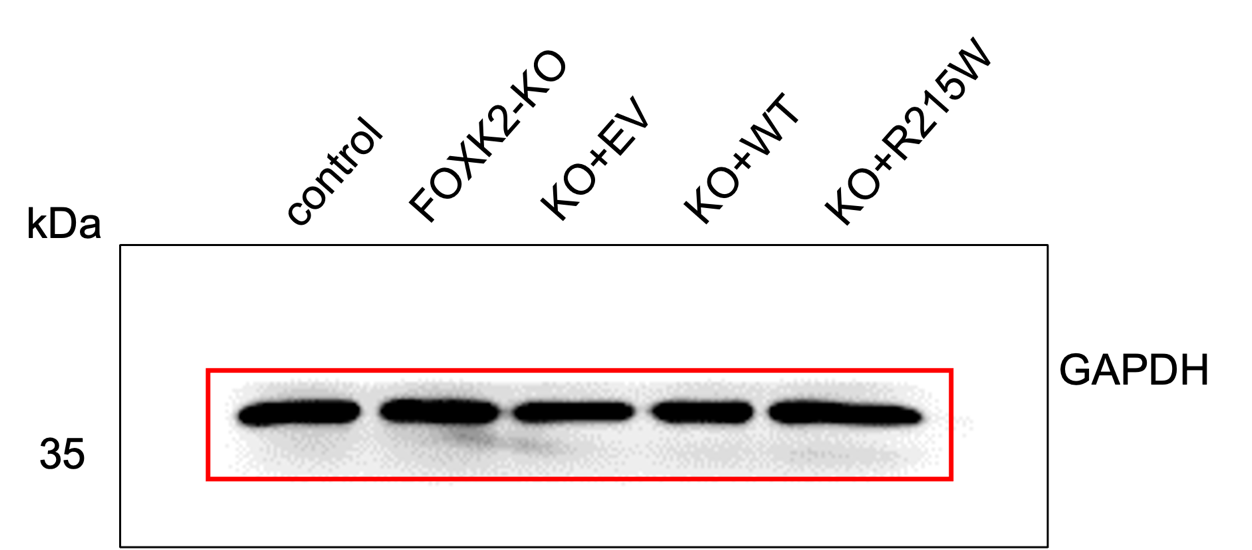

Supplement: Supplementary file 12 — Source data Fig. 5 [file 44321_2025_247_MOESM12_ESM.zip › Figure 5/Figure 5_Panel C/Read me.docx]

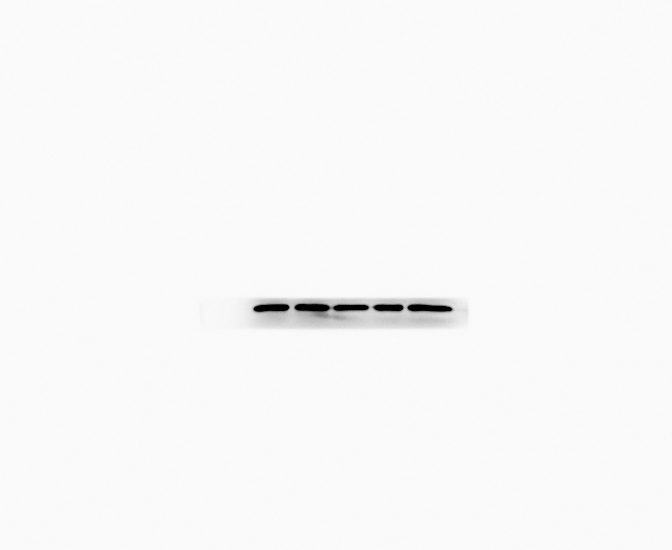

Supplement: Supplementary file 12 — Source data Fig. 5 [file 44321_2025_247_MOESM12_ESM.zip › Figure 5/Figure 5_Panel C/Figure 5_Panel C_WB-GAPDH.tif]

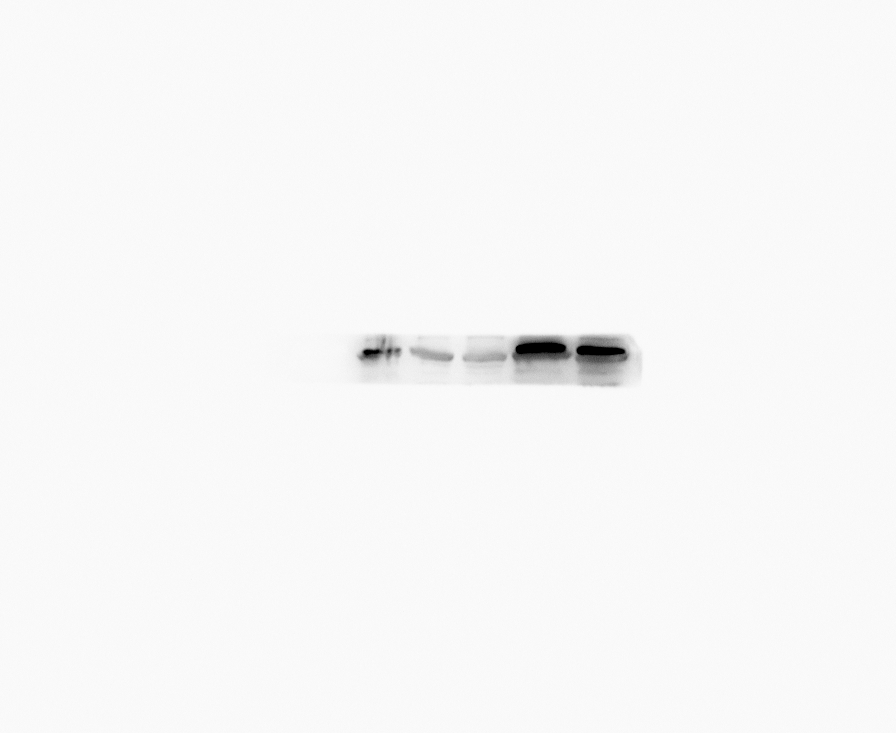

Supplement: Supplementary file 12 — Source data Fig. 5 [file 44321_2025_247_MOESM12_ESM.zip › Figure 5/Figure 5_Panel C/Figure 5_Panel C_WB-FOXK2.tif]

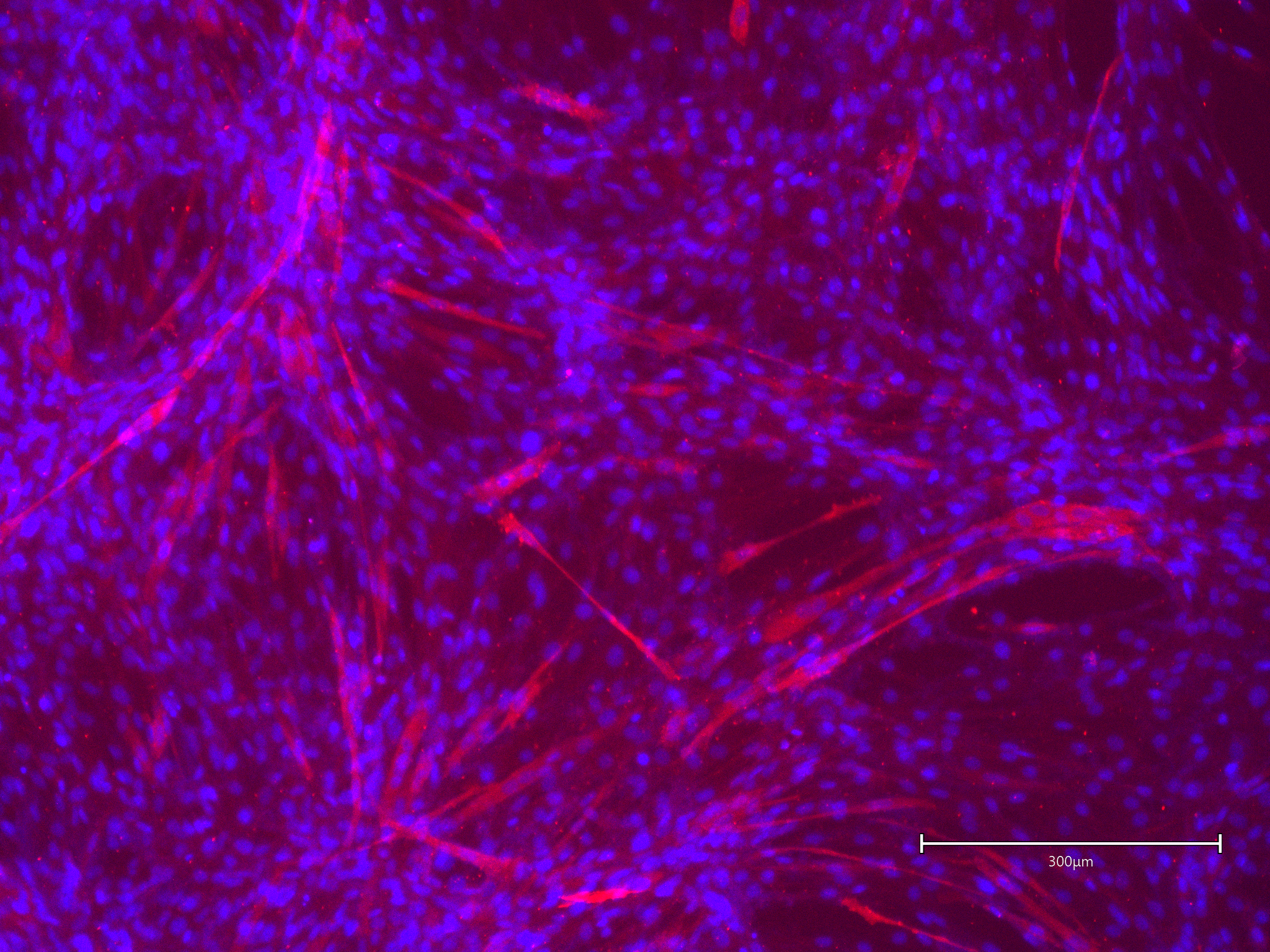

Supplement: Supplementary file 12 — Source data Fig. 5 [file 44321_2025_247_MOESM12_ESM.zip › Figure 5/Figure 5_Panel E/Figure 5_Panel E_KO+WT_IF-merge.tif]

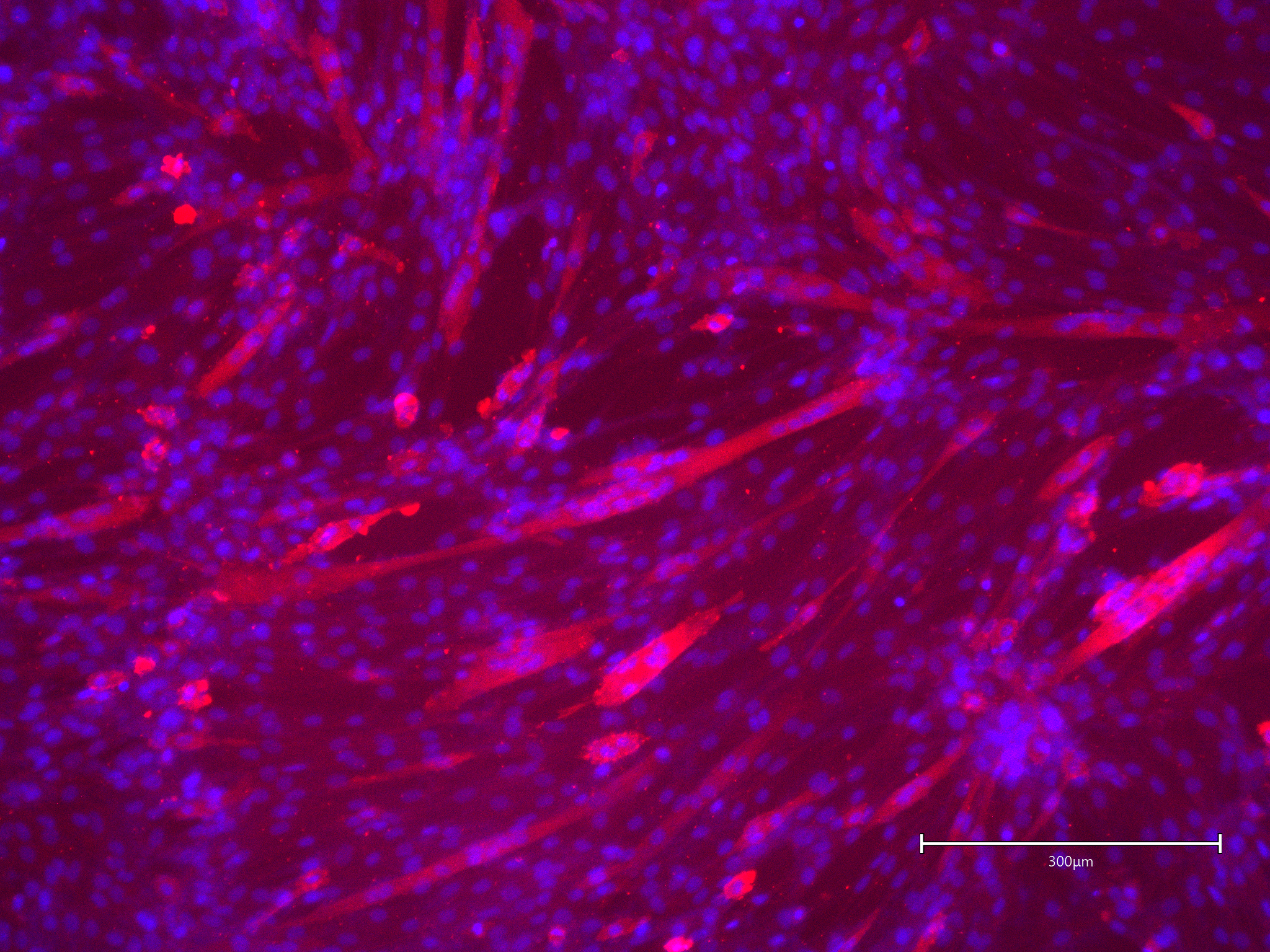

Supplement: Supplementary file 12 — Source data Fig. 5 [file 44321_2025_247_MOESM12_ESM.zip › Figure 5/Figure 5_Panel E/Figure 5_Panel E_control_IF-merge.tif]

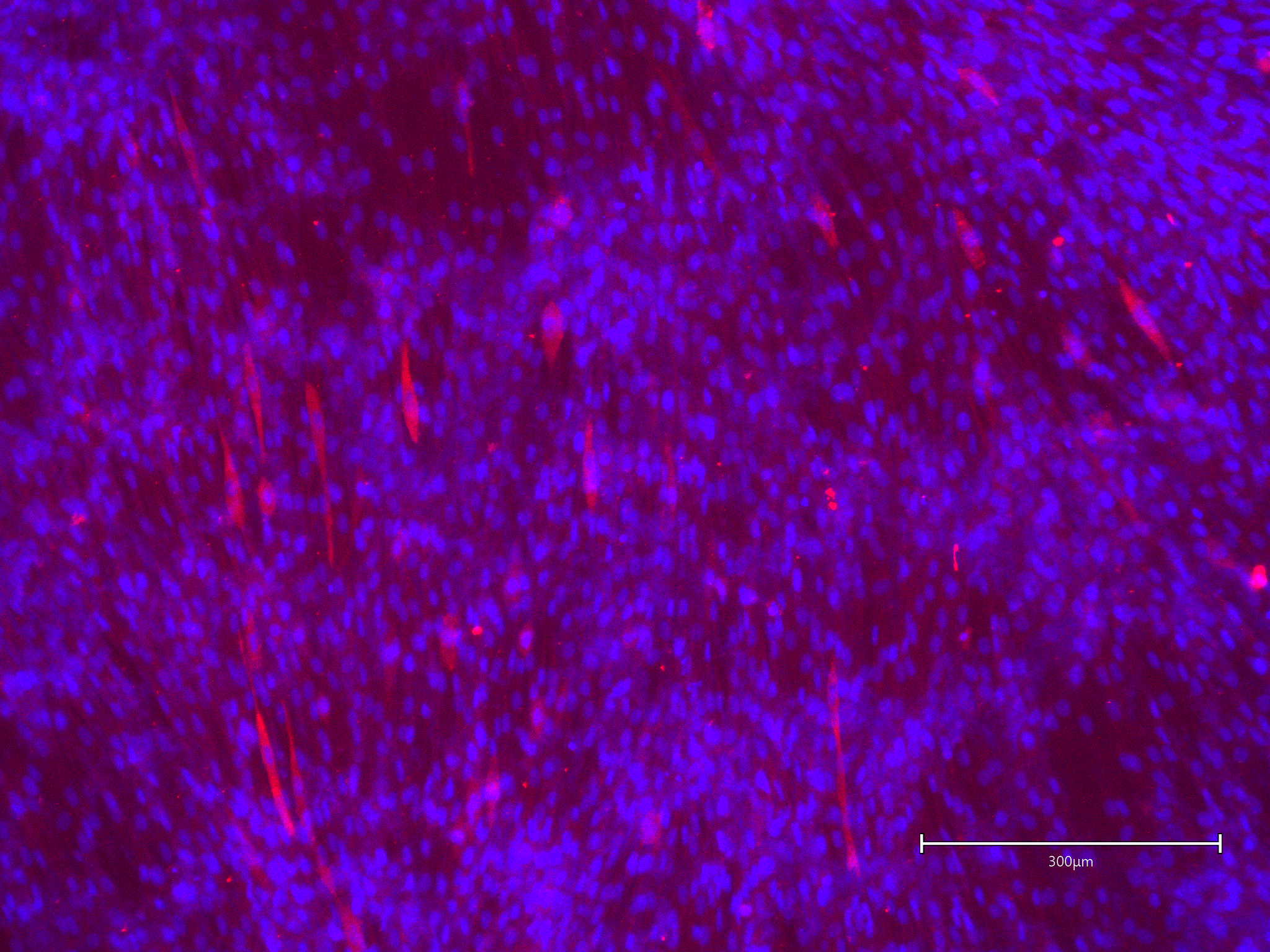

Supplement: Supplementary file 12 — Source data Fig. 5 [file 44321_2025_247_MOESM12_ESM.zip › Figure 5/Figure 5_Panel E/Figure 5_Panel E_KO+EV_IF-merge.tif]

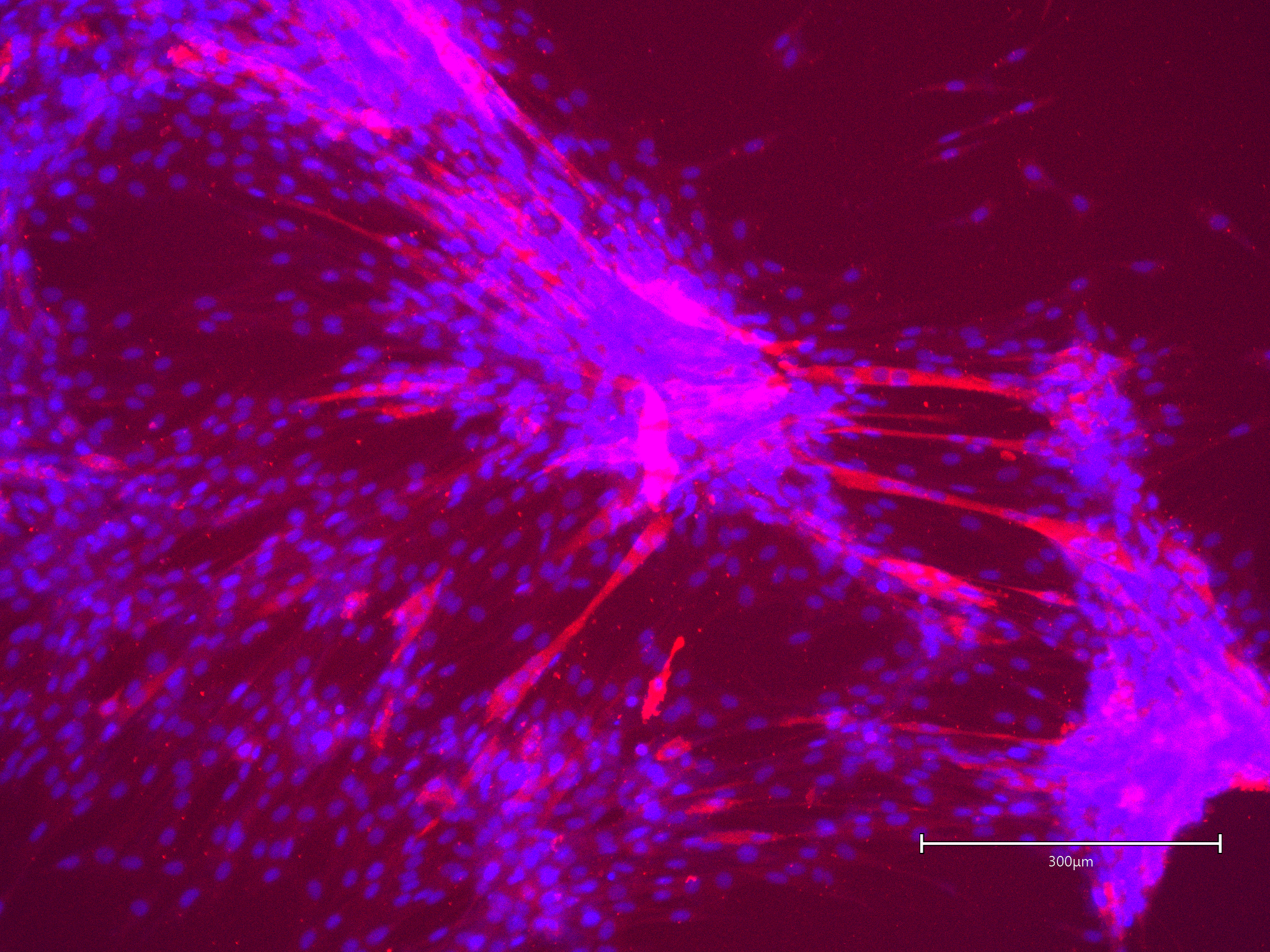

Supplement: Supplementary file 12 — Source data Fig. 5 [file 44321_2025_247_MOESM12_ESM.zip › Figure 5/Figure 5_Panel E/Figure 5_Panel E_blank_IF-merge.tif]

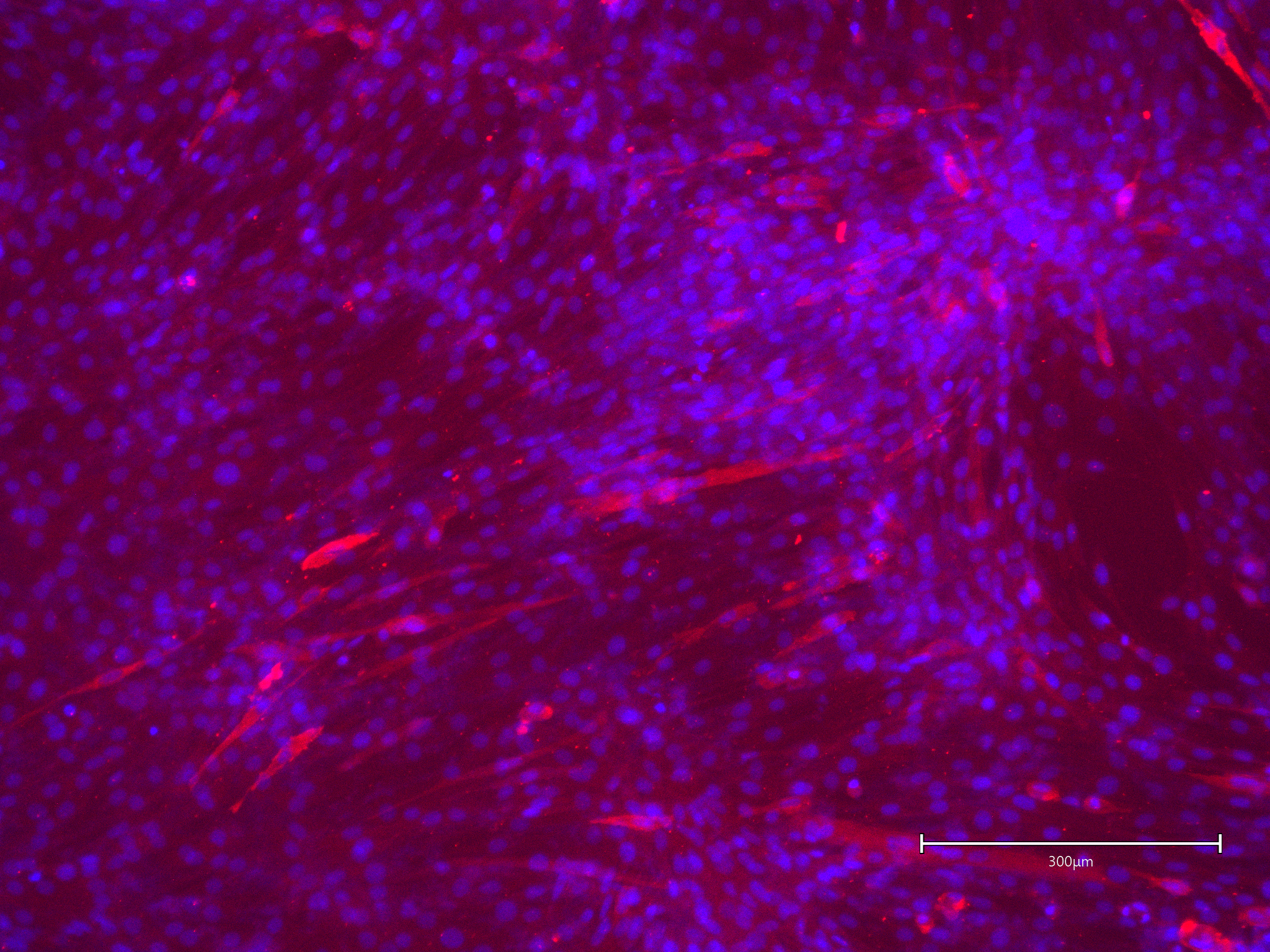

Supplement: Supplementary file 12 — Source data Fig. 5 [file 44321_2025_247_MOESM12_ESM.zip › Figure 5/Figure 5_Panel E/Figure 5_Panel E_FOXK2-KO_IF-merge.tif]

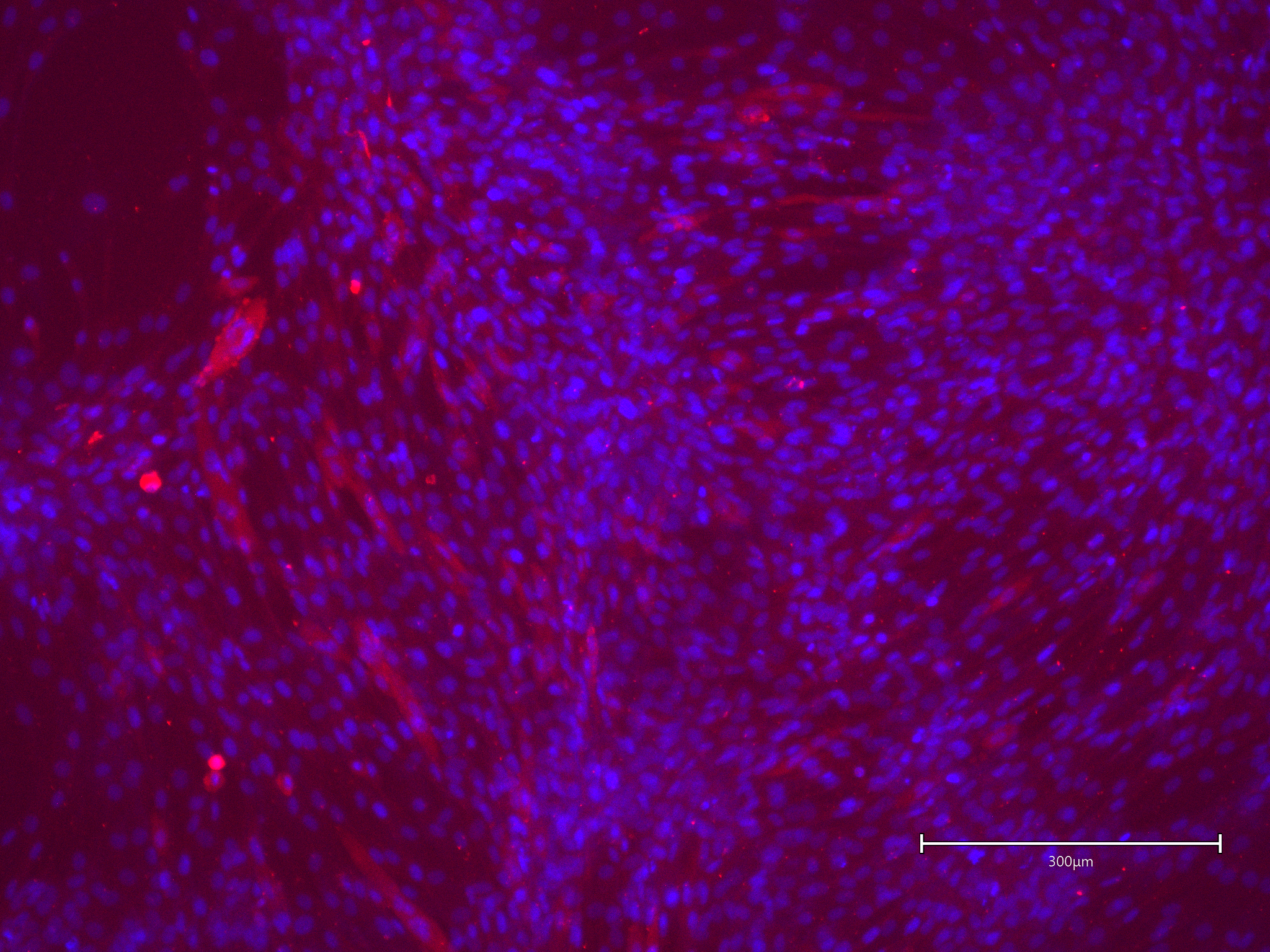

Supplement: Supplementary file 12 — Source data Fig. 5 [file 44321_2025_247_MOESM12_ESM.zip › Figure 5/Figure 5_Panel E/Figure 5_Panel E_KO+R215W_IF-merge.tif]

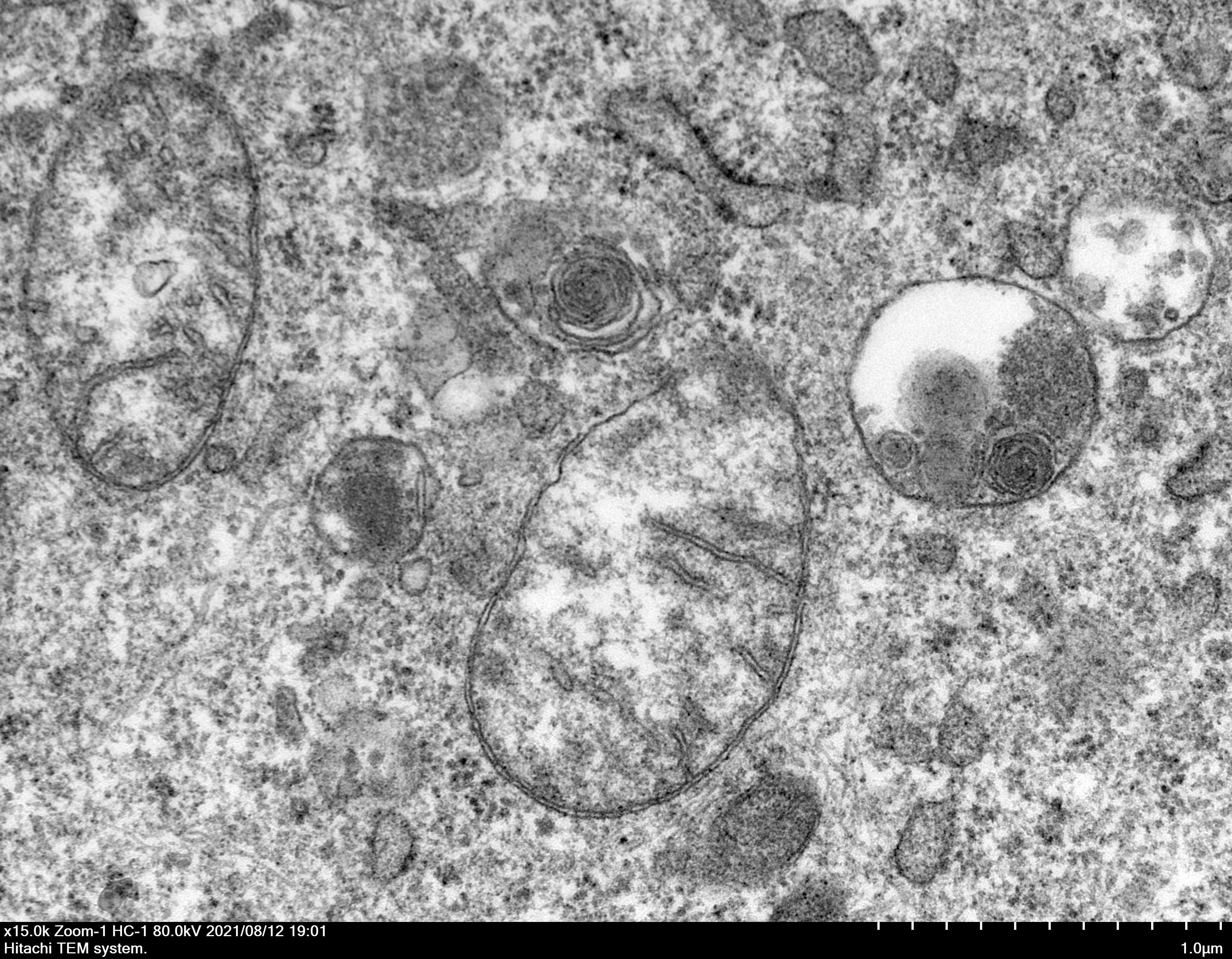

Supplement: Supplementary file 13 — Source data Fig. 6 [file 44321_2025_247_MOESM13_ESM.zip › Figure 6/Figure 6_Panel A/Figure 6_Panel A_KO+EV_TEM.tif]

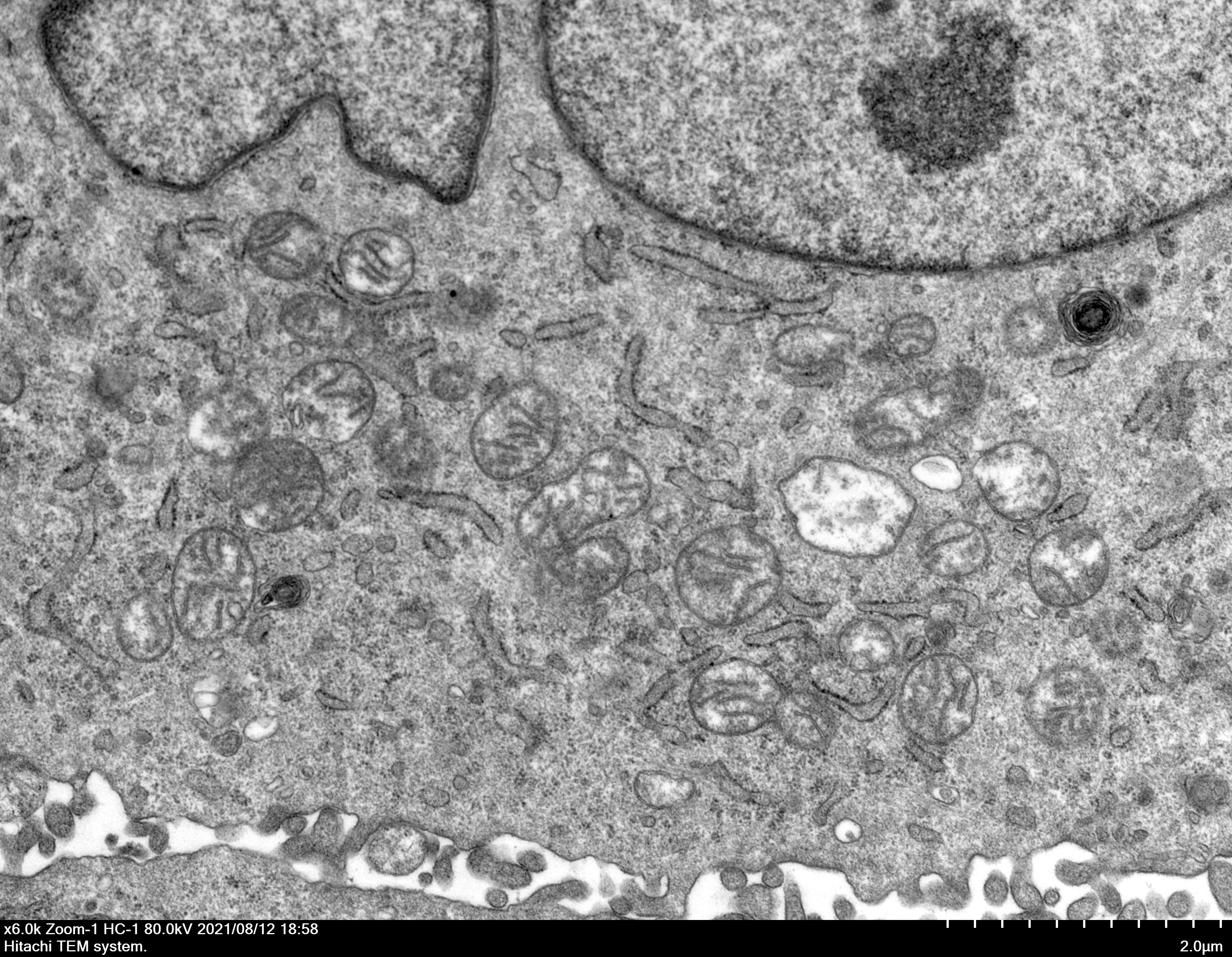

Supplement: Supplementary file 13 — Source data Fig. 6 [file 44321_2025_247_MOESM13_ESM.zip › Figure 6/Figure 6_Panel A/Figure 6_Panel A_blank_TEM.tif]

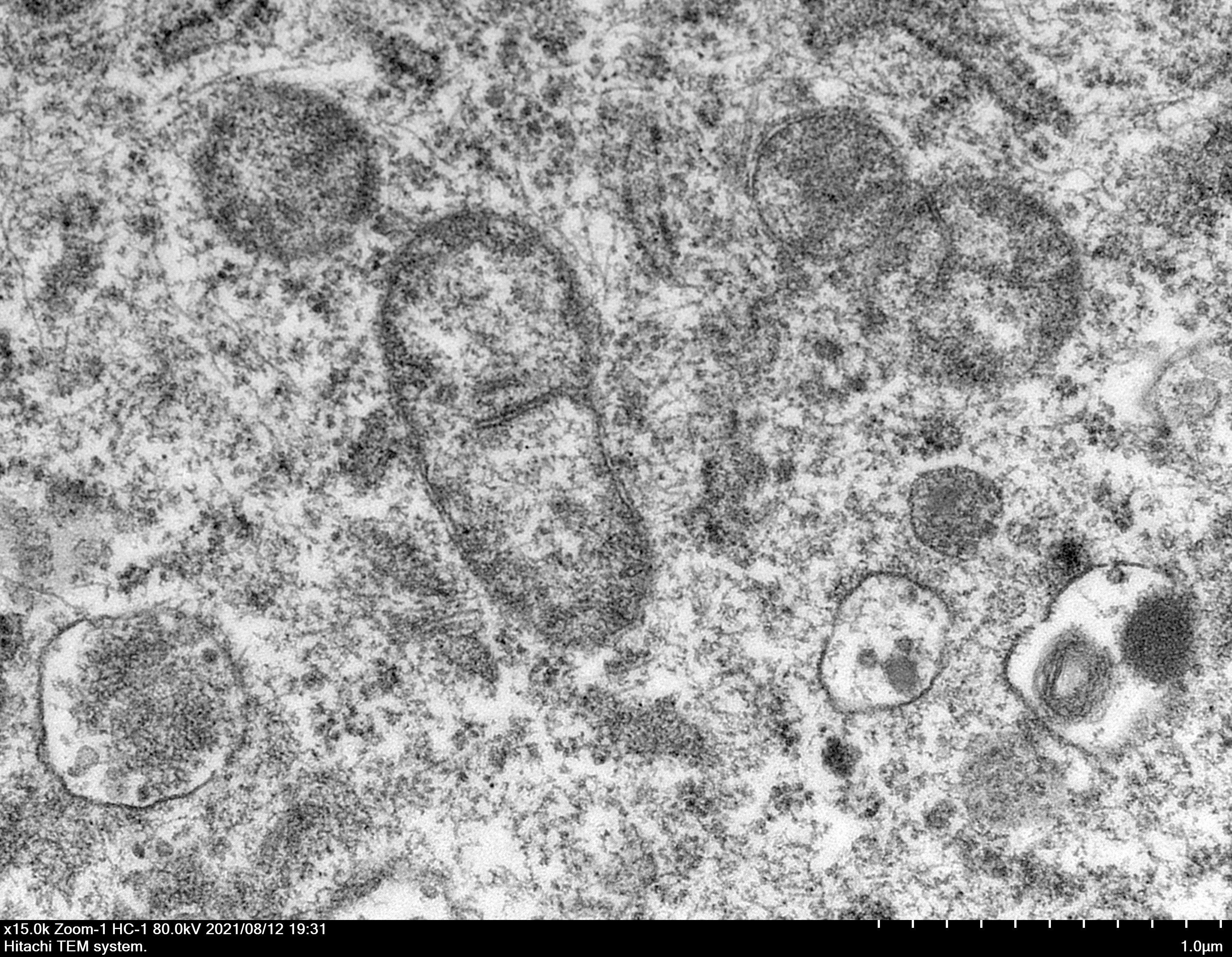

Supplement: Supplementary file 13 — Source data Fig. 6 [file 44321_2025_247_MOESM13_ESM.zip › Figure 6/Figure 6_Panel A/Figure 6_Panel A_KO+WT_TEM.tif]

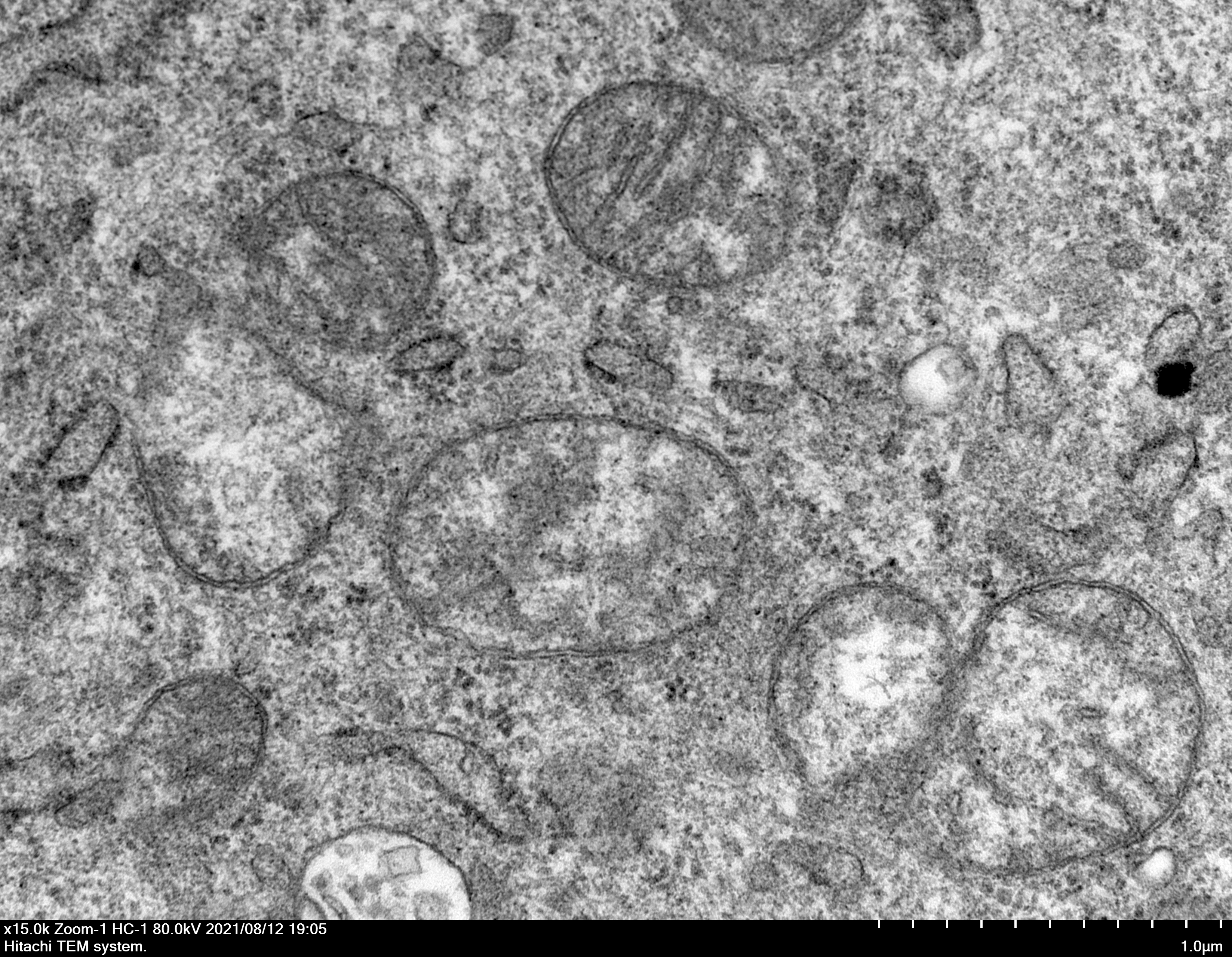

Supplement: Supplementary file 13 — Source data Fig. 6 [file 44321_2025_247_MOESM13_ESM.zip › Figure 6/Figure 6_Panel A/Figure 6_Panel A_FOXK2-KO_TEM.tif]

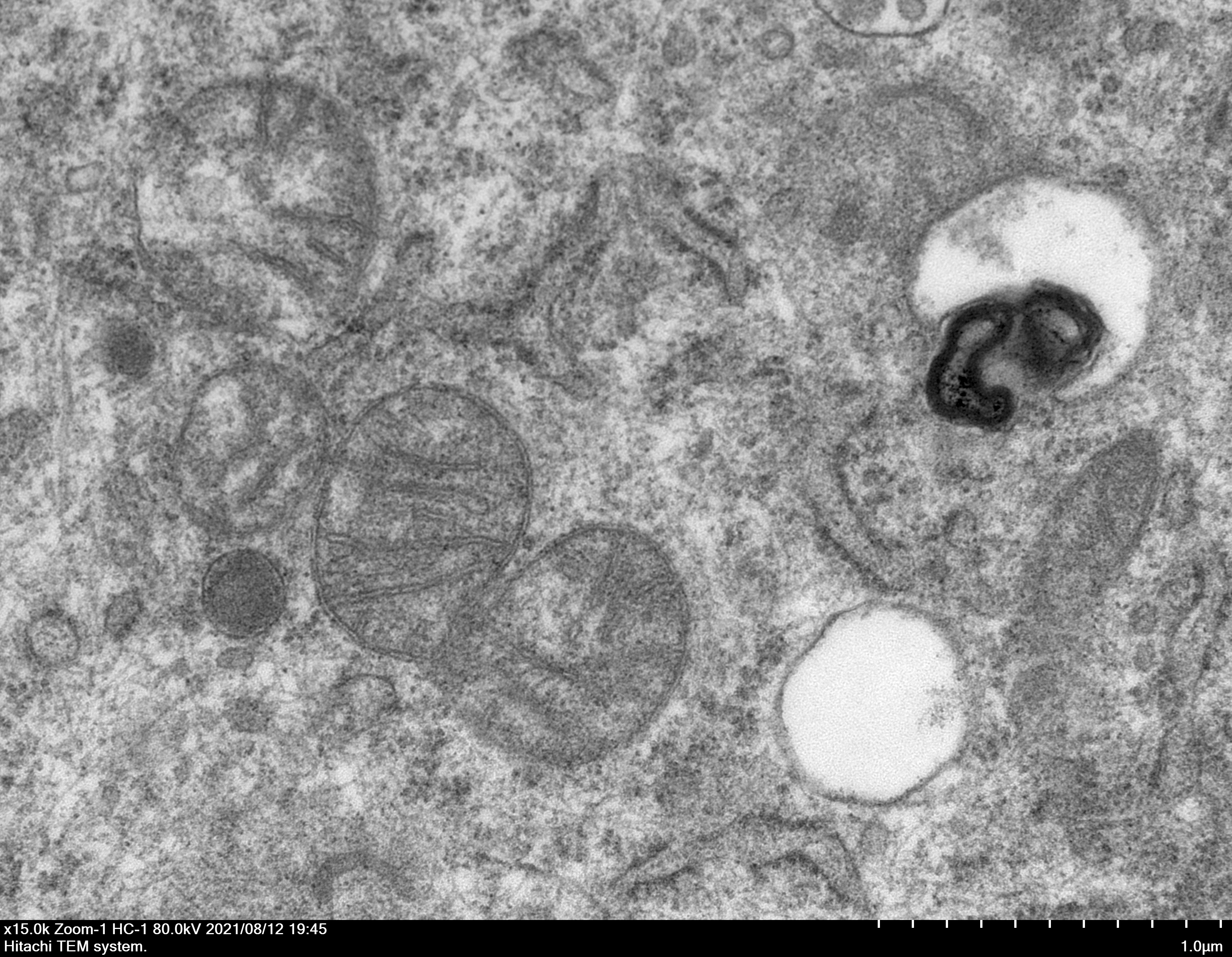

Supplement: Supplementary file 13 — Source data Fig. 6 [file 44321_2025_247_MOESM13_ESM.zip › Figure 6/Figure 6_Panel A/Figure 6_Panel A_control_TEM.tif]

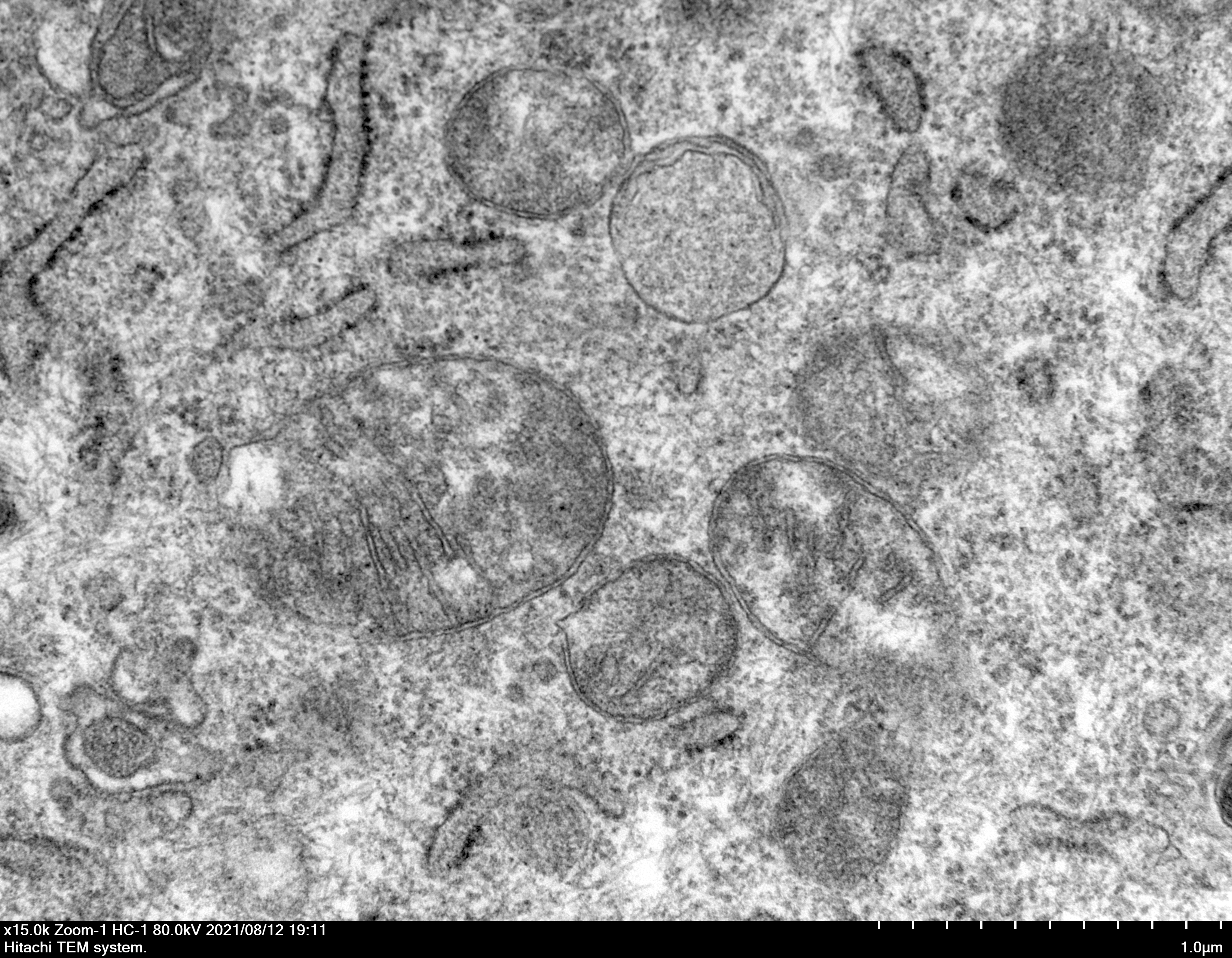

Supplement: Supplementary file 13 — Source data Fig. 6 [file 44321_2025_247_MOESM13_ESM.zip › Figure 6/Figure 6_Panel A/Figure 6_Panel A_KO+R215W_TEM.tif]

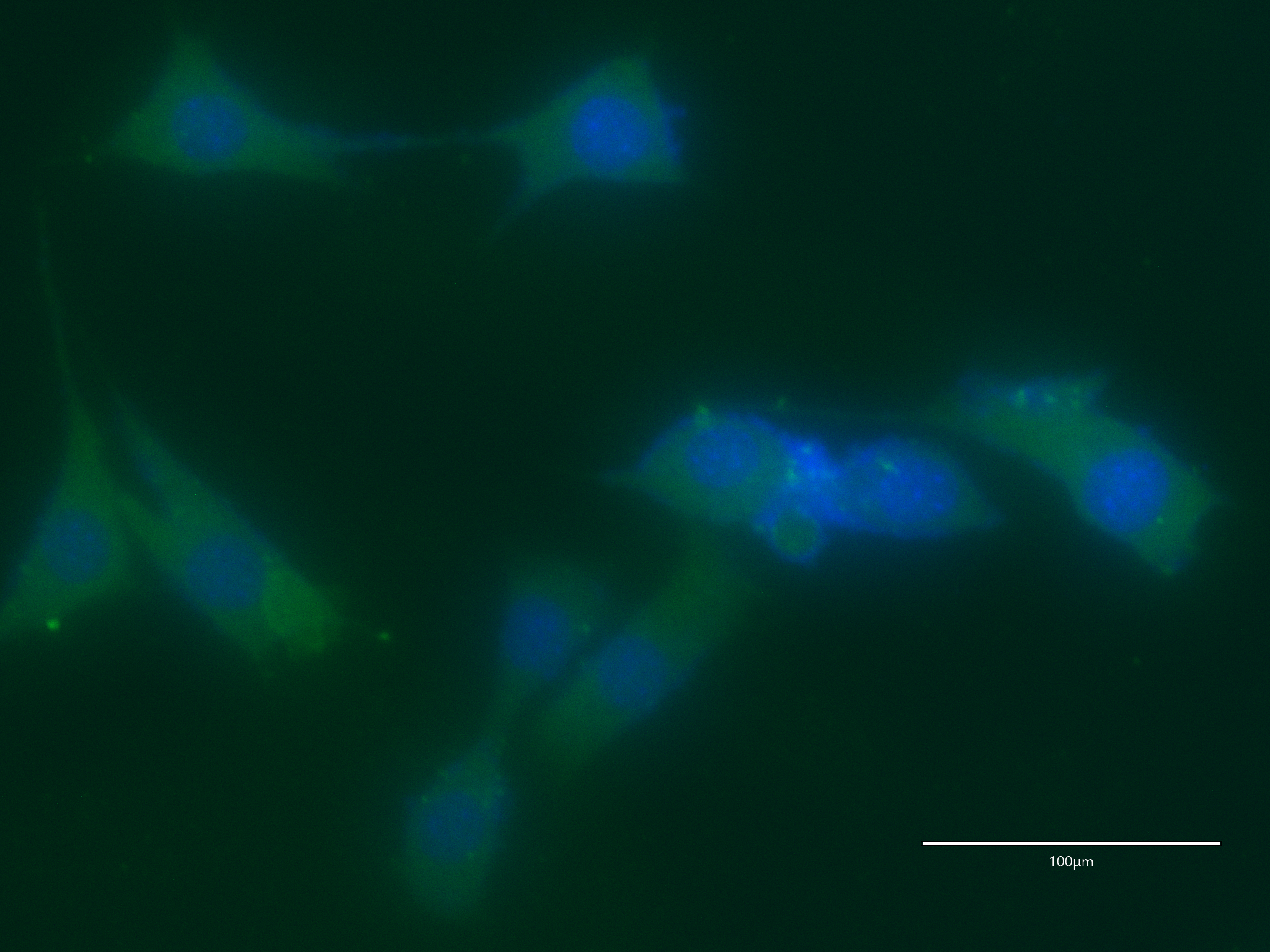

Supplement: Supplementary file 13 — Source data Fig. 6 [file 44321_2025_247_MOESM13_ESM.zip › Figure 6/Figure 6_Panel B/Figure 6_Panel B_control_IF-DRP1.tif]

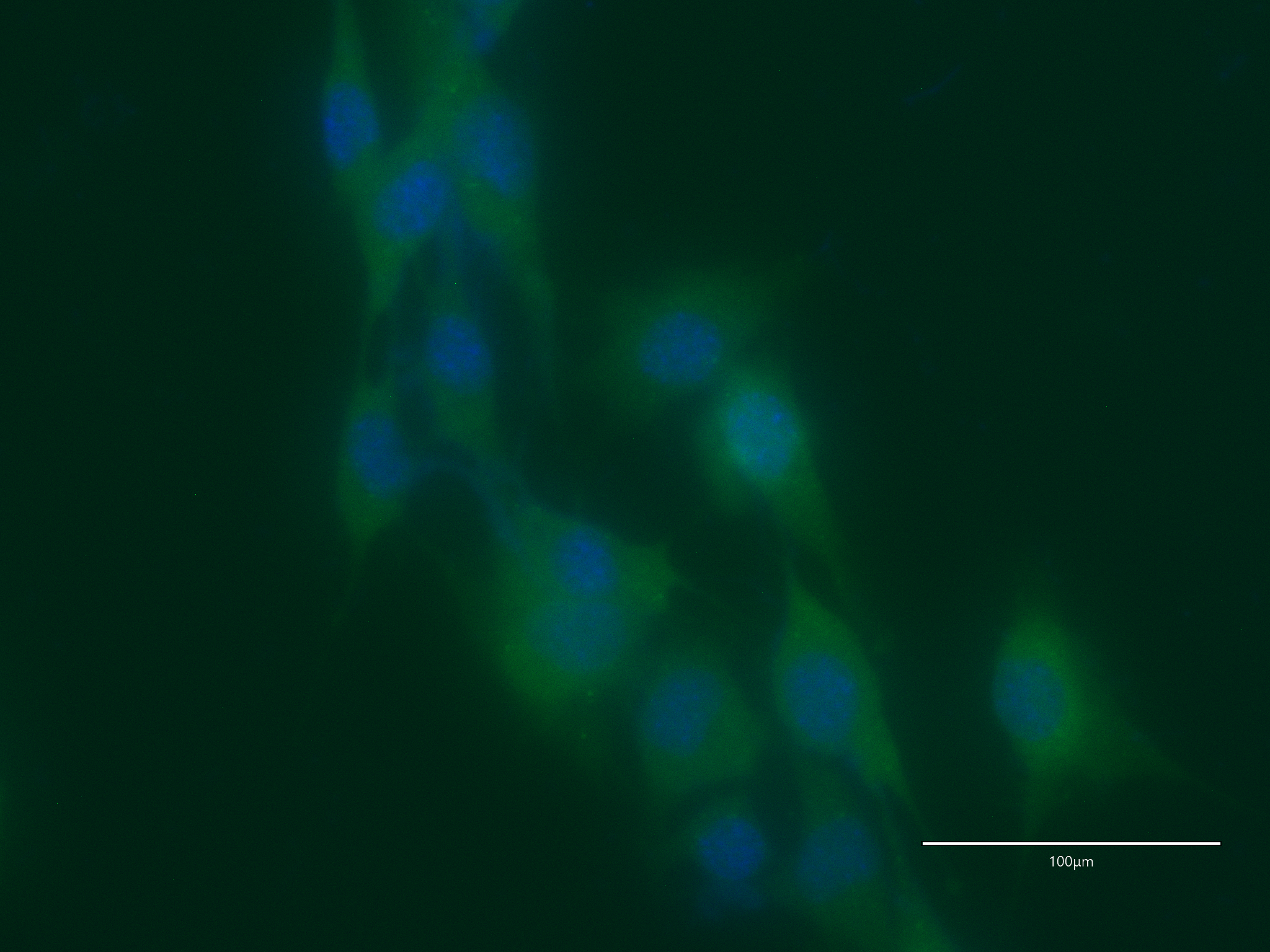

Supplement: Supplementary file 13 — Source data Fig. 6 [file 44321_2025_247_MOESM13_ESM.zip › Figure 6/Figure 6_Panel B/Figure 6_Panel B_KO+WT_IF-DRP1.tif]

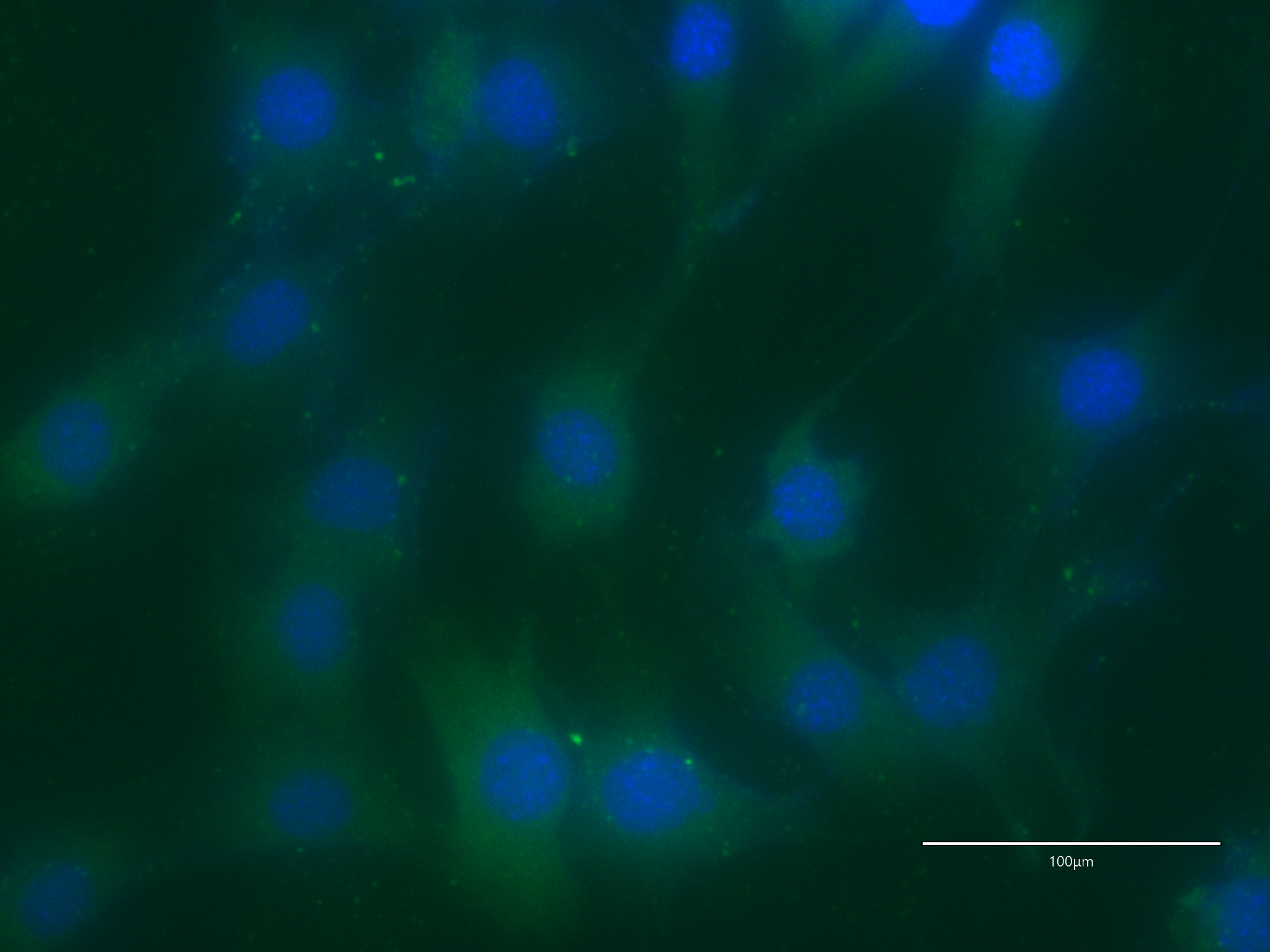

Supplement: Supplementary file 13 — Source data Fig. 6 [file 44321_2025_247_MOESM13_ESM.zip › Figure 6/Figure 6_Panel B/Figure 6_Panel B_blank_IF-DRP1.tif]

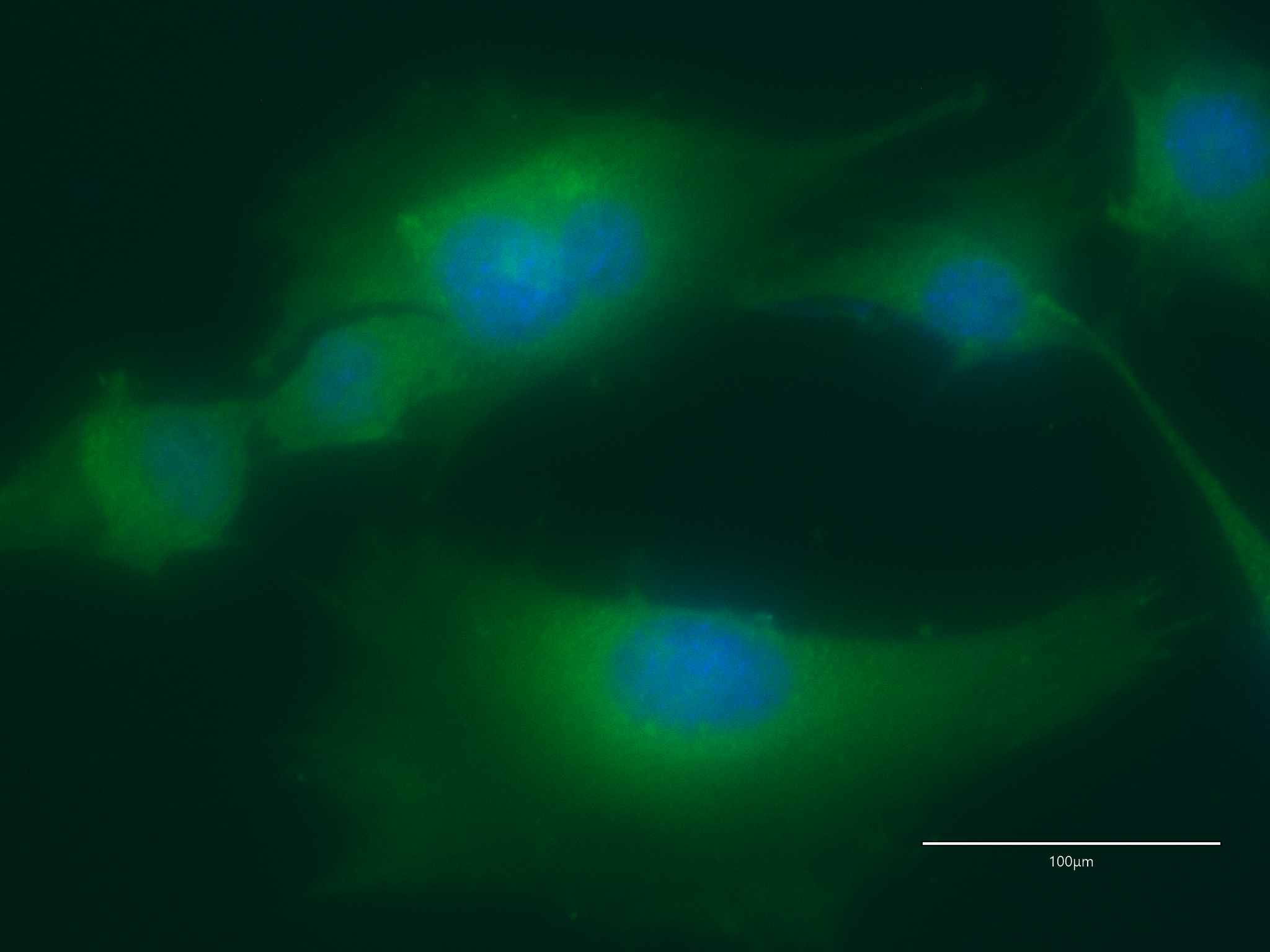

Supplement: Supplementary file 13 — Source data Fig. 6 [file 44321_2025_247_MOESM13_ESM.zip › Figure 6/Figure 6_Panel B/Figure 6_Panel B_KO+R215W_IF-DRP1.tif]

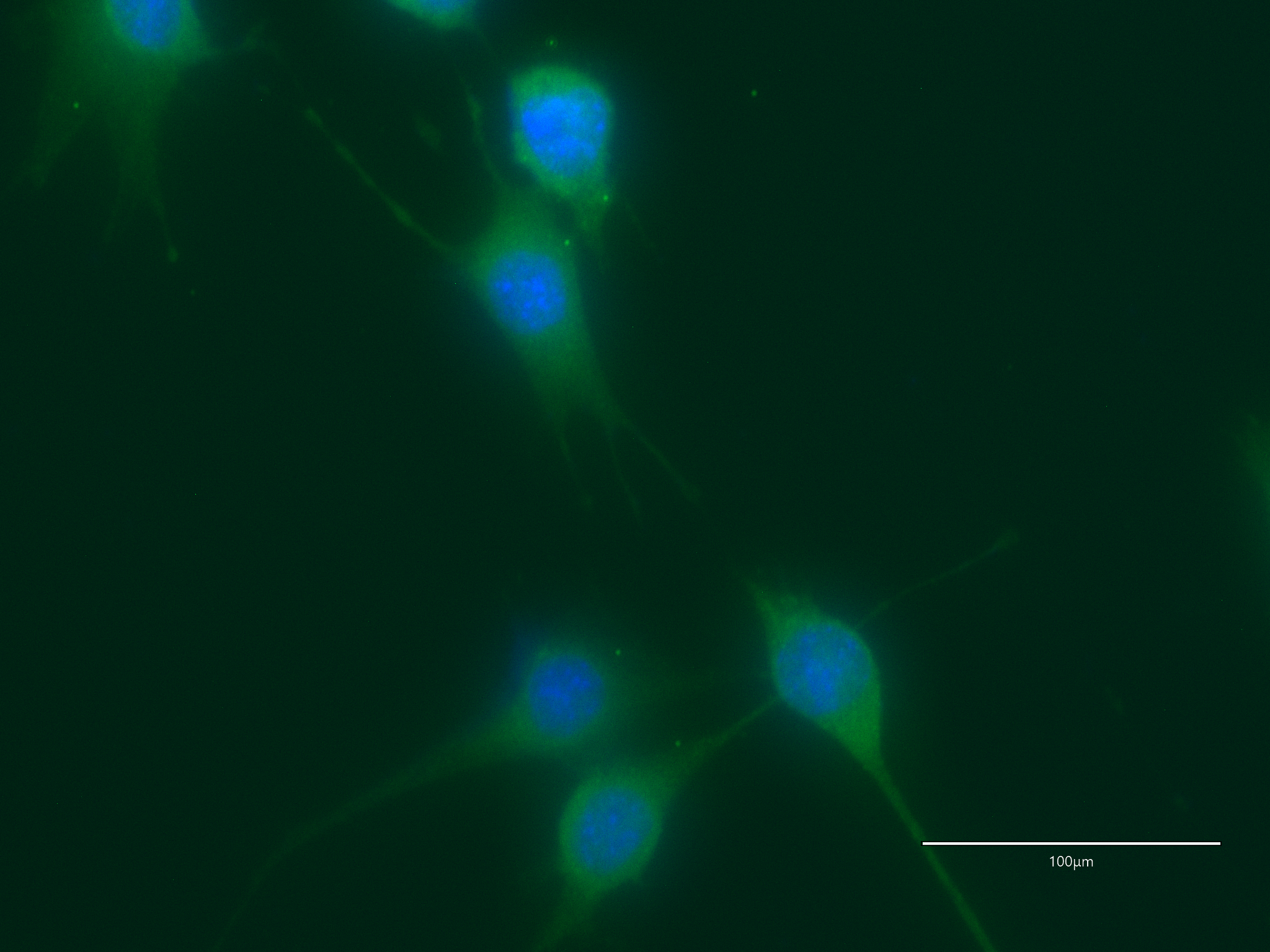

Supplement: Supplementary file 13 — Source data Fig. 6 [file 44321_2025_247_MOESM13_ESM.zip › Figure 6/Figure 6_Panel B/Figure 6_Panel B_KO+EV_IF-DRP1.tif]

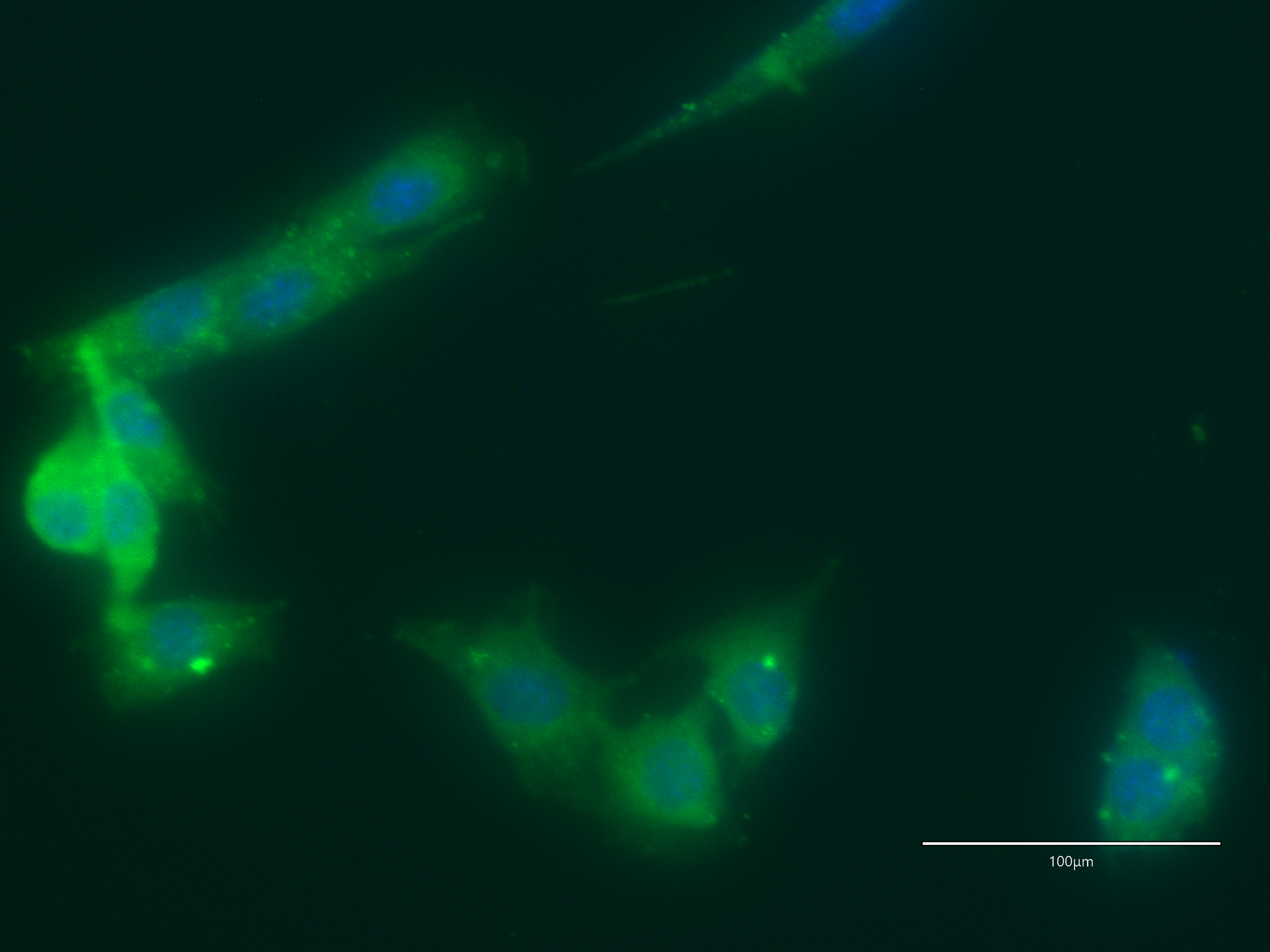

Supplement: Supplementary file 13 — Source data Fig. 6 [file 44321_2025_247_MOESM13_ESM.zip › Figure 6/Figure 6_Panel B/Figure 6_Panel B_FOXK2-KO_IF-DRP1.tif]

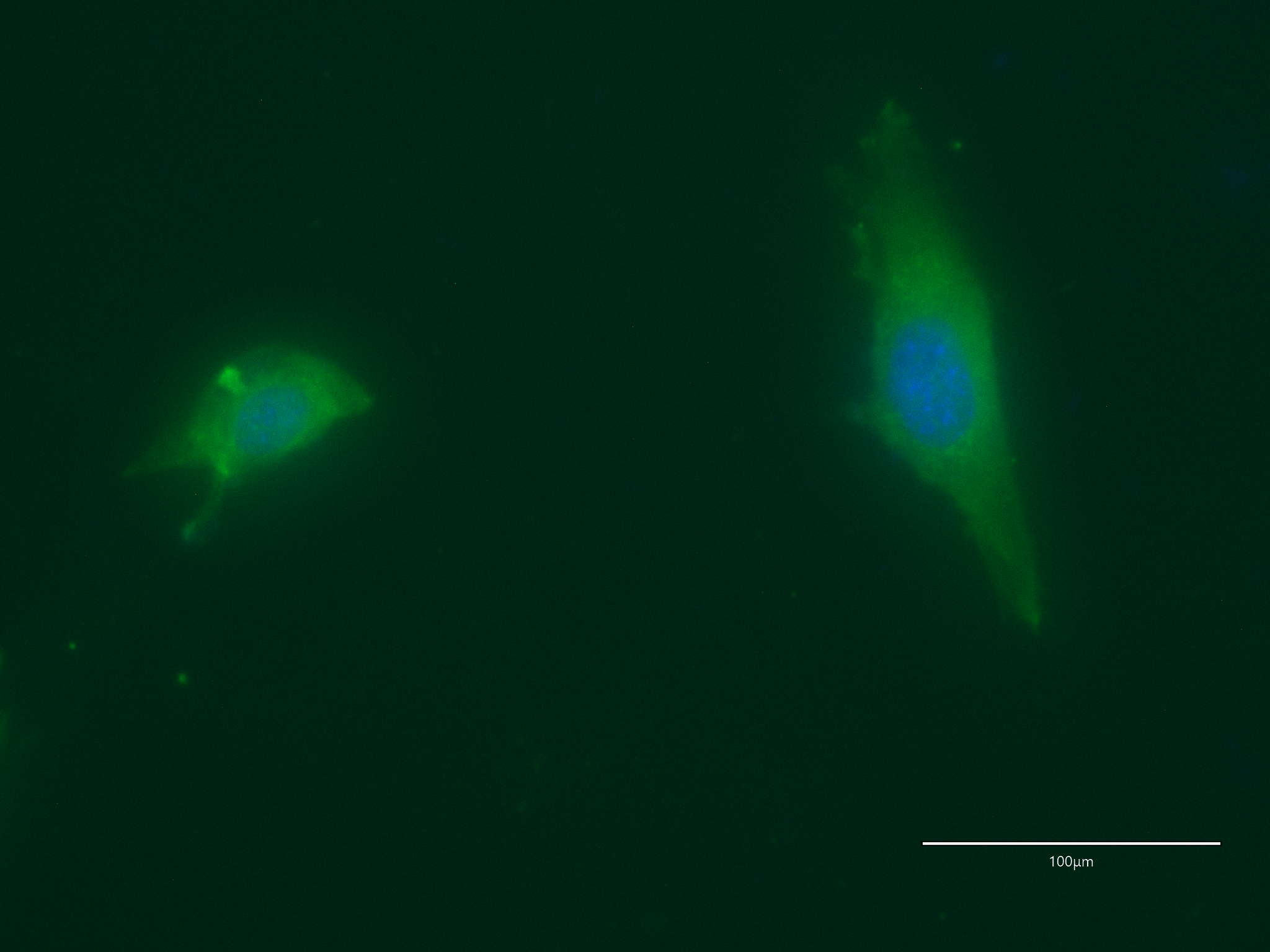

Supplement: Supplementary file 13 — Source data Fig. 6 [file 44321_2025_247_MOESM13_ESM.zip › Figure 6/Figure 6_Panel C/Figure 6_Panel C_control_IF-OPA1.tif]
