# Supplementary figures and images for: FOXK2 in skeletal muscle development: a new pathogenic gene for congenital myopathy with ptosis (part 2 of 2)
Source: EMBO Mol Med. 2025 May 23;17(7):1599–630. doi: 10.1038/s44321-025-00247-x (PMC12254393; doi:10.1038/s44321-025-00247-x)

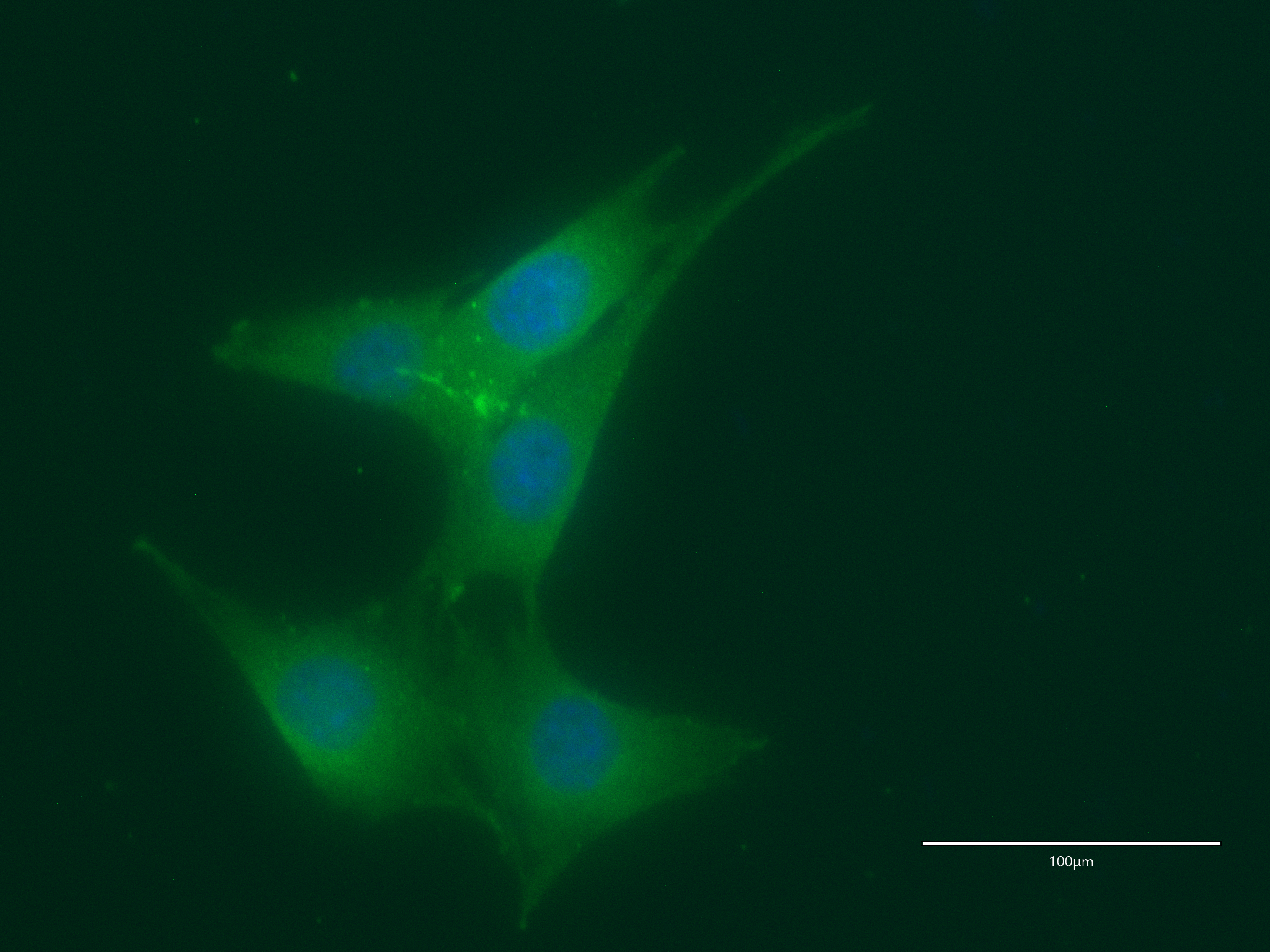

Supplement: Supplementary file 13 — Source data Fig. 6 [file 44321_2025_247_MOESM13_ESM.zip › Figure 6/Figure 6_Panel C/Figure 6_Panel C_blank_IF-OPA1.tif]

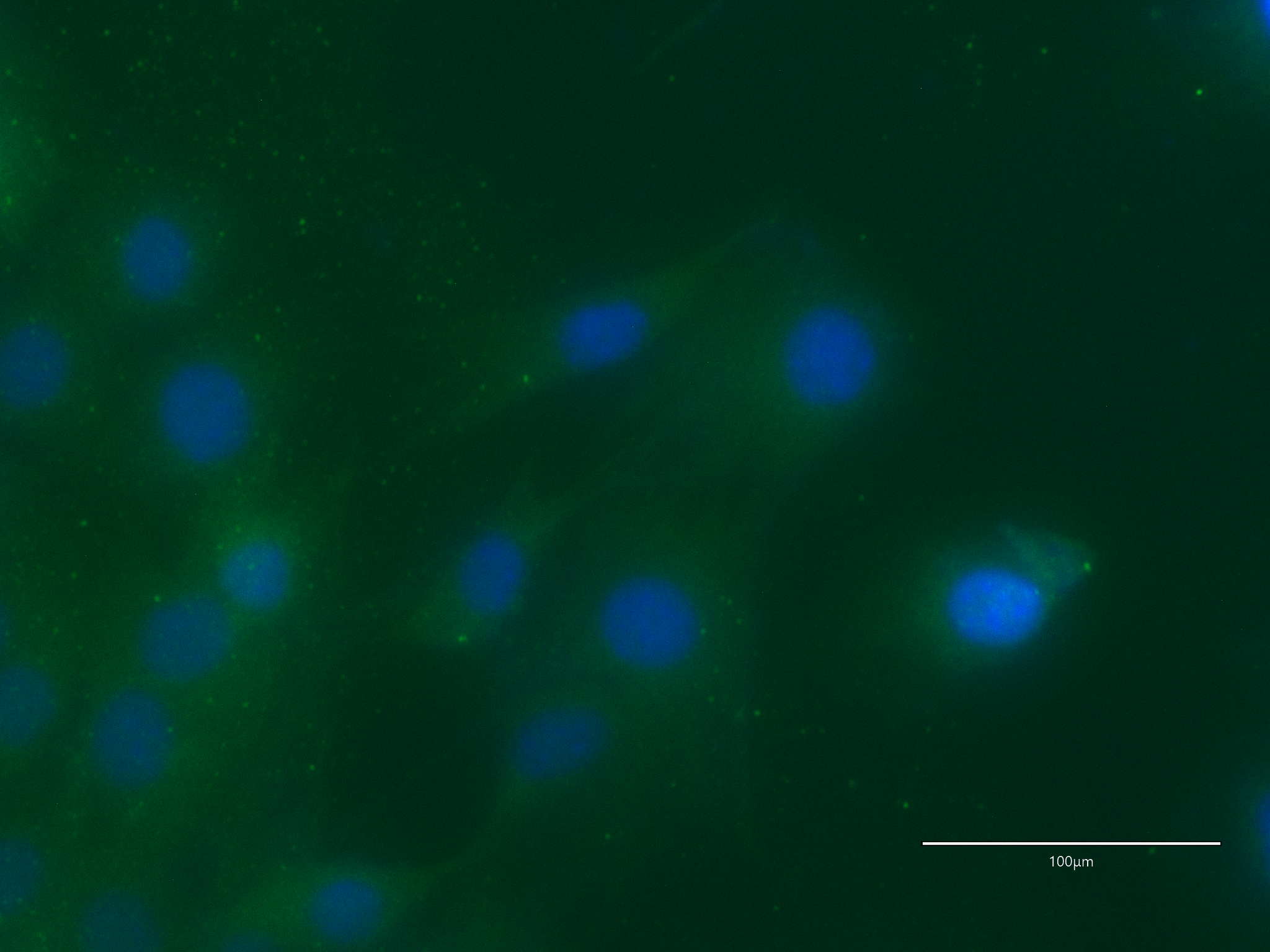

Supplement: Supplementary file 13 — Source data Fig. 6 [file 44321_2025_247_MOESM13_ESM.zip › Figure 6/Figure 6_Panel C/Figure 6_Panel C_KO+R215W_IF-OPA1.tif]

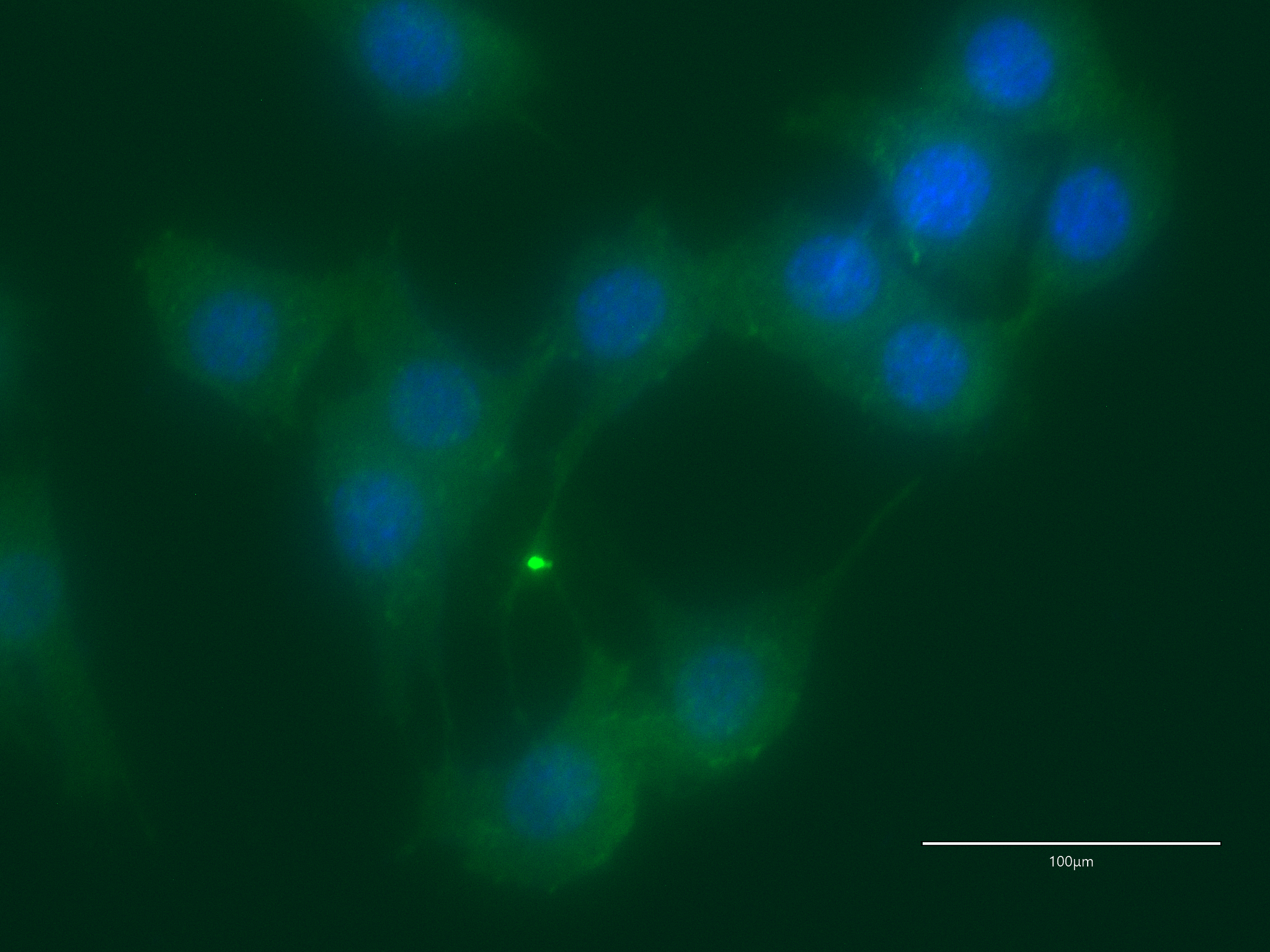

Supplement: Supplementary file 13 — Source data Fig. 6 [file 44321_2025_247_MOESM13_ESM.zip › Figure 6/Figure 6_Panel C/Figure 6_Panel C_FOXK2-KO_IF-OPA1.tif]

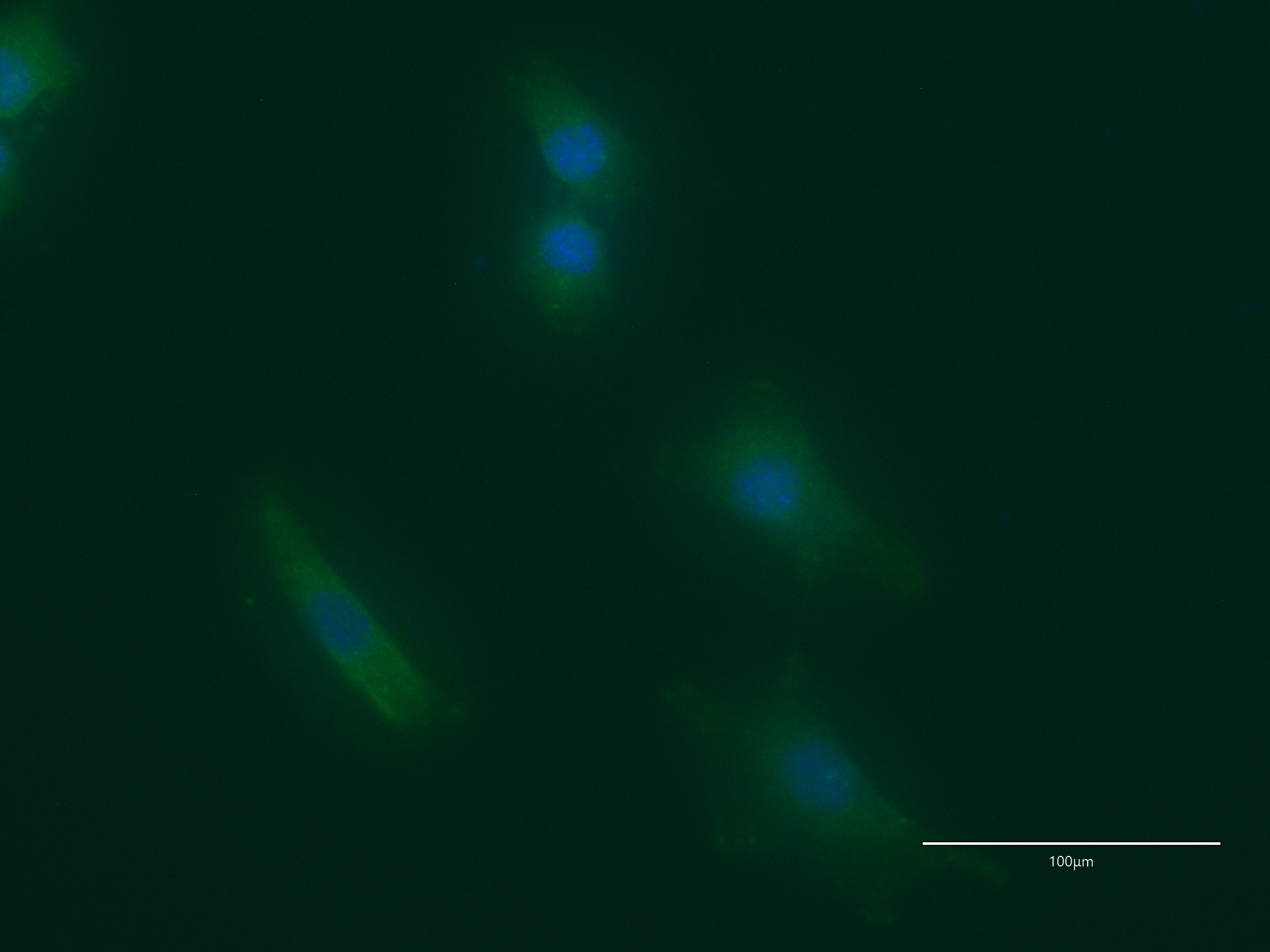

Supplement: Supplementary file 13 — Source data Fig. 6 [file 44321_2025_247_MOESM13_ESM.zip › Figure 6/Figure 6_Panel C/Figure 6_Panel C_KO+EV_IF-OPA1.tif]

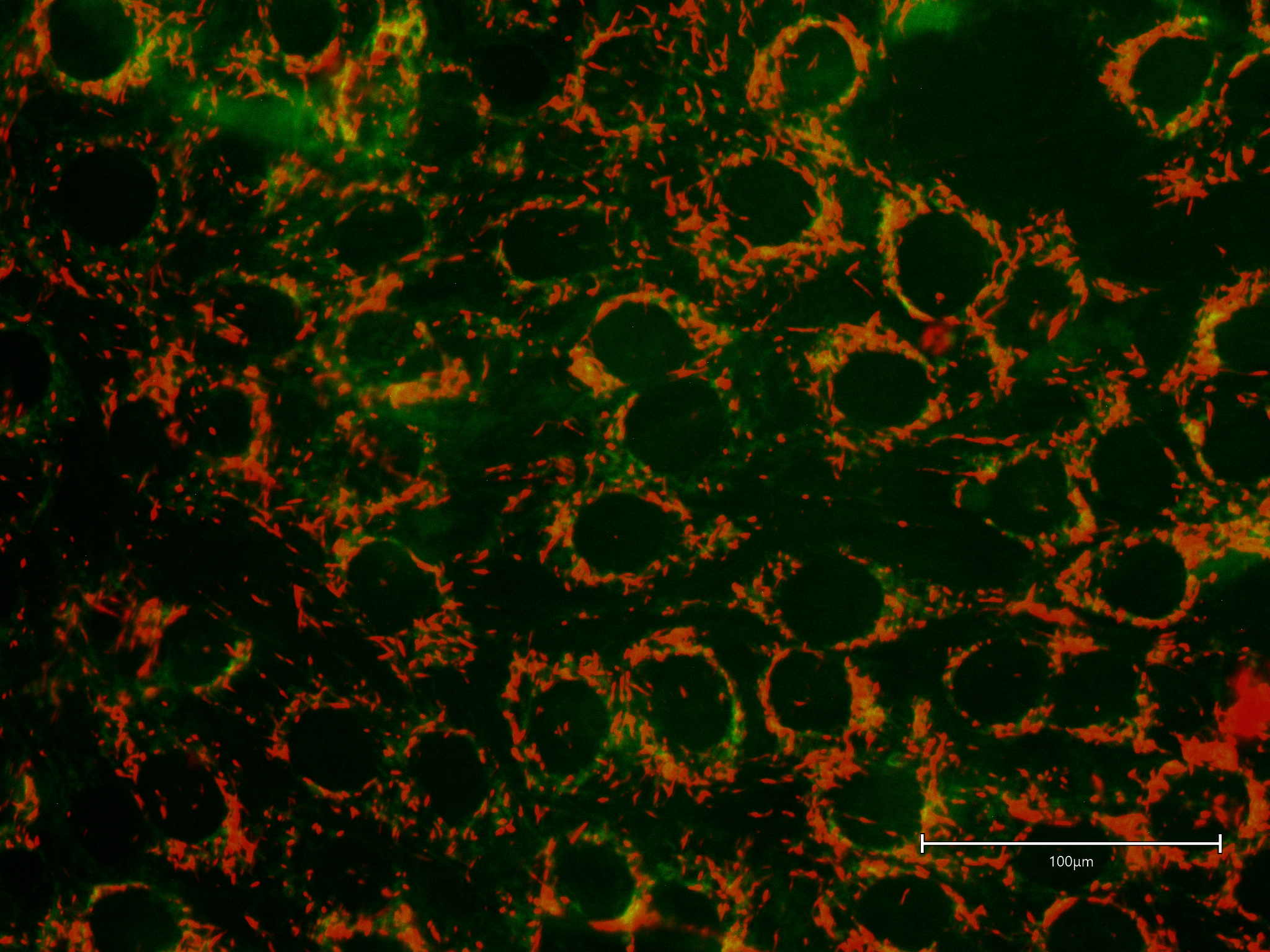

Supplement: Supplementary file 13 — Source data Fig. 6 [file 44321_2025_247_MOESM13_ESM.zip › Figure 6/Figure 6_Panel D/Figure 6_Panel D_control_JC-1.tif]

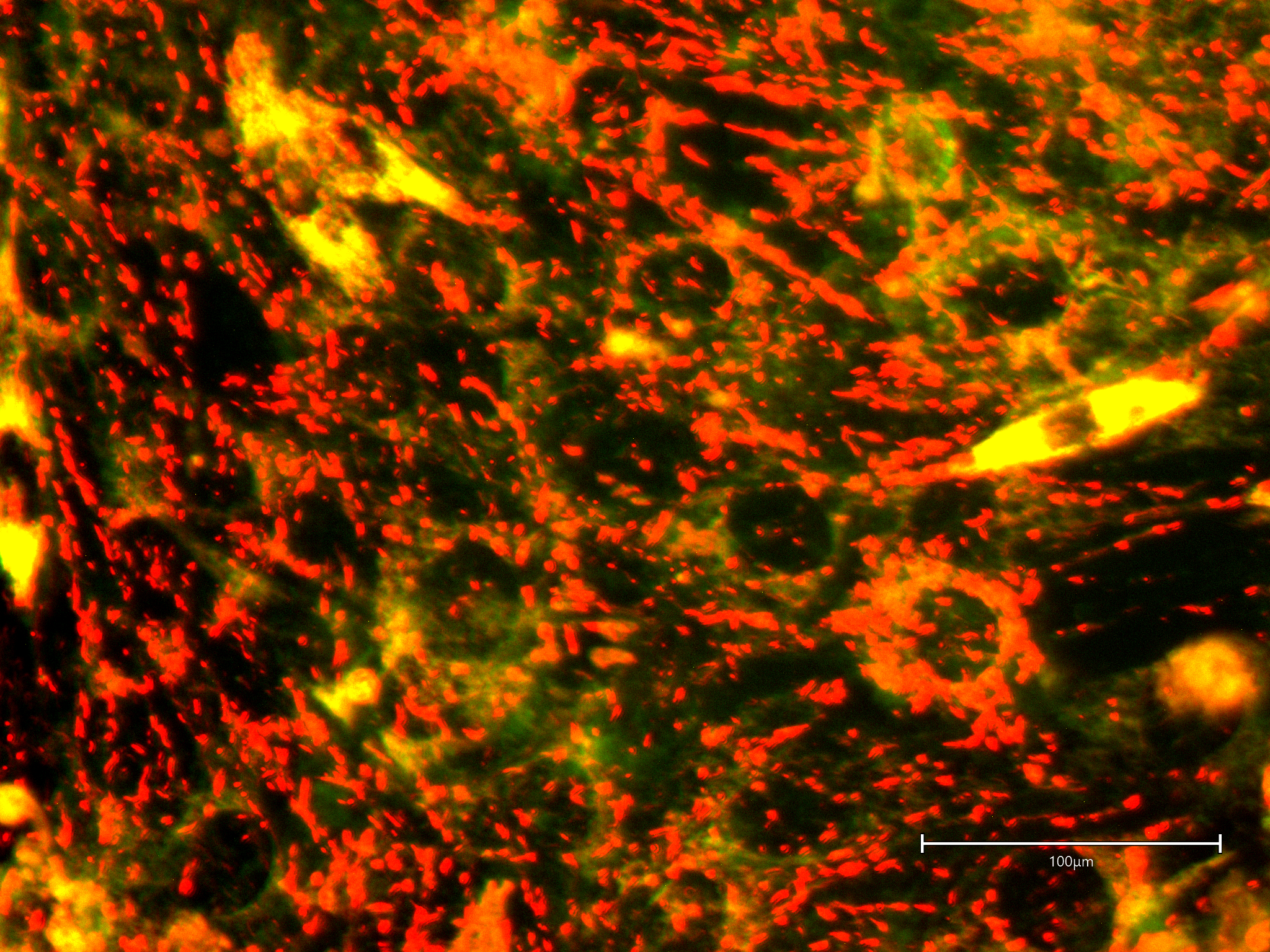

Supplement: Supplementary file 13 — Source data Fig. 6 [file 44321_2025_247_MOESM13_ESM.zip › Figure 6/Figure 6_Panel D/Figure 6_Panel D_KO+WT_JC-1.tif]

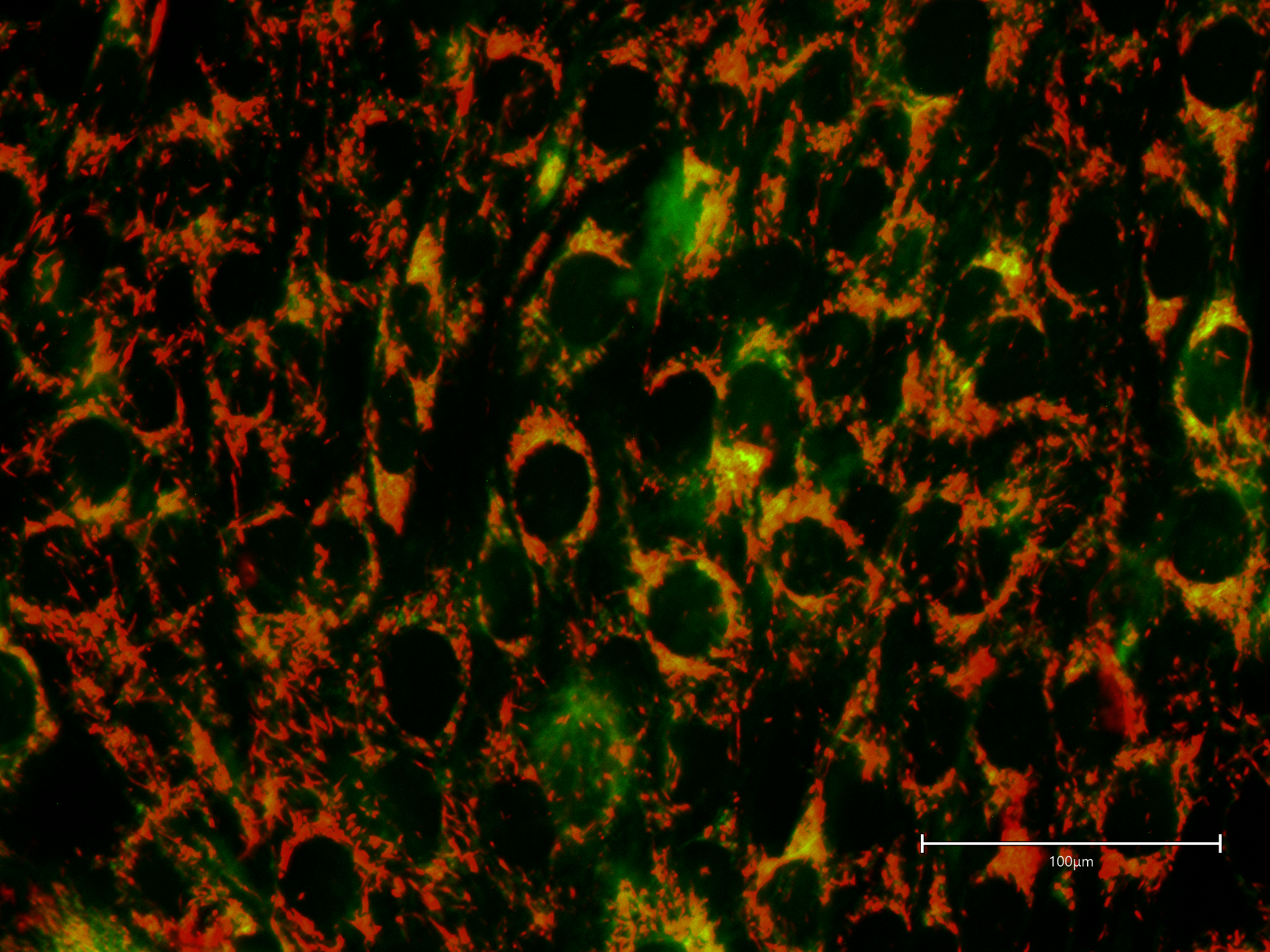

Supplement: Supplementary file 13 — Source data Fig. 6 [file 44321_2025_247_MOESM13_ESM.zip › Figure 6/Figure 6_Panel D/Figure 6_Panel D_KO+R215W_JC-1.tif]

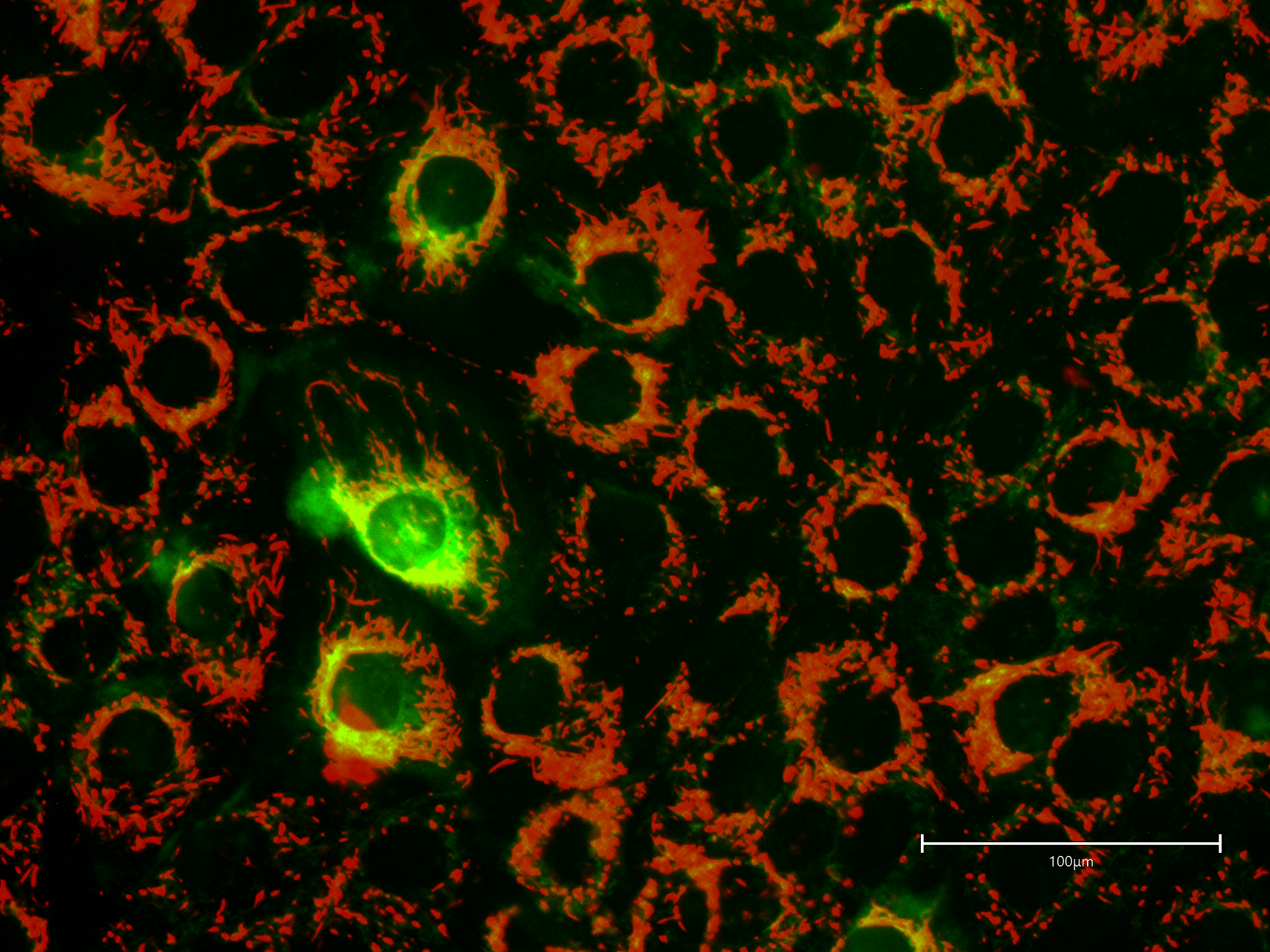

Supplement: Supplementary file 13 — Source data Fig. 6 [file 44321_2025_247_MOESM13_ESM.zip › Figure 6/Figure 6_Panel D/Figure 6_Panel D_KO+EV_JC-1.tif]

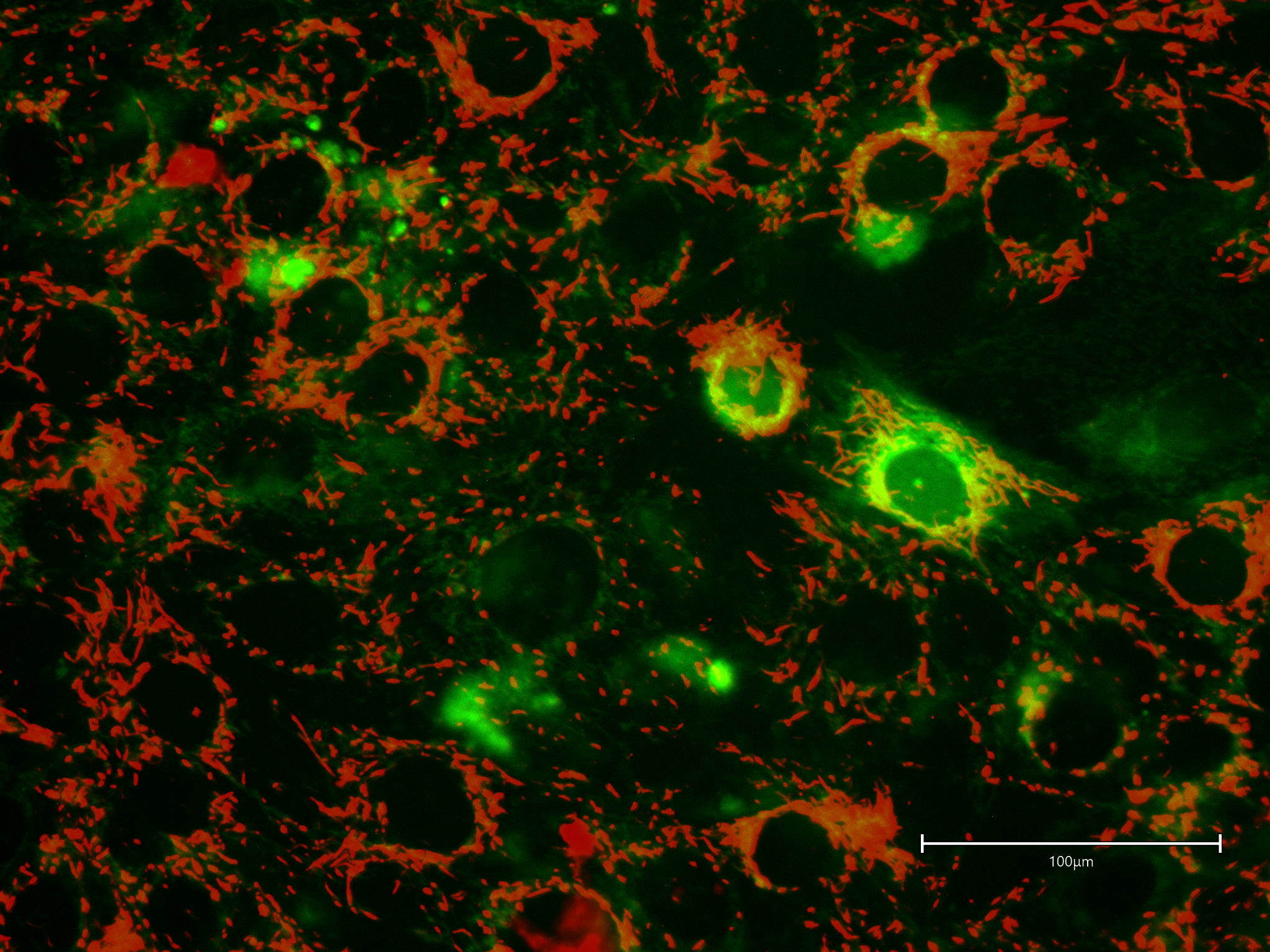

Supplement: Supplementary file 13 — Source data Fig. 6 [file 44321_2025_247_MOESM13_ESM.zip › Figure 6/Figure 6_Panel D/Figure 6_Panel D_FOXK2-KO_JC-1.tif]

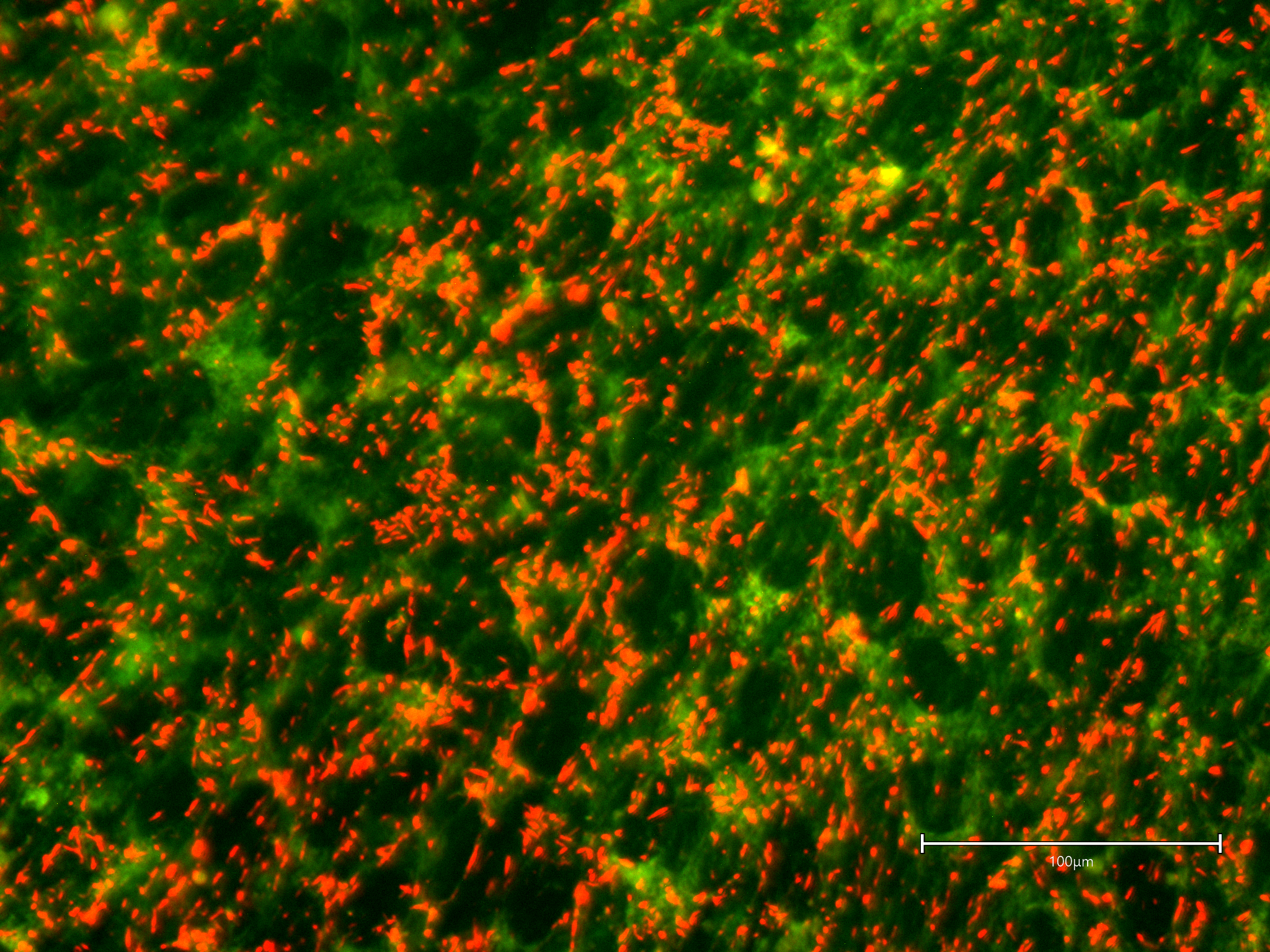

Supplement: Supplementary file 13 — Source data Fig. 6 [file 44321_2025_247_MOESM13_ESM.zip › Figure 6/Figure 6_Panel D/Figure 6_Panel D_blank_JC-1.tif]
