# Supplementary material for: FOXK2 in skeletal muscle development: a new pathogenic gene for congenital myopathy with ptosis
Source: EMBO Mol Med. 2025 May 23;17(7):1599–630. doi: 10.1038/s44321-025-00247-x (PMC12254393; doi:10.1038/s44321-025-00247-x)
Supplement: Supplementary file 15 — Expanded View Figures [file 44321_2025_247_MOESM15_ESM.pdf]

Expanded View Figures

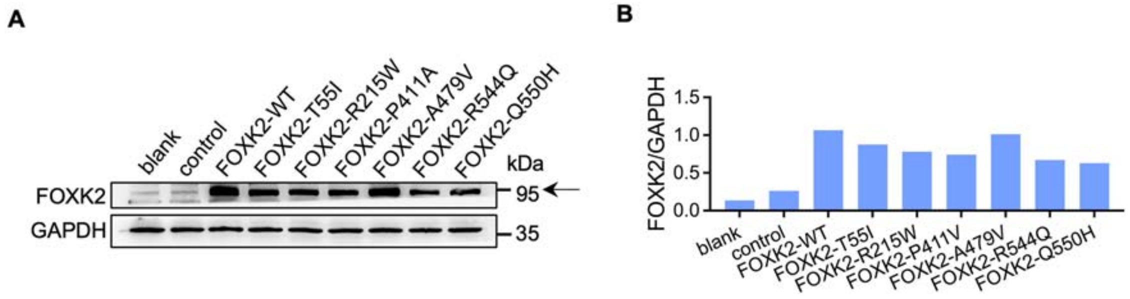

Figure EV1. The protein expression of FOXK2 mutants in HEK-293T cells.

(**A**, **B**) Western blot analysis of FOXK2 expression level in HEK-293T cells. The black arrow points to specific protein band. The statistical analysis of FOXK2 protein expression level relative to GAPDH is shown in (**B**).

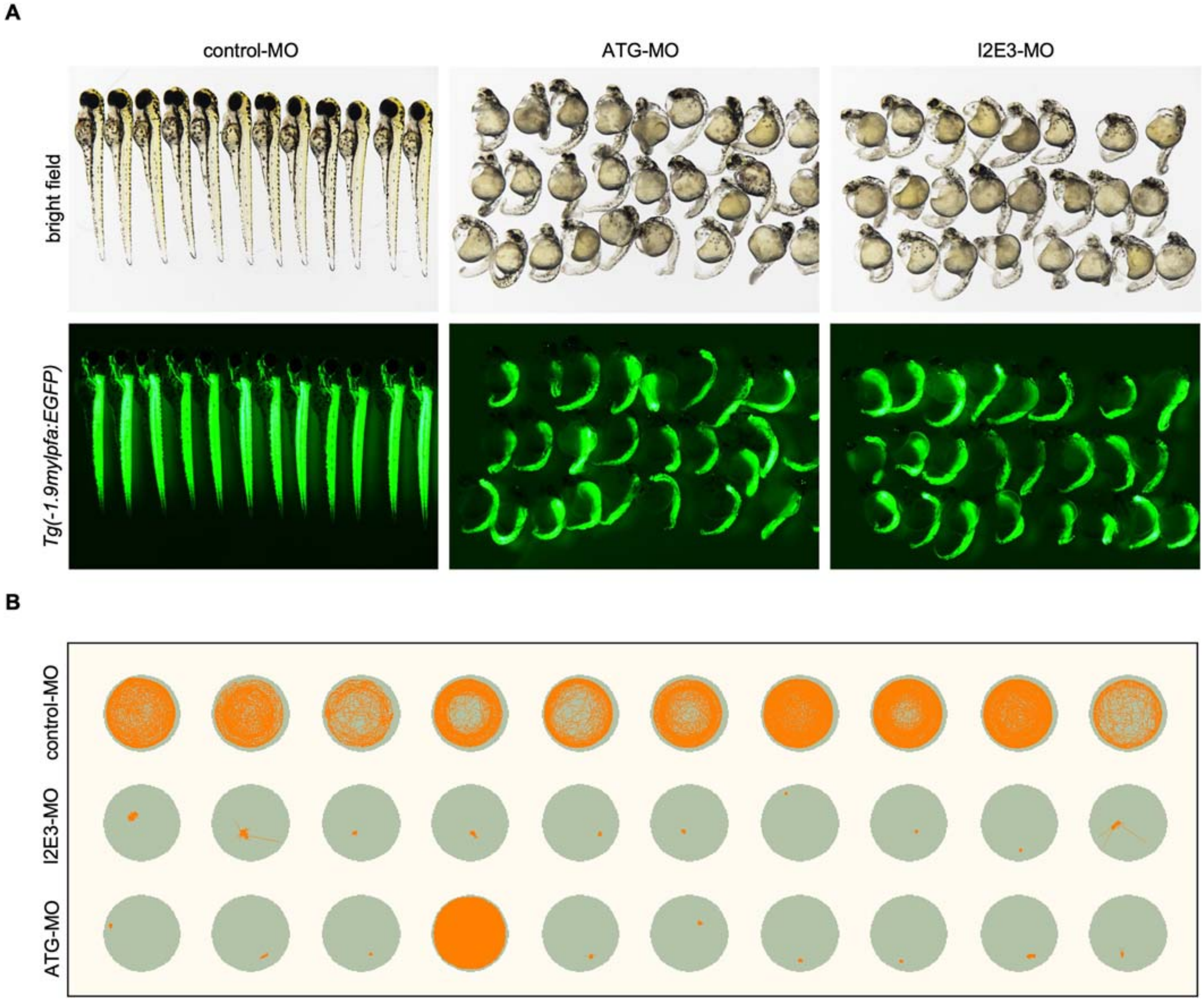

Figure EV2. Gross morphology and locomotor capacity of *foxk2* morphants.

(**A**) Gross morphology of *Tg(-1.9mylpfa:EGFP)* zebrafish larvae at 72 hpf. (**B**) Digital movement trajectories of zebrafish larvae at 5 dpf. One embryo was placed per well ($n = 10$ per group). MO morpholino, hpf hours post fertilization, dpf days post fertilization.

 © The Author(s)

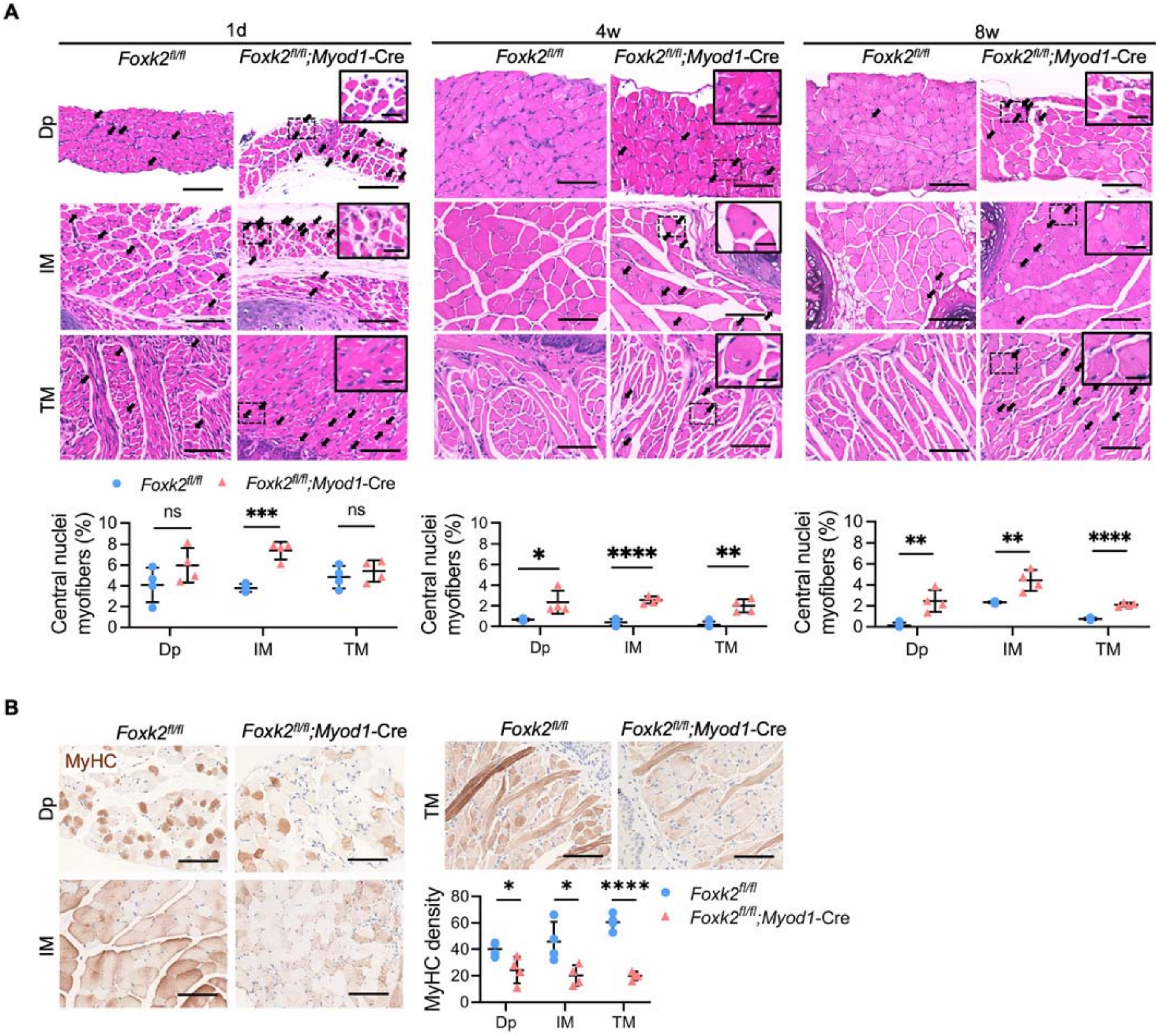

Figure EV3. Abnormal skeletal muscle development in muscle stem cells-specific *Foxk2* conditional knockout mice.

(**A**) H&E staining illustrating histological aspects and the percentage of central nuclei myofibers in the skeletal muscles (Dp, IM, and TM) of *Foxk2*$^{fl/fl}$ and *Foxk2*$^{fl/fl}$; *Myod1*-Cre littermates at postnatal day 1, 4 weeks, and 8 weeks ($n = 4$ areas). Black arrows indicate central nuclei. The inset in the upper right corner (scale bars: 25 µm) is an enlarged view of the boxed area in the main image, highlighting central nuclei myofibers (scale bars: 100 µm). *P* value: 1 d: Dp, ns-$P = 0.1619$; IM, ***$P = 0.0003$; TM, ns-$P = 0.4606$. 4w: Dp, *$P = 0.0244$; IM, ****$P < 0.0001$; TM, **$P = 0.0023$. 8w: Dp, **$P = 0.0051$; IM, **$P = 0.0057$; TM, ****$P < 0.0001$. (**B**) IHC staining of MyHC in the skeletal muscles (Dp, IM, TM) of *Foxk2*$^{fl/fl}$ and *Foxk2*$^{fl/fl}$; *Myod1*-Cre littermates at 8 weeks ($n = 4$ areas). MyHC was quantified by InDen/Area using ImageJ software. scale bars: 100 µm. *P* value: Dp, *$P = 0.0332$; IM, *$P = 0.0238$; TM, ****$P < 0.0001$. Data were analyzed by Student's t test. All error bars indicate mean ± standard deviation. d day, w week, Dp diaphragm, IM intercostal muscle, TM tongue muscle, H&E hematoxylin-eosin staining, IHC immunohistochemistry.

© The Author(s)

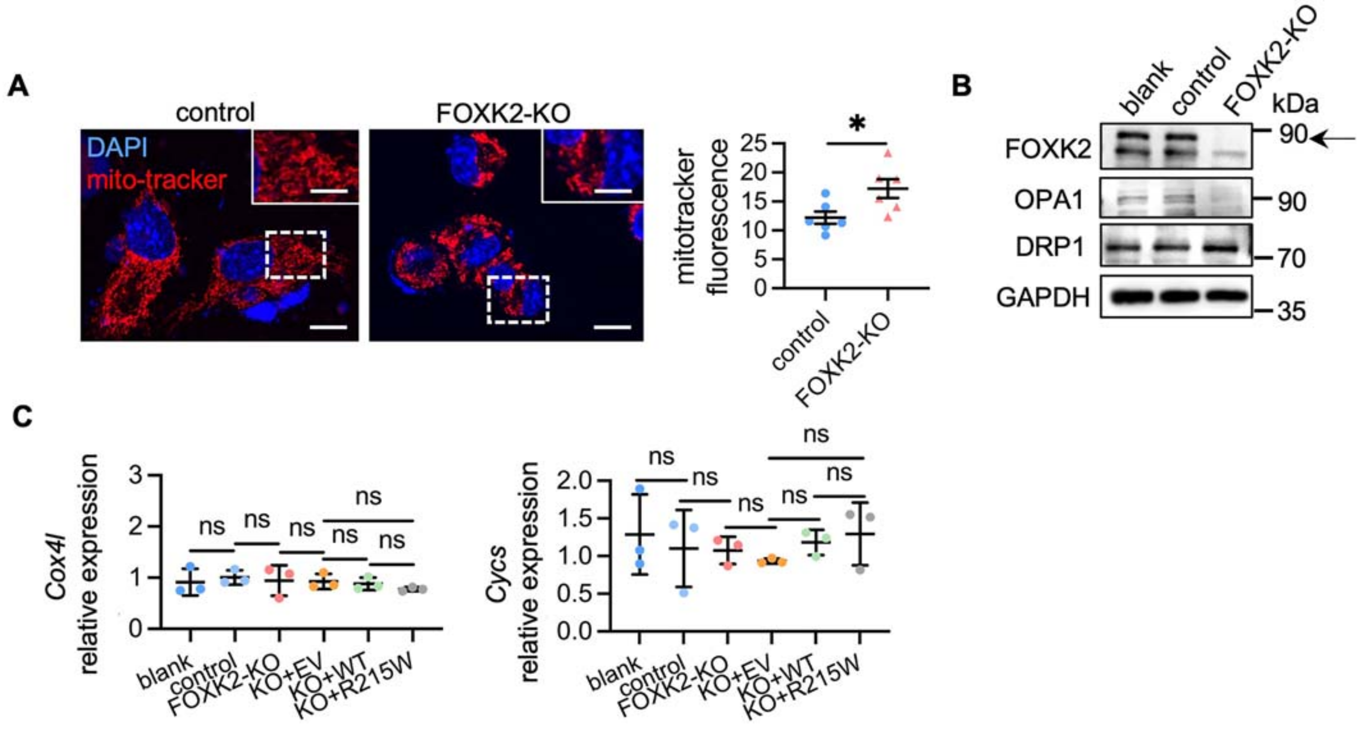

Figure EV4. Mitochondrial morphology and mitochondrial function-related gene expression in C2C12 cells.

(**A**) Confocal images of mitochondria labeled with mitotracker (red) and nuclei stained with DAPI (blue). The inset in the upper right corner provides an enlarged view (scale bars: 8 µm) of the boxed area in the main image, highlighting mitochondria (scale bars: 20 µm). Fluorescence intensity was quantified using ImageJ software ($n = 6$ areas). *P* value: $^*P = 0.0281$. (**B**) Western blot analysis showing the expression levels of OPA1 and DRP1 in C2C12 cells. The black arrow indicates the specific protein band. (**C**) Quantitative of relative mitochondrial gene expression levels in C2C12 cells by qPCR ($n = 3$ repeats). Data were analyzed by Student's t test. All error bars indicate mean ± standard deviation. Blank untreated wild-type C2C12 cells, control C2C12 cells transfected with a blank vector as a control for the FOXK2-KO group, FOXK2-KO FOXK2 knockout C2C12 cells, KO + EV FOXK2-KO cells transfected with a blank vector as a control for KO + WT and KO + R215W groups, KO + WT FOXK2-KO cells with overexpression of the human wild-type FOXK2 vector, KO + R215W FOXK2-KO cells with overexpression of the human FOXK2 R215W mutation vector, qPCR quantitative real-time polymerase chain reaction.

 © The Author(s)

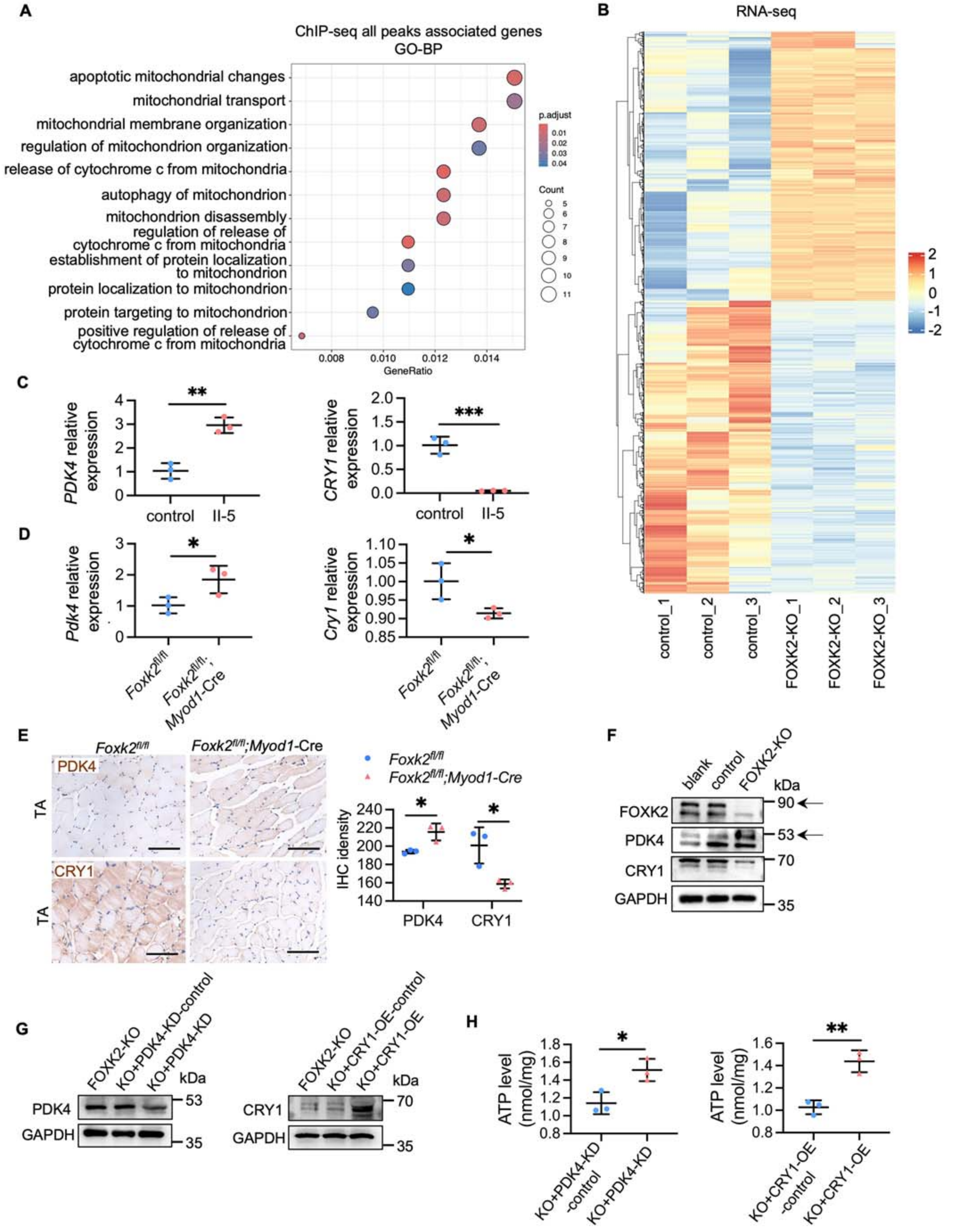
A
ChIP-seq all peaks associated genes
GO-BP
apoptotic mitochondrial changes
mitochondrial transport
mitochondrial membrane organization
regulation of mitochondrion organization
release of cytochrome c from mitochondria
autophagy of mitochondrion
mitochondrion disassembly
regulation of release of cytochrome c from mitochondria
establishment of protein localization to mitochondrion
protein localization to mitochondrion
protein targeting to mitochondrion
positive regulation of release of cytochrome c from mitochondria
0.008 0.010 0.012 0.014
GeneRatio
p.adjust
0.01 0.02 0.03 0.04
Count
5 6 7 8 9 10 11
B
RNA-seq
2 1 0 -1 -2
control_1 control_2 control_3 FOXK2-KO_1 FOXK2-KO_2 FOXK2-KO_3
C
PDK4 relative expression
**
control II-5
CRY1 relative expression
***
control II-5
D
Pdk4 relative expression
*
Foxk2fl/fl Foxk2fl/fl; Myod1-Cre
Cry1 relative expression
*
Foxk2fl/fl Foxk2fl/fl; Myod1-Cre
E
Foxk2fl/fl Foxk2fl/fl;Myod1-Cre
PDK4
CRY1
TA
Foxk2fl/fl
Foxk2fl/fl;Myod1-Cre
IHC idensity
PDK4 CRY1
F
blank control FOXK2-KO
kDa
FOXK2 90
PDK4 53
CRY1 70
GAPDH 35
G
FOXK2-KO KO+PDK4-KD-control KO+PDK4-KD
kDa
PDK4 53
GAPDH 35
FOXK2-KO KO+CRY1-OE-control KO+CRY1-OE
kDa
CRY1 70
GAPDH 35
H
ATP level (nmol/mg)
*
KO+PDK4-KD -control KO+PDK4-KD
**
KO+CRY1-OE -control KO+CRY1-OE

◀ **Figure EV5. Chromatin immunoprecipitation sequencing and RNA sequencing analysis with target gene validation in FOXK2-KO C2C12 cells.**

(**A**) GO analysis (BP) of all genes of which peaks bound by FOXK2, focusing on mitochondrion-related terms. (**B**) Heatmap of DEGs based on RNA-seq in FOXK2-KO C2C12 cells, with upregulated genes shown in red and downregulated genes in blue. (**C**) Quantitative of *PDK4* and *CRY1* gene expression levels in the EM of the proband and a control from pedigree1 by qPCR ($n = 3$ repeats). *P* value: ****P* = 0.0008; ***P* = 0.0020. (**D**) Quantitative of *Pdk4* and *Cry1* gene expression levels in TA muscles of *Foxk2*$^{fl/fl}$ and *Foxk2*$^{fl/fl}$; *Myod1*-Cre littermates at 8 weeks by qPCR ($n = 3$ repeats). *P* value: *Pdk4*, **P* = 0.0487; *Cry1*, **P* = 0.0418. (**E**) IHC staining of PDK4 and CRY1 in the TA muscles of *Foxk2*$^{fl/fl}$ and *Foxk2*$^{fl/fl}$; *Myod1*-Cre littermates at 8 weeks ($n = 3$ areas). The intensity of PDK4 and CRY1 was quantified by InDen/Area using ImageJ software. Scale bars: 100 µm. *P* value: PDK4, **P* = 0.0174; CRY1, **P* = 0.0233. (**F**) Western blot analysis of PDK4 and CRY1 expression level in C2C12 cells. The black arrows indicate the specific protein bands. (**G**) Western blot analysis of PDK4 knockdown and CRY1 overexpression levels in FOXK2-KO C2C12 cells. (**H**) Quantification of ATP contents in C2C12 cells ($n = 3$ repeats). Values were normalized to the total cellular protein level. *P* value: ***P* = 0.0064; **P* = 0.024. Data were analyzed by Student's t test. All error bars indicate mean ± standard deviation. ChIP-seq chromatin immunoprecipitation sequencing, RNA-seq RNA sequencing, GO Gene Ontology, BP biological process, DEGs differentially expressed genes, EM eyelid muscle, TA tibialis anterior, qPCR quantitative real-time polymerase chain reaction, IHC immunohistochemistry, blank untreated wild-type C2C12 cells, control C2C12 cells transfected with a blank vector as a control for the FOXK2-KO group, FOXK2-KO FOXK2 knockout C2C12 cells, KO + PDK4-KD-control FOXK2-KO C2C12 cells transfected with a blank vector as a control for the KO + PDK4-KD group, KO + PDK4-KD FOXK2-KO C2C12 cells transfected with a PDK4 knockdown plasmid, KO + CRY1-OE-control FOXK2-KO C2C12 cells transfected with a blank vector as a control for the KO + CRY1-OE group, KO + CRY1-OE FOXK2-KO C2C12 cells transfected with a CRY1 overexpression plasmid.

© The Author(s)
